# Unexpected Formation of Neutral Alkyl-Ni(II) Complexes Bearing Unsymmetrical *C*,*N*,*N*’‑Pincer Iminopyrrolyl Ligands via Intramolecular C–H Bond Activation: A Distinct Type of Single-Component Ethylene Polymerization Catalysts

**DOI:** 10.1021/acs.inorgchem.5c03794

**Published:** 2025-12-09

**Authors:** Cláudia A. Figueira, Patrícia S. Lopes, Ricardo Meyrelles, Clara S. B. Gomes, Joselaine C. S. Gomes, Luís F. Veiros, Pedro T. Gomes

**Affiliations:** Centro de Química Estrutural, Institute of Molecular Sciences, Departamento de Engenharia Química, Instituto Superior Técnico, Universidade de Lisboa, Av. Rovisco Pais, 1, 1049-001 Lisboa, Portugal

## Abstract

During the development of neutral aryl-nickel­(II) triphenylphosphine complexes bearing bidentate 5-aryl-2-(*N*-aryl)­formiminopyrrolyl ligands as single-component ethylene polymerization catalysts, an unexpected intramolecular C–H activation was observed. Reaction of sodium salts of ligand precursors 5-(2,6-Me_2_C_6_H_3_)-2-(*N*-2,6-R_2_C_6_H_3_)­formiminopyrrole (R = Me **I**, *i*Pr **II**, 3,5-(CF_3_)_2_C_6_H_3_ **III**) with *trans*-[Ni­(Ar)­(PPh_3_)_2_Cl] (Ar = *o*-C_6_H_4_Cl or C_6_H_5_) afforded unsymmetrical tridentate *C*,*N*,*N’*-pincer iminopyrrolyl nickel­(II) complexes [Ni­{κ^3^ *C,N,N*’-5-(2′-CH_2_–6-MeC_6_H_3_)-NC_4_H_2_-2-C­(H)=N-2,6-R_2_C_6_H_3_}­(PPh_3_)] (R = Me **1**, *i*Pr **2**, 3,5-(CF_3_)_2_C_6_H_3_ **3**). Complexes **1**-**3** and their ligand precursors were characterized using multinuclear NMR, X-ray diffraction, and elemental analysis. NMR revealed initial coexistence of tridentate *C,N*,*N’*- and bidentate *N*,*N’*-iminopyrrolyl Ni­(II) complexes; at higher temperature, only the tridentate remain. DFT calculations indicated that cyclometalation proceeds via an agostic C–H interaction with the Ni center, enabling C–H activation. Thermally stable **1**-**3** were evaluated as single-component catalysts for ethylene polymerization (25–50 °C, 3–15 bar), both with and without Ni­(COD)_2_ as phosphine scavenger. The resulting monomodal hyperbranched polyethylenes (HBPEs) displayed higher molecular weights and branching than those from parent bidentate 2-iminopyrrolyl phenyl-Ni­(II) catalysts. These novel tridentate *C*,*N*,*N’*-pincer systems exhibited significantly enhanced activity and robustness. The HBPEs produced are promising as synthetic base oils for high-performance lubricants and pour-point depressants.

## Graphical abstract

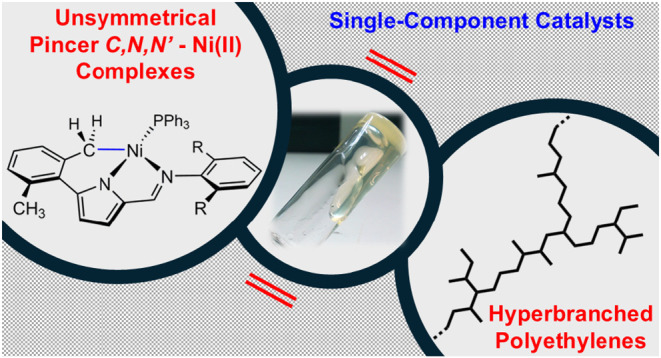

## Introduction

Over the past two decades, pincer ligands have emerged as one of the primary classes of polydentate ligands in modern coordination/organometallic chemistry and homogeneous catalysis. Their popularity can be attributed not only to the relatively easy tuning of their electronic and steric properties but also to the enhanced thermodynamic and kinetic robustness that these tridentate ligands confer on their metal complexes.^1^ Most of these ligands are structurally symmetrical. However, unsymmetrical pincer ligands have been gaining significant importance, particularly in catalysis, due to the potential for decoordination of the more labile donor arm.^2^ This process creates a free coordination site while maintaining the bond between the ligand and the metal center through the remaining two donor arms.

Group 10 metal complexes bearing unsymmetrical tridentate *C,N,N’* pincer ligands are relatively uncommon in the literature. In general, they can be synthesized through cyclometalation via three main pathways: (a) intramolecular oxidative addition of an aryl halide substituent on an *N,N’*-bidentate ligand precursor to low-valent metal starting materials (e.g., Ni(0), Pd(0) and Pt­(II)); (b) C–H activation of a suitably positioned alkyl or aryl group on a *N,N’*-bidentate ligand metal complex by an external base (e.g., amines, acetate or alkoxide salts); and (c) intramolecular C–H activation of a suitably positioned alkyl or aryl group on a *N,N’*-bidentate ligand metal complex by a coordinated basic monoanionic ligand (e.g., alkoxide, acetate, alkyl or aryl) situated *cis* to the *N,N′*-chelate.^3–7^ In the latter case (c), the reactive C–H bond is usually situated either at the *ortho*-position of a phenyl (or other aryl) group or on an alkyl substituent (e.g., Me, *i*Pr or *t*Bu) adjacent to or bonded to an *ortho*-aryl carbon. Such spatial arrangements bring the C–H bond into close proximity with the metal center, thereby facilitating C–H activation and subsequent cyclometalation to form the tridentate complex, typically accompanied by the elimination of the corresponding protonated ligand as e.g., an alcohol, carboxylic acid, alkane or arene.

Examples of *C*,*N,N’*-ligand complexes of platinum and palladium resulting from cyclometalation through aromatic *ortho* C–H activation^5,6b,6c^ are shown in Chart 1 (e.g., **A**,^4a^ **B**,^6b^ and **C** ^6c^). *C*,*N,N’*-ligand palladium complexes obtained by alkyl C–H activation^6^ are less common and are formed predominantly by the third pathway mentioned above. Most of these examples are also represented in Chart 1 (e.g., **D**,^6b^ **E**,^6b^ **F**,^6c^ **G**,^6a^ **H**,^6d^ and **I** ^6e^). By contrast, *C,N,N′*-ligand nickel complexes are much less frequently reported. The first example of a nickel complex bearing an unsymmetrical pincer *C,N,N′*-ligand was described by tom Dieck et al., in the course of attempting to arylate a NiBr_2_ adduct of neutral bis­(2,6-diisopropylphenyl)­diazabutadiene (**K**).^7a^ A second example was reported by Bazan and co-workers in their study of pyridinecarboxamidato nickel complexes (**L**).^7b^ Subsequently, Nemykin, Du et al. reported nickel complexes with *C*,*N*,*N’*-tridentate ligands based on β-diketiminato-type frameworks, formed through intramolecular C–H activation of a methyl group on a 2,6-dimethylaniline moiety (**M**).^7c^

**Chart 1:**
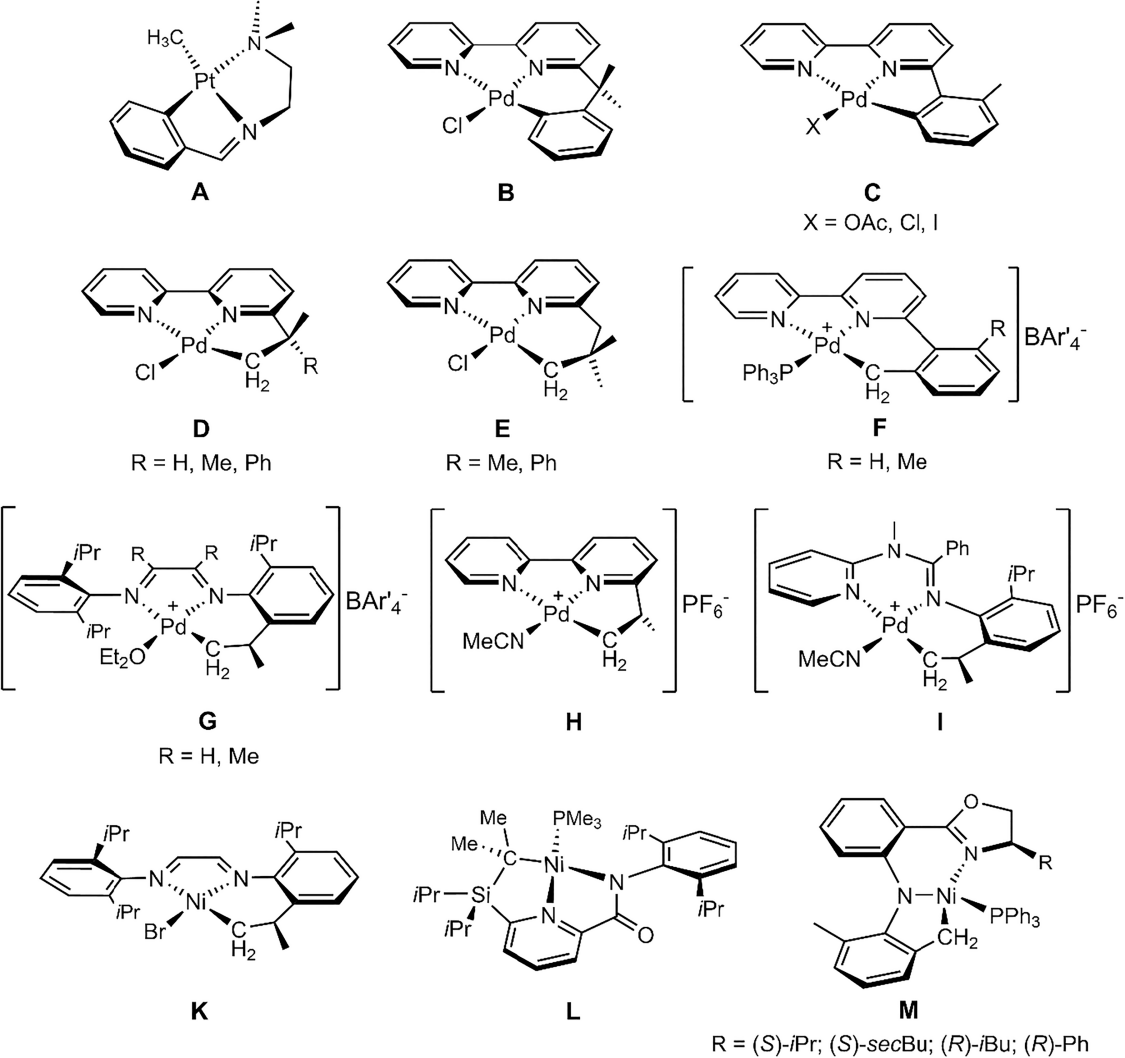
Examples of Pt and Pd Complexes Obtained by Cyclometallation through Aromatic *Ortho* C–H Activation (**A–C**) and of Pd and Ni Complexes Obtained by Cyclometallation through Alkyl C–H activation (**D–I** and **K–M**)

Stable transition-metal complexes of nickel or palladium featuring metal–carbon bonds are promising candidates for well-defined, single-component homogeneous catalysts in ethylene polymerization. Such complexes typically possess *N,N*- or *N,O*-bidentate ligands that stabilize the metal center, while the remaining coordination sites are available for binding the growing polymer chain and incoming ethylene monomers.^8^ However, reports on the use of tridentate ligands, including pincer-type frameworks, for this purpose are virtually nonexistent. Among the nickel complexes with tridentate *C,N,N′*-pincer ligands **K**-**M** (Chart 1), only complex **L** was tested in this reaction, demonstrating ethylene oligomerization activity only when a 10-fold excess of B­(C_6_F_5_)_3_, used as a phosphine scavenger, was employed.^7c^

We have been developing first row late-transition metal complexes featuring the 2-iminopyrrolyl framework as bidentate chelating ligand.^9^ This research includes the study of nickel derivatives as aluminum-free homogeneous catalysts for ethylene polymerization. Initially, the use of simple 2-(*N*-arylimino)­pyrrolyl ligands resulted in particularly low catalytic activity and the production of low-molecular-weight ethylene oligomers.^10^ This led us to explore a new family of aryl-nickel­(II) complexes that incorporate sterically crowded 2-iminopyrrolyl ligands, in order to enhance the catalysts performance through the protection of the metal center and increase the molecular weights of the polyethylene (PE) products. To this end, we synthesized several 5-aryl-2-(*N*-aryl)­formiminopyrrole ligand precursors (Scheme 1, **HL**), by condensing a primary amine with corresponding 5-aryl-2-formylpyrroles precursors,^11^ where both aryl rings were substituted with groups of varying steric and/or electronic properties. We then reacted their sodium salts with the starting materials *trans*-[Ni­(Ar)­(PPh_3_)_2_Cl] (where Ar = C_6_H_5_ or *o*-C_6_H_4_Cl).^12^ For R^1^ = H, *i*Pr, this approach yielded a family of thermally stable bidentate 2-iminopyrrolyl nickel complexes with the general formula [Ni­(Ar)­{κ^2^ *N,N*’-5-(aryl)-NC_4_H_2_-2-C­(H)=N-2,6-(aryl)}­(PPh_3_)], which display distorted square planar structures around the Ni center (Scheme 1, **Ni**_**B**_). All these complexes served as well-defined single-component catalysts for the oligo-/polymerization of ethylene to hyperbranched oligo-/polyethylenes.^12^

**Scheme 1:**
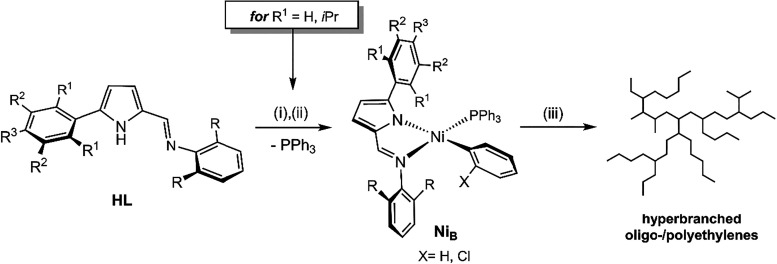
(i) NaH, THF; (ii) *trans*-[Ni­(C_6_H_4_X)­(PPh_3_)_2_Cl] (X = H, *o*-Cl), toluene, −40 °C to r.t.; (iii) Ni_B_ (10^–2^ mmol), ethylene (3-15 bar), [Ni­(COD)_2_] (0 or 2 equiv), 25-50 °C, toluene (50 mL)^12^

In this work, the reaction of the sodium salt of the specific ligand precursors **HL** with R_1_ = CH_3_ and R_2_ = R_3_= H unexpectedly led to the formation of neutral alkyl-Ni­(II) complexes with unsymmetrical *C*,*N*,*N’*-pincer iminopyrrolyl ligands. This occurred via intramolecular C–H activation, accompanied by the elimination of arene C_6_H_5_X (X= Cl, H) – see Scheme 2 in the next section. The synthesis and full characterization of these pincer complexes were carried out experimentally, and their formation was rationalized using DFT calculations. The novel tridentate complexes act as well-defined single-component catalysts for the polymerization of ethylene, which, to the best of our knowledge, is the first time a *C*,*N*,*N’*-pincer nickel complex has exhibited effective catalytic activity in this type of reaction.

**Scheme 2:**
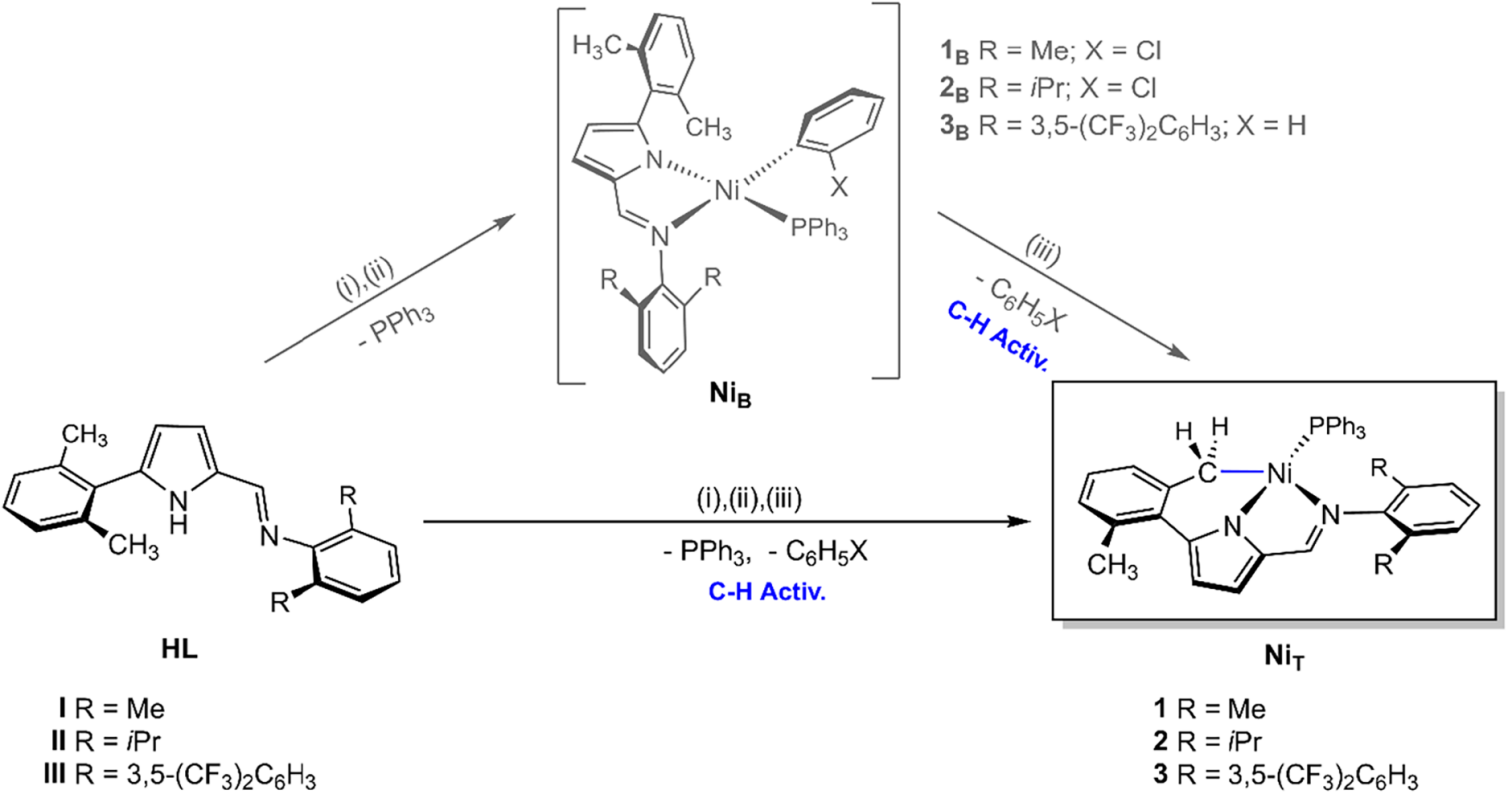
(i) NaH, THF; (ii) *trans*-[Ni­(*o*-C_6_H_4_X)­(PPh_3_)_2_Cl] (X = H, Cl), toluene, −40 °C to r.t.; (iii) from r.t. to 45 °C (1), 30 °C (2), 65 °C (3), for 12 h

## Results and Discussion

### Synthesis and NMR Characterization of the Tridentate *C*,*N*,*N’*-Pincer Nickel Complexes

In the current study, the previously reported 5-(2,6-dimethylphenyl)-2-formylpyrrole (i.e., R^1^ = Me, R_2_ = R_3_ = H)^11^ was reacted with two different 2,6-di­(R)­anilines (R = Me and 3,5-(CF_3_)_2_C_6_H_3_) to produce the new **HL** condensation products, **I** and **III** (Scheme 2), yielding 74 and 43% respectively, with full characterization provided in the Supporting Information (SI).

These 2-iminopyrroles, along with the already described **II**,^9a^ served as the ligand precursors in this work. After deprotonation of **I**–**III** with sodium hydride, the resulting sodium salts **I**_**Na**_–**III**_**Na**_ were reacted with the complex *trans*-[Ni­(*o*-C_6_H_4_X)­(PPh_3_)_2_Cl] (X= Cl, H) at −40 °C, with subsequent warming up to room temperature, followed by heating to temperatures of 45, 30, and 65 °C, for 12 h, for ligand precursors **I**, **II** and **III**, respectively. Surprisingly, instead of forming the anticipated bidentate aryl-nickel­(II) complexes (**Ni**_**B**_), an intramolecular C–H activation took place, leading to cyclometalation at one of the *ortho*-methyl groups on the 5-aryl substituent. This reaction yielded a new family of thermally stable tridentate *C*,*N*,*N’*-pincer iminopyrrolyl nickel­(II) complexes (**1–3**), featuring progressively bulkier substituents (see next subsection). The transformation occurred with the concomitant elimination of the arene C_6_H_5_X (Scheme 2, **Ni**_**T**_). Complexes **1**–**3** were isolated as solids in 65, 50 and 61% yields, respectively.

The tridentate complexes **1**–**3** were fully characterized by multinuclear NMR spectroscopy (^1^H, ^13^C­{^1^H} and ^31^P­{^1^H}) (Figures S1–S7, SI). For this purpose, we employed CD_2_Cl_2_ as the solvent for recording the NMR spectra of complexes **1**-**3**, despite the well-documented detrimental effect of aliphatic chlorinated solvents on Ni­(II)-alkyl species.^13^ The use of CD_2_Cl_2_ offers a broader and cleaner spectral window for the numerous aromatic resonances in these asymmetric complexes, compared to aromatic deuterated solvents such as C_6_D_6_ or toluene-*d*_8_. Interestingly, complexes **1**-**3** exhibit markedly higher stability in CD_2_Cl_2_ than most Ni­(II)-alkyl derivatives, owing to the substantial steric protection surrounding the Ni-alkyl bond, which mitigates decomposition via protonolysis or chlorination. All three complexes, including the least sterically encumbered complex **1**, remain sufficiently stable in solution to allow acquisition of both ^1^H and even ^13^C­{^1^H} NMR spectra, although traces of decomposition products may eventually be detected. The ^1^H and ^13^C­{^1^H} spectra show characteristic CH_2_ doublets at 2.1–2.3 ppm (^3^ *J*_HP_= 11.1–11.4 Hz) and 24.1–24.8 ppm (^2^ *J*_CP_= 20.9–21.8 Hz), together with a CH_3_ singlet at 2.59–2.61 ppm and 23.7–24.0, respectively, arising from the 5-aryl-pyrrolyl substituent. NOESY correlations (Figure S3b, SI) confirm that these two distinct resonances correspond to CH_2_ and CH_3_ groups on the same ring. The ^31^P­{^1^H} spectra exhibit resonances at 33–36 ppm, significantly downfield from those of related iminopyrrolyl bidentate complexes (12–20 ppm),^12^ in line with other PPh_3_-coordinated *C*,*N*,*N’*-pincer nickel­(II) systems.^7c^ Long-range ^31^P *trans* couplings are evident in the *trans*-disposed pyrrolyl ring, causing the ^1^H signals to appear as multiplets (rather than the usual doublets) and the ^13^C­{^1^H} signals as doublets, with ^5^ *J*_HP_ (pyrrolyl H3 and H4), ^3^ *J*_CP_ (pyrrolyl C2 and C5), and ^4^ *J*_CP_ (pyrrolyl C3 and C4). Similar ^31^P coupling is observed in the imine carbons (N = *C*H) over three-bonds (^3^ *J*_CP_), giving rise to doublets at ca. 162 ppm. Although overlapped in all complexes, the ^1^H resonances of the imine protons (N = C*H*) appear as singlets rather than the doublets typical of the related bidentate complexes, where the imine group is positioned *trans* to the PPh_3_ coligand.^12^

### X-ray Diffraction Structural Studies

Crystals suitable for X-ray Diffraction were obtained for complexes **1–3**, allowing the determination of their molecular structures. Complex **1** crystallizes in the triclinic crystal system (space group *P*-1), whereas complexes **2** and **3** crystallize in the monoclinic crystal system, in space groups *P*2_1_/*n* and *P*2_1_/*c*, respectively (Table S2, SI).

The molecular structures of complexes **1–3** are depicted in Figure 1, and selected bond distances (Å) and angles (°) are summarized in Tables S4 and S5 (SI). The X-ray Diffraction analyses confirm the presence of a cyclometalated *C*,*N*,*N’*-pincer iminopyrrolyl ligand and a PPh_3_ coligand in the nickel coordination sphere, while the *o*-chlorophenyl or phenyl ligands from the starting materials *trans*-[Ni­(*o*-C_6_H_4_X)­(PPh_3_)_2_Cl] (X= Cl, H) are absent, as previously inferred from NMR data. The corresponding ligand precursors **I**-**III** were also characterized by X-ray Diffraction (Tables S1 and S3, and Figures S8 and S9, SI).

**Figure 1:**
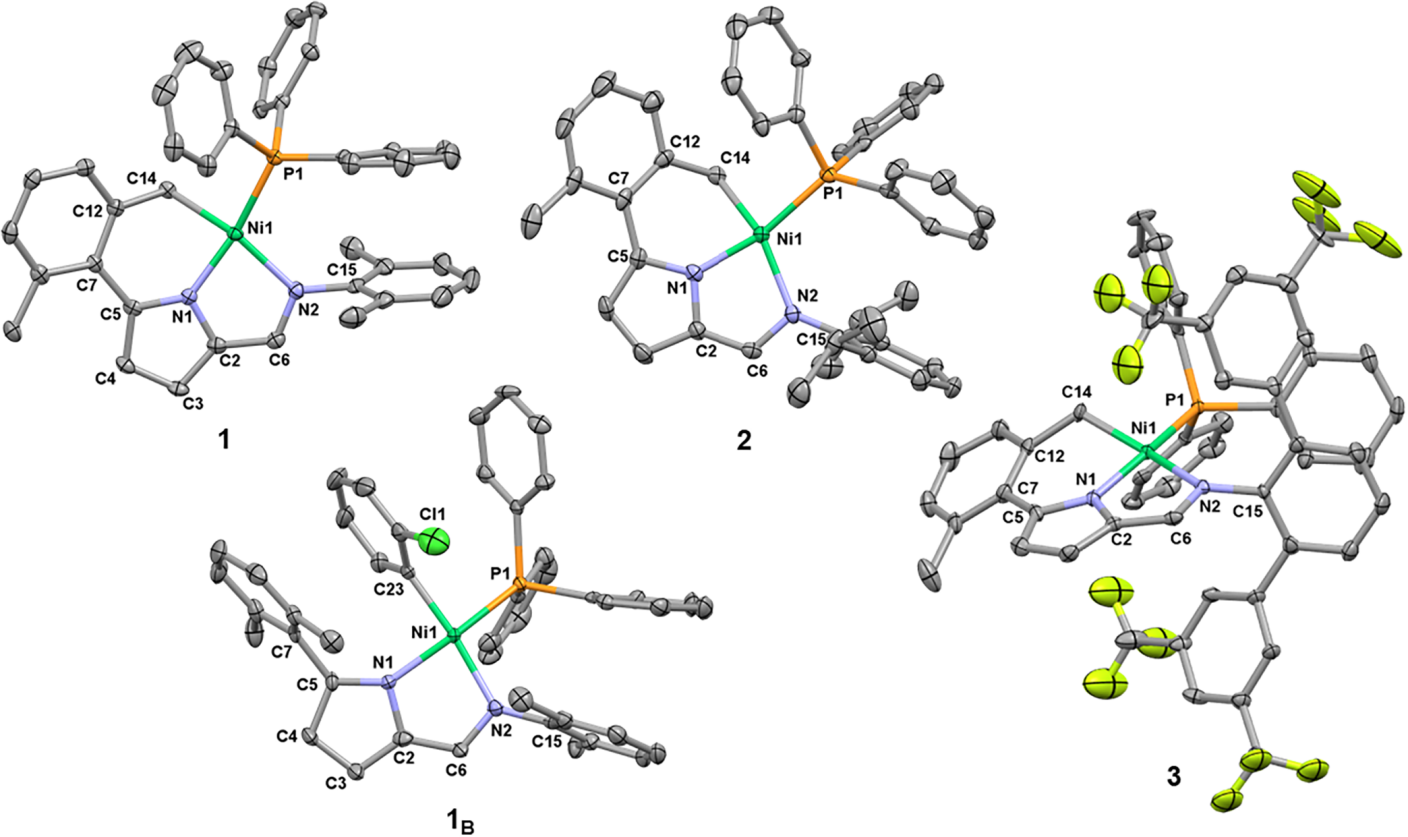
Perspective views of the molecular structures of complexes **1–3** and **1**_**B**_ using 50% probability level ellipsoids for **1**, **2** and **1**_**B**_ and 35% for **3**. All the hydrogen atoms, and the cocrystallized PPh_3_ in **1**, were omitted for clarity. All the crystallographic data (Table S2) and selected bond distances and angles (Tables S4 and S5) are listed in the SI.

In general, the geometries of the iminopyrrolyl ligands in complexes **1**-**3** closely resemble those of their corresponding neutral precursors **I**-**III**, with comparable bond lengths and internal angles (Table S3, SI). The most significant structural change upon coordination arises from the markedly reduced dihedral angles between the 5-aryl substituent and the pyrrolyl ring. Coordination of the benzylic CH_2_ group to the nickel center enforces a more coplanar arrangement between these two fragments, decreasing the inter-ring dihedral angles from *ca*. 56–64° in the free ligands to 22–29° in the complexes (cf. Tables S3 and S5).

In the molecular structures of **1**-**3**, the tridentate ligands form a new *C*,*N* six-membered chelate ring (Ni1–N1–C5–C7–C12-C14) in addition to the typical *N*,*N’* five-membered chelate (Ni1–N1–C2–C6-N2). The *N*,*N’* ring is essentially planar, whereas the *C*,*N* ring adopts a distorted envelope-like conformation, as exemplified by complex **2** in Figure S10 (SI). Coordination of the methylene carbon to nickel shifts the PPh_3_ coligand to a site *trans* to the pyrrolyl ring, in agreement with NMR observations.

The coordination environment around the nickel center is best described as square planar with a moderate tetrahedral distortion, as indicated by τ_4_ parameters^14^ of 0.23, 0.30 and 0.26, and dihedral angles between N1–Ni1–N2 and P1–Ni1–C14 planes of 18.42, 25.01 and 19.87° for complexes **1**-**3**, respectively. Such distortions are typical of aryl-nickel­(II) systems bearing sterically demanding iminopyrrolyl chelates.^12^ In contrast to their bidentate analogues, where distortion occurs mainly at the PPh_3_ coordination site, the methylene donor in these tridentate complexes coordination contributes most significantly to the deviation from planarity. This, combined with the less sterically encumbered position of the phosphine ligand, results in shorter Ni1–P1 bond lengths (2.14–2.16 Å) and larger N1–Ni1–P1 *trans* angles (165.84° in **1**, 162.38° in **2**, and 164.70° in **3**). The near coplanarity of the P atom with the iminopyrrolyl plane likely accounts for the increased ^31^P coupling constants observed in the ^1^H and ^13^C­{^1^H} NMR spectra. Notably, the molecular structure of complex **1** revealed a 1:1 cosolvate with free PPh_3_, corroborated by ^1^H and ^31^P­{^1^H} NMR spectra. Washing the crystals with *n*-hexane and extended drying under vacuum reduced the PPh_3_ content to a 1:0.6 ratio.

Complexes **1**-**3** feature *C,N,N’*-tridentate ligands with progressively increasing steric bulk, which can be quantitatively assessed through the calculation of the percentage of buried volume (% V_Bur_)^15^ contributed by these ligands within a sphere of arbitrary radius centered on the metal. For example, for a sphere of radius 7 Å, which encompasses second coordination sphere effects,^16^ the calculated %V_Bur_ values for the *C,N,N′*-iminopyrrolyl ligands in complexes **1**-**3** are 24.1, 29.2 and 38.4, respectively. These values were determined for radii ranging from 3.5 Å (reflecting mainly first coordination sphere effects) to 9 Å (Table S6 and Figure S11, SI) using freely available software tools (see Experimental Section). Visualization of the calculated buried volumes for Ni complexes **1**-**3** and corresponding steric maps for sphere radii of 3.5 and 7 Å are shown in Figure S12­(SI). These maps reveal that the least sterically hindered quadrants in these complexes correspond to the regions where the benzylic arms coordinate to the Ni center (NE in **1**, SE in **2**, and SW in **3**).

During the workup of one of the first reactions between the starting material *trans*-[Ni­(*o*-C_6_H_4_Cl)­(PPh_3_)_2_Cl] (**SM**) and sodium salt **I**_**Na**_ at −30 °C, under conditions previously established for the synthesis of bidentate **Ni**_**B**_ complexes (Scheme 1),^12^ a reaction intermediate was fortuitously isolated as a single crystal. This species was identified as the kinetically unstable bidentate precursor **1**_**B**_ of complex **1** (Scheme 2 and Figure 1). Attempts to isolate pure and quantifiable amounts of any **Ni**_**B**_ intermediate (**1**_**B**_-**3**_**B**_) proved unsuccessful due to their intrinsic instability and rapid conversion to the corresponding tridentate **Ni**_**T**_ complexes, even at low temperature. The molecular structure of **1**_**B**_ closely resembles those of reported bidentate 2-iminopyrrolyl nickel­(II) complexes, both in bond metrics and in the structural changes of the chelating ligand upon coordination (Tables S4 and S5, SI).^12^ Remarkably, the PPh_3_ coligand in **1**_**B**_ adopts a *cis* configuration relative to the imine moiety – an uncommon feature among such complexes – which typically display the *trans* isomer.^12^ The *cis* geometry, similar to that observed for the 5-(anthracen-9-yl)-2-(*N*-2,6-diisopropylphenyl)-substituted analogue,^12b^ results in a slightly longer Ni1–P1 bond (2.187 Å, cf. 2.19–2.20 Å for the anthracenyl analogue, vs ca. 2.17 Å in *trans* isomers) and produces wider *trans* angles (N1–Ni1–P1 = 164.11°, N2–Ni1–C*_ipso_* = 173.87°). The geometry is the least distorted square-planar arrangement in this family (τ_4_ = 0.16, dihedral angle between the N1–Ni1–N2 and P1–Ni1–C*_ipso_* planes = 15°),^12^ closely approaching ideal planarity.

### NMR Studies on the Formation of Tridentate *C*,*N*,*N’*-Pincer Nickel Complexes

The formation of the tridentate complexes **2** (Scheme 2) was examined by detailed NMR tube-scale experiments conducted in toluene-*d*_8_. Variable-temperature (VT) ^31^P­{^1^H} and ^1^H NMR spectra of the stoichiometric (1:1) reaction between sodium salt **II**_**Na**_ and the nickel precursor *trans*-[Ni­(*o*-C_6_H_4_Cl)­(PPh_3_)_2_Cl] (**SM**), in toluene-*d*_8_, were recorded from −40 to 50 °C. Clear evidence for the transformation was obtained from the VT-^31^P­{^1^H} NMR monitoring experiment of the reaction **II**_**Na**_ + **SM** → **2** (Figure 2), showing the progressive formation of complex **2** between 0 and 50 °C, in 10 °C increments, with no reaction observed below 0 °C.

**Figure 2:**
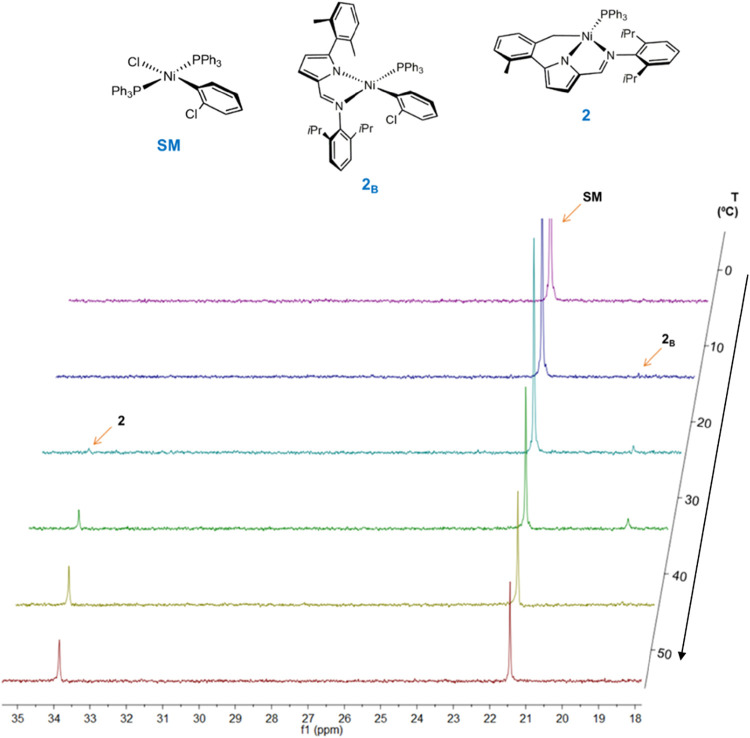
VT-^31^P­{^1^H} NMR spectra of the reaction **II**_**Na**_ + **SM** → **2** in toluene-*d*_8_, recorded at increasing temperatures. The three distinct resonances are labeled as **SM** (starting material *trans*-[Ni­(*o*-C_6_H_4_Cl)­(PPh_3_)_2_Cl]), **2**_**B**_ (transient bidentate intermediate) and **2** (tridentate complex).

Upon increasing the temperature to 10–20 °C, two weak resonances emerge in the ^31^P­{^1^H} spectrum: one at ca. 19 ppm, characteristic of the bidentate intermediate **2**_**B**_, and another at 34 ppm, corresponding to the tridentate complex **2**.^7c^ Both species coexist in solution up to ca. 40 °C. Above this temperature, the **2**_**B**_ intermediate resonance gradually disappears while the signal intensity of tridentate **2** intensifies, indicating its predominant formation.

The corresponding VT-^1^H NMR spectra are complex in the aromatic region due to overlapping resonances from reactants and products. However, in the aliphatic region (Figure S13, SI), the evolution of the methyl and isopropyl resonances from the sodium salt **II**_**Na**_, the bidentate intermediate **2**_**B**_ and the tridentate complex **2** mirrors the ^31^P­{^1^H} data.

In the kinetically unstable intermediate **2**_**B**_, the PPh_3_ coligand appears to adopt a *cis* configuration relative to the imino moiety, as indicated by its ^31^P­{^1^H} resonance at 18.9 ppm, closely matching the value reported for the *cis* isomer of the 5-(anthracen-9-yl)-substituted bidentate analogue.^12b^ This assignment is consistent with the configuration determined for the related intermediate **1**_**B**_ (see above). In contrast, most bidentate 2-iminopyrrolyl nickel­(II) complexes display the *trans* configuration, with ^31^P­{^1^H} resonances in the 12–17 ppm range.^12^

Additionally, the reaction was monitored for 13 h at constant temperatures of 25, 30, 35, and 45 °C. At 25 °C, a small amount of the transient bidentate intermediate **2**_**B**_ was detected together with the tridentate complex **2** within the first hour (Figure S14, SI). The intermediate was gradually converted into **2** over time. At higher temperatures, only the tridentate complex was observed from the start, indicating that its formation is both kinetically and thermodynamically favored under these conditions.

A comparable behavior was observed in the reaction between sodium salt **I**_**Na**_ and **SM**, leading to complex **1**, in which **1**_**B**_ and **1** coexist up to 35 °C, whereas at 45 °C only the tridentate complex is detected. In contrast, a similar study on a bidentate **Ni**_**B**_ complex supported by a 2-iminopyrrolyl ligand bearing R = R_1_ = R_3_ = *i*Pr, R_2_ = H, X = H (see Scheme 1)^12a^ revealed this species to be thermally stable and resistant to C–H activation of the −CHMe_2_ groups at the 2,6 positions of the 5-phenyl ring, likely due to steric hindrance, even after prolonged heating.

Further NMR-tube scale experiments showed that the addition of PPh_3_ (2 equiv) to an equimolar mixture of *trans*-[Ni­(*o*-C_6_H_4_Cl)­(PPh_3_)_2_Cl] and ligand precursor **II** significantly retards the formation of complex **2** (see Figure S15, SI; cf. Figure 2), indicating that phosphine dissociation is involved in the rate-determining step of the reaction.

From these combined NMR, the optimal temperatures for the synthesis of the tridentate complexes were determined to be 45 °C for **1**, 30 °C for **2**, and 65 °C for the bulkier complex **3**. This trend indicates that the propensity for intramolecular C–H activation qualitatively increases in the order **3** < **1** < **2**.

### Mechanistic Insights into the Intramolecular C–H Activation and Precatalysts Formation

The C–H activation of the iminopyrrolyl ligand leading to the formation of the tridentate complex **2** was investigated with DFT methods aiming a plausible mechanism (see Experimental Section for details). The obtained Gibbs free energy profile is presented in Figure 3, starting from the Ni­(II) bisphospine complex **A**. This complex is formed from the starting material, *trans*-[Ni­(*o*-C_6_H_4_Cl)­(PPh_3_)_2_Cl], after an initial substitution step between the chloride ligand and the sodium salt **II**_**Na**_, with elimination of NaCl. Dissociation of one phosphine ligand from **A** leads to intermediate **B**. In this complex, the metal center presents a square planar geometry featuring an agostic C–H interaction between the metal center and one methyl group of the 2,6-dimethylphenyl substituent of the coordinated pyrrolyl ligand. Although the formation of **B** is endergonic (Δ*G*(**A**→**B**) = 8.8 kcal/mol), it opens two distinct pathways to the formation of the experimentally observed tridentate Ni complex **2**. The lowest-energy pathway, highlighted in blue in Figure 3, starts with a Ni-mediated σ-bond metathesis at **TS**_**BC**_. In this step, the agostic C–H bond is cleaved, forming a new Ni–C and an *o*–H-C_6_H_4_Cl bond, which results in the release of chlorobenzene and in the formation of bidentate Ni intermediate **C**, presenting a T-shaped geometry. Finally, an exergonic rotation of the σ–C-C bond of the iminopyrrolyl ligand, step **C**→**D**, enables the coordination of the imine to the metal, yielding the thermodynamically stable tridentate complex **D** (Δ*G*(**C**→**D**) = −16.1 kcal/mol) with a moderately low activation barrier of 15.8 kcal/mol. The overall formation of the tridentate complex **D** through the proposed C–H activation mechanism presents an apparent activation barrier of 24.8 kcal/mol, determined between the reactant complex **A** and the transition state **TS**_**BC**_, which is consistent with experimental conditions (observed formation of this species at 30 °C).

**Figure 3:**
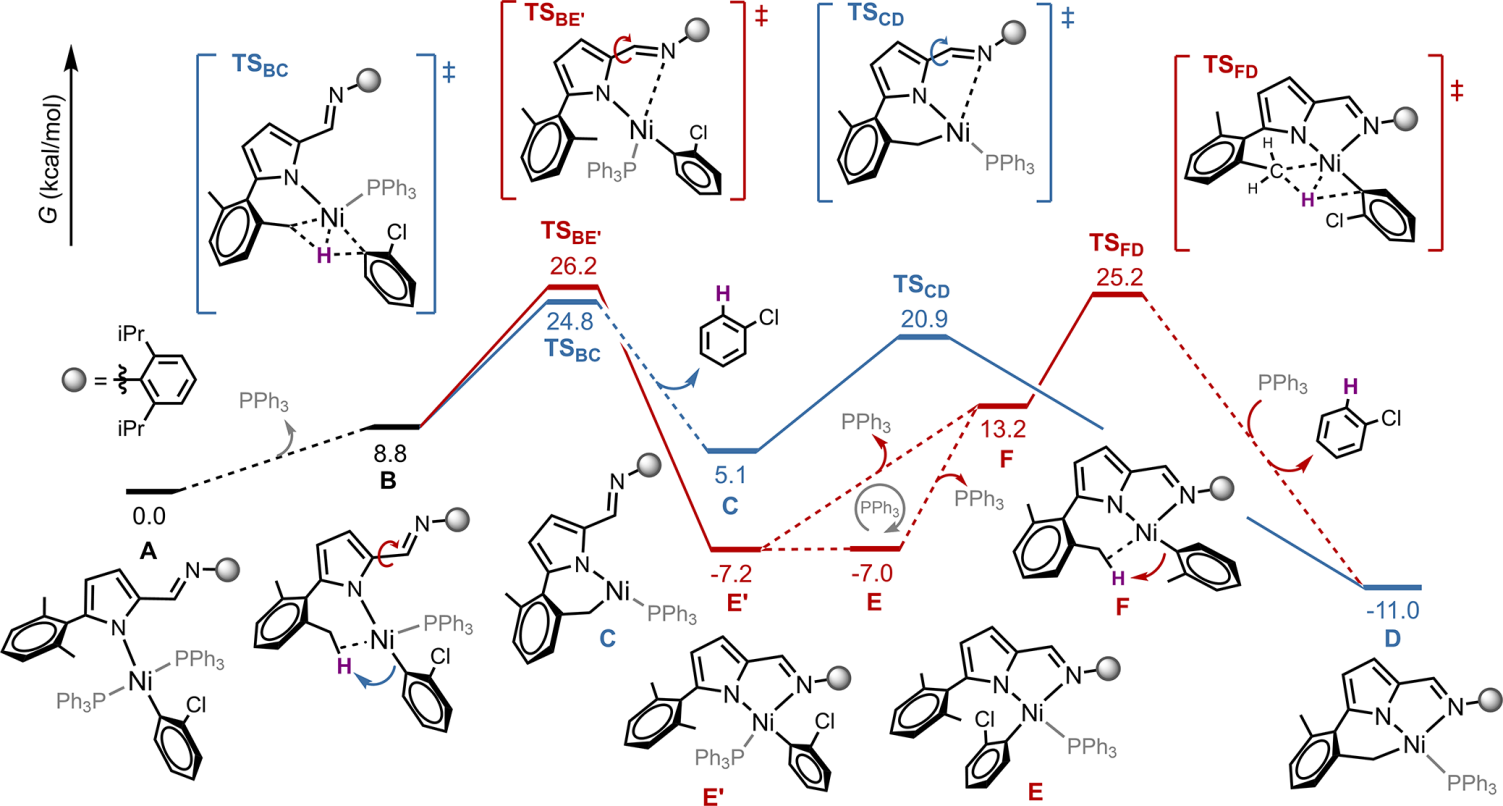
Computed Gibbs free energy profiles for possible pathways leading to the formation of tridentate Ni complex **D**, corresponding to the experimentally observed product **2**. Relative Gibbs free energies are presented in kcal/mol (298 K).

Alternatively, from structure **B**, a rotation of the σ–C-C bond of the iminopyrrolyl ligand could occur first, forming bidentate Ni complex **E’** (pathway highlighted in red). This step, **B**→**E’**, has an activation barrier of 26.2 kcal/mol, only 1.4 kcal/mol higher than step **B**→**C**, indicating that both steps can occur simultaneously under the experimental conditions. Along the pathway highlighted in red, intermediate **E’** presents the *ortho-*chlorophenyl ligand *trans* to the pyrrolyl ligand and can be converted into the bidentate complex **E**, which is isoenergetic to **E’** (Δ*G*(**E’**→**E**) = 0.2 kcal/mol). **E** presents the *ortho-*chlorophenyl ligand *cis-* to the pyrrolyl and corresponds to the experimentally detected complex **2**_**B**_. This isomerization can occur through a reversible dissociation of the second phosphine, involving the formation of the phosphine-free intermediate **F**, in which the Ni center is stabilized by an agostic C–H bond. This interaction enables a Ni-mediated σ-bond metathesis, producing the tridentate complex **D** upon phosphine recoordination and release of chlorobenzene. Following the red pathway, the conversion of the bidentate Ni complex **E** into **D** can therefore be achieved via an apparent activation barrier of 32.4 kcal/mol, obtained between **E’** and **TS**_**FD**_. Alternatively, **D** can be formed if **E** isomerizes to **E’** and step **B**→**E’** is reversed (**E’**→**B**), from which the pathway highlighted in blue is followed from **B** to **D**, presenting an apparent activation barrier of 33.4 kcal/mol, obtained between **E’** and **TS**_**BE’**_. The small difference between the activation barriers of the pathways does not result in a significant kinetic preference for either route, indicating that both can occur under experimental conditions. These results are therefore in line with the observed thermally induced conversion of **2**_**B**_ into **2**. Other C–H activation pathways from bidentate Ni complexes were also explored but exhibited prohibitively high activation barriers, mostly resulting from geometric constraints imposed by the high structural rigidity of the studied 2-iminopyrrolyl ligands (see SI for details and Figure S16).

### Catalytic Studies on the Polymerization of Ethylene Promoted by Complexes 1–3. Mass and Microstructural Characterization of the Resulting Polyethylenes

Building on previous studies that used stable bidentate (5-aryl-2-(*N*-aryl)­formiminopyrrolyl) aryl-nickel­(II) precatalysts as well-defined single-component catalysts for ethylene polymerization,^12^ we tested the novel complexes **1**-**3** for this catalytic reaction. Unlike the tridentate *C*,*N*,*N’*-pincer nickel complexes **F**-**H** reported in the literature (Chart 1), the new tridentate complexes **1**-**3** proved to be active toward ethylene polymerization. To the best of our knowledge, this is the first time that a *C*,*N*,*N’-*pincer nickel complex effectively promoted the polymerization of ethylene. All catalytic results, including the molar mass characteristics and the degrees of branching of the produced PEs, are presented in Table 1. A summary chart comparing catalytic activities and the molecular weights of the produced PEs is presented in Figure 4.

**Table 1:**
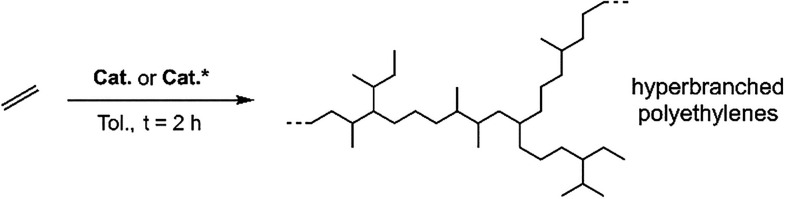
Polymerization of Ethylene Catalyzed by the Tridentate Complexes **1**-**3**, and Characterization of the Corresponding Synthesized Polymers in All the Tested Conditions^a^

entry	Cat. or Cat.*^,^ ^b^	*P*_abs_ (bar)	T (°C)	PE (g)	activity (kg_PE_/ (mol_Ni_·h·bar))	*M*_n_ (GPC)^c^ (g/mol)	*M*_n_ (NMR)^d^ (g/mol)	*Đ* ^c^,^e^	*N* ^f^
1	1*	3	25	0.91	15.24	4300	2800	1.5	122
2	1*	3	50	0.12	1.93	3900	-	1.6	-
3	**1**	9	25	0.21	1.14	4700	-	1.6	-
4	**1**	9	50	1.90	10.53	3000	1900	1.5	113
5	1*	9	25	4.98	25.41	4900	3200	1.5	120
6	1*	9	50	4.04	27.69	3400	2500	1.7	115
7	**1**	15	25	0.38	1.26	4900	-	1.4	-
8	**1**	15	50	1.64	5.46	3500	3700	1.4	122
9	1*	15	25	5.03	16.78	5600	3300	1.4	121
10	1*	15	50	6.77	22.57	4400	3300	1.5	119
11	**2**	3	25	0.45	7.51	7600	6100	1.4	129
12	**2**	3	50	1.41	23.46	4700	3700	1.9	122
13	2*	3	25	1.71	28.55	7700	6600	1.6	133
14	2*	3	50	2.01	33.49	6400	3900	1.9	121
15	**2**	9	25	0.31	1.75	7800	4100	1.6	131
16	**2**	9	50	2.37	13.15	5700	4200	1.6	120
17	2*	9	25	6.06	33.69	9400	7600	1.4	130
18	2*	9	50	7.36	40.91	6600	5400	1.7	123
19	**2**	15	25	0.65	2.16	7700	5400	1.5	127
20	**2**	15	50	2.02	6.72	6300	4200	1.5	121
21	2*	15	25	4.72	15.72	9900	8100	1.5	127
22	2*	15	50	3.08	10.25	7700	4400	1.4	120
23	3*	3	25	2.43	40.49	16500	-^g^	1.5	119
24	3*	3	50	4.09	68.21	10200	-^g^	1.5	101
25	**3**	9	25	0.98	5.46	16700	-^g^	1.5	95
26	**3**	9	50	6.60	36.71	12300	-^g^	1.3	94
27	3*	9	25	8.37	46.52	20200	-^g^	1.2	97
28	3*	9	50	12.84	71.37	10500	-^g^	1.3	98
29	3*	15	25	11.18	37.28	17700	-^g^	1.5	101

a Experimental conditions: 10 μmol Ni catalyst; reaction time = 2 h; solvent: toluene, 50 mL.

b Cat.* = **Cat.** + 2 equiv of [Ni­(COD)_2_].

c Determined by GPC/SEC (calibration with polystyrene standards), chromatograms in Figures S52–S80.

d Calculated from ^1^H NMR intensity ratios of unsaturated end groups vs. overall integral (spectra in Figures S17–S42 and eq S1,^8e^ SI).

e *Đ* = *M*_n_/*M*_w_.

f Degree of branching (number of branches/1000C atoms), determined by ^1^H NMR, and corrected for methyl end-groups (spectra in Figures S17–S42 and eq S2,^17^ SI).

g Not calculated due to low intensity/resolution in the integration.

**Figure 4:**
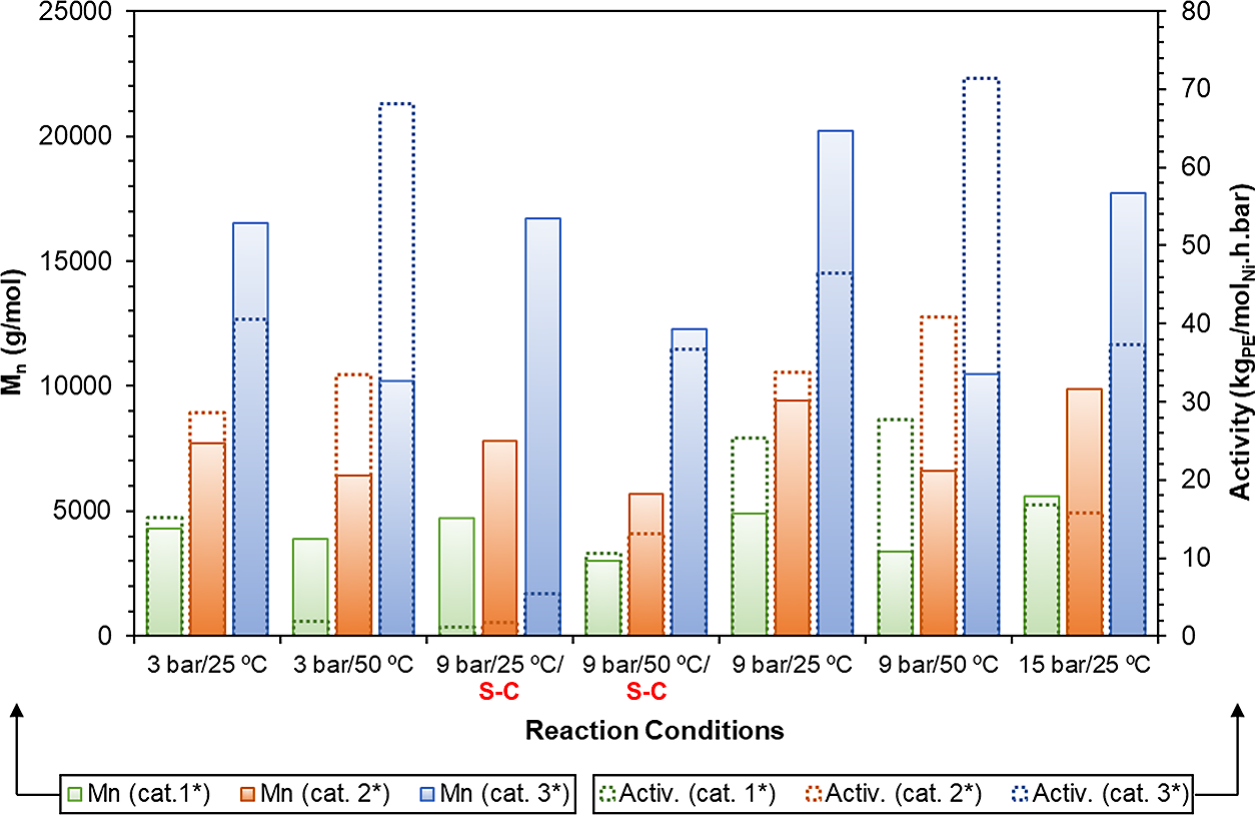
Summary chart comparing catalytic activities (right axis, open bars with dotted outlines) and molecular weights of the resulting PEs (left axis, filled bars) from selected catalytic reactions conducted under various conditions, using tridentate catalyst systems 1*−3*. The exceptions are the two tests labeled as “S–C”, where catalysts **1**−**3** were employed as well-defined *Single-Component* catalysts, i.e., without addition of [Ni­(COD)_2_] as phosphine scavenger. Both activities and PE molecular weights were measured after 2 h of reaction.

Catalysts **1**−**3** displayed activities in the range 1.14–36.71 kg_PE_·mol_Ni_^–1^·h^–1^·bar^–1^ in the absence of activators. In general, upon addition of 2 equiv of the phosphine scavenger [Ni­(COD)_2_], yielding catalyst systems 1*−3*, their performance was significantly enhanced, both in terms of activity and product molecular weight. Under these conditions, the catalytic activities increased to 1.92–71.37 kg_PE_·mol_Ni_^–1^·h^–1^·bar^–1^, with the highest values observed at 9 bar and 50 °C. Control experiments using [Ni­(COD)_2_] alone confirmed its inactivity toward the ethylene oligo-/polymerization under the range of temperatures and pressures examined.

Increasing the reaction temperature enhanced catalytic activity but was accompanied by a reduction in the molecular weights of the resulting PEs, as higher temperatures promote chain transfer processes (e.g., β-H elimination or hydrogen transfer to monomer). In contrast, increasing the reaction pressure from 3 to 9 bar resulted in higher catalytic activities, attributable to the greater solubility of ethylene in toluene. However, a further increase to 15 bar, led, in general, to a decline in activity, possibly due to mass transfer limitations. The molecular weight of the PEs generally increased with reaction pressure, except in the case of catalyst 3*, where a slight decrease was observed between 9 and 15 bar, at 25 °C.

The polymers displayed monomodal and relatively narrow molecular weight distributions (*Đ* = 1.2–1.9), consistent with a well-defined single-site catalytic mechanism. However, despite the narrow distributions, these polymerization processes are governed by chain transfer events that limit the achievable molecular weights. In fact, these catalytic polymerizations lack a living character, as the PE molecular weight remains essentially constant over time.

Increasing the steric bulk of the imine arm leads to a marked enhancement in the catalyst performance. This effect is evident when progressing from *N*-2,6-dimethylphenyl in catalyst **1**, to *N*-2,6-diisopropylphenyl in catalyst **2**, and finally to the more sterically demanding 2,6-terphenyl imine (*N*-2,6-[3,5-(CF_3_)_2_C_6_H_3_]_2_C_6_H_3_) in catalyst **3**. For catalyst **3**, both catalytic activity and PE molecular weight approximately doubled or even tripled. Under identical reaction conditions, catalysts **1**−**2** and 1*−2* exhibited activities and, in some cases, PE molecular weights comparable to those reported for related bidentate 2-iminopyrrolyl **Ni**_**B**_ catalysts (see Scheme 1).^12^ In the latter catalyst systems, these properties depended not only on the structural features of the imine arm but also on the steric and electronic nature of the 5-aryl substituents in the iminopyrrolyl bidentate framework. For the **Ni**_**B**_ catalysts, it was generally observed that the most active systems, such as those incorporating the strongly electron-withdrawing pyrrolyl substituent 5-[3,5-(CF_3_)_2_C_6_H_3_] (see Scheme 1, with R = *i*Pr, R^1^=R^3^=X=H, R^2^=CF_3_, hereinafter referred to as **Ni**_**B**_-6*), produced low molecular weight PEs, whereas bulkier and electron-donating 5-substituents afforded higher molecular weight products at the expense of catalytic activity.^12a^ In contrast, catalysts **3** and 3*, incorporating the much bulkier 2,6-terphenyl imine arm, outperform both the tridentate **Ni**_**T**_ systems and all previously reported **Ni**_**B**_ catalysts,^12^ delivering the highest activities and PE molecular weights *simultaneously*. This performance can be attributed to steric protection of the Ni center, which significantly reduces chain transfer reactions, and possibly also possibly to the influence of the electron-withdrawing CF_3_ groups. The latter may modulate the electron density at the metal either through bonds or via second coordination sphere effects arising from through-space inductive interactions.^18^

After isolation, highly viscous PE oils to pastes were obtained (for dynamic viscosity data of selected samples see Table S7, SI), with molecular weights ranging from 3000 to 20,200 g mol^–1^, which are in general an order of magnitude higher than those obtained using the bidentate catalyst systems.^12^ The very high branching values observed (Table[Table tbl1]), ranging from 94 to 133 branches/1000 C atoms, are comparable to or even exceed those produced with the bidentate catalyst systems. The degree of branching (*N*) is significantly influenced by the bulkiness of the imine arm, decreasing from 120 to 130 with precatalysts **1** and **2** to values under 100 branches/1000 C atoms with the bulkier precatalyst **3**.

A microstructural analysis of these PE products was conducted on selected samples using ^13^C­{^1^H} NMR spectroscopy (Table 2). In all the samples, over 50% of the branches correspond to methyl groups (isolated or paired), followed by a quite significant number (15–22%) of long branches (greater than C4). Most notably, *sec*-butyl groups, characteristic of a branch-on-branch structure, are also present, indicating a hyperbranched microstructure in the PEs. The PEs obtained with precatalysts **1** and **2** also exhibit a higher degree of hyperbranching compared to those obtained with precatalyst **3**, as the content of *sec*-butyl groups decreases from 7 to 8% to 2–3%, respectively. Compared to the bidentate systems,^12^ there is a general decrease in the percentage of butyl, long and *sec*-butyl branches balanced by an increase in methyl branches to values exceeding *ca*. 50%. An increase in the reaction temperature leads to a decrease in methyl branches and an increase in butyl, *sec*-butyl and longer branches. As expected,^19^ there is a significant decrease in the *sec*-butyl branches with an increase in the reaction pressure from 3 to 15 bar.

**Table 2:** Microstructural Analysis of the PEs Obtained with Catalyst Systems 1*−3* (**1**−**3** + [Ni­(COD)_2_] (2 equiv)), at 25 and 50 °C and at Different Ethylene Pressures

					type of branches (%)^b^					
entry	Cat.	*P*_abs_ (bar)	*T* (°C)	*N* ^a^	methyl	ethyl	propyl	butyl	*sec*-butyl	long
5	1*	9	25	120	54	7	3	10	7	18
6	1*	9	50	115	49	9	3	10	8	21
13	2*	3	25	133	56	9	2	9	9	15
14	2*	3	50	121	52	9	2	10	9	18
17	2*	9	25	130	61	9	4	4	7	15
18	2*	9	50	123	53	10	3	9	8	17
21	2*	15	25	127	64	7	3	8	5	13
27	3*	9	25	97	72	5	2	1	2	18
28	3*	9	50	98	57	8	2	8	3	22

a Branches/1000 C atoms, determined by ^1^H NMR (see Table 1).

b Determined by ^13^C­{^1^H} NMR (spectra in Figures S43–S51 and eq S11,^20^ SI).

To assess the robustness of catalyst systems 1*−3*, kinetic profiles of ethylene consumption were monitored over time during catalytic reactions performed at *P*_abs_ = 9 bar and *T* = 25 °C (Figure 5). In all cases, an increase in the reaction rate was observed during the first 30 to 50 min, reaching a maximum ethylene consumption, followed by a gradual decline in catalytic activity (occurring between 60 and 150 min for catalysts 1* and 2*, and between 30 and 210 min for catalyst 3*), and finally a phase of rapid deactivation (after 150 to 200 min). For comparison, the kinetic profile of the most active **Ni**_**B**_ catalyst previously reported (**Ni**_**B**_ -6*),^12a^ is also shown in Figure 5, exhibiting an increase in the reaction rate up to ca. 100 min, a subsequent decline between 100 and 235 min and rapid deactivation thereafter.

**Figure 5:**
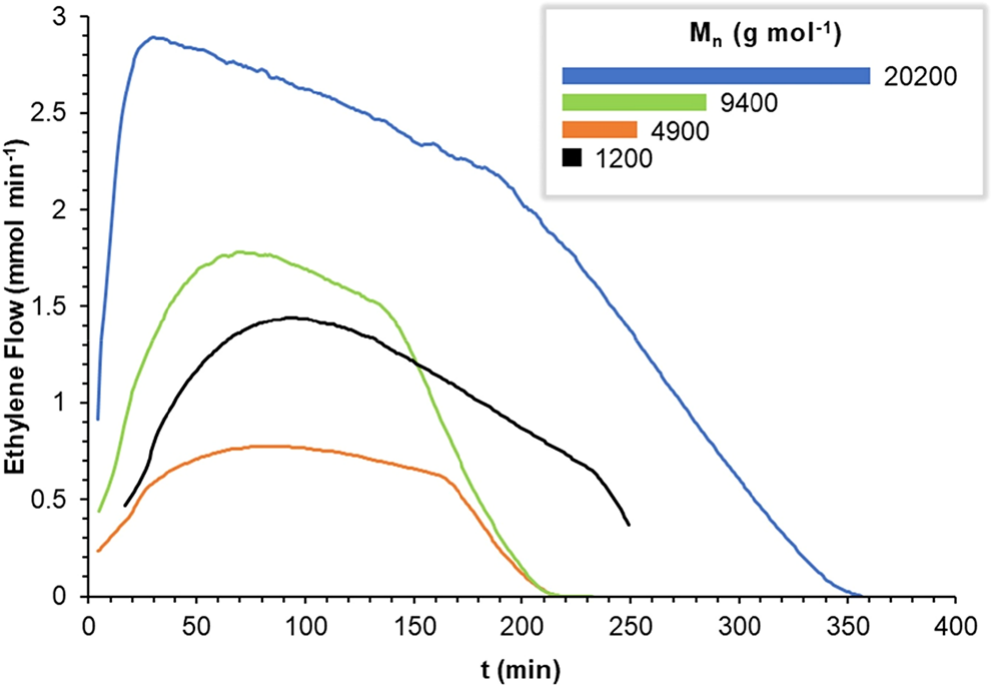
Catalytic profiles for ethylene polymerization using tridentate systems 1* (orange), 2* (green), 3* (blue), compared with the bidentate **Ni**_**B**_-6* (black) system,^12a^ where X* = **X** + [Ni­(COD)_2_] (2 equiv), at 25 °C, under a constant ethylene pressure of 9 bar (absolute). The molecular weights of the resulting PEs were determined at the end of each reaction.

Catalyst systems 1* and 2* remained active for almost 4 h of reaction under the tested conditions. However, 2* is generally 3 times more active than 1*, due to the higher steric hindrance of its imine arm. The replacement of methyl with isopropyl substituents in 2* enhances the steric protection of the Ni catalytic centers, influencing not only the rate of monomer consumption but also the resulting PE product, whose molecular weight is nearly twice that produced by 1* (4900 vs 9400 g mol^–1^). In the case of catalyst system 3*, the differences relative to the other two systems are striking: it is 2 to 5 times more active and remains active for nearly twice as long. Moreover, the PEs obtained with 3* exhibit much higher molecular weights, reaching up to 20,200 g mol^–1^, which is 2 to 4 times greater than those from 1* and 2*. The bidentate catalyst **Ni**_**B**_ -6* displays an activity comparable to that of 2*, although its lifetime appears to be longer. Its activity profile, combined with the substantially lower molecular weight of the resulting PE (1200 g mol^–1^), likely arises from the presence of electron-withdrawing CF_3_ groups in the pyrrolyl substituent 5-[3,5-(CF_3_)_2_C_6_H_3_]. These groups render the Ni center more electrophilic, promoting stronger activation of coordinated ethylene, faster insertion, and a greater propensity for β-hydride elimination compared to **Ni**_**B**_ catalysts lacking such electron-withdrawing substituents.

The overall results for catalysts 1*−3* are consistent with the well-established mechanistic understanding that both the activity of polymerization catalysts and the molecular weight of the resulting PEs are strongly influenced by the steric volume of the chelating ligand in nickel complexes.^8a–8c,19,21^ In the specific case of the *C*,*N*,*N’*-pincer iminopyrrolyl nickel precatalysts studied here, the metal–carbon bond that initiates ethylene insertion is embedded within the chelating ligand itself (see Figure S81 in the SI for possible polymerization pathways). Following the initial intramolecular insertions of ethylene into the *C,N,N’*-metalacycle, β-hydride elimination can occur. If this elimination happens immediately after the first insertion, it yields a bidentate nickel species bearing a 2-propenyl-6-xylyl group at the 5-position of the pyrrolyl ring (species **A** in Figure S81), whose steric profile is comparable to that of previously reported bidentate nickel (**Ni**_**B**_) precatalysts.^12^ However, if β-hydride elimination occurs only after several ethylene insertions, the resulting bidentate species may retain an oligo- or polyethylene chain covalently attached to the 5-phenyl ring of the iminopyrrolyl ligand framework (see schematic representation below in Scheme 3 and species **B** in Figure S81). This substantial increase in steric hindrance could provide additional protection to the metal center, potentially accounting for the enhanced catalytic activity and higher PE molecular weights observed, relative to the **Ni**_**B**_-based precatalysts.^12^ Preliminary attempts to validate this hypothesis by probing the initial ethylene insertions into the *C,N,N’*-tridentate nickel precatalyst **2** (7:1 ethylene-to**-**precatalyst molar ratio in C_6_D_6_) using ^1^H and DOSY NMR spectroscopy were unsuccessful (see details and Figures S82–S84 in the SI). This outcome may reflect the intrinsic nature of the catalytic system, which does not exhibit typical features of a living polymerization process, suggesting that only a very small fraction of the precatalyst is converted into catalytically active species, remaining below the detection limit of ^1^H NMR. Nevertheless, this possibility cannot be definitively excluded. Further studies could be pursued, for example by quench-labeling or -hydrolyzing the ethylene polymerization mixture, and analyzing the products by LC-MS to detect ligand-oligomer adducts, as previously reported for olefin polymerization catalyzed by dimethyl hafnium complexes bearing pyridine- or quinoline-amido *C,N,N*-tridentate ligands.^22^ However, such studies are beyond the scope of the present work.

**Scheme 3:**
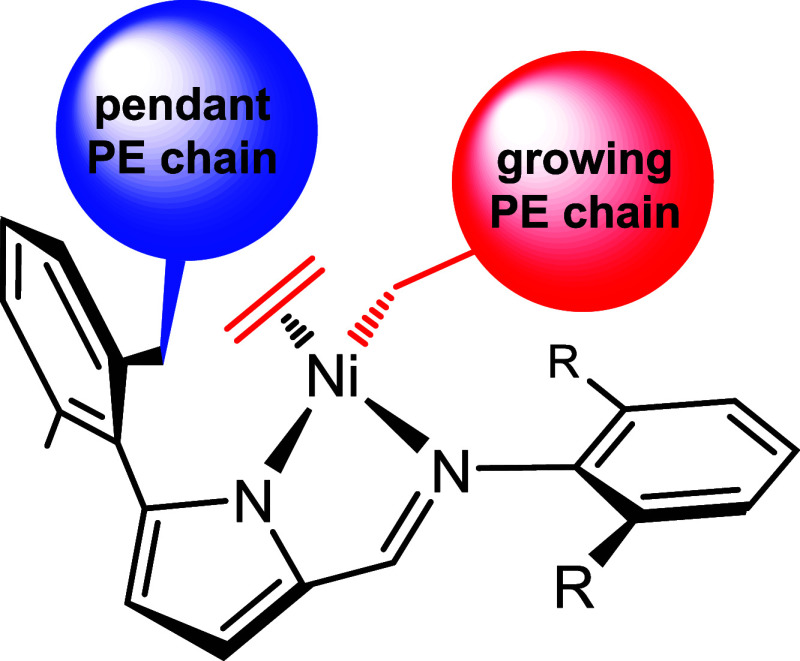
Schematic Representation of the Hypothetic Bidentate Species Arising from β-Hydride Elimination within the Expanded *C,N,N’*-Metalacycle (after Several Ethylene Insertions), Which May Retain an Oligo- or Polyethylene Pendant Chain Attached to the 5-Phenyl Ring of the Iminopyrrolyl Ligand Framework

## Conclusions

This study reports the synthesis and characterization of a novel family of thermally stable tridentate *C*,*N*,*N’*-pincer iminopyrrolyl nickel­(II) complexes **1**−**3**, formed via cyclometalation through an unexpected intramolecular C–H activation, and their application as catalysts for the polymerization of ethylene to hyperbranched polyethylenes (HBPEs). The optimal reaction temperatures for synthesizing these tridentate complexes were determined to be 45 °C for complex **1**, 30 °C for complex **2**, and 65 °C for complex **3**. These conditions yielded pure solids with high efficiency, confirmed through detailed NMR spectroscopy and single-crystal X-ray diffraction.

NMR spectroscopy studies revealed that, at reaction temperatures of 20–30 °C, the formation of tridentate *C*,*N*,*N’*-pincer nickel­(II) complexes (**Ni**_**T**_) occurs simultaneously with the formation of bidentate *N,N’*-2-iminopyrrolyl nickel complexes (**Ni**_**B**_). At higher reaction temperatures, only the tridentate complexes are identifiable. Notably, when the reaction was conducted at ambient temperature, a single crystal of the minor bidentate complex **1**_**B**_ was obtained and structurally characterized by X-ray diffraction, although it could not be isolated in pure form due to its intrinsic kinetic thermal instability. The cyclometalation mechanism was analyzed by DFT calculations, indicating that the C–H activation proceeds via a Ni-mediated σ-bond metathesis mechanism.

Catalytic studies showed that complexes **1**−**3** exhibited notable activity in ethylene polymerization, producing HBPEs with molecular weights up to an order of magnitude greater than those obtained with the most closely related bidentate *N,N′*-iminpyrrolyl precatalysts.^12^ The addition of the phosphine scavenger [Ni­(COD)_2_] led to a marked enhancement in catalytic performance. The resulting polyethylenes featured relatively narrow molecular weight distributions and hyperbranched architectures, consistent with well-defined single-site catalysis involving an extensive chain-walking mechanism, although the system does not exhibit the characteristics of a living polymerization process.

Increasing the bulkiness of the imine arm significantly improved catalyst performance, with complex **3** showing the highest activity and molecular weight of the polyethylene produced. The results underscore the influence of steric bulk on catalyst performance, enhancing protection of the nickel center and reducing chain transfer reactions.

Overall, this research highlights the potential of tridentate *C*,*N*,*N’*-pincer iminopyrrolyl nickel­(II) complexes as an effective and distinct class of well-defined single-component catalysts for the polymerization of ethylene to HBPEs. These findings offer valuable insights for developing more robust single-component alkyl-nickel precatalysts supported by pincer ligands, emphasizing the importance of reactive pincer arms and steric tuning in designing efficient catalysts.

The polyethylene oils and pastes obtained in this work, featuring hyperbranched architectures and relatively low molecular weights, hold potential as synthetic base oils for high -performance lubricants or as pour-point depressants.^23^ Furthermore, the presence of vinylene unsaturations in the polymer chains enables their postfunctionalization or application as macromonomers.

## Experimental Section

### General Procedures

The experiments dealing with air- and/or moisture-sensitive materials were carried out under nitrogen atmosphere using a glovebox, a dual vacuum/nitrogen line and standard Schlenk techniques.^24^ The nitrogen gas was supplied in cylinders from Air Liquide and purified passing through 4 Å molecular sieves. The solvents were predried with 4 Å molecular sieves, purified by refluxing over a drying agent (sodium/benzophenone for diethyl ether, THF and toluene; calcium hydride for *n*-hexane), and further distilled and stored under nitrogen in J. Young ampules.

The 2,6-dimethylaniline reagent was dried over calcium hydride, distilled trap-to-trap and stored under nitrogen. Sodium hydride was purchased in 60% mineral oil dispersion, washed several times with *n*-hexane, dried under vacuum and stored under nitrogen. The 5-(2,6-dimethylphenyl)-2-formyl-1*H*-pyrrole,^11^ the 2,6-bis­[3,5-bis­(trifluoromethyl)­phenyl] aniline,^26^ the ligand precursor **II** (5-(2,6-dimethylphenyl)-2-[*N*-(2,6-diisopropylphenyl)­formimino]-1*H*-pyrrole),^9a^ the starting materials *trans*-[Ni­(Ph)­(PPh_3_)_2_Cl][Bibr cit12a] and *trans*-[Ni­(*o*-C_6_H_4_Cl)­(PPh_3_)_2_Cl],^12b^ and the phosphine abstractor [Ni­(COD)_2_],^27^ were prepared as described in the literature.

NMR spectra were recorded on Bruker Avance III 300 and Bruker Avance III 400 spectrometers. Deuterated solvents were dried over molecular sieves (4 Å for toluene-*d*_*8*_, CDCl_3_ and CD_2_Cl_2_ or 3 Å for CD_3_CN), degassed by the freeze–pump–thaw method and stored under inert atmosphere in J. Young ampules. Spectra were referenced internally using the residual resonance for ^1^H and ^13^C from the solvent relative to tetramethylsilane (δ = 0). ^23^Na and ^31^P were referenced using NaCl 1 M (δ = 0) and H_3_PO_4_ 85% (δ = 0), respectively. Sodium spectra of the samples were obtained after digital subtraction of the ^23^Na resonance characteristic of the 5 mm tube glass material, which was measured in a blank experiment performed with an empty tube. The chemical shifts are quoted in δ (ppm) and the coupling constants *J* in Hz. Multiplicities were abbreviated as follows: broad (br), singlet (s), doublet (d), doublet of doublets (dd), triplet (t), heptet (h) and multiplet (m). To perform nuclei resonance assignments, 2D NMR experiments were also performed (^1^H–^1^H COSY, ^1^H–^1^H NOESY, ^1^H–^13^C HSQC and/or ^1^H–^13^C HMBC). For the air- and/or moisture-sensitive materials, samples were prepared inside the glovebox in J. Young tubes.

The elemental analyses were performed in a Fisons Instrument Mod EA-1108, at *Laboratório de Análises* (IST). For each compound two independent determinations were executed.

### Syntheses of Ligand Precursors and their Corresponding Sodium Salts

As noted above, ligand precursors **I** and **III** are new compounds, whereas ligand precursor **II** was previously reported.^9a^ In contrast, the sodium salts of **I** and **II** (**I**_**Na**_ and **II**_**Na**_, respectively), obtained by deprotonation with NaH, were isolated and characterized here for the first time. The synthesis and characterization of ligand precursors **I**, **III**, **I**_**Na**_ and **II**_**Na**_ are provided in Supporting Information (SI).

### Synthesis of Complex [Ni­{κ^3^ *C,N,N*’-5-(2′-CH_2_–6-MeC_6_H_3_)-NC_4_H_2_-2-C­(H)=N-2,6-Me_2_C_6_H_3_}­(PPh_3_)] (**1**)

Complex *trans*-[Ni­(*o*-C_6_H_4_Cl)­(PPh_3_)_2_Cl] (1.24 g, 1.70 mmol) was suspended in toluene (30 mL) at −40 °C, and a toluene solution (10 mL) of sodium salt **I**_**Na**_ (0.844 g, 1.84 mmol) was added dropwise. The cooling bath was then replaced by an oil bath, and the mixture gradually heated to 45 °C and stirred at this temperature overnight. The solvent of the resulting red mixture was removed under vacuum, and the residue dissolved in diethyl ether at −10 °C. The resulting dark orange solution afforded red crystals of **1** suitable for single-crystal X-ray diffraction. X-ray analysis showed that the pure product formed a 1:1 cosolvate of **1** with free PPh_3_. The crystals were washed with cold *n*-hexane to remove the free PPh_3_, yielding an orange powder that, after drying for several hours, was identified by NMR as a 1:0.6 cosolvate (0.810 g, 62%).

In a second synthesis, the same quantities of reagents and solvent, as well as identical reaction time and temperature, were employed. At the end of the reaction, the solution was allowed to cool to room temperature and then filtered. The solvent was removed under reduced pressure, and the resulting residue was washed with cold *n*-hexane, extracted with diethyl ether, and reprecipitated by addition of *n*-hexane. The resulting red solid was collected by filtration and dried under vacuum to afford analytically pure complex **1** (0.687 g, 65%).

**Anal. Calcd.** for C_39_H_35_N_2_NiP: C 75.38, H 5.68, N 4.51; **Found**: C 75.51, H 5.70, N 4.25.

^**1**^ **H NMR** (300 MHz, CD_2_Cl_2_): δ 7.55–7.49 (m, 6H, Ni-PPh_3_-H_*ortho*_), 7.35–7.30 (m, 4H, Ni-PPh_3_-H_*para*_ and N = C*H*), 7.21–7.17 (m, 6H, Ni-PPh_3_-H_*meta*_), 6.98 (d, ^3^ *J*_HH_ = 7.4 Hz, 1H, 5-Ph-H_*meta*_^CH_3_^), 6.86–6.83 (m, 2H, H3 and H4), 6.66–6.55 (m, 2H, N-Ph-H_*para*_ and 5-Ph-H_*para*_), 6.50 (d, ^3^ *J*_HH_ = 7.5 Hz, 2H, N-Ph-H_*meta*_), 6.09 (d, ^3^ *J*_HH_ = 7.5 Hz, 1H, 5-Ph-H_*meta*_^CH_2_^), 2.62 (s, 3H, 5-Ph–C*H*_3_), 2.34 (d, ^3^ *J*_HP_ = 11.1 Hz, 2H, C*H*_2_), 2.08 (s, 6H, N-Ph–C*H*_3_). ^**13**^ **C­{** ^**1**^ **H} NMR** (75 MHz, CD_2_Cl_2_): δ 161.6 (d, ^3^ *J*_CP_ = 2.6 Hz, N = *C*H), 149.7 (N-Ph–C_*ipso*_), 145.0 (d, ^3^ *J*_CP_ = 1.8 Hz, 5-Ph–C_*ortho*2_^CH^), 144.4 (d, ^3^ *J*_CP_ = 1.5 Hz, C5), 141.6 (C2), 135.5 (5-Ph–C_*ortho*_^CH_3_^), 134.5 (d, ^2^ *J*_CP_ = 11.3 Hz, PPh_3_-C_*ortho*_), 134.1 (5-Ph–C_*ipso*_), 131.7 (d, ^1^ *J*_CP_ = 42.0 Hz, PPh_3_-C_*ipso*_), 131.0 (N-Ph–C_*ortho*_), 130.1 (d, ^4^ *J*_CP_ = 2.1 Hz, PPh_3_-C_*para*_), 128.5 (d, ^3^ *J*_CP_ = 9.7 Hz, PPh_3_-C_*meta*_), 128.3 (N-Ph–C_*meta*_), 127.6 (5-Ph–C_*meta*_^CH_3_^), 126.8 (5-Ph–C_*meta*_^CH_2_^), 125.0 (N-Ph–C_*para*_), 124.8 (5-Ph–C_*para*_), 117.3 (d, ^4^ *J*_CP_ = 1.7 Hz, C3), 113.9 (d, ^4^ *J*_CP_ = 3.3 Hz, C4), 24.5 (d, ^2^ *J*_CP_ = 21.5 Hz, *C*H_2_), 24.1 (5-Ph-*C*H_3_), 19.2 (N-Ph-*C*H_3_). ^**31**^ **P­{** ^**1**^ **H} NMR** (121 MHz, CD_2_Cl_2_): δ 35.9 (Ni-*P*Ph_3_).

### Synthesis of Complex [Ni­{κ^3^ *C,N,N’*-5-(2′-CH_2_–6-MeC_6_H_3_)-NC_4_H_2_-2-C­(H)=N-2,6-*i*Pr_2_C_6_H_3_}­(PPh_3_)] (**2**)

Complex *trans*-[Ni­(*o*-C_6_H_4_Cl)­(PPh_3_)_2_Cl] (1.24 g, 1.70 mmol) was suspended in toluene (30 mL) at −40 °C, and a toluene solution (10 mL) of sodium salt **II**_**Na**_ (0.765 g, 1.72 mmol) was added dropwise. The cooling bath was then replaced by an oil bath, and the mixture gradually warmed to 30 °C and stirred at this temperature overnight. At the end of the reaction, the solution was allowed to cool to room temperature and then filtered. The solvent was removed under reduced pressure, and the resulting red residue was washed with cold *n*-hexane and dissolved in diethyl ether at −10 °C. The resulting dark orange solution was concentrated and stored at −20 °C, affording complex **2** as a red powder (0.576 g, 50%). A small portion was recrystallized from diethyl ether at −20 °C to yield red crystals suitable for single-crystal X-ray diffraction.

**Anal. Calcd.** for C_43_H_43_N_2_NiP: C 76.23, H 6.40, N 4.13; **Found**: C 76.04, H 6.40, N 4.12.

^**1**^ **H NMR** (300 MHz, CD_2_Cl_2_): δ 7.42–7.36 (m, 7H, N = C*H* and PPh_3_-H_*ortho*_), 7.33–7.28 (m, 3H, PPh_3_-H_*para*_), 7.19–7.14 (m, 6H, PPh_3_-H_*meta*_), 6.96–6.92 (m, 2H, N-Ph-H_*para*_ and 5-Ph-H_*meta*_^CH_3_^), 6.86 (s, 1H, H3), 6.81 (s, 1H, H4), 6.74 (d, ^3^ *J*_HH_ = 7.7 Hz, 2H, N-Ph-H_*meta*_), 6.48 (t, ^3^ *J*_HH_ = 7.5 Hz, 1H, 5-Ph-H_*para*_), 5.80 (d, ^3^ *J*_HH_ = 7.5 Hz, 1H, 5-Ph-H_*meta*_^CH_2_^), 3.58–3.50 (m, 2H, C*H*(CH_3_)_2_), 2.60 (s, 3H, C*H*_3_), 2.34 (d, ^3^ *J*_HP_ = 11.4 Hz, 2H, C*H*_2_), 1.05 (d, ^3^ *J*_HH_ = 6.8 Hz, 6H, CH­(C*H*_3_)­(CH_3_)), 0.82 (d, ^3^ *J*_HH_ = 6.9 Hz, 6H, CH­(CH_3_)­(C*H*_3_)). ^**13**^ **C­{** ^**1**^ **H} NMR** (75 MHz, CD_2_Cl_2_): δ 161.8 (d, ^3^ *J*_CP_ = 2.6 Hz, N = *C*H), 148.8 (N-Ph–C_*ipso*_), 144.7 (d, ^3^ *J*_CP_ = 1.8 Hz, 5-Ph–C_*ortho*_^CH_2_^), 144.7 (d, ^3^ *J*_CP_ = 1.6 Hz, C5), 141.8 (N-Ph–C_*ortho*_), 141.1 (d, ^3^ *J*_CP_ = 0.5 Hz, C2), 135.1 (5-Ph–C_*ortho*_^CH_3_^), 134.5 (d, ^2^ *J*_CP_ = 11.4 Hz, PPh_3_-C_*ortho*_), 134.0 (5-Ph–C_*ipso*_), 131.9 (d, ^1^ *J*_CP_ = 41.9 Hz, PPh_3_-C_*ipso*_), 129.9 (d, ^4^ *J*_CP_ = 2.1 Hz, PPh_3_-C_*para*_), 128.6 (d, ^3^ *J*_CP_ = 9.6 Hz, PPh_3_-C_*meta*_), 127.5 (N-Ph–C_*para*_), 126.4 (5-Ph–C_*meta*_^CH_2_^), 126.0 (5-Ph–C_*meta*_^CH_3_^), 124.9 (5-Ph–C_*para*_), 123.3 (N-Ph–C_*meta*_), 117.2 (d, ^4^ *J*_CP_ = 1.6 Hz, C3), 113.7 (d, ^4^ *J*_CP_ = 3.3 Hz, C4), 28.5 (*C*H­(CH_3_)_2_), 25.8 (CH­(*C*H_3_)­(CH_3_)), 24.1 (d, ^2^ *J*_CP_ = 21.8 Hz, *C*H_2_), 23.7 (*C*H_3_), 22.0 (CH­(CH_3_)­(*C*H_3_)). ^**31**^ **P­{** ^**1**^ **H} NMR** (121 MHz, CD_2_Cl_2_): δ 33.4.

### Synthesis of Complex [Ni­{κ^3^ *C,N,N*’-5-(2′-CH_2_–6-MeC_6_H_3_)-NC_4_H_2_-2-C­(H)=N-2,6-[3,5-(CF_3_)_2_C_6_H_3_]_2_C_6_H_3_}­(PPh_3_)] (**3**)

Ligand precursor **III** (1.05 g, 1.5 mmol) was suspended in THF (10 mL) and added dropwise to a Schlenk tube containing NaH powder (0.05 g, 2.0 mmol) under stirring. After the addition, the mixture was refluxed for 2 h, cooled to room temperature, and filtered to remove excess NaH. The solvent was then removed under vacuum, affording sodium salt **III**_**Na**_ as a powder, which was dried and used without further purification. Complex *trans*-[Ni­(Ph)­(PPh_3_)_2_Cl] (0.83 g, 1.2 mmol) was suspended in toluene (30 mL) at −20 °C, and a toluene solution (10 mL) of sodium salt **III**_**Na**_ (0.844 g, 1.84 mmol) was added dropwise. The cooling bath was then replaced by an oil bath, and the mixture gradually heated to 65 °C and stirred at this temperature overnight. After cooling to room temperature, the resulting red mixture was filtered. The solvent was removed under reduced pressure, and the residue was washed with *n*-pentane at −20 °C. The resulting orange solid of complex **3** was dried under vacuum (0.74 g, 61%). The filtered *n*-pentane solution was subsequently concentrated and stored at −20 °C, yielding orange crystals suitable for single-crystal X-ray diffraction.

**Anal. Calcd.** for C_53_H_35_F_12_N_2_NiP: C 62.56, H 3.47, N 2.75; **Found**: C 62.78, H 3.20, N 2.67.

^**1**^ **H NMR** (300 MHz, CD_2_Cl_2_): δ 8.06 (s, 4H, H18), 7.89 (s, 2H, H21), 7.32–7.04 (m, 19H, C*H*N, N-Ph-H_*meta*_ and H_*para*_, and PPh_3_), 6.99 (d, 1H, ^3^ *J*_HH_ = 7.3 Hz, H12), 6.87–6.83 (m, 2H, H3 and H4), 6.54 (t, 1H, ^3^ *J*_HH_ = 7.4 Hz, 5-Ph-H_*para*_), 6.01 (d, 1H, ^3^ *J*_HH_ = 7.4 Hz, H10), 2.61 (s, 3H, C*H*_3_), 2.13 (d, 2H, ^3^ *J*_HP_ = 11.1 Hz, C*H*_2_). ^**13**^ **C­{** ^**1**^ **H} NMR** (75 MHz, CD_2_Cl_2_): δ 162.4 (d, ^3^ *J*_CP_ = 2.6 Hz, *C*HN), 148.9 (N-Ph–C_*ipso*_), 147.8 (d, ^3^ *J*_CP_ = 1.1 Hz, C5), 145.0 (d, ^3^ *J*_CP_ = 1.6 Hz, C8), 141.6 (C2), 141.3 (C16), 135.9 (C13), 134.3 (d, ^2^ *J*_CP_ = 11.4 Hz, PPh_3_-C_*ortho*_), 133.6 (5-Ph–C_*ipso*_), 133.5 (C17), 131.9 (PPh_3_-C_*para*_), 131.7 (quart, ^2^ *J*_CF_ = 33.0 Hz, C19), 131.3 (d, ^3^ *J*_CF_ = 2.7 Hz, C18), 130.2 (br, N-Ph–C_*meta*_ and C_*para*_), 128.6 (d, ^3^ *J*_CP_ = 10.4 Hz, PPh_3_-C_*meta*_), 127.7 (C12), 126.7 (d, ^4^ *J*_CP_ = 7.7 Hz, C10), 125.2 (5-Ph–C_*para*_), 123.9 (quart, ^1^ *J*_CF_ = 271.3 Hz, *C*F_3_), 121.0 (sept, ^3^ *J*_CF_ = 3.6 Hz, C21), 120.2 (d, ^4^ *J*_CP_ = 1.7 Hz, C3), 115.3 (d, ^4^ *J*_CP_ = 3.3 Hz, C4), 24.8 (d, ^2^ *J*_CP_ = 20.9 Hz, Ni-*C*H_2_), 23.7 (*C*H_3_). ^**31**^ **P­{** ^**1**^ **H} NMR** (CD_2_Cl_2_, 121 MHz): δ 33.1. ^**19**^ **F­{** ^**1**^ **H} NMR** (CD_2_Cl_2_, 282 MHz): δ −63.0.

### X-ray Data Collection

Crystals were selected from the growing environment, under nitrogen if sensitive to air and/or moisture and covered with polyfluoroether oil. Crystallographic data were collected using graphite monochromated Mo–Kα radiation (λ = 0.71073Å) on a Bruker AXS-KAPPA APEX II diffractometer equipped with an Oxford Cryosystem open-flow nitrogen cryostat, at 150 K. Cell parameters were retrieved using Bruker SMART software and refined using Bruker SAINT on all observed reflections. Absorption corrections were applied using SADABS.^28^ Structure solution and refinement were performed using direct methods with program SIR2004,^29^ SIR2014,^30^ and SHELXL,^31^ all included in the software package WinGX v2018.3.^32^ Non-hydrogen atoms were refined anisotropically and all the hydrogen atoms, with exception of the N*H* protons, were inserted in idealized positions and allowed to refine riding on the parent carbon atom. Figures of the molecular structures were generated using Mercury 2023.3.1, software available at the *Cambridge Crystallographic Data Centre (CCDC)*. Data have been deposited with the CCDC under deposition numbers 2373040 for **I**, 2373041 for **II**, 2373042 for **III**, 2373046 for **1**_**B**_, 2373043 for **1**, 2373044 for **2**, and 2373045 for **3**. The supplementary crystallographic data can be obtained free of charge from The Cambridge Crystallographic Data Centre via www.ccdc.cam.ac.uk/data_request/cif.

For the iminopyrrole ligand precursors **I**–**III**, the crystallographic and experimental details of the crystal structure determinations are listed in Table S1and the selected bond distances and angles in Table S3. For complexes **1**–**3** and **1**_**B**_, the crystallographic and experimental details of the crystal structure determinations are listed in Table S2, the selected bond distances and angles in Table S4 and the dihedral angles in Table S5.

Ligand precursor **II** had been previously reported, and the described synthetic procedure was followed.^9a^ However, its recrystallization in *n*-hexane at −20 °C afforded red crystals that were characterized by X-ray diffraction. The crystals were of poor quality and showed a weak diffracting power for resolutions better than 0.82. It has a high *R*_int_ (0.1455) and a relatively low ratio of observed/unique reflections (43%), nevertheless the structure refined to convergence.

### Calculation of the Percentage of Buried Volume (%V_Bur_) of *C,N,N’*-Pincer Iminopyrrolyl Ligands in Complexes **1–3**

The percentage of buried volume (% V_Bur_)^15^ contributed by the ligands within spheres of 3.5, 5, 7, and 9 Å radius centered on the nickel atoms of complexes **1**-**3** was determined using either the SambVca 2.1 ^33^ web application or its implementation integrated in ChimeraX software^34^ (version 1.10.1) through the SEQCROW add-on^35^ (Table S6 and Figure S11). Visualization of the buried volumes and generation of the corresponding steric maps^36^ for complexes **1**-**3** (Figure S12) were performed using ChimeraX.

### Typical Experimental Procedure for Reactions at NMR Tube Scale

Inside a glovebox, a solution of the nickel precursor *trans*-[Ni­(*o*-C_6_H_4_Cl)­(PPh_3_)_2_Cl] (**SM**, 0.013 mmol) in toluene-*d*_8_ (0.5 mL) was prepared in a J. Young NMR tube. Separately, a solution of the corresponding ligand sodium salt (0.013 mmol) in toluene-*d*_8_ (0.5 mL) was prepared in a small Schlenk tube. In the experiment designed to evaluate the influence of free phosphine, PPh_3_ (0.026 mmol) was also added to the ligand salt solution. The NMR tube containing the nickel starting material solution was placed inside a larger Schlenk tube connected to a dual vacuum/nitrogen line and cooled to −80 °C. The sodium salt solution was then transferred to the NMR tube via cannula. The reaction mixture was maintained at −80 °C until the start of the NMR measurements. Two types of NMR experiments were performed: (a) Variable-temperature (VT) experiments, from −40 to 50 °C in 10 °C increments, recording both ^1^H and ^31^P­{^1^H} NMR spectra at each temperature after a stabilization period of *ca*. 5 min (Figures 2, S13 and S15); and (b) Constant temperature experiments, in which ^1^H NMR spectra were recorded every 5 min during the first hour, followed by two ^1^H and two ^31^P­{^1^H} per hour for the next 12 h (Figure S14). Typically, each ^1^H spectrum required 5 min, and each ^31^P­{^1^H} spectrum 25 min.

### Computational Details

Quantum chemical calculations were performed using the Gaussian 16 rev C.01 software package^37^ and the PBE0 functional,^38^ including empirical correction for dispersion obtained through Grimme’s DFT-D3 method^39^ with Becke and Jonhson short distance damping.^40^ The basis set used for the geometry optimizations (basis b1) consisted of the Stuttgart/Dresden ECP (SDD) basis set^41^ augmented with an f-polarization function to describe the electrons of Ni,^42^ and a standard 6–31G­(d,p)^43,44^ basis set for all other atoms. Transition state optimizations were performed with the Synchronous Transit-Guided Quasi-Newton Method (STQN) developed by Schlegel et al.,^45,46^ following extensive searches of the Potential Energy Surface. Frequency calculations were performed to confirm the nature of the stationary points, yielding one imaginary frequency for the transition states and none for the minima. Each transition state was further confirmed by following its vibrational mode downhill on both sides and obtaining the minima presented on the energy profile. The electronic energies obtained at the PBE0/b1 level of theory were converted to free energy at 298.15 K and 1 atm by using zero-point energy and thermal energy corrections based on structural and vibration frequency data calculated at the same level. Single point energy calculations were performed on the geometries optimized at the PBE0/b1 level using the same functional, the same basis set for the metal atoms, and a standard 6–311++G­(d,p)^47,48^ basis set for the other elements (basis b2). Implicit solvent effects were considered using the Polarizable Continuum Model (PCM) initially devised by Tomasi and co-workers^49,50^ with radii and nonelectrostatic terms of the SMD solvation model, developed by Truhlar et al. considering toluene as solvent.^51^ The same computational procedure was applied to investigate a potential C–H activation in the triplet-state, for which a more appropriate the level of theory was chosen, including the range-corrected functional developed by Head-Gordon and co-workers: ωB97X-D,SMD/def2-TZVP//ωB97X-D,SMD/def2-SVP.^52,53^

### Polymerization Tests Procedure

The polymerizations were carried out in 300 mL Miniclave Drive Büchi pressure reactors, equipped with magnetically stirred glass reaction vessels with external protective meshes, which were previously dried at 140 °C in the oven. The reaction vessels were degassed during the cooling with 3 cycles of vacuum/nitrogen, and 50 mL of freshly distilled toluene were added, by cannula to the glass reaction vessels, under a pressure of 1.3 bar of nitrogen. The reaction temperature was defined, and the autoclave immersed in a thermostatic bath at the selected temperature. After a slight degassing, the reactors were filled with an ethylene atmosphere at approximately 5 bar (relative pressure).

After preconditioning the reactor and solvent at the selected temperature, for the polymerizations carried out with cocatalyst, 2 mL of a 10^–2^ M solution of [Ni­(COD)_2_] in toluene were added, immediately followed by 1 mL of a 10^–2^ M solution of the catalyst in toluene, both *via* glass syringes. For the polymerizations without cocatalyst, only 1 mL of a 10^–2^ M solution of the catalyst in toluene was added. The pressure was raised up to the desired pressure (3, 9, or 15 bar – absolute pressure) and maintained during the reaction time (2 h). At the end of the test, the ethylene supply valve was closed, the reactor was removed from the thermostatic bath and the pressure was slowly released. The reactor was opened, and the reaction medium was then quenched with methanol while stirring. The polyethylene products were isolated by removal of the volatiles in the rotary evaporator and drying in a vacuum line, and later in a vacuum oven at room temperature, until constant weight, yielding colorless dense oils.

Blank tests were performed in the studied reaction conditions (3, 9, and 15 bar; and 25 and 50 °C), using 2 eq. of [Ni­(COD)_2_] or a mixture of [Ni­(COD)_2_]:PPh_3_ (2:1). In all cases no ethylene oligomers or polymers were formed.

Once the molecular structure of complex **1** was determined to be a 1:1 cosolvate with free PPh_3_ and extensive vacuum drying resulted in a final ratio of **1**:PPh_3_ at 1:0.6 (see above), this quantity of PPh_3_ was considered when preparing the catalyst solutions.

For the catalytic reactions at 9 bar, 25 °C, in the presence of [Ni­(COD)_2_], the ethylene uptake was also monitored along the reaction time for the three catalysts 1*−3*, by a Bronkhorst EL-Flow mass flowmeter (ref: F-111CM-500-AGD-88-K) connected to an acquisition computer by a RS232 interface. The polymerization procedure described above was followed. After pressurizing the reactor, the flowmeter was opened and allowed to equilibrate for 10 min. Data acquisition started prior to the addition of both solutions, with the time immediately following the second injection considered as *t* = 0. FlowDDE, FlowView and FlowPlot were the software used to monitor and acquire data from the setup and the computer.

### Polyethylene Characterization by ^1^H and ^13^C­{^1^H} NMR – Determination of *M*_n_, Degree of Branching and Microstructure Analysis

The polyethylene samples were dissolved in a mixture of 1,2,4-trichlorobenzene (TCB) and C_6_D_6_ (2:1 v/v) and the spectra recorded at 80 °C. The ^1^H NMR results provided information about the degree of branching (N), and the ^13^C­{^1^H} NMR results allowed the determination of the type and distribution of the branches within the polymers. The ^13^C­{^1^H} resonances characteristic of the PEs microstructure were identified as described in the literature.^54^ The distribution of branches in the PEs is detailed in Table 2 of the manuscript. The ^1^H and ^13^C­{^1^H} NMR spectra are presented in Figures S17–S42 and S43–S51 of the SI, respectively. Details of the calculations are provided in the SI.

### Polyethylene Characterization by GPC/SEC – Molecular Weights Determination

The Gel Permeation Chromatography/Size Exclusion Chromatography (GPC/SEC) analyses were conducted on two different chromatographs. Some analyses utilized a Waters GPC 150CV chromatograph, where the temperatures of the injector, column and pump sections were stabilized at 35, 35, and 30 °C, respectively. The sample elution was performed through two MesoPore columns, protected by a MesoPore Guard column (Polymer laboratories). Other analyses were carried out on a Waters HPLC chromatograph equipped with an isocratic pump (Waters 1515) and a refractive index detector (Waters 2414). Here, the oven was stabilized at 30 °C and the samples were eluted through two PolyPore columns, protected by a PolyPore Guard column (Polymer laboratories).

THF was used as the eluent, at a flow rate of 1.0 mL/min. The solvent was filtered before use through 0.45 μm PTFE membranes (Fluoropore, Millipore) and degassed in an ultrasound bath for 15–20 min. The PE samples were filtered through 0.20 μm PTFE filters (Durapore, Millipore). All acquisition and data processing were performed using Empower software.

Molecular weights were calibrated relative to polystyrene (PS) standards (TSK Tosoh Co.) and are therefore presented in PS units, which should differ from the actual values in PE units due to the variations in hydrodynamic volumes of the two polymers. All GPC/SEC chromatograms obtained are presented in Figures S52–S80 (SI).

### NMR Tube-Scale Monitoring of the Reaction of Ethylene with Precatalyst 2

Attempts to investigate the initial insertions of ethylene into the *C,N,N’*-tridentate nickel precatalyst **2** were conducted in an NMR-tube scale experiment under mild conditions (50 °C and 1 atm ethylene), using an initial ethylene:**2** molar ratio of 7:1. For this purpose, 13 μmol of precatalyst **2** were dissolved in 0.5 mL of benzene-*d*_6_, and the solution was exposed to 88 μmol of ethylene gas. Further experimental information and the corresponding ^1^H and ^1^H DOSY NMR spectra are presented in Figures S82–S84 (SI).

## Supplementary Material





## Notes

Experimental and computational details; characterization data of ligand precursors **I−III**; X-ray data, %V_Bur_ calculations, NMR spectra; GPC/SEC chromatograms (PDF)
Joint CIF files of ligand precursors **I−III**, and of complexes **1**, **1_B_**, **2** and **3** (CIF); Cartesian coordinates of calculated structures (XYZ)

## †

LAQV-REQUIMTE, Departamento de Química, Faculdade de Ciências e Tecnologia, Universidade NOVA de Lisboa, 2829-516 Caparica, Portugal

## Abbreviations

Cat.: catalyst
COD: 1,5-cyclooctadiene
PPh_3_: triphenylphosphine
DFT: Density Functional Theory
PE: polyethylene
GPC/SEC: Gel Permeation Chromatography/Size-Exclusion Chromatography
