# Supplementary material for: Unexpected Formation of Neutral Alkyl-Ni(II) Complexes Bearing Unsymmetrical C,N,N’‑Pincer Iminopyrrolyl Ligands via Intramolecular C–H Bond Activation: A Distinct Type of Single-Component Ethylene Polymerization Catalysts
Source: Inorg Chem. 2025 Dec 9;64(50):24479–96. doi: 10.1021/acs.inorgchem.5c03794 (PMC12818750; doi:10.1021/acs.inorgchem.5c03794)
Supplement: Supplementary file 1 [file ic5c03794_si_001.pdf]

Supporting Information for

Unexpected formation of neutral alkyl-Ni(II) complexes bearing unsymmetrical *C,N,N'*-pincer iminopyrrolyl ligands via intramolecular C-H bond activation: a distinct type of single-component ethylene polymerization catalysts

Cláudia A. Figueira, Patrícia S. Lopes, Ricardo Meyrelles, Clara S. B. Gomes, Joselaine C. S. Gomes, Luís F. Veiros and Pedro T. Gomes*

Centro de Química Estrutural, Institute of Molecular Sciences, Departamento de Engenharia Química, Instituto Superior Técnico, Universidade de Lisboa, Av. Rovisco Pais, 1, 1049-001 Lisboa, Portugal

* Corresponding Author; e-mail: pedro.t.gomes@tecnico.ulisboa.pt

Table of Contents

	Page
Synthetic Procedures and Characterization of Ligand Precursors **I** and **III** and Sodium Salts $\mathbf{I_{Na}}$ and $\mathbf{II_{Na}}$	S3
NMR Spectra of Complexes **1–3**	S6
X-Ray Diffraction Structural Studies	S10
X-Ray Diffraction Crystallographic Data of Ligand Precursors **I-III** and Complexes **1-3**	S10
X-Ray Crystal and Molecular Structures of Ligand Precursors **I-III**	S12
Further Details and Data on the Molecular Structures of Complexes **1-3**	S14
Calculation of the Buried Volumes of Unsymmetrical *C,N,N'*-Pincer Iminopyrrolyl Ligands in Complexes **1-3**	S17
NMR Tube-Scale Monitoring of the Formation of Complex **2**	S19
VT-^{1}H NMR Spectra of the Formation of Complex **2**	S19
VT-^{31}P{^{1}H} NMR Spectra of the Formation of Complex **2** in the Presence of Free PPh_3 (2 equiv)	S21
Computational Calculations	S23
Computed Alternate C-H Activation Pathways	S23
Atomic Coordinates	S25
Polyethylene Characterization by ^{1}H NMR - Determination of M_n and Branching Degree	S32
Determination of the M_n of Polyethylene by End-group Analysis	S32
Determination of the Degree of Branching of Polyethylene	S32
^{1}H NMR Spectra of Polyethylene Products	S33
Polyethylene Characterization by ^{13}C{^{1}H} NMR - Microstructure Analysis	S46
Determination of the Distribution of Branches (%)	S46
^{13}C{^{1}H} NMR Spectra of Selected Polyethylene Products	S47
GPC/SEC Chromatograms of Polyethylene Products	S52
Dynamic Viscosity Measurements of Selected Polyethylene Samples	S67
Possible Polymerization Mechanistic Scheme	S68
NMR Tube-Scale Monitoring of the Reaction of Ethylene with Precatalyst **2**	S69
References and Notes	S72

Synthetic Procedures and Characterization of Ligand Precursors I and III and Sodium Salts $\mathbf{I_{Na}}$ and $\mathbf{II_{Na}}$

As noted in the article, ligand precursors **I** and **III** are new compounds, whereas ligand precursor **II** was previously reported.[1] In contrast, the sodium salts of **I** and **II** ($\mathbf{I_{Na}}$ and $\mathbf{II_{Na}}$, respectively), obtained by deprotonation with NaH, were isolated and characterized here for the first time. The synthesis and characterization of ligand precursors **I**, **III**, $\mathbf{I_{Na}}$ and $\mathbf{II_{Na}}$ are provided below. The general procedures and reagents used are described in the Experimental Section of the article.

Synthesis of Ligand Precursor 5-(2,6-dimethylphenyl)-2-[*N*-(2,6-dimethylphenyl) formimino]-1*H*-pyrrole (I): In a round-bottom flask, 5-(2,6-dimethylphenyl)-2-formyl-1*H*-pyrrole (0.60 g, 3.0 mmol), 2,6-dimethylaniline (0.44 mL, 3.6 mmol), and a catalytic amount of *p*-toluenesulfonic acid (PTSA) were dissolved in toluene (20 mL). A Soxhlet extractor containing pre-activated 4 Å molecular sieves, fitted with a condenser and a $CaCl_2$ guard tube at the top, was attached to the flask, and the mixture was refluxed at 120-140 ºC for 44 h. After cooling to room temperature, the solvent was removed under vacuum, and the crude product was washed with *n*-hexane. and extracted with diethyl ether. The resulting solution was concentrated and stored at -20 ºC to afford compound **I** (0.671 g, 74%) as colorless crystals suitable for single-crystal X-ray diffraction.

Anal. Calcd. for $C_{21}H_{22}N_2$: C 83.40, H 7.33, N 9.26; **Found**: C 83.07, H 7.38, N 9.26.

^{1}H NMR (300 MHz, $CDCl_3$): δ 9.34 (br, 1H, N*H*), 7.97 (s, 1H, N=C*H*), 7.24-7.19 (m, 1H, 5-Ph-H_{para}), 7.13 (d, $^3J_{HH}$ = 7.4 Hz, 2H, 5-Ph-H_{meta}), 7.06 (d, $^3J_{HH}$ = 7.4 Hz, 2H, N-Ph-H_{meta}), 6.96-6.91 (m, 1H, N-Ph-H_{para}), 6.70 (d, $^3J_{HH}$ = 3.5 Hz, 1H, H3), 6.20 (d, $^3J_{HH}$ = 3.5 Hz, 1H, H4), 2.24 (s, 6H, 5-Ph-CH_3), 2.17 (s, 6H, N-Ph-CH_3). **^{13}C{^{1}H} NMR** (75 MHz, $CDCl_3$): δ 152.2 (N=*C*H), 150.9 (N-Ph-C_{ipso}), 138.3 (5-Ph-C_{ortho}), 135.0 (C5), 132.5 (5-Ph-C_{ipso}), 130.1 (C2), 128.5 (5-Ph-C_{para}), 128.2 (N-Ph-C_{meta}), 127.7 (5-Ph-C_{meta} and N-Ph-C_{ortho}), 123.6 (N-Ph-C_{para}), 116.4 (C3), 110.9 (C4), 20.8 (5-Ph-*C*H_3), 18.6 (N-Ph-*C*H_3).

Synthesis of Ligand Precursor 5-(2,6-dimethylphenyl)-2-{*N*-[2,6-bis(3,5-bis(trifluoromethyl)phenyl)phenyl] formimino}-1*H*-pyrrole (III): In a round-bottom flask, 5-(2,6-dimethylphenyl)-2-formyl-1*H*-pyrrole (0.90 g, 4.5 mmol), 2,6-bis[3,5-bis(trifluoromethyl)phenyl]aniline (2.17 g, 4.2 mmol), and a catalytic amount of *p*-toluenesulfonic acid (PTSA) were dissolved in toluene (50 mL). A Soxhlet extractor containing

pre-activated 4 Å molecular sieves, fitted with a condenser and a $CaCl_2$ guard tube at the top, was attached to the flask, and the mixture was refluxed at 120-140 ºC for 107 h. After cooling to room temperature, the solvent was removed under vacuum, and the crude product was extracted with *n*-hexane. The resulting solution was concentrated and stored at -20 ºC to afford compound **III** as a yellow powder (1.26 g, 43%). Yellow crystals suitable for single-crystal X-ray diffraction were obtained from a concentrated *n*-hexane solution at -20 ºC.

Anal. Calcd. for $C_{35}H_{22}F_{12}N_2$: C 60.18, H 3.17, N 4.01; **Found**: C 59.88, H 3.02, N 3.91.

^{1}H NMR ($CDCl_3$, 300 MHz): δ 8.80 (br, 1H, N*H*), 7.93 (s, 4H, CF_3-Ph-H_{ortho}), 7.77 (s, 2H, CF_3-Ph-H_{para}), 7.61 (s, 1H, C*H*N), 7.52 (d, 2H, $^3J_{HH}$ = 7.1 Hz, N-Ph-H_{meta}), 7.38 (t, 1H, $^3J_{HH}$ = 7.1 Hz, N-Ph-H_{para}), 7.20 (t, 1H, $^3J_{HH}$ = 7.3 Hz, 5-Ph-H_{para}), 7.09 (d, 2H, $^3J_{HH}$ = 7.3 Hz, 5-Ph-H_{meta}), 6.44 (s, 1H, H3), 6.06 (s, 1H, H4), 2.08 (s, 6H, CH_3). **^{13}C{^{1}H} NMR** ($CDCl_3$, 75 MHz): δ 155.2 (*C*HN), 149.1 (N-Ph-C_{ipso}), 141.8 (N-Ph-C_{ortho}), 138.5 (5-Ph-C_{ortho}), 136.8 (C5), 132.2 (5-Ph-C_{ipso}), 131.6 (q, $^2J_{CF}$ = 20.3 Hz, CF_3-Ph-C_{meta}), 131.2 (N-Ph-C_{meta}), 130.9 (CF_3-Ph-C_{ipso}), 130.4 (CF_3-Ph-C_{ortho}), 129.4 (C2), 128.8 (5-Ph-C_{para}), 127.4 (5-Ph-C_{meta}), 125.3 (N-Ph-C_{para}), 123.4 (q, $^1J_{CF}$ = 263.3 Hz, *C*F_3), 120.6 (CF_3-Ph-C_{para}), 118.6 (C3), 111.2 (C4), 20.0 (CH_3). **^{19}F{^{1}H} NMR** (282 MHz, $CDCl_3$): δ -62.9 (CF_3).

Synthesis of Ligand Precursor 5-(2,6-dimethylphenyl)-2-[*N*-(2,6-dimethylphenyl) formimino]pyrrolyl Sodium Salt (I_{Na}): Ligand precursor **I** (0.393 g, 1.30 mmol) was suspended in THF (10 mL) and added dropwise to a Schlenk tube containing NaH powder (0.035 g, 1.46 mmol) under stirring. Immediate evolution of hydrogen was observed. After complete addition, the mixture was refluxed at 90 ºC for 2 h. The reaction was then cooled to room temperature, and the solution was filtered; the NaH residue was further washed with THF. The combined THF solutions were evaporated to dryness under vacuum, affording an orange foam. After several hours drying under vacuum, the material was ground to yield the THF adduct (1:1) of **I_{Na}** as a fine salmon-colored powder (0.504 g, 97%).

^{1}H NMR (400 MHz, CD_3CN): δ 7.69 (s, 1H, N=C*H*), 7.02-7.00 (m, 5H, N-Ph-H_{meta}, 5-Ph-H_{meta} and N-Ph-H_{para}), 6.82 (t, $^3J_{HH}$ = 7.5 Hz, 1H, 5-Ph-H_{para}), 6.56 (d, $^3J_{HH}$ = 3.0 Hz, 1H, H3), 5.92 (d, $^3J_{HH}$ = 2.9 Hz, 1H, H4), 3.66-3.63 (m, 4H, (2,5)-CH_2 THF), 2.18 (s, 6H, 5-Ph-CH_3), 2.11 (s, 6H, *N*-Ph-CH_3), 1.82-1.79 (m, 4H, (3,4)-CH_2 THF). **^{13}C{^{1}H} NMR** (101 MHz, CD_3CN): δ 159.5 (N=*C*H), 154.7 (N-Ph-C_{ipso}), 148.0 (C5), 142.7 (5-Ph-C_{ipso}), 139.4 (C2), 138.4 (N-Ph-C_{ortho}), 130.2 (5-Ph-C_{ortho}), 128.7 (5-Ph-C_{meta}), 127.7 (N-Ph-C_{meta}), 126.4 (N-Ph-C_{para}), 122.9

(5-Ph-C_{para}), 120.3 (C3), 110.7 (C4), 68.3 ((2,5)-CH_2 THF), 26.3 ((3,4)-CH_2 THF), 21.5 (5-Ph-CH_3), 19.0 (N-Ph-CH_3). ^{23}**Na NMR** (106 MHz, CD_3CN): δ 3.76 (s, $\Delta\nu_{½}$ = 518 Hz).

Synthesis of Ligand Precursor 5-(2,6-dimethylphenyl)-2-[*N*-(2,6-diisopropylphenyl) formimino] pyrrolyl Sodium Salt (II_{Na}): Ligand precursor **II** (0.466 g, 1.30 mmol) was suspended in THF (10 mL) and added dropwise to a Schlenk tube containing NaH powder (0.035 g, 1.46 mmol) under stirring. Immediate evolution of hydrogen was observed. After complete addition, the mixture was refluxed at 90 ºC for 2 h. The reaction was then cooled to room temperature, and the solution was filtered; the NaH residue was further washed with THF. The combined THF solutions were evaporated to dryness under vacuum, affording an oil, which was further dried under vacuum to give a golden-brown foam. This material was ground to yield the THF adduct (1:1) of **II_{Na}** as a crystalline golden-brown solid (0.568 g, 99%).

^{1}**H NMR** (400 MHz, CD_3CN): δ 7.70 (s, 1H, N=C*H*), 7.11 (d, $^3J_{HH}$ = 7.6 Hz, 2H, N-Ph-H_{meta}), 7.04 (br, 3H, 5-Ph-H_{meta} and N-Ph-H_{para}), 7.01-6.98 (m, 1H, 5-Ph-H_{para}), 6.58 (s, 1H, H3), 5.94 (s, 1H, H4), 3.66-3.63 (m, 3.2H, (2,5)-CH_2 THF), 3.24-3.14 (m, 2H, C*H*$(CH_3)_2$), 2.22 (s, 6H, CH_3), 1.84-1.77 (m, 3.2H, (3,4)-CH_2 THF), 1.15 (d, $^3J_{HH}$ = 6.9 Hz, 12H, $CH(CH_3)_2$). ^{13}**C{^{1}H} NMR** (101 MHz, CD_3CN): δ 159.6 (N=*C*H), 141.1 (N-Ph-C_{ipso}), 138.5 (5-Ph-C_{ortho} and N-Ph-C_{ortho}), resonances absent (C5, 5-Ph-C_{ipso} and C2), 127.7 (5-Ph-C_{meta}), 126.5 (N-Ph-C_{para}), 123.7 (N-Ph-C_{meta} and 5-Ph-C_{para}), 120.5 (C3), 110.8 (C4), 68.3 ((2,5)-CH_2 THF), 28.4 (*C*H$(CH_3)_2$), 26.3 ((3,4)-CH_2 THF), 24.2 ($CH(CH_3)_2$), 21.5 (CH_3). ^{23}**Na NMR** (106 MHz, CD_3CN): δ 3.63 (s, $\Delta\nu_{1/2}$ = 476 Hz).

***In Situ* Synthesis of 5-(2,6-dimethylphenyl)-2-{*N*-[2,6-bis(3,5-bis(trifluoromethyl) phenyl)phenyl]formimino}pyrrolyl Sodium Salt (III_{Na}):** Ligand precursor **III** (1.05 g, 1.5 mmol) was suspended in THF (10 mL) and added dropwise to a Schlenk tube containing NaH powder (0.05 g, 2.0 mmol) under stirring. After addition, the mixture was refluxed for 2 h, cooled to room temperature, and filtered to remove the excess NaH. The solvent was then removed under vacuum to afford sodium salt **III_{Na}** as a powder, which was dried and used *in situ* without further purification or characterization.

NMR Spectra of Complexes 1–3

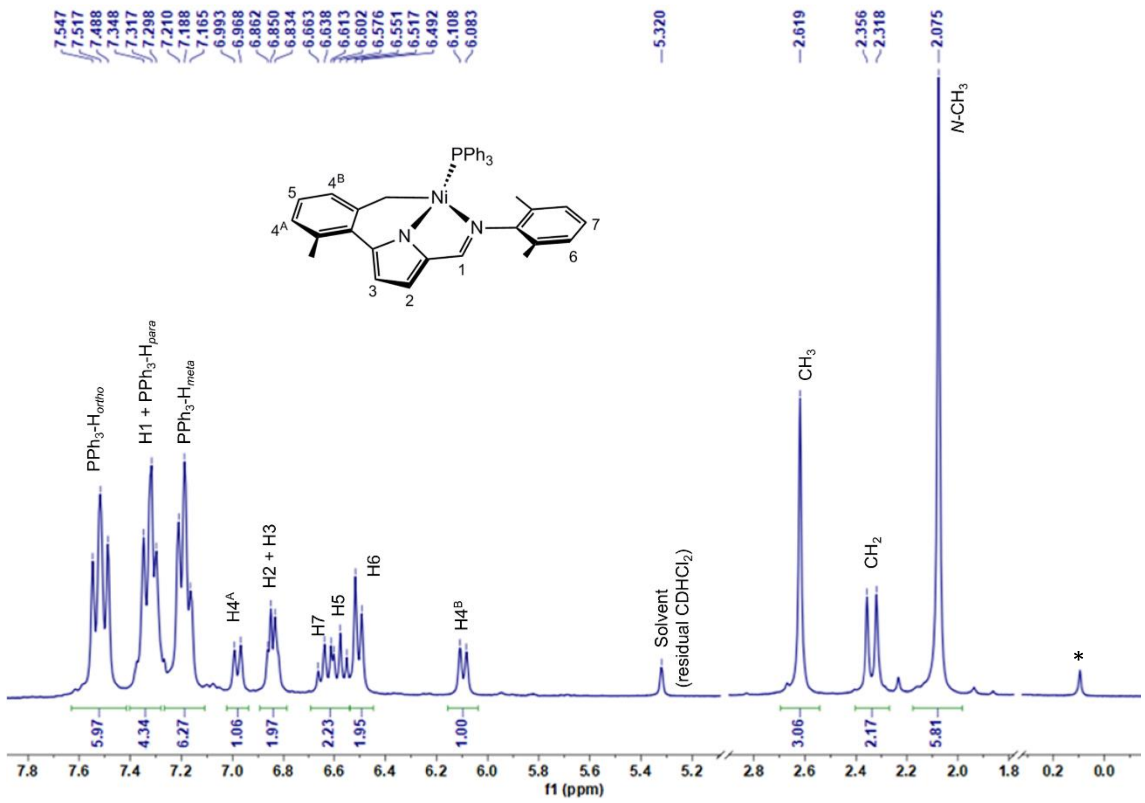

Figure S1 ^{1}H NMR (300 MHz, CD_2Cl_2) spectrum of complex **1** (grease denoted by ∗).

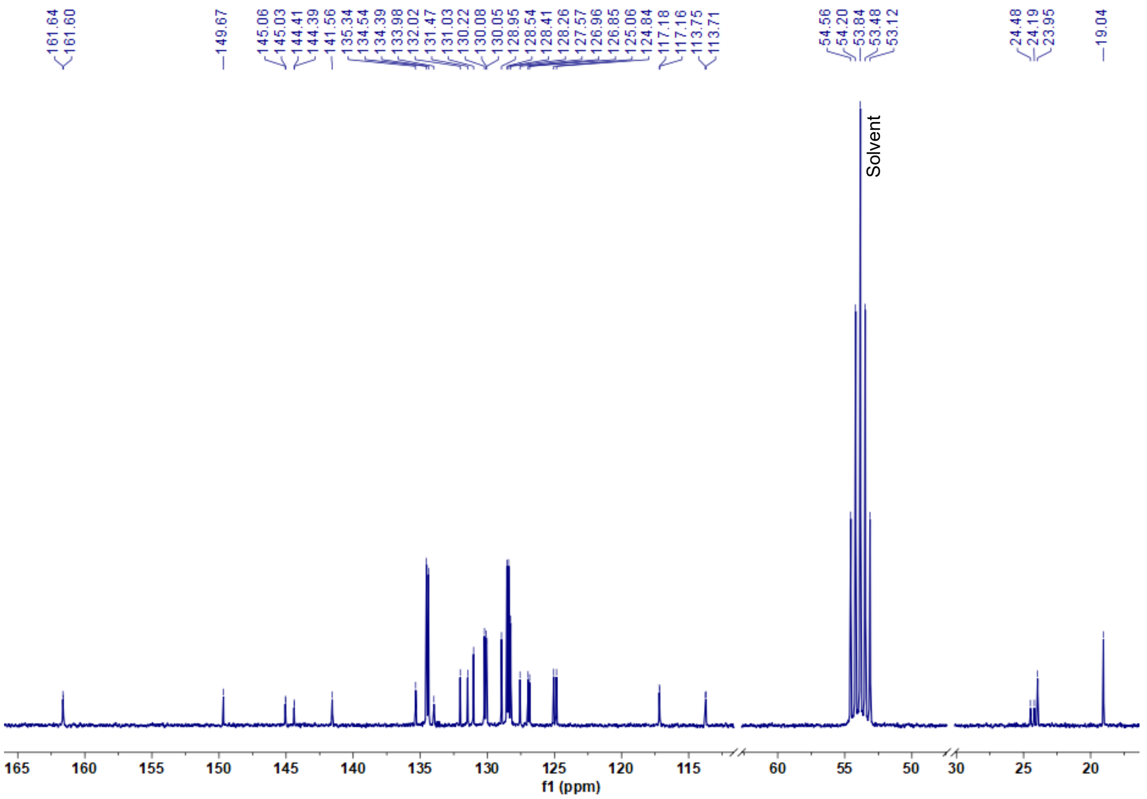

Figure S2 $^{13}C\{^{1}H\}$ NMR (75 MHz, CD_2Cl_2) spectrum of complex **1**.

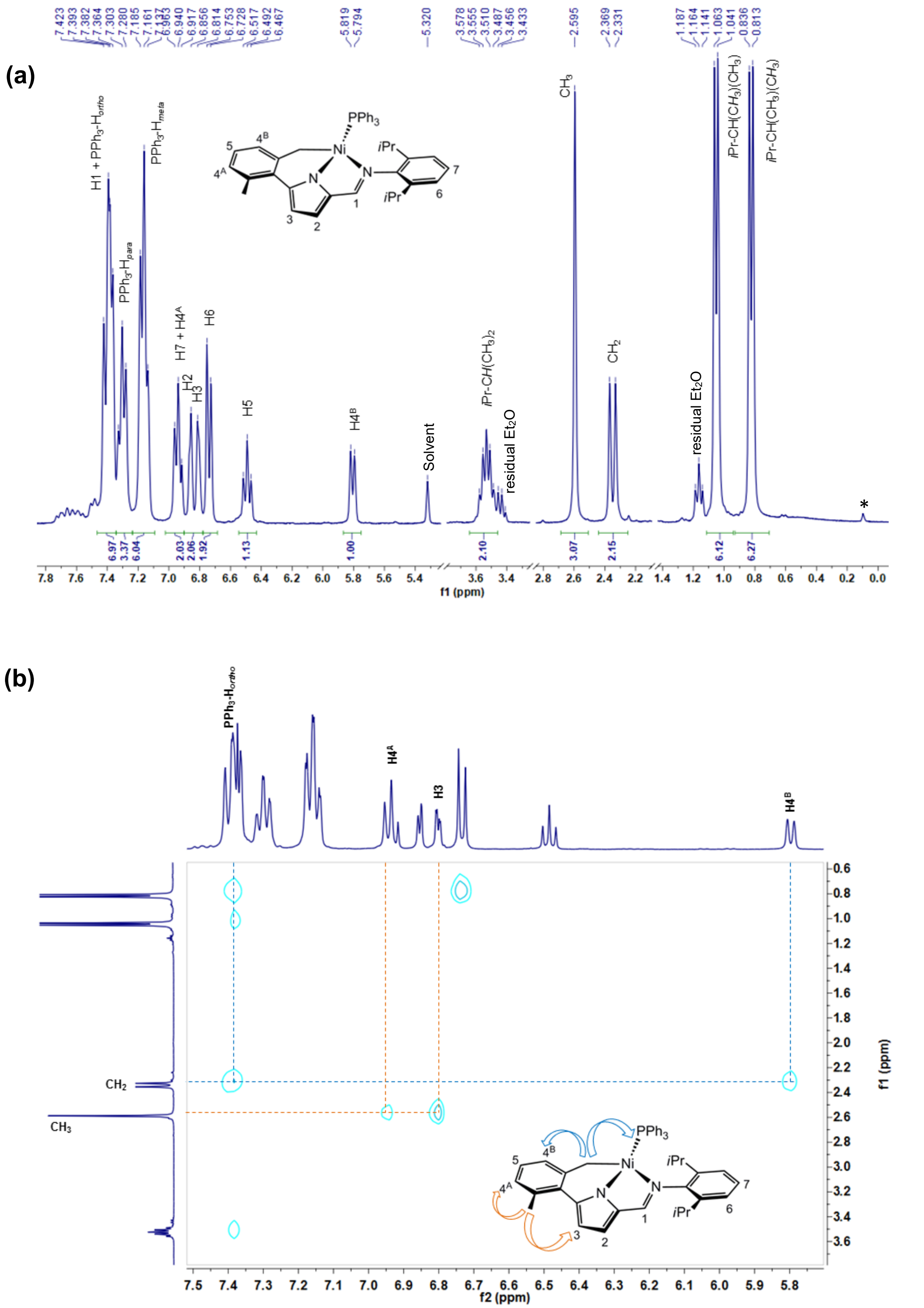

Figure S3 (a) ^{1}H NMR (300 MHz, CD_2Cl_2) spectrum (grease denoted by ∗) and (b) section of the ^{1}H-^{1}H NOESY spectrum with the identification of space correlations of the substituents in the 5-aryl ring (for CH_3 in orange and for CH_2 in blue), of complex **2**.

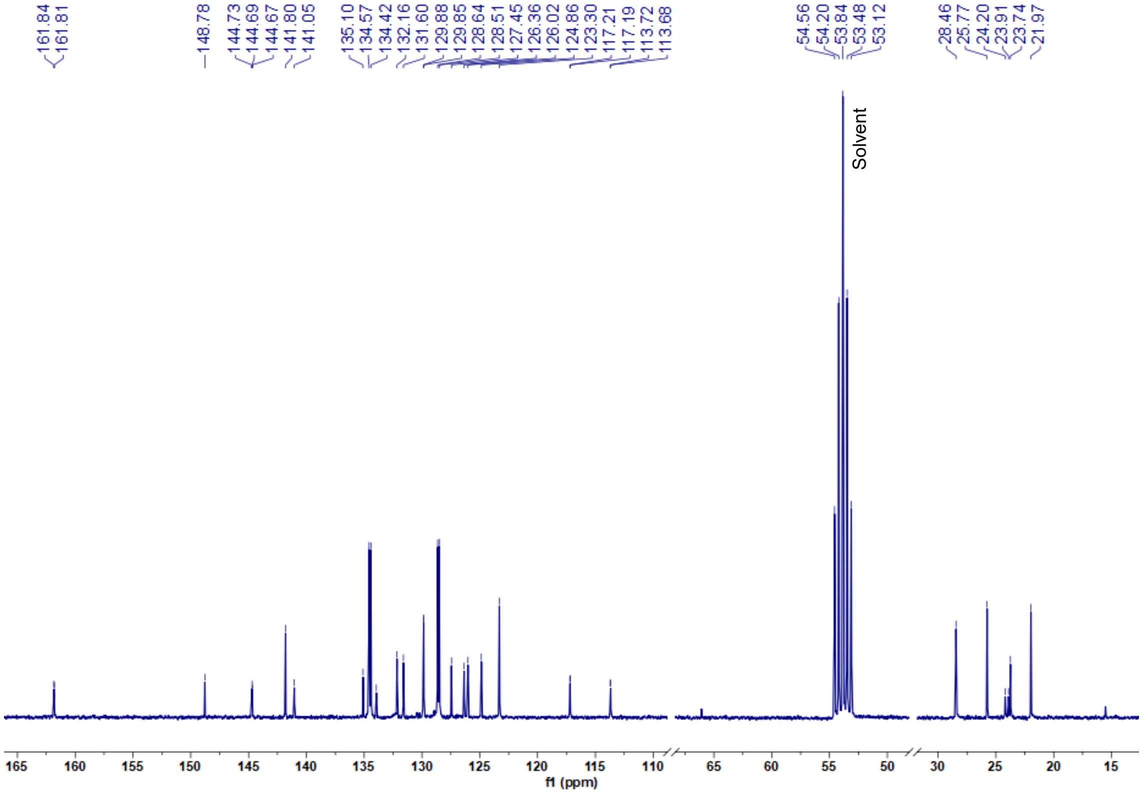

Figure S4 $^{13}C\{^{1}H\}$ NMR (75 MHz, CD_2Cl_2) spectrum of complex **2**.

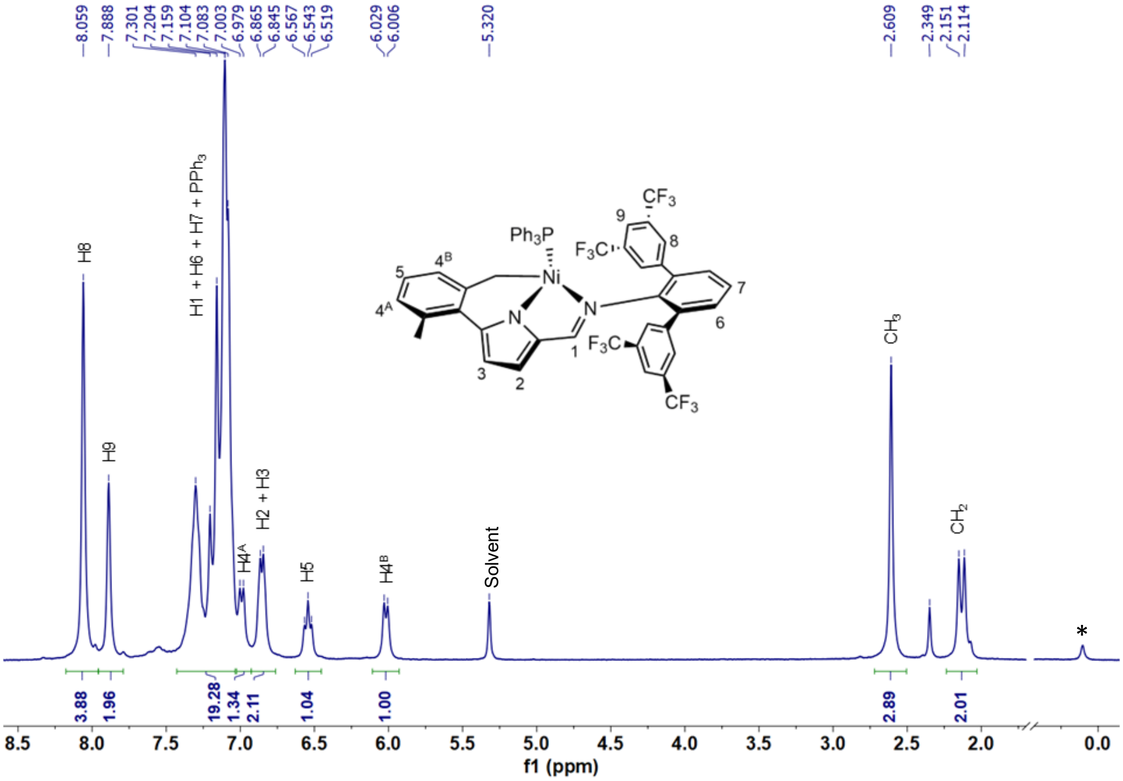

Figure S5 ^{1}H NMR (300 MHz, CD_2Cl_2) spectrum of complex **3** (grease denoted by ∗).

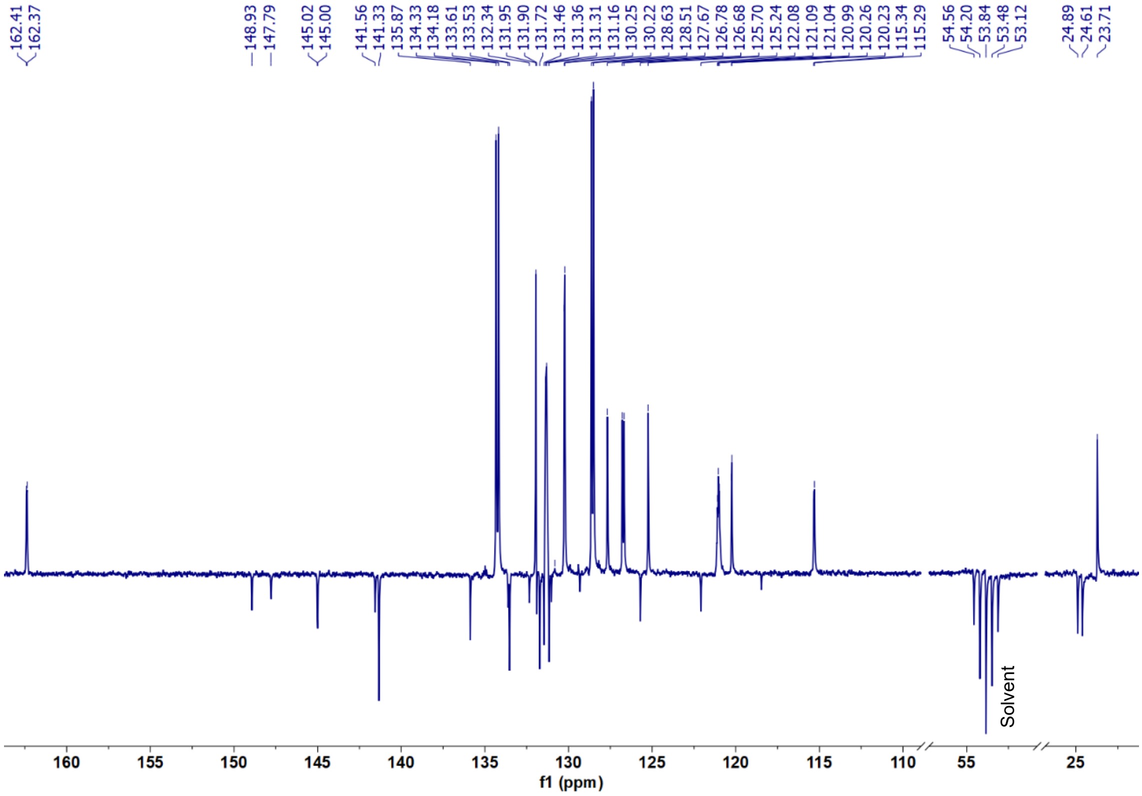

Figure S6 ^{13}C APT NMR (75 MHz, CD_2Cl_2) spectrum of complex **3**.

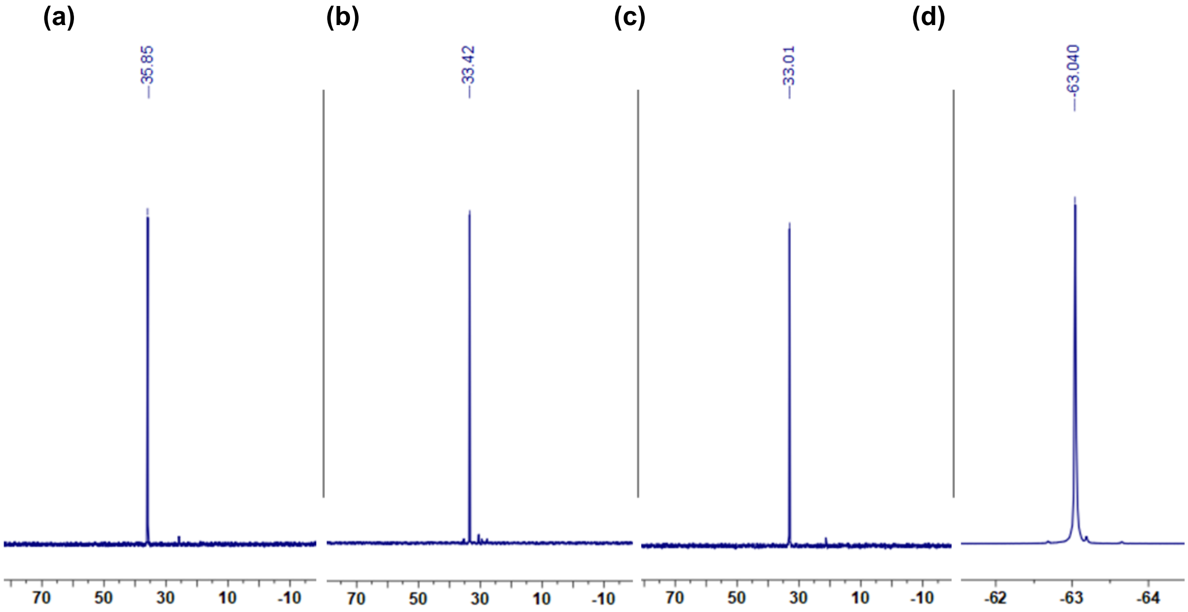

Figure S7 (a)–(c) $^{31}P\{^1H\}$ NMR (121 MHz, CD_2Cl_2) spectra of complexes: **1**, **2** and **3**, respectively, and (d) $^{19}F\{^1H\}$ NMR (282 MHz, CD_2Cl_2) spectrum of complex **3**.

X-Ray Diffraction Structural Studies

X-Ray Diffraction Crystallographic Data of Ligand Precursors I-III and Complexes 1-3:

Table S1 Crystallographic data and refinement details for the structures of the ligand precursors 5-(2,6-dimethylphenyl)-2-(*N*-(2,6-di(R)phenylformimino)pyrroles **I** (R = Me), **II** (R = *i*Pr) and **III** (R = 3,5-$(CF_3)_2C_6H_2$).

	**I**	**II**	**III**
Formula	$C_{21}H_{22}N_2$	$C_{25}H_{30}N_2$	$C_{35}H_{22}F_{12}N_2$
M	302.40	358.51	698.55
λ (Å)	0.71073	0.71073	0.71073
T (K)	150(2)	150(2)	150(2)
Crystal system	Orthorhombic	Orthorhombic	Triclinic
Space group	*Pbca*	$Pna2_1$	*P*-1
a (Å)	15.682(2)	15.277(3)	8.6059(16)
b (Å)	18.813(2)	8.2318(13)	13.728(3)
c (Å)	23.023(3)	33.145(6)	14.320(3)
α (°)	90	90	104.898(9)
β (°)	90	90	105.926(9)
γ (°)	90	90	93.975(9)
V ($Å^3$)	6792.6(17)	4168.1(12)	1554.3(6)
Z	16	8	2
ρ_{calc} ($g.cm^{-3}$)	1.183	1.143	1.493
μ (mm^{-1})	0.069	0.066	0.138
Crystal size	0.20×0.18×0.10	0.32×0.22×0.10	0.20×0.15×0.12
Crystal color	Colourless	Red	Yellow
Crystal description	Prism	Prism	Prism
θ_{max} (°)	25.734	25.735	25.772
Total data	32803	19976	12368
Unique data	6449	7541	5855
R_{int}	0.1546	0.1455	0.0429
$R[I>2\sigma(I)]$	0.0569	0.0797	0.0666
R_w	0.0874	0.1504	0.2032
Goodness of fit	1.043	0.881	1.018
ρ_{min}	-0.258	-0.247	-0.621
ρ_{max}	0.238	0.317	0.586

Table S2 Crystallographic data and refinement details for the structures of the iminopyrrolyl nickel bidentate complex **1_B**, and tridentate complexes **1**–**3**.

	**1_B**	**1**	**2**	**3**
Formula	$C_{45}H_{40}ClN_2NiP$	$C_{39}H_{35}N_2NiP \cdot C_{18}H_{15}P$	$C_{43}H_{43}N_2NiP$	$C_{53}H_{35}F_{12}N_2NiP$
M	733.92	883.64	677.47	1017.51
λ (Å)	0.71073	0.71073	0.71073	0.71073
T (K)	150(2)	150(2)	150(2)	150(2)
Crystal system	Monoclinic	Triclinic	Monoclinic	Monoclinic
Space group	$P2_1/n$	*P*-1	$P2_1/n$	$P2_1/c$
a (Å)	17.2975(12)	13.336(4)	11.7796(13)	10.130(2)
b (Å)	13.6209(11)	13.941(4)	23.658(3)	16.566(4)
c (Å)	18.1254(14)	14.730(4)	13.3814(18)	27.959(5)
α (°)	90	104.866(12)	90	90
β (°)	97.681(3)	90.355(14)	110.391(4)	98.066(10)
γ (°)	90	114.606(12)	90	90
V (Å^3)	4232.2(6)	2386.5(12)	3495.5(7)	4645.6(17)
Z	4	2	4	4
ρ_{calc} (g.cm^{-3})	1.152	1.230	1.287	1.455
μ (mm^{-1})	0.590	0.513	0.634	0.540
Crystal size	0.30×0.20×0.18	0.20×0.10×0.08	0.18×0.16×0.12	0.20×0.16×0.12
Crystal color	Red	Red	Orange-red	Orange
Crystal description	Prism	Prism	Block	Prism
θ_{max} (º)	26.252	26.079	25.726	25.776
Total data	44007	32496	22043	26406
Unique data	8505	9132	6650	8861
R_{int}	0.0757	0.0682	0.0905	0.1360
$R[I>2\sigma(I)]$	0.0877	0.0430	0.0520	0.1161
R_w	0.2583	0.0963	0.0937	0.3047
Goodness of fit	1.091	1.004	0.932	1.085
ρ_{min}	-1.153	-0.384	-0.544	-1.583
ρ_{max}	1.121	0.606	0.554	2.119

X-Ray Crystal and Molecular Structures of Ligand Precursors I-III: The iminopyrrolyl ligand precursors **I** and **II** crystallized in the orthorhombic crystal system, in the *Pbca* space group for **I** and $Pna2_1$ for **II**, both having two molecules in the asymmetric unit, A and B. Ligand precursor **III** crystallized in the triclinic system, *P*-1 space group, and a single molecule is present in the asymmetric unit. Figure S8 shows the perspective views of their molecular structures. The bond lengths and angles are similar in all these molecules, with the iminic C=N bond coplanar with the pyrrolyl ring. The C2-C6 bond length is also typically shorter than that of C5-C7, due to the extension of the pyrrole ring π-electronic delocalization toward the imine function.

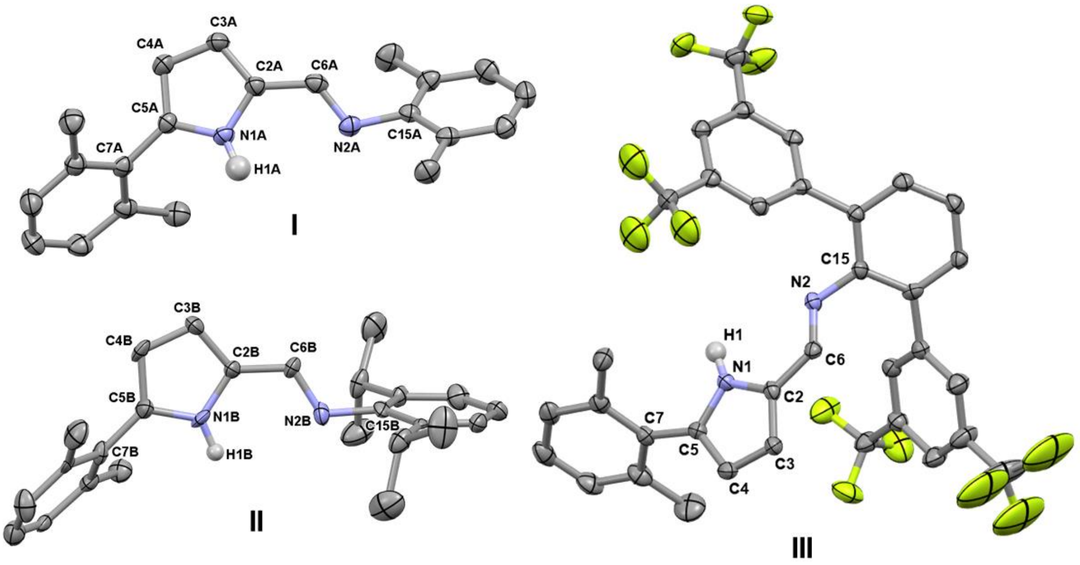

Figure S8 Perspective views of the molecular structures of the 2-iminopyrrole ligand precursors **I**–**III**, using 50% probability level ellipsoids for **I** (molecule A) and **II** (molecule B) and 40% for **III**. The hydrogen atoms were omitted for clarity, except for the N*H* ones.

Ligand precursors **I** and **II** differ in the substituents of the iminic phenyl ring (2,6-dimethyl in **I** and 2,6-diisopropyl in **II**), which confers a higher dihedral to the angle between the aryl iminic ring and the iminopyrrole plane in **II**. Nevertheless, the dihedral angle between these two planes is also different due to the different packing of the molecules. In **I**, as previously observed for other iminopyrrole compounds with the 2,6-dimethylphenylformimino substituent,[2] there is formation of dimers across two complementary hydrogen-bond interactions of the type N-H···N(Ar)=C, with a dihedral angle of 45.87º between dimer fragments (Figure S9A,B). However, this is a limiting case, since in **II** and **III**, due to the steric constraints imposed by the 5-aryl substituents and the superior steric hindrance of the 2,6-substituents in the *N*-

phenylimino groups, they block the approach between the molecules and the dimers cannot be formed. Nonetheless, in **II** each pair of molecules A and B of the asymmetric unit can organize in pseudo-dimers by complementary interactions of the type N-H···πC (Figure S13C). In **III** the molecules also adopt a supramolecular arrangement organized in pseudo-dimers, in this case through the establishment of complementary N-H···πC and C-H···N(Ar)=C intermolecular interactions (Figure S9D).

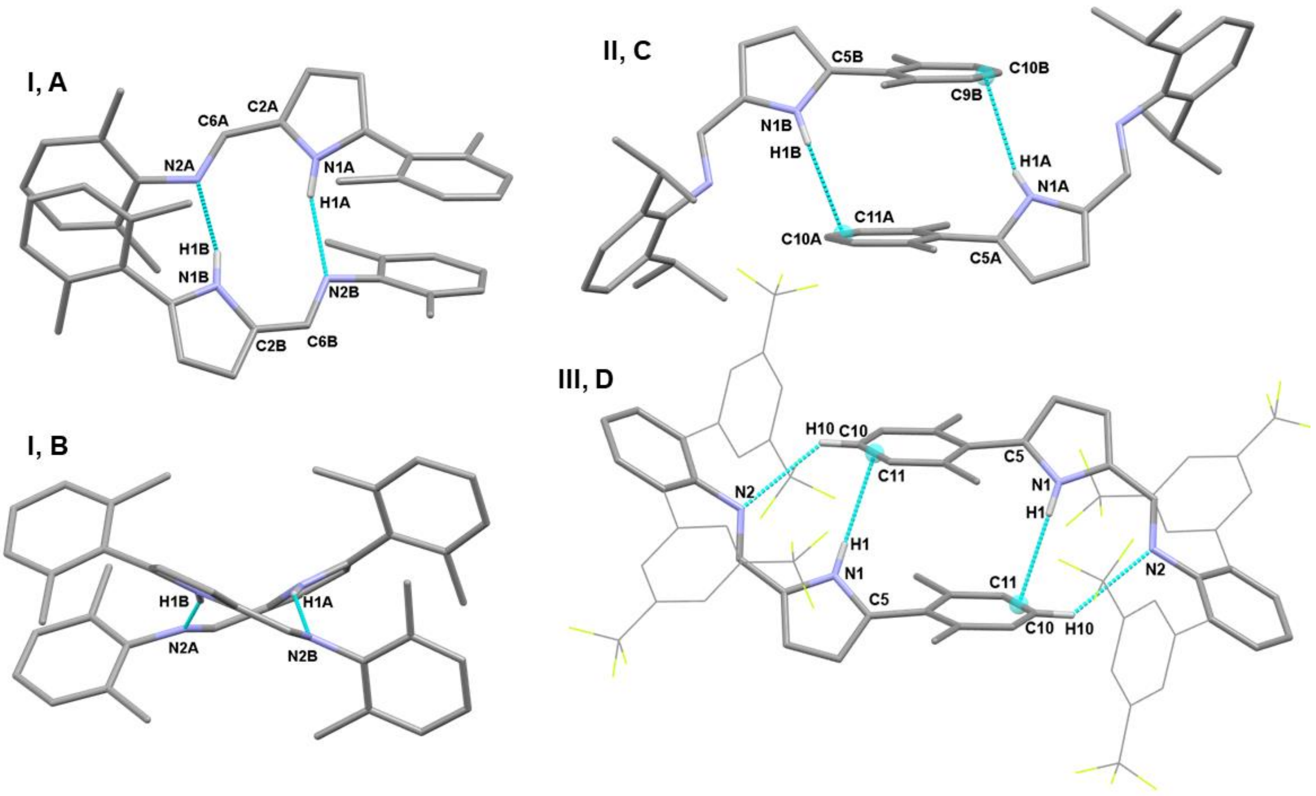

Figure S9 A) Complementary N-H···N hydrogen-bonds between sterically constrained dimers in **I**; **B)** Side-view of the dimers in **I**, with a dihedral angle of 47.94º between the two iminopyrrolyl planes of the dimer; **C)** Pseudo-dimerization through N-H···πC intermolecular interactions in **II**; **D)** Pseudo-dimerization through N-H···πC and C-H···N(Ar)=C intermolecular interactions in **III**.

Further Details and Data on the Molecular Structures of Complexes 1-3:

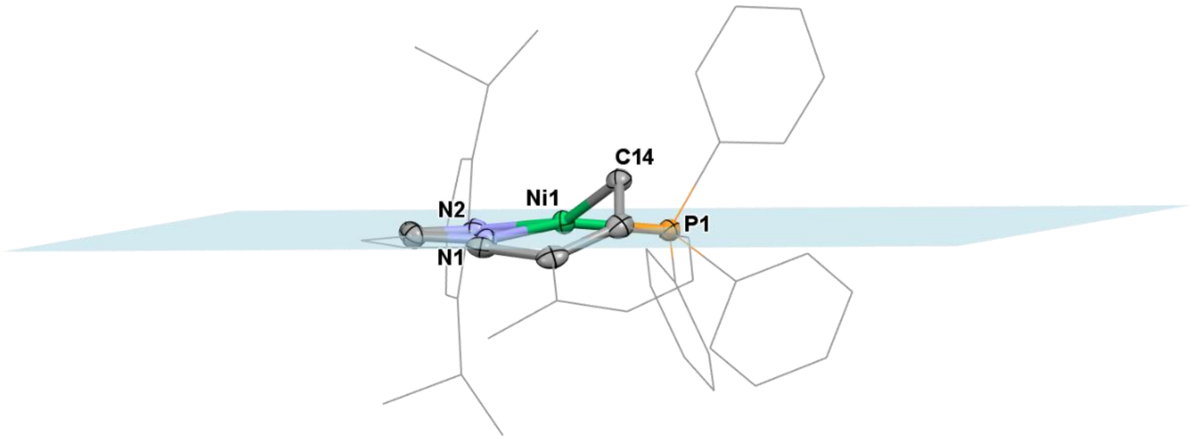

Figure S10 Representation of the plane defined by atoms (N1-Ni1-N2-P1) in complex **2**, emphasizing the envelope conformation of the six-membered chelate ring.

Table S3 Selected bond distances (Å), angles (º) and dihedral angles between (a) *N*-(2,6-aryl)formimino and 2-iminopyrrole[a] ring planes and (b) 5-aryl and pyrrole ring planes, for compounds **I** to **III**.

	**I**		**II**		**III**
Distances (Å)	**Molecule A**	**Molecule B**	**Molecule A**	**Molecule B**	
N1-C2	1.378(3)	1.378(3)	1.392(11)	1.366(11)	1.366(4)
C2-C3	1.374(3)	1.371(3)	1.381(12)	1.397(12)	1.382(4)
C3-C4	1.400(3)	1.402(3)	1.414(12)	1.400(12)	1.396(4)
C4-C5	1.380(3)	1.372(3)	1.392(13)	1.375(13)	1.381(4)
C5-N1	1.368(3)	1.367(3)	1.392(11)	1.368(11)	1.365(4)
C5-C7	1.472(4)	1.479(3)	1.481(13)	1.461(12)	1.476(4)
C2-C6	1.432(3)	1.415(3)	1.450(12)	1.461(12)	1.431(4)
C6-N2	1.276(3)	1.276(3)	1.297(13)	1.292(13)	1.285(4)
N2-C*ipso*	1.438(3)	1.431(3)	1.395(13)	1.446(13)	1.403(4)
Angles (º)					
C5-N1-C2	109.5(2)	109.9(2)	109.3(7)	109.3(7)	110.2(3)
N1-C2-C3	107.2(2)	107.0(2)	107.9(8)	106.4(7)	107.3(3)
C2-C3-C4	108.2(2)	107.8(2)	107.6(8)	106.1(8)	107.4(3)
C3-C4-C5	107.4(2)	108.0(2)	110.5(9)	110.5(9)	108.1(3)
C4-C5-N1	107.7(2)	107.2(2)	106.8(8)	104.7(8)	106.9(3)
C7-C5-N1	123.5(2)	122.5(2)	122.5(9)	124.0(9)	124.1(3)
N1-C2-C6	125.0(2)	124.7(2)	121.3(8)	124.0(8)	124.7(3)
C2-C6-N2	125.5(2)	123.8(2)	123.3(9)	121.3(9)	123.9(3
C6-N2-C*ipso*	116.1(2)	119.3(2)	117.6(9)	119.1(9)	117.4(3)
Dihedral Angles (º)					
2-*N*-2,6-Ar/ NC_4H_2(N1-C2-C6-N2)	58.50(13)	57.37(13)	75.6(4)	80.3(5)	43.43(19)
5-Ar/NC_4H_2	58.29(10)	64.16(10)	62.7(4)	62.2(4)	55.48(13)
Dimer	45.68(10)		-		-

[a] The 2-iminopyrrole ring plane is defined by atoms N1-C2-C6-N2, thus including part of the pyrrole ring (atoms N1 and C2) and of the iminic bond (atoms C6 and N2).

Table S4 Selected bond distances (Å) and angles (º) for complexes **1-3** and **1B**.

Distances (Å)	**1B**	**1**	**2**	**3**
Ni1-N1	1.9382(17)	1.8690(18)	1.873(2)	1.874(7)
Ni1-N2	2.0094(18)	1.989(2)	2.032(2)	2.035(7)
Ni1-P1	2.1861(6)	2.1391(8)	2.1590(9)	2.166(2)
Ni1-C_{ipso}	1.894(2)	-	-	-
Ni1-C14	-	1.936(2)	1.950(3)	1.941(8)
N1-C2	1.393(3)	1.371(3)	1.367(4)	1.395(11)
C2-C6	1.406(3)	1.408(3)	1.401(4)	1.379(12)
C6-N2	1.304(3)	1.298(3)	1.306(4)	1.311(10)
N2-C15	1.441(3)	1.425(3)	1.436(4)	1.424(10)
N1-C5	1.361(3)	1.358(3)	1.360(4)	1.362(11)
C5-C7	1.490(3)	1.466(3)	1.459(4)	1.470(13)
C7-C12	1.406(3)	1.411(3)	1.413(4)	1.408(12)
C12-C14	1.505(3)	1.484(3)	1.490(4)	1.514(12)
C8-C13	1.509(3)	1.503(3)	1.504(5)	1.507(13)
Cl1-C24	1.751(3)	-	-	-
Angles (º)				
N1-Ni1-N2	83.40(7)	82.67(8)	81.91(10)	82.9(3)
N2-Ni1-P1	100.86(5)	106.72(6)	109.23(7)	108.0(2)
N1-Ni1-C14	-	85.72(9)	84.50(12)	84.4(3)
C14-Ni1-P1	-	87.59(7)	89.35(10)	87.8(3)
N1-Ni1-C_{ipso}	90.93(8)	-	-	-
P1-Ni1-C_{ipso}	84.19(7)	-	-	-
Ni1-N1-C2	-	114.59(15)	115.6(2)	114.2(5)
Ni1-N1-C5	143.45(15)	136.97(15)	135.9(2)	138.8(6)
Ni1-N2-C6	109.35(14)	111.48(15)	109.8(2)	108.8(6)
C6-N2-C15	115.07(18)	116.56(19)	115.7(3)	116.2(7)
Ni1-N2-C15	135.34(14)	131.89(15)	134.43(19)	134.2(5)
C5-N1-C2	105.65(17)	108.27(18)	108.1(3)	106.9(7)
N1-C5-C7	126.92(18)	117.76(19)	118.1(3)	118.3(8)
C5-C7-C12	-	118.5(2)	117.6(3)	117.1(8)
N1-C2-C6	115.23(18)	113.4(2)	112.9(3)	109.7(8)
C7-C12-C14	-	122.0(2)	122.4(3)	124.3(8)
C12-C14-Ni1	-	117.27(15)	117.5(2)	119.7(6)
C2-C6-N2	118.4(2)	117.3(2)	118.6(3)	120.1(8)
N2-Ni1-C14	-	161.36(9)	155.26(12)	159.3(3)
N2-Ni1-C_{ipso}	174.10(8)	-	-	-
N1-Ni1-P1	164.14(6)	165.87(6)	162.39(8)	164.8(2)

Table S5 Dihedral angles and τ_4 parameters for bidentate complex $\mathbf{1_B}$ and tridentate complexes **1**-**3**.

Dihedral Angles (º)	$\mathbf{1_B}$	**1**	**2**	**3**
2-*N*-2,6-Ar/N1-C2-C6-N2	74.96(11)	85.97(12)	84.06(15)	73.0(4)
5-(2-CH_2-6-Me)C_6H_3/NC_4H_2	-	21.81(8)	28.87(12)	22.7(3)
N1-Ni1-N2/P1-Ni1-C14	-	18.39(7)	25.01(11)	19.8(3)
5-Ar/NC_4H_2	71.03(9)	-	-	-
N1-Ni1-N2/P1-Ni1-C_{ipso}	15.17(6)	-	-	-
τ_4 [a]	0.16	0.23	0.30	0.26

[a] Ref. 3

Calculation of the Percentage of Buried Volume (%V_{Bur})[4] of *C,N,N'*-Pincer Iminopyrrolyl Ligands in Complexes 1-3:

Table S6 %V_{Bur} of *C,N,N'*-iminopyrrolyl ligands in Ni complexes **1**-**3** for sphere radii of 3.5, 5, 7 and 7 Å.

Complex	%V_{Bur} [a]			
	**3.5 Å**	**5 Å**	**7 Å**	**9 Å**
**1**	57.7	43.3	24.1	11.7
**2**	57.0	46.1	29.2	14.2
**3**	63.7	55.4	38.4	21.3

[a] Calculations performed using the programs described in Refs. 5 and 6.

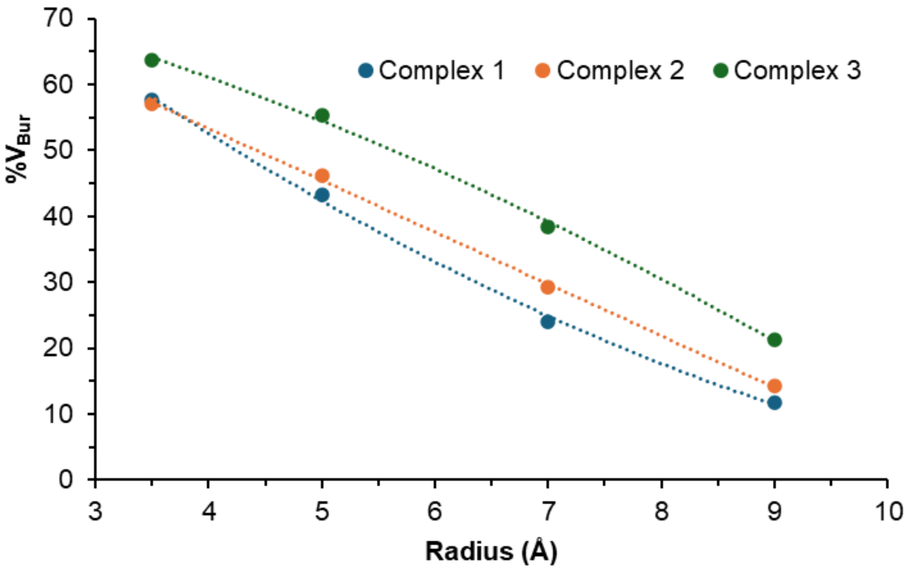

Figure S11 Distance dependence of %V_{Bur} for the *C,N,N'*-iminopyrrolyl ligands in Ni complexes **1**-**3**.

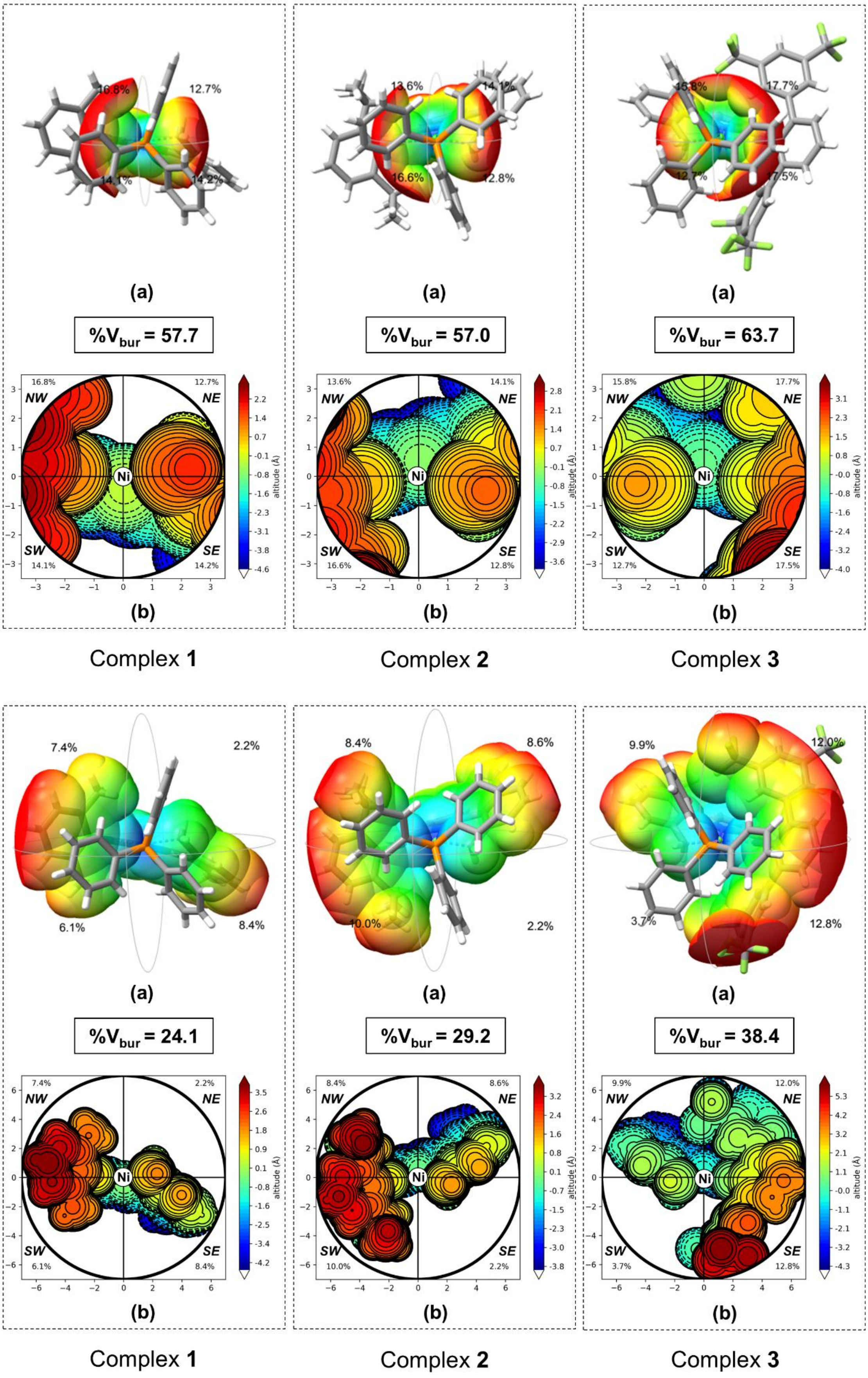

Figure S12 (a) Visualization of buried volumes of the *C,N,N'*-iminopyrrolyl ligands in Ni complexes **1**-**3**, and (b) corresponding steric maps with $\%V_{Bur}$ parameters, calculated for sphere radii of 3.5 Å (top) and 7 Å (bottom).

NMR Tube-Scale Monitoring of the Formation of Complex 2

VT-^{1}H NMR Spectra of the Formation of Complex 2:

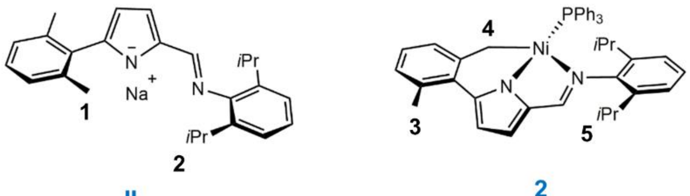

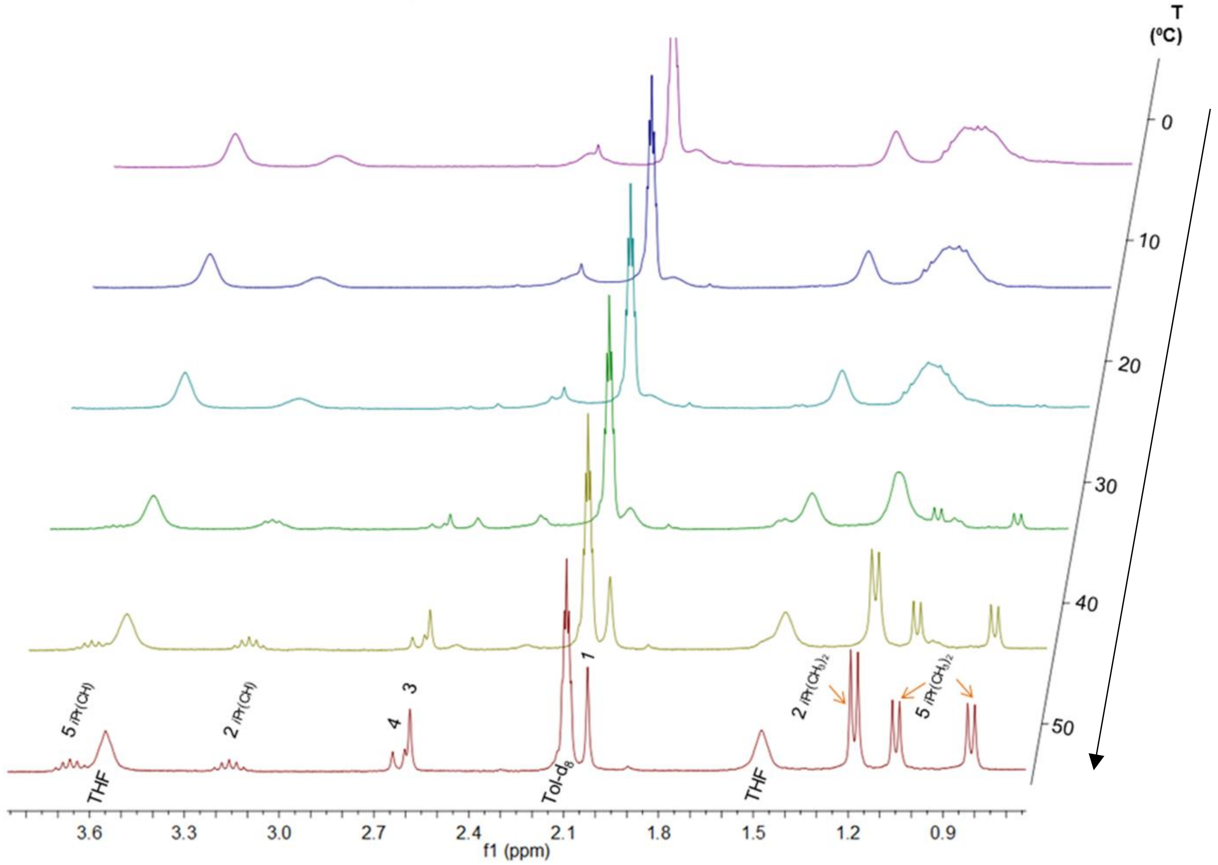

Figure S13 VT-^{1}H NMR spectra (aliphatic region) of the reaction **II$_{Na}$** + **SM** → **2** in toluene-d_8, recorded at increasing temperatures. Black labels identify the methyl and isopropyl resonances of the ligand sodium salt **II$_{Na}$** (labels **1** and **2**) and the tridentate complex **2** (labels **3** and **5**), and the methylene resonance of complex **2** (label **4**).

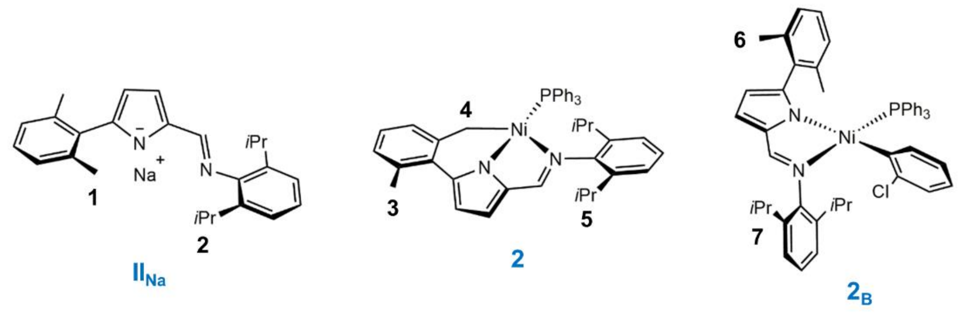

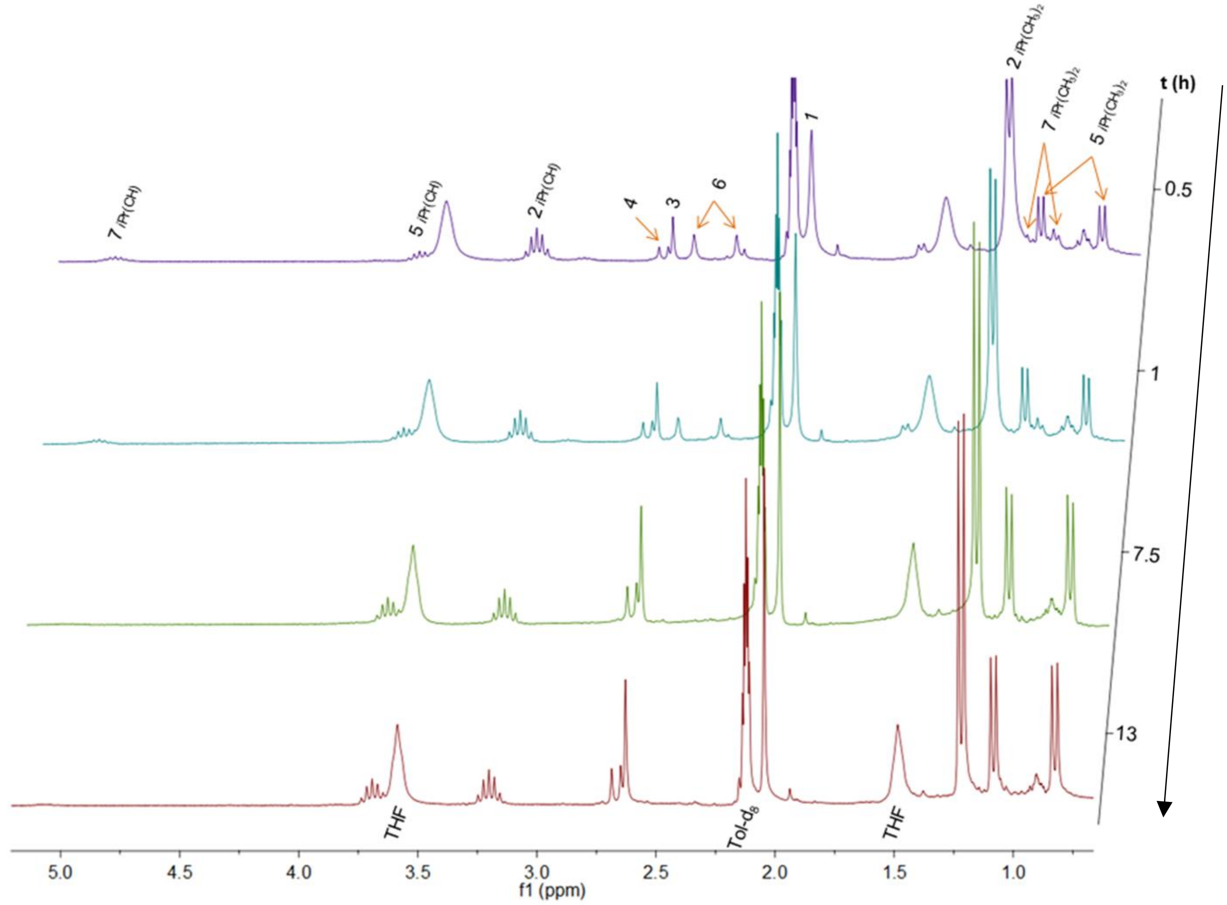

Figure S14 ^{1}H NMR spectra (aliphatic region) showing the time evolution of the reaction **II$_{Na}$** + **SM** → **2** in toluene-d_8 at 25 ºC. Four spectra, recorded at different reaction times over the 13 h reaction period, are displayed. Black labels identify the Me and *i*Pr resonances corresponding to the sodium salt **II$_{Na}$**, the tridentate complex **2**, and the transient bidentate intermediate **2$_B$**, the latter being non-isolable and observable only during the first hour of reaction).

VT-$^{31}P\{^1H\}$ NMR Spectra of the Formation of Complex 2 in the Presence of Free PPh_3 (2 equiv): In an NMR tube-scale reaction conducted under conditions analogous to those in Figure 2 of the main text (see Experimental Section), PPh_3 (2 equiv) were also added at -40 ºC, followed by a gradual increase in temperature. Reaction monitoring by both ^1H and $^{31}P\{^1H\}$ NMR clearly revealed that free PPh_3 markedly retards the formation of complex **2**. For clarity, only the $^{31}P\{^1H\}$ NMR data are shown (Figure S15). In the presence of added PPh_3, the tridentate complex **2** becomes detectable only at *ca.* 50 ºC, whereas in its absence it is already observed at 10-20 ºC (see Figure 2 for comparison). The progressive broadening of the free PPh_3 resonance above 40 ºC is attributed to dynamic exchange between free and coordinated PPh_3 in both the starting material (*trans*-[Ni(*o*-C_6H_4Cl)(PPh_3)$_2$Cl], **SM**) and complex **2**. Notably, the bidentate intermediate **2_B** is not detected under these conditions.

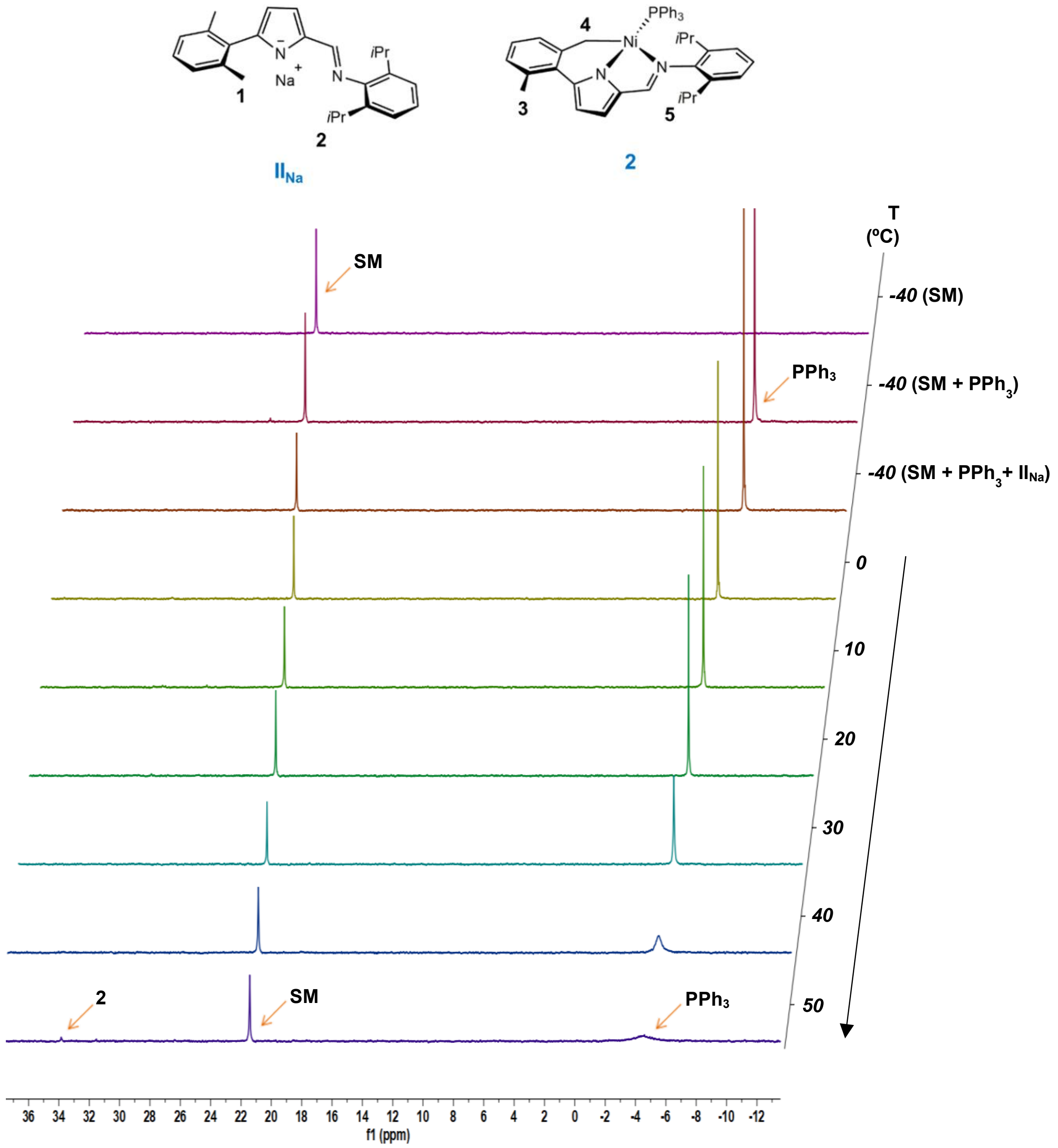

Figure S15 VT-$^{31}P\{^1H\}$ NMR spectra of the reaction **SM** + 2 **PPh$_3$** + **II$_{Na}$** → **2**, recorded at increasing temperatures. The three distinct resonances are labeled as: **SM** (starting material *trans*-[Ni(*o*-C_6H_4Cl)(PPh_3)$_2$Cl]), **PPh$_3$** (free triphenylphosphine) and **2** (tridentate complex).

Computational Calculations

Computed Alternate C-H Activation Pathways: The most kinetically favoured pathways leading to the C-H activation of the studied 2-iminopyrrolyl ligand are discussed in the main text. Additionally, we have computed C-H activation steps leading to the protonation of the *ortho*-chlorophenyl ligand from the bidentate complexes **E** and **E'**, as shown in Figure S16.

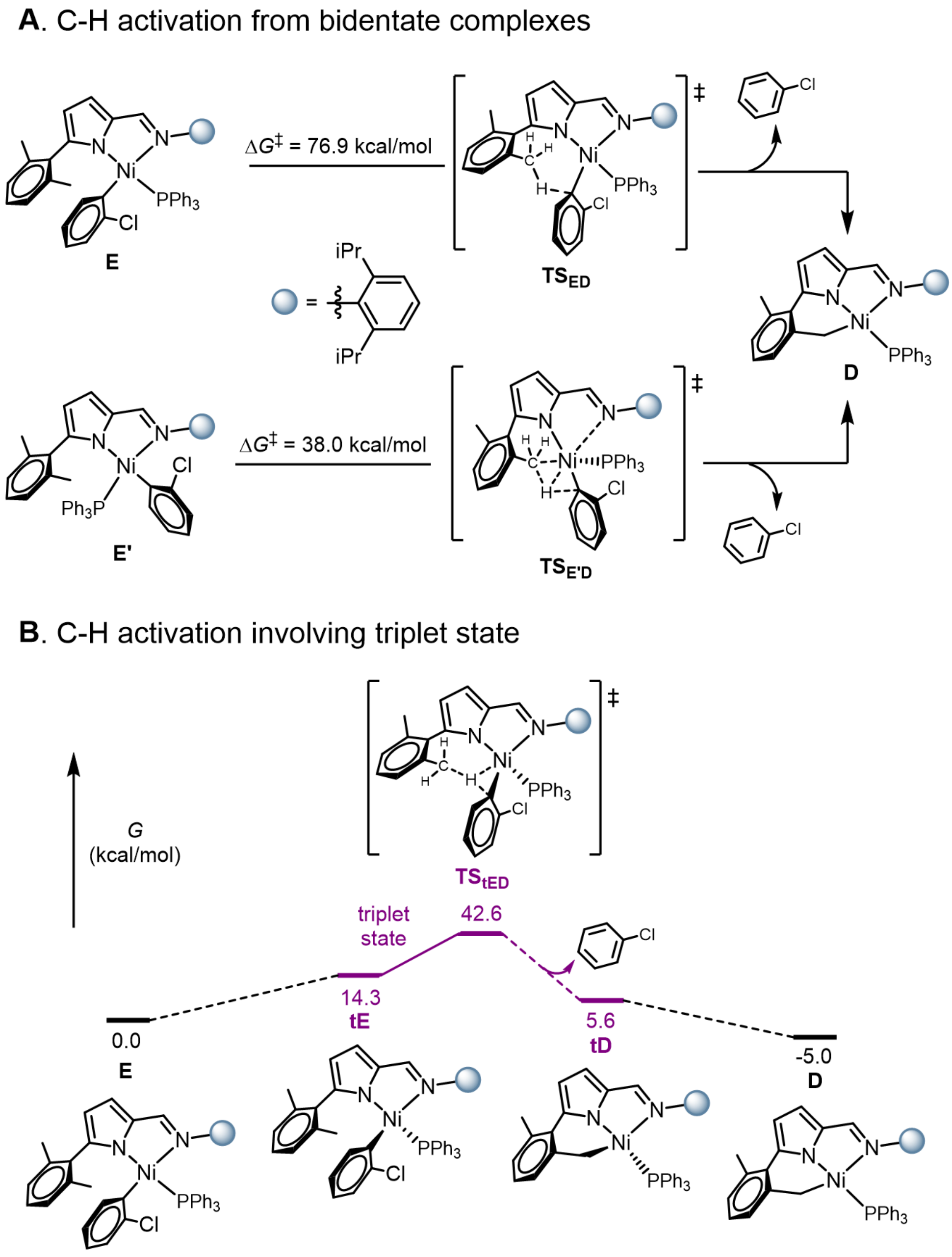

Figure S16 Computed pathways for the C-H activation of bidentate Ni complexes. A) Pathways in singlet state; B) Energy profile for a triplet-state C-H activation. Relative Gibbs free energies are presented in kcal/mol at the ωB97X-D,SMD/def2-TZVP//ωB97X-D,SMD/def2-SVP level of theory.

From **E**, in which the pyrrolyl and the *ortho*-chlorophenyl ligands present *cis* configuration, a proton transfer to the chlorophenyl ligand results in an activation barrier of 76.9 kcal, which is impedingly high for this step to occur in the reaction media (Figure S15A, top route). From the *trans*-configured bidentate complex **E'**, the proton transfer to the chlorophenyl involves a transition state, $\mathbf{TS_{E'D}}$, in which the metal center presents a trigonal bipyramidal geometry (Figure S15A, bottom route). In this structure, an elongation of the Ni-N_{imino} allows the formation of an agostic C-H interaction with the metal center which, together with the imine and the phosphine, occupies the equatorial coordination positions of the trigonal bipyramidal geometry. The formed agostic interaction allows an approximation of the hydrogen to the metal center, enabling the Ni-mediated σ-bond metathesis that forms the tridentate complex **D**, with the release of chlorobenzene. Although this process is mediated by the metal center, the obtained activation energy ($\Delta G^{\ddagger}$(**E'**→**D**) = 38.0 kcal/mol) is still too high to be competitive with the σ-bond metathesis presented in Scheme 3 of the main text. These results indicate that the C-H activation is much less kinetically favorable if the metal is coordinated by the imine.

A possible pathway involving a triplet state Ni complex was also considered, as shown in Figure S15B. The conversion of the singlet-state square planar Ni complex **E** into a triplet-state tetrahedral complex **tE** is thermodynamically disfavored (ΔG(**E**→**tE**) = 14.3 kcal/mol). From this triplet-state structure, a σ-bond metathesis step, followed by the exclusion of chlorobenzene from the computational model leads to the formation of the triplet-state tridentate complex **tD**. The conversion of this intermediate into the final singlet-state tridentate complex **D** is exergonic (ΔG(**E'**→**D**) = -10.6 kcal/mol). The apparent activation barrier of the process is 42.6 kcal/mol, determined between the relative Gibbs free energy of **E** and $\mathbf{TS_{tED}}$, is impedingly high for a spin-crossing mechanism to be kinetically favorable. Hence, the obtained results strongly suggest that the C-H activation should occur from a monodentate complex, which allows the formation of a stable agostic C-H interaction with the metal that in turn enables a facile C-H bond cleavage in a σ-bond metathesis step. However, the C-H activation from a bidentate complex could still be enabled at very high reaction temperatures.

It should be taken into consideration that our NMR studies (Figure S8) indicate that only the bidentate complex $\mathbf{2_B}$ (computed as **E**) is detected by NMR at low temperatures and/or relatively short reaction times, while its *trans*-isomer **E'** is not. This is likely the result of the lower reactivity of **E** towards C-H activation compared to **E'**, as shown in Figure S15A.

Atomic Coordinates: Cartesian coordinates of the most stable (ΔG_{298}) conformations, computed at the PBE0-D3BJ/b2,SMD//PBE0-D3BJ/b1 and ωB97X-D,SMD/def2-TZVP//ωB97X-D,SMD/def2-SVP level of theory:

136

A

Ni	1.565588000	2.910318000	0.979172000
P	2.048106000	4.693765000	-0.387488000
N	-0.342917000	3.327078000	1.229223000
N	-0.439126000	5.442364000	4.210747000
C	-0.854902000	4.076189000	2.277033000
C	-2.252440000	4.058221000	2.251571000
H	-2.892394000	4.568844000	2.958049000
C	-2.611498000	3.280983000	1.153123000
H	-3.608151000	3.055273000	0.795113000
C	-1.422747000	2.854144000	0.534217000
C	-0.010240000	4.796979000	3.184770000
H	1.060408000	4.788621000	2.926804000
C	-1.403709000	2.082679000	-0.743018000
C	-1.683462000	2.739007000	-1.961126000
C	-1.685021000	2.004705000	-3.154044000
H	-1.890734000	2.525078000	-4.087223000
C	-1.466428000	0.636640000	-3.157721000
H	-1.476788000	0.079838000	-4.090904000
C	-1.275302000	-0.023060000	-1.948946000
H	-1.148318000	-1.102648000	-1.932394000
C	-1.251760000	0.678144000	-0.743899000
C	-2.043628000	4.197814000	-2.044447000
H	-2.166679000	4.645313000	-1.059691000
H	-2.984957000	4.314924000	-2.592782000
H	-1.285163000	4.775724000	-2.583160000
C	-1.090094000	-0.085741000	0.535753000
H	-1.897186000	0.143512000	1.238345000
H	-0.145289000	0.161109000	1.026762000
C	0.464593000	6.167655000	5.004357000
C	0.963561000	7.419538000	4.572613000
C	1.832434000	8.113668000	5.417156000
H	2.232885000	9.070726000	5.097627000
C	2.193251000	7.607773000	6.659965000
H	2.873802000	8.163596000	7.299295000
C	1.658130000	6.398997000	7.091808000
H	1.923845000	6.020304000	8.074247000
C	0.781773000	5.666983000	6.288574000
C	0.484246000	8.023862000	3.262782000
H	0.547732000	7.248702000	2.490791000
C	0.132261000	4.382947000	6.775665000
H	0.122999000	3.675648000	5.936930000
C	0.801751000	5.918176000	-0.986981000
C	-0.218783000	6.340617000	-0.129700000
H	-0.358393000	5.860168000	0.831627000
C	-1.091598000	7.354885000	-0.513763000
H	-1.883763000	7.657474000	0.164867000
C	-0.962135000	7.959209000	-1.760467000
H	-1.647167000	8.746982000	-2.060912000
C	0.048258000	7.543182000	-2.623147000
H	0.157745000	8.005254000	-3.600381000
C	0.926249000	6.532600000	-2.240629000
H	1.705109000	6.219264000	-2.928032000
C	2.892286000	4.228160000	-1.947257000
C	2.124520000	3.624981000	-2.952428000
H	1.062303000	3.451914000	-2.800562000
C	2.717950000	3.230729000	-4.146538000
H	2.110765000	2.762464000	-4.915699000
C	4.082654000	3.423833000	-4.347534000
H	4.545159000	3.113708000	-5.280404000
C	4.852627000	4.008931000	-3.346888000
H	5.919569000	4.152451000	-3.491787000
C	4.262874000	4.409513000	-2.150914000
H	4.874845000	4.851085000	-1.371573000
C	3.201231000	5.853655000	0.458503000
C	3.490200000	5.692260000	1.814218000
H	3.075327000	4.847101000	2.354748000
C	4.312667000	6.595836000	2.483126000
H	4.512938000	6.454765000	3.540902000
C	4.863561000	7.671497000	1.794833000
H	5.509213000	8.376425000	2.310921000
C	4.573801000	7.850404000	0.442582000
H	4.990281000	8.696030000	-0.097241000
C	3.740427000	6.955725000	-0.218724000
H	3.505335000	7.119337000	-1.266318000
H	-1.087942000	-1.163154000	0.349968000
C	3.341380000	2.351943000	0.489801000
C	4.589142000	2.728514000	1.011710000
C	3.395582000	1.427813000	-0.563485000
C	5.792218000	2.234833000	0.504196000
C	4.575739000	0.928241000	-1.103171000
C	5.789677000	1.339983000	-0.561850000
H	4.636304000	3.418310000	1.847336000
Cl	1.886889000	0.833277000	-1.210087000
H	6.731550000	2.553477000	0.948464000
H	4.536040000	0.220463000	-1.925087000
H	6.722171000	0.958306000	-0.968066000
C	3.212309000	2.034482000	3.890310000
C	3.010237000	3.261137000	4.537187000
C	4.394505000	1.334374000	4.145433000
C	3.974449000	3.791115000	5.389211000
H	2.078746000	3.802386000	4.408755000
C	5.360246000	1.864488000	4.999502000
H	4.566999000	0.365472000	3.691396000
C	5.160111000	3.095390000	5.615718000
H	3.778382000	4.739283000	5.883065000
H	6.271946000	1.303281000	5.184586000
H	5.915979000	3.503733000	6.280537000
C	2.310246000	-0.317891000	2.399516000
C	3.517937000	-0.644121000	1.765239000
C	1.421601000	-1.355136000	2.713513000
C	3.824503000	-1.967724000	1.463652000
H	4.229795000	0.129410000	1.502983000
C	1.728758000	-2.675762000	2.400362000
H	0.481145000	-1.135049000	3.207065000
C	2.931746000	-2.988130000	1.775905000
H	4.767924000	-2.194093000	0.975162000
H	1.021960000	-3.461331000	2.651903000
H	3.172535000	-4.019632000	1.534865000
C	0.534545000	1.170264000	4.048846000
C	-0.816150000	1.331676000	3.732173000
C	0.884471000	0.716681000	5.330167000
C	-1.798835000	1.052164000	4.681181000
H	-1.110651000	1.676920000	2.749894000
C	-0.098824000	0.429438000	6.269082000
H	1.929663000	0.584208000	5.592859000
C	-1.445000000	0.601006000	5.947246000
H	-2.842391000	1.200528000	4.420265000
H	0.187386000	0.074525000	7.255150000
H	-2.212959000	0.384021000	6.684570000
P	1.856990000	1.430320000	2.788527000
C	-0.989993000	8.429089000	3.384821000
H	-1.356971000	8.837300000	2.436172000
H	-1.610520000	7.571590000	3.659956000
H	-1.110032000	9.200496000	4.153719000
C	1.318478000	9.201816000	2.768758000
H	2.378515000	8.943614000	2.685223000
H	0.969247000	9.507009000	1.777124000
H	1.224384000	10.071839000	3.429088000
C	-1.331470000	4.644132000	7.153295000
H	-1.875845000	5.069381000	6.306684000
H	-1.823613000	3.709600000	7.445421000
H	-1.392499000	5.342008000	7.996188000
C	0.870831000	3.712247000	7.930535000
H	0.403183000	2.748737000	8.154873000
H	1.923012000	3.526922000	7.689612000
H	0.829464000	4.313304000	8.846324000

102

B

Ni	1.624263000	3.415071000	-0.013456000
P	2.230094000	5.413991000	-0.576827000
N	-0.206048000	3.609160000	0.425112000
N	-0.503979000	5.518049000	3.498243000
C	-0.820527000	4.231726000	1.484512000
C	-2.206900000	4.145405000	1.332483000
H	-2.937136000	4.534426000	2.028613000
C	-2.438969000	3.460599000	0.133556000
H	-3.398406000	3.186724000	-0.285066000
C	-1.183205000	3.139507000	-0.402816000
C	-0.025856000	4.873716000	2.497361000
H	1.063008000	4.781306000	2.353351000
C	-0.793863000	2.395736000	-1.611212000
C	-1.335598000	2.694223000	-2.881279000
C	-0.843074000	2.024727000	-4.004220000
H	-1.255705000	2.266523000	-4.980984000
C	0.159419000	1.067684000	-3.895937000
H	0.529761000	0.559875000	-4.781991000
C	0.666160000	0.747194000	-2.642227000
H	1.422763000	-0.027123000	-2.542594000
C	0.196480000	1.389906000	-1.495490000
C	-2.420011000	3.719882000	-3.061100000
H	-2.480952000	4.038985000	-4.105106000
H	-2.253615000	4.599766000	-2.433696000
H	-3.400840000	3.316884000	-2.784139000
C	0.692172000	0.926130000	-0.147136000
H	1.097205000	-0.087649000	-0.224657000
H	-0.097359000	0.949814000	0.606376000
C	0.405542000	6.097751000	4.402366000
C	0.584733000	7.498634000	4.368151000
C	1.464370000	8.079207000	5.283188000
H	1.623356000	9.153245000	5.263776000
C	2.138989000	7.307045000	6.222654000
H	2.818093000	7.776431000	6.929193000
C	1.929891000	5.933969000	6.263136000
H	2.452606000	5.340012000	7.006343000
C	1.066736000	5.302680000	5.365198000
C	-0.202984000	8.332484000	3.371826000
H	-0.211694000	7.788383000	2.418550000
C	0.776059000	3.812976000	5.456692000
H	0.768130000	3.408013000	4.437620000
C	1.009102000	6.790839000	-0.544718000
C	-0.356288000	6.544070000	-0.713099000
H	-0.725639000	5.525922000	-0.757405000
C	-1.252240000	7.606184000	-0.812253000
H	-2.311831000	7.397179000	-0.925353000
C	-0.796548000	8.918819000	-0.755753000
H	-1.498589000	9.744440000	-0.830773000
C	0.565098000	9.171917000	-0.601534000
H	0.930467000	10.193791000	-0.557669000
C	1.463725000	8.116580000	-0.497092000
H	2.521106000	8.328162000	-0.371622000
C	2.741331000	5.472413000	-2.333732000
C	2.071884000	4.636919000	-3.234962000
H	1.338222000	3.918668000	-2.873664000
C	2.342746000	4.717452000	-4.597531000
H	1.816891000	4.060963000	-5.284461000
C	3.284269000	5.628173000	-5.069858000
H	3.499905000	5.687108000	-6.132849000
C	3.946848000	6.467092000	-4.177263000
H	4.677956000	7.182935000	-4.541900000
C	3.672811000	6.397441000	-2.814522000
H	4.188075000	7.062437000	-2.127796000
C	3.610057000	6.020413000	0.452330000
C	4.943505000	5.737852000	0.133633000
H	5.185975000	5.200164000	-0.777967000
C	5.962514000	6.125173000	0.998309000
H	6.994392000	5.906313000	0.739553000
C	5.662389000	6.772561000	2.194455000
H	6.461686000	7.062665000	2.870440000
C	4.336850000	7.035512000	2.528899000
H	4.083627000	7.515476000	3.469740000
C	3.315052000	6.664030000	1.661503000
H	2.286560000	6.875466000	1.938105000
H	1.553394000	1.492203000	0.277144000
C	3.420745000	2.859367000	-0.264852000
C	4.153209000	2.591087000	0.895657000
C	4.039115000	2.504020000	-1.472392000
C	5.423987000	2.026871000	0.894218000
C	5.311083000	1.932279000	-1.509307000
C	6.008085000	1.701763000	-0.326567000
Cl	3.419688000	2.956900000	2.450346000
H	3.517425000	2.678427000	-2.409003000
H	5.940080000	1.844371000	1.831552000
H	5.757311000	1.669394000	-2.464670000
H	7.001763000	1.263352000	-0.347826000
C	-0.624072000	3.588467000	6.041005000
H	-1.382561000	4.096487000	5.438810000
H	-0.865218000	2.520026000	6.070729000
H	-0.677955000	3.979266000	7.063330000
C	1.819880000	3.021779000	6.240120000
H	1.815208000	3.283316000	7.304470000
H	1.602225000	1.951269000	6.170800000
H	2.831282000	3.186388000	5.853936000
C	0.397625000	9.710295000	3.105484000
H	0.338821000	10.359008000	3.987028000
H	1.448003000	9.646263000	2.800872000
H	-0.155278000	10.205814000	2.301380000
C	-1.662428000	8.460474000	3.826076000
H	-2.249928000	9.014678000	3.085234000
H	-2.111876000	7.472782000	3.954537000
H	-1.721062000	8.997897000	4.779343000

102

TSBC

Ni	1.569586000	3.250961000	0.097192000
P	2.260788000	5.357794000	-0.483291000
N	-0.228995000	3.788823000	0.275490000
N	-0.585309000	5.665172000	3.361879000
C	-0.851564000	4.471390000	1.288090000
C	-2.236052000	4.443816000	1.081873000
H	-2.970765000	4.912141000	1.722123000
C	-2.453177000	3.706141000	-0.082887000
H	-3.409111000	3.452762000	-0.519978000
C	-1.191440000	3.286401000	-0.546397000
C	-0.082050000	5.054049000	2.352982000
H	1.008496000	4.916033000	2.262469000
C	-0.814295000	2.374500000	-1.631401000
C	-1.440679000	2.406273000	-2.896582000
C	-0.982385000	1.559211000	-3.909077000
H	-1.461073000	1.604158000	-4.884468000
C	0.070126000	0.676858000	-3.696982000
H	0.409835000	0.025443000	-4.497218000
C	0.673919000	0.624424000	-2.446917000
H	1.478604000	-0.083520000	-2.261359000
C	0.243631000	1.450826000	-1.403707000
C	-2.573586000	3.346206000	-3.200680000
H	-2.679291000	3.486135000	-4.279971000
H	-2.424987000	4.322604000	-2.730914000
H	-3.528307000	2.957230000	-2.827702000
C	0.913710000	1.331999000	-0.071924000
H	1.482577000	0.396861000	-0.011379000
H	0.239299000	1.376667000	0.785344000
C	0.286716000	6.137773000	4.358918000
C	0.489460000	7.531509000	4.470579000
C	1.330393000	8.002904000	5.479077000
H	1.511388000	9.069662000	5.569323000
C	1.942065000	7.131177000	6.372446000
H	2.593861000	7.516420000	7.151843000
C	1.704881000	5.765888000	6.273936000
H	2.175489000	5.094664000	6.985594000
C	0.878156000	5.240612000	5.278525000
C	-0.233239000	8.471367000	3.521511000
H	-0.181826000	8.029011000	2.518906000
C	0.546674000	3.757026000	5.225634000
H	0.606211000	3.433160000	4.179420000
C	1.175635000	6.844019000	-0.374860000
C	-0.160759000	6.733381000	-0.780199000
H	-0.571969000	5.766680000	-1.051472000
C	-0.971623000	7.861877000	-0.835590000
H	-2.009007000	7.756185000	-1.138590000
C	-0.461619000	9.113105000	-0.499913000
H	-1.098259000	9.992254000	-0.544454000
C	0.868177000	9.232402000	-0.107841000
H	1.276640000	10.204008000	0.154378000
C	1.683760000	8.105555000	-0.043262000
H	2.718052000	8.215807000	0.265130000
C	2.573494000	5.365253000	-2.293326000
C	2.005938000	4.365604000	-3.089053000
H	1.444207000	3.554866000	-2.630499000
C	2.139995000	4.406145000	-4.475328000
H	1.686297000	3.623240000	-5.075901000
C	2.844952000	5.443098000	-5.077542000

H	2.953024000	5.474021000	-6.158047000
C	3.401154000	6.451693000	-4.292238000
H	3.939116000	7.272051000	-4.758734000
C	3.257126000	6.420939000	-2.909719000
H	3.670135000	7.226294000	-2.308816000
C	3.811348000	5.886798000	0.331509000
C	5.061749000	5.792594000	-0.286550000
H	5.137998000	5.439432000	-1.309569000
C	6.217051000	6.135581000	0.411569000
H	7.182061000	6.058672000	-0.081277000
C	6.138337000	6.568268000	1.731749000
H	7.041510000	6.836517000	2.272502000
C	4.897694000	6.651263000	2.359978000
H	4.821285000	6.978068000	3.392842000
C	3.741802000	6.308675000	1.667653000
H	2.783282000	6.380491000	2.174766000
H	2.259752000	1.895375000	0.005013000
C	3.432006000	2.694014000	-0.087420000
C	4.204047000	2.668162000	1.084150000
C	4.113398000	2.458724000	-1.292783000
C	5.581565000	2.491808000	1.076512000
C	5.493633000	2.277903000	-1.324808000
C	6.227523000	2.301515000	-0.141800000
Cl	3.392179000	2.858112000	2.617110000
H	3.548593000	2.415547000	-2.219102000
H	6.134011000	2.499978000	2.010198000
H	5.993296000	2.107482000	-2.274063000
H	7.303878000	2.157436000	-0.160262000
C	-0.897642000	3.526451000	5.686915000
H	-1.597791000	4.101565000	5.074647000
H	-1.164108000	2.466116000	5.613388000
H	-1.017982000	3.837438000	6.730701000
C	1.508266000	2.875029000	6.018002000
H	1.422750000	3.046946000	7.097054000
H	1.275361000	1.820213000	5.840688000
H	2.551632000	3.043573000	5.730725000
C	0.385061000	9.863975000	3.440312000
H	0.268598000	10.416678000	4.379696000
H	1.452880000	9.823511000	3.200111000
H	-0.114884000	10.446059000	2.659546000
C	-1.717449000	8.566661000	3.897746000
H	-2.255873000	9.200963000	3.184213000
H	-2.178478000	7.576094000	3.894592000
H	-1.833313000	9.002967000	4.896373000

102
C+PhCl

Ni	1.512491000	3.074835000	0.056255000
P	2.306664000	5.287140000	-0.605510000
N	-0.276315000	3.649950000	0.274896000
N	-0.303314000	5.442080000	3.432238000
C	-0.791282000	4.337739000	1.342436000
C	-2.177607000	4.469905000	1.179591000
H	-2.839524000	4.979992000	1.865826000
C	-2.501931000	3.838470000	-0.020567000
H	-3.489370000	3.718963000	-0.444221000
C	-1.304606000	3.318204000	-0.552048000
C	0.077410000	4.805704000	2.384121000
H	1.143394000	4.563700000	2.229666000
C	-1.022200000	2.478061000	-1.713110000
C	-1.675788000	2.596842000	-2.954802000
C	-1.248226000	1.798693000	-4.019142000
H	-1.739881000	1.905603000	-4.983118000
C	-0.203033000	0.891328000	-3.875855000
H	0.109048000	0.282464000	-4.720025000
C	0.427025000	0.757030000	-2.644881000
H	1.219478000	0.023103000	-2.513068000
C	0.019156000	1.526652000	-1.548612000
C	-2.799397000	3.570238000	-3.173688000
H	-2.951851000	3.752593000	-4.240881000
H	-2.604910000	4.527141000	-2.679848000
H	-3.744705000	3.190028000	-2.768296000
C	0.648475000	1.361111000	-0.221720000
H	1.357393000	0.525069000	-0.214235000
H	-0.081671000	1.236903000	0.582912000
C	0.679381000	5.814682000	4.366141000
C	1.109537000	7.160273000	4.398036000
C	2.070795000	7.528647000	5.341082000
H	2.421616000	8.555862000	5.372649000
C	2.584013000	6.605865000	6.245706000
H	3.329824000	6.911756000	6.974262000
C	2.124839000	5.294395000	6.226741000
H	2.517736000	4.585960000	6.949703000
C	1.169597000	4.872295000	5.299567000
C	0.491037000	8.167766000	3.444213000
H	0.402314000	7.683707000	2.463539000
C	0.601932000	3.461442000	5.325543000
H	0.558014000	3.097493000	4.290554000
C	1.148341000	6.725978000	-0.631548000
C	-0.182264000	6.516878000	-1.013242000
H	-0.540351000	5.514331000	-1.220403000
C	-1.061762000	7.589316000	-1.118463000
H	-2.094185000	7.402651000	-1.399254000
C	-0.627971000	8.885343000	-0.855443000
H	-1.317830000	9.720604000	-0.936795000
C	0.696092000	9.104392000	-0.487667000
H	1.049259000	10.111845000	-0.286081000
C	1.579245000	8.033498000	-0.373310000
H	2.606226000	8.224417000	-0.079636000
C	2.707196000	5.158079000	-2.394027000
C	2.529525000	3.935858000	-3.046417000
H	2.178388000	3.068421000	-2.492775000
C	2.763959000	3.823087000	-4.415288000
H	2.604070000	2.868477000	-4.907458000
C	3.185032000	4.931076000	-5.141647000
H	3.368837000	4.845412000	-6.208937000
C	3.356011000	6.158741000	-4.501715000
H	3.669971000	7.030233000	-5.069234000
C	3.108403000	6.275274000	-3.139410000
H	3.213223000	7.241075000	-2.652382000
C	3.790887000	6.037797000	0.174263000
C	5.015466000	6.199076000	-0.478968000
H	5.126093000	5.887887000	-1.513088000
C	6.105750000	6.751039000	0.193923000
H	7.050748000	6.872163000	-0.328617000
C	5.986648000	7.146976000	1.522770000
H	6.837023000	7.580355000	2.041672000
C	4.768742000	6.987714000	2.183597000
H	4.655290000	7.288510000	3.221601000
C	3.682395000	6.433505000	1.515791000
H	2.742369000	6.317350000	2.049757000
H	3.208828000	1.298995000	-0.633588000
C	3.549993000	1.971512000	0.146340000
C	4.775770000	2.644061000	-0.031603000
C	2.952281000	1.984488000	1.421710000
C	5.375708000	3.332561000	1.003532000
C	3.536611000	2.732435000	2.460777000
C	4.733548000	3.389848000	2.248280000
Cl	5.569412000	2.543996000	-1.570045000
H	2.140922000	1.305402000	1.655436000
H	6.322690000	3.835544000	0.847842000
H	3.065411000	2.753458000	3.438082000
H	5.197060000	3.950654000	3.053606000
C	-0.839075000	3.483099000	5.850686000
H	-1.463770000	4.138656000	5.238460000
H	-1.273147000	2.477227000	5.834540000
H	-0.861674000	3.849069000	6.883093000
C	1.440503000	2.464817000	6.122191000
H	1.433273000	2.695269000	7.193419000
H	1.029227000	1.456887000	6.007445000
H	2.486167000	2.442494000	5.794768000
C	1.321297000	9.433780000	3.253347000
H	1.365214000	10.035989000	4.168179000
H	2.347860000	9.207578000	2.944814000
H	0.867092000	10.057757000	2.477447000
C	-0.931093000	8.520748000	3.898432000
H	-1.403636000	9.204118000	3.184000000
H	-1.546036000	7.620421000	3.971346000
H	-0.911391000	9.010543000	4.878645000

90
C

Ni	1.312486000	4.475587000	-0.084083000
P	3.050850000	5.654391000	-0.696387000
N	-0.180666000	3.667291000	0.663518000
N	0.354026000	4.568512000	4.128623000
C	-0.382566000	3.623801000	2.021792000
C	-1.368228000	2.675002000	2.314933000
H	-1.748806000	2.446887000	3.301217000
C	-1.772500000	2.130983000	1.094581000
H	-2.509612000	1.353951000	0.952258000
C	-1.009805000	2.750786000	0.086254000
C	0.349167000	4.538963000	2.847432000
H	0.955492000	5.271232000	2.272577000
C	-0.949062000	2.554904000	-1.358156000
C	-2.083172000	2.238403000	-2.139377000
C	-1.954554000	2.184648000	-3.527498000
H	-2.835947000	1.962175000	-4.124345000
C	-0.739824000	2.433845000	-4.160116000
H	-0.669547000	2.391087000	-5.243505000
C	0.379294000	2.727023000	-3.396359000
H	1.340149000	2.899297000	-3.875281000
C	0.302348000	2.765388000	-1.997539000
C	-3.435753000	1.998598000	-1.529737000
H	-4.218760000	2.081186000	-2.288429000
H	-3.647646000	2.712476000	-0.728273000
H	-3.510606000	0.995598000	-1.093084000
C	1.538501000	2.964102000	-1.211687000
H	2.417730000	3.045725000	-1.857037000
H	1.685810000	2.186072000	-0.452095000
C	1.105471000	5.567576000	4.770710000
C	2.338187000	5.212120000	5.356715000
C	3.071686000	6.194305000	6.023967000
H	4.026915000	5.935558000	6.472253000
C	2.595156000	7.494957000	6.132020000
H	3.174088000	8.247477000	6.660480000
C	1.362489000	7.825151000	5.576834000
H	0.988244000	8.839028000	5.683669000
C	0.595541000	6.881070000	4.891225000
C	2.843050000	3.788147000	5.223876000
H	1.951918000	3.156704000	5.116674000
C	-0.783981000	7.216378000	4.349029000
H	-0.876936000	6.751539000	3.360021000
C	2.889942000	7.326441000	0.034179000
C	2.908924000	7.438397000	1.432067000
H	3.088895000	6.563421000	2.052547000
C	2.709760000	8.672741000	2.041048000
H	2.718457000	8.738119000	3.125240000
C	2.486923000	9.805082000	1.259363000
H	2.328228000	10.769343000	1.733685000
C	2.466251000	9.700337000	-0.128761000
H	2.295168000	10.582102000	-0.739617000
C	2.664426000	8.466289000	-0.743359000
H	2.646379000	8.391189000	-1.826617000
C	3.233942000	5.919093000	-2.494095000
C	2.072554000	5.894870000	-3.276061000
H	1.112879000	5.680443000	-2.811837000
C	2.146699000	6.120849000	-4.647645000
H	1.240436000	6.095788000	-5.245465000
C	3.379810000	6.359719000	-5.248644000
H	3.438588000	6.528115000	-6.320156000
C	4.540058000	6.371451000	-4.477525000
H	5.503987000	6.547873000	-4.946001000
C	4.470390000	6.152977000	-3.105036000
H	5.379720000	6.153133000	-2.511283000
C	4.679881000	5.070823000	-0.103601000
C	4.870020000	3.702489000	0.118703000
H	4.048305000	3.010376000	-0.039631000
C	6.102829000	3.228429000	0.557999000
H	6.239378000	2.165389000	0.732970000
C	7.151214000	4.115714000	0.785210000
H	8.110537000	3.745269000	1.134841000
C	6.966047000	5.480754000	0.576669000
H	7.778777000	6.176425000	0.764431000
C	5.735865000	5.958953000	0.136629000
H	5.588175000	7.026323000	-0.003800000
C	-1.033812000	8.710596000	4.163862000
H	-0.261906000	9.177070000	3.542415000
H	-2.000526000	8.867075000	3.674806000
H	-1.068539000	9.241094000	5.122296000
C	-1.862737000	6.590883000	5.241869000
H	-1.816685000	7.014674000	6.251337000
H	-2.861929000	6.783177000	4.835575000
H	-1.723188000	5.508910000	5.316782000
C	3.621313000	3.303217000	6.446439000
H	4.584131000	3.816312000	6.550910000
H	3.054476000	3.458419000	7.369371000
H	3.836695000	2.233477000	6.355061000
C	3.681037000	3.615770000	3.951444000
H	4.022898000	2.579515000	3.847085000
H	3.103682000	3.867672000	3.056121000
H	4.566191000	4.262288000	3.975403000

90
TSCD

Ni	1.593663000	3.699998000	0.300435000
P	2.831522000	5.518621000	-0.498547000
N	-0.116643000	4.238616000	0.686745000
N	0.828575000	4.939736000	3.630250000
C	-0.527345000	5.082913000	1.666298000
C	-1.893984000	5.309585000	1.530797000
H	-2.499233000	5.950486000	2.158415000
C	-2.323025000	4.541712000	0.432089000
H	-3.341568000	4.438368000	0.086605000
C	-1.205628000	3.863973000	-0.067111000
C	0.451724000	5.626857000	2.626163000
H	0.873979000	6.626559000	2.427887000
C	-1.014360000	2.862028000	-1.112320000
C	-1.790392000	2.796972000	-2.287956000
C	-1.447582000	1.871592000	-3.277312000
H	-2.040612000	1.839837000	-4.188382000
C	-0.360686000	1.016027000	-3.133495000
H	-0.112796000	0.309785000	-3.921133000
C	0.395576000	1.062154000	-1.968383000
H	1.226214000	0.374120000	-1.826384000
C	0.070995000	1.954465000	-0.940311000
C	-2.954326000	3.716464000	-2.523157000
H	-3.244603000	3.712781000	-3.577345000
H	-2.717216000	4.743194000	-2.226487000
H	-3.832108000	3.412701000	-1.940137000
C	0.848461000	1.981654000	0.319802000
H	1.727932000	1.315231000	0.303891000
H	0.250125000	1.816276000	1.218893000
C	1.818860000	5.440583000	4.499193000
C	3.020341000	4.700622000	4.580489000
C	4.019326000	5.155999000	5.440880000
H	4.958027000	4.614273000	5.503290000
C	3.826189000	6.283633000	6.231441000
H	4.612900000	6.622201000	6.899927000
C	2.615230000	6.962143000	6.183432000
H	2.466365000	7.823810000	6.826031000
C	1.587347000	6.562168000	5.325814000
C	3.173848000	3.418579000	3.777074000
H	2.771880000	3.609661000	2.769352000
C	0.223537000	7.237507000	5.365502000
H	-0.129994000	7.362268000	4.336497000
C	1.977688000	7.135949000	-0.364693000
C	0.600291000	7.168218000	-0.621589000
H	0.063879000	6.250718000	-0.849670000
C	-0.089101000	8.375367000	-0.564426000
H	-1.158134000	8.386657000	-0.754390000
C	0.582518000	9.553857000	-0.248446000
H	0.039156000	10.493184000	-0.197740000
C	1.950655000	9.525643000	0.008374000
H	2.479243000	10.441823000	0.256040000
C	2.648722000	8.322338000	-0.049305000
H	3.714321000	8.307096000	0.159470000
C	3.078246000	5.335167000	-2.304074000
C	2.676196000	4.150393000	-2.928038000
H	2.210415000	3.355642000	-2.348460000
C	2.830505000	3.991865000	-4.303757000
H	2.501254000	3.071034000	-4.775773000
C	3.386679000	5.015117000	-5.063699000
H	3.504700000	4.893538000	-6.136719000
C	3.779854000	6.204988000	-4.450817000
H	4.202055000	7.010352000	-5.045013000
C	3.621157000	6.368314000	-3.079796000
H	3.909948000	7.304370000	-2.608836000
C	4.498834000	5.818089000	0.197970000
C	5.674156000	5.679167000	-0.546917000
H	5.623006000	5.425797000	-1.601281000
C	6.914941000	5.866827000	0.058689000
H	7.820656000	5.755008000	-0.530566000
C	6.995687000	6.198809000	1.407492000
H	7.964732000	6.347728000	1.875052000
C	5.828968000	6.331434000	2.158923000
H	5.874998000	6.575401000	3.216354000
C	4.589161000	6.131493000	1.562079000
H	3.689961000	6.218343000	2.167708000
C	0.237160000	8.627930000	5.995652000
H	0.973555000	9.284353000	5.520845000
H	-0.748287000	9.092048000	5.888771000
H	0.458491000	8.586655000	7.067902000
C	-0.782332000	6.329621000	6.084365000
H	-0.470854000	6.161806000	7.121145000
H	-1.777577000	6.787417000	6.096760000
H	-0.858867000	5.356423000	5.590930000
C	2.321463000	2.302735000	4.396370000
H	2.679166000	2.067905000	5.405169000
H	1.272807000	2.601242000	4.455947000
H	2.387638000	1.390834000	3.792230000
C	4.617672000	2.949995000	3.616289000
H	4.651329000	2.081036000	2.950819000
H	5.259836000	3.726595000	3.189913000
H	5.048112000	2.634203000	4.573637000

102
TSBE'

Ni	1.774334000	3.467237000	0.177469000
P	2.711456000	5.158126000	-0.745643000
N	-0.031943000	3.968177000	0.210105000
N	0.152553000	5.019477000	3.359997000
C	-0.679640000	4.797593000	1.092592000
C	-2.011618000	4.918832000	0.735564000
H	-2.764676000	5.487319000	1.266343000
C	-2.195576000	4.120686000	-0.417094000
H	-3.125157000	3.933057000	-0.939499000
C	-0.967956000	3.555327000	-0.720444000
C	-0.030651000	5.525451000	2.202722000
H	0.224739000	6.581619000	2.018480000
C	-0.647918000	2.560791000	-1.769935000
C	-0.718601000	2.907299000	-3.138466000
C	-0.426501000	1.942407000	-4.102728000
H	-0.485316000	2.214084000	-5.154153000
C	-0.057933000	0.651040000	-3.740393000
H	0.169270000	-0.087865000	-4.504310000
C	-0.012512000	0.304262000	-2.397793000
H	0.236651000	-0.713865000	-2.107520000
C	-0.323568000	1.236263000	-1.402345000
C	-1.064824000	4.301731000	-3.585359000
H	-1.933894000	4.700327000	-3.058498000
H	-1.268832000	4.322176000	-4.659523000
H	-0.230188000	4.988393000	-3.397761000
C	-0.364858000	0.774015000	0.031228000
H	-0.973944000	1.437487000	0.649206000
H	0.636099000	0.727100000	0.476884000
C	0.604882000	5.880257000	4.389114000
C	-0.244587000	6.895145000	4.889451000
C	0.229187000	7.686068000	5.938601000
H	-0.401360000	8.474051000	6.337336000
C	1.491467000	7.491760000	6.484137000
H	1.840537000	8.129574000	7.291303000
C	2.297273000	6.468127000	6.003563000
H	3.276760000	6.308355000	6.444019000
C	1.867126000	5.639769000	4.967399000
C	-1.670287000	7.054283000	4.377958000
H	-1.649989000	7.018459000	3.282954000
C	2.697448000	4.457758000	4.506212000
H	2.529688000	4.337106000	3.429006000
C	1.866762000	6.741375000	-0.366397000
C	0.548421000	6.925668000	-0.805948000
H	0.032110000	6.132149000	-1.338693000
C	-0.114593000	8.117083000	-0.536268000
H	-1.138106000	8.245374000	-0.875210000
C	0.521514000	9.128961000	0.181587000
H	-0.003254000	10.055320000	0.397386000
C	1.827264000	8.947702000	0.626807000
H	2.327206000	9.729774000	1.190504000
C	2.501131000	7.759345000	0.353153000
H	3.520933000	7.628623000	0.700542000
C	2.653633000	5.080191000	-2.569141000
C	2.556267000	3.842379000	-3.210114000
H	2.472071000	2.932025000	-2.625375000
C	2.552172000	3.776509000	-4.600899000
H	2.464608000	2.809809000	-5.086289000
C	2.636868000	4.940858000	-5.357498000
H	2.629641000	4.886042000	-6.442456000
C	2.714852000	6.180301000	-4.724243000
H	2.765911000	7.092580000	-5.311496000
C	2.718199000	6.253103000	-3.335738000
H	2.759290000	7.221821000	-2.845555000
C	4.451100000	5.482191000	-0.284525000
C	5.363036000	6.033379000	-1.189171000
H	5.065196000	6.225667000	-2.215108000
C	6.663543000	6.317299000	-0.783947000
H	7.368658000	6.735096000	-1.496648000
C	7.061163000	6.060323000	0.525316000
H	8.078080000	6.279447000	0.837992000
C	6.155634000	5.514441000	1.431259000
H	6.460168000	5.303980000	2.452172000
C	4.856594000	5.221037000	1.028183000
H	4.160665000	4.772859000	1.731094000
H	-0.781585000	-0.236680000	0.088568000
C	3.231635000	2.390398000	0.576632000
C	4.375292000	1.794918000	0.045118000
C	2.751723000	1.854434000	1.784083000
C	5.004755000	0.716080000	0.663837000
C	3.352751000	0.765247000	2.415929000
C	4.491368000	0.201427000	1.850456000
Cl	5.067907000	2.349338000	-1.461622000
H	1.866876000	2.300047000	2.255517000
H	5.884774000	0.276871000	0.204693000
H	2.939493000	0.372216000	3.340217000
H	4.983797000	-0.641260000	2.326863000
C	2.194537000	3.182504000	5.193482000
H	1.139337000	3.009650000	4.964164000
H	2.772889000	2.311751000	4.866351000
H	2.298890000	3.270556000	6.280572000
C	4.200080000	4.626613000	4.720173000
H	4.466510000	4.607956000	5.782837000
H	4.737118000	3.800510000	4.241150000
H	4.565873000	5.569039000	4.298470000
C	-2.320979000	8.383017000	4.755252000
H	-2.498536000	8.458560000	5.833814000
H	-1.709525000	9.238487000	4.450116000
H	-3.294540000	8.470043000	4.262670000
C	-2.531999000	5.879272000	4.856194000
H	-3.554410000	5.966723000	4.471932000
H	-2.119365000	4.924550000	4.517970000
H	-2.579436000	5.862907000	5.950574000

102

E'

Ni	1.840707000	3.271897000	1.373889000
P	3.092918000	4.313090000	-0.178353000
N	0.251662000	4.459602000	1.434926000
N	1.453783000	3.060418000	3.324831000
C	0.003407000	4.800734000	2.748367000
C	-1.024081000	5.751894000	2.834895000
H	-1.430970000	6.182776000	3.740880000
C	-1.430650000	5.977783000	1.524810000
H	-2.236706000	6.614310000	1.184113000
C	-0.641987000	5.151515000	0.692625000
C	0.645300000	4.009898000	3.717158000
H	0.431308000	4.147369000	4.778527000
C	-0.926176000	4.896001000	-0.737393000
C	-0.792738000	5.906063000	-1.708310000
C	-1.131379000	5.621958000	-3.034727000
H	-1.007745000	6.397080000	-3.787280000
C	-1.632243000	4.379396000	-3.395708000
H	-1.898813000	4.177487000	-4.429915000
C	-1.818224000	3.402240000	-2.422508000
H	-2.251148000	2.441799000	-2.691954000
C	-1.481177000	3.643269000	-1.089859000
C	-0.349748000	7.297481000	-1.351537000
H	0.198534000	7.757627000	-2.178268000
H	0.282064000	7.314490000	-0.462925000
H	-1.219310000	7.933518000	-1.143544000
C	-1.781388000	2.600442000	-0.047795000
H	-2.377658000	1.790454000	-0.477033000
H	-2.340846000	3.037507000	0.786177000
C	2.056720000	2.247269000	4.322299000
C	1.663250000	0.894314000	4.395802000
C	2.243100000	0.087267000	5.372198000
H	1.951112000	-0.955878000	5.444371000
C	3.184665000	0.596280000	6.259728000
H	3.622631000	-0.046879000	7.018055000
C	3.562513000	1.927756000	6.173972000
H	4.300673000	2.318916000	6.869360000
C	3.019251000	2.778044000	5.206368000
C	0.582831000	0.362101000	3.475257000
H	0.651249000	0.928374000	2.539162000
C	3.479958000	4.226534000	5.150796000
H	3.098520000	4.663726000	4.221371000
C	2.881786000	6.141467000	-0.099976000
C	2.483246000	6.725554000	1.108103000
H	2.190170000	6.096341000	1.943367000
C	2.436328000	8.111401000	1.240374000
H	2.119753000	8.549038000	2.182641000
C	2.776576000	8.928087000	0.165519000
H	2.734433000	10.008867000	0.266822000
C	3.160962000	8.353846000	-1.043579000
H	3.419090000	8.985003000	-1.889345000
C	3.215388000	6.969630000	-1.176874000
H	3.513141000	6.533801000	-2.125444000
C	3.016284000	3.914542000	-1.967119000
C	1.828391000	3.411735000	-2.503217000
H	0.972268000	3.236505000	-1.861804000
C	1.733316000	3.134779000	-3.864695000
H	0.800903000	2.745238000	-4.260693000
C	2.819615000	3.357440000	-4.703667000
H	2.744624000	3.135535000	-5.764604000
C	4.006951000	3.864146000	-4.179399000
H	4.860346000	4.040845000	-4.828039000
C	4.107401000	4.140327000	-2.820259000
H	5.041732000	4.521398000	-2.419554000
C	4.896570000	4.170901000	0.179287000
C	5.611950000	3.052728000	-0.269214000
H	5.107005000	2.275924000	-0.835711000
C	6.966545000	2.921818000	0.013127000
H	7.505315000	2.050172000	-0.346674000
C	7.623329000	3.890886000	0.768376000
H	8.680799000	3.784027000	0.993144000
C	6.917167000	4.994820000	1.234283000
H	7.419287000	5.756612000	1.823943000
C	5.563241000	5.138479000	0.936726000
H	5.030999000	6.015449000	1.291325000
H	-0.872472000	2.165653000	0.375621000
C	3.080771000	1.837073000	1.372360000
C	2.809412000	0.698360000	0.611933000
C	4.231338000	1.762198000	2.169114000
C	3.609282000	-0.443718000	0.610647000
C	5.043506000	0.633194000	2.210481000
C	4.737691000	-0.473515000	1.422616000
Cl	1.360171000	0.655087000	-0.387490000
H	4.503905000	2.622846000	2.771745000
H	3.338482000	-1.292791000	-0.009324000
H	5.920290000	0.622663000	2.852088000
H	5.367743000	-1.358390000	1.439122000
C	-0.805704000	0.627448000	4.070328000
H	-0.924879000	0.102213000	5.024871000
H	-0.970215000	1.694116000	4.249644000
H	-1.588834000	0.276618000	3.389222000
C	0.744976000	-1.116713000	3.129822000
H	0.553110000	-1.764494000	3.993025000
H	0.026251000	-1.395316000	2.352609000
H	1.749909000	-1.329955000	2.754270000
C	5.005854000	4.363663000	5.121598000
H	5.451333000	3.814585000	4.287458000
H	5.284844000	5.417757000	5.016319000
H	5.464065000	3.997200000	6.046313000
C	2.912217000	5.035337000	6.323631000
H	3.268942000	4.634032000	7.278669000
H	3.226995000	6.082968000	6.261502000
H	1.818710000	5.011517000	6.348717000

102

E

Ni	1.757178000	3.450136000	1.285592000
P	3.687533000	2.311618000	0.852926000
N	-0.128530000	3.956434000	1.385224000
N	1.532752000	3.030181000	3.261587000
C	-0.558493000	3.922891000	2.696632000
C	-1.887523000	4.350847000	2.806706000
H	-2.469312000	4.416590000	3.717153000
C	-2.284725000	4.649221000	1.510861000
H	-3.253639000	4.994735000	1.174896000
C	-1.185762000	4.392024000	0.659782000
C	0.345757000	3.430992000	3.642525000
H	0.061110000	3.375161000	4.695368000
C	-1.304646000	4.519941000	-0.813659000
C	-1.270941000	5.794112000	-1.409128000
C	-1.514191000	5.906814000	-2.779518000
H	-1.476270000	6.889835000	-3.242822000
C	-1.812641000	4.788577000	-3.547643000
H	-2.007681000	4.893745000	-4.611646000
C	-1.876918000	3.537199000	-2.946751000
H	-2.132451000	2.661552000	-3.539277000
C	-1.631389000	3.386442000	-1.581590000
C	-1.011802000	7.025736000	-0.586743000
H	-0.790083000	7.882541000	-1.229047000
H	-0.173868000	6.883671000	0.100336000
H	-1.884515000	7.281118000	0.025392000
C	-1.728503000	2.025154000	-0.951167000
H	-2.268578000	1.331364000	-1.601815000
H	-2.239772000	2.068322000	0.015440000
C	2.356264000	2.538199000	4.318139000
C	2.130305000	1.246405000	4.836374000
C	2.897382000	0.830267000	5.929208000
H	2.727468000	-0.156795000	6.352317000
C	3.859713000	1.657917000	6.490174000
H	4.439877000	1.318781000	7.344192000
C	4.076430000	2.924512000	5.959315000
H	4.831752000	3.566432000	6.402416000
C	3.335306000	3.388317000	4.872988000
C	1.065733000	0.320032000	4.270228000
H	0.698145000	0.766378000	3.340607000
C	3.518029000	4.797402000	4.342444000
H	3.243021000	4.781304000	3.280906000
C	4.609633000	2.765579000	-0.674169000
C	5.062803000	4.084547000	-0.810478000
H	4.817835000	4.825860000	-0.054863000
C	5.806102000	4.464166000	-1.922404000
H	6.144615000	5.491993000	-2.014185000
C	6.096942000	3.537240000	-2.920172000
H	6.670731000	3.836715000	-3.792531000
C	5.638139000	2.230240000	-2.799272000
H	5.850369000	1.502767000	-3.577704000
C	4.899850000	1.844406000	-1.683003000
H	4.540319000	0.823588000	-1.611440000
C	3.103687000	0.596398000	0.550118000
C	1.730569000	0.378523000	0.399923000
H	1.046004000	1.218543000	0.505353000
C	1.238430000	-0.897222000	0.133735000
H	0.168707000	-1.047992000	0.020270000
C	2.115760000	-1.971040000	0.021802000
H	1.734607000	-2.967425000	-0.183075000
C	3.485798000	-1.768180000	0.182513000
H	4.173593000	-2.605284000	0.103868000
C	3.978760000	-0.494199000	0.446554000
H	5.046460000	-0.346569000	0.581436000
C	5.094901000	2.046063000	2.026120000
C	5.003180000	1.071950000	3.026472000
H	4.099155000	0.485232000	3.134525000
C	6.064390000	0.841978000	3.895531000
H	5.964486000	0.083138000	4.665342000
C	7.234890000	1.586132000	3.786437000
H	8.064490000	1.405069000	4.463916000
C	7.335525000	2.562365000	2.800104000
H	8.245686000	3.146677000	2.698630000
C	6.276388000	2.790180000	1.925505000
H	6.389966000	3.536588000	1.147539000
H	-0.733881000	1.602521000	-0.768537000
C	1.898454000	4.202732000	-0.438325000
C	2.253933000	5.548321000	-0.517392000
C	1.687401000	3.559623000	-1.664799000
C	2.400079000	6.242946000	-1.714820000
C	1.829089000	4.220141000	-2.881917000
C	2.185961000	5.565317000	-2.909733000
Cl	2.587725000	6.434489000	0.972790000
H	1.411357000	2.507602000	-1.673807000
H	2.678214000	7.292459000	-1.704252000
H	1.650558000	3.684604000	-3.809932000
H	2.293184000	6.089570000	-3.854891000
C	-0.121681000	0.195001000	5.231810000
H	0.192404000	-0.239855000	6.187382000
H	-0.574346000	1.167807000	5.445406000
H	-0.896896000	-0.451687000	4.806711000
C	1.613490000	-1.067570000	3.925237000
H	1.984982000	-1.592148000	4.812164000
H	0.821955000	-1.685348000	3.488178000
H	2.426601000	-1.014756000	3.194992000
C	4.953405000	5.311601000	4.444104000
H	5.669309000	4.600300000	4.023208000
H	5.045749000	6.255949000	3.897657000
H	5.242059000	5.513959000	5.482005000
C	2.562564000	5.770451000	5.044767000
H	2.771483000	5.805803000	6.120162000
H	2.682348000	6.781804000	4.641456000
H	1.516175000	5.482493000	4.912518000

68

F

Ni	0.854345000	3.055733000	1.084257000
N	-0.612974000	4.236527000	1.360218000
N	1.292024000	3.425649000	2.897659000
C	-0.456981000	4.919802000	2.536512000
C	-1.364745000	5.987453000	2.594404000
H	-1.467522000	6.712720000	3.390685000
C	-2.086463000	5.932964000	1.410690000
H	-2.850919000	6.631552000	1.113568000
C	-1.609981000	4.821267000	0.657331000
C	0.553442000	4.402977000	3.368387000
H	0.718087000	4.768984000	4.381764000
C	-2.060869000	4.308151000	-0.644195000
C	-3.355857000	4.604703000	-1.150984000
C	-3.730931000	4.128513000	-2.407153000
H	-4.726981000	4.364365000	-2.773128000
C	-2.879624000	3.358271000	-3.185585000
H	-3.192119000	3.003662000	-4.163135000
C	-1.632515000	3.028034000	-2.681512000
H	-0.959878000	2.404237000	-3.264622000
C	-1.216039000	3.478167000	-1.428123000
C	-4.397592000	5.381085000	-0.390633000
H	-5.385735000	5.187181000	-0.815838000
H	-4.230720000	6.462906000	-0.449989000
H	-4.421522000	5.109987000	0.667980000

C	0.140759000	3.011353000	-0.991329000
H	0.805086000	3.846874000	-0.690091000
H	0.678721000	2.496285000	-1.789193000
C	2.208442000	2.775413000	3.778792000
C	1.754184000	1.667014000	4.520347000
C	2.646306000	1.059160000	5.404965000
H	2.318342000	0.206856000	5.992759000
C	3.946786000	1.526208000	5.545679000
H	4.626212000	1.041456000	6.241236000
C	4.379233000	2.608969000	4.791976000
H	5.399855000	2.963504000	4.902119000
C	3.527191000	3.250555000	3.890809000
C	0.326458000	1.164746000	4.393864000
H	-0.048396000	1.490214000	3.415178000
C	4.007142000	4.445818000	3.091014000
H	3.330100000	4.559812000	2.237512000
H	0.027458000	2.200245000	-0.212833000
C	2.353692000	1.948238000	0.741868000
C	3.452779000	2.362906000	-0.012356000
C	2.383079000	0.614623000	1.174770000
C	4.529359000	1.533558000	-0.322548000
C	3.439191000	-0.243749000	0.877260000
C	4.518228000	0.218460000	0.129184000
Cl	3.524393000	4.015646000	-0.625838000
H	1.553633000	0.235654000	1.767985000
H	5.358366000	1.917449000	-0.908872000
H	3.421132000	-1.270504000	1.232637000
H	5.351154000	-0.438129000	-0.105098000
C	-0.574158000	1.792861000	5.464287000
H	-0.219854000	1.529939000	6.467289000
H	-0.591540000	2.884231000	5.392454000
H	-1.604025000	1.434016000	5.362295000
C	0.218488000	-0.360509000	4.448155000
H	0.438791000	-0.749811000	5.447979000
H	-0.801053000	-0.672766000	4.200117000
H	0.904579000	-0.842039000	3.744786000
C	5.417682000	4.256252000	2.531453000
H	5.500271000	3.318873000	1.974360000
H	5.661188000	5.078148000	1.851001000
H	6.175462000	4.253247000	3.322891000
C	3.930600000	5.728642000	3.927303000
H	4.574854000	5.655349000	4.810852000
H	4.259457000	6.592057000	3.339066000
H	2.912401000	5.928113000	4.275899000

68

TSFD

Ni	0.468668000	2.820413000	1.170115000
N	-0.856956000	4.067081000	1.521980000
N	1.068565000	3.275644000	2.994529000
C	-0.893388000	4.512563000	2.811182000
C	-2.010578000	5.345424000	2.983573000
H	-2.315843000	5.846774000	3.892673000
C	-2.642377000	5.399181000	1.744101000
H	-3.555471000	5.930669000	1.527226000
C	-1.888753000	4.599937000	0.839400000
C	0.208406000	4.073579000	3.577784000
H	0.358693000	4.386775000	4.611720000
C	-1.999924000	4.349676000	-0.600451000
C	-2.653873000	5.254522000	-1.471312000
C	-2.637966000	5.016113000	-2.846298000
H	-3.132281000	5.727325000	-3.503243000
C	-1.993838000	3.912095000	-3.386725000
H	-1.986147000	3.749735000	-4.460556000
C	-1.360957000	3.017946000	-2.537082000
H	-0.865935000	2.141010000	-2.947226000
C	-1.355259000	3.208612000	-1.152816000
C	-3.344704000	6.500824000	-0.990987000
H	-3.571120000	7.155365000	-1.836520000
H	-2.728892000	7.058870000	-0.279555000
H	-4.294416000	6.274878000	-0.492332000
C	-0.724799000	2.137305000	-0.325878000
H	0.691440000	1.942694000	-0.055203000
H	-0.503576000	1.277145000	-0.969984000
C	2.178314000	2.793045000	3.745886000
C	2.019585000	1.655725000	4.562325000
C	3.120140000	1.217481000	5.302040000
H	3.017778000	0.348965000	5.946426000
C	4.343612000	1.868976000	5.224730000
H	5.187872000	1.512785000	5.808512000
C	4.487814000	2.972956000	4.394082000
H	5.449939000	3.472746000	4.332298000
C	3.417343000	3.453373000	3.638406000
C	0.688508000	0.931768000	4.664412000
H	0.108242000	1.193297000	3.770802000
C	3.568416000	4.684588000	2.765510000
H	2.800823000	4.624967000	1.985131000
H	-1.387027000	1.760527000	0.465226000
C	1.917021000	1.619694000	0.611257000
C	3.028475000	2.114934000	-0.079751000
C	2.027839000	0.329261000	1.148434000
C	4.220709000	1.405356000	-0.184631000
C	3.209382000	-0.399639000	1.058543000
C	4.308293000	0.144846000	0.398703000
Cl	2.916560000	3.672980000	-0.863041000
H	1.169291000	-0.104982000	1.654724000
H	5.060462000	1.833087000	-0.722789000
H	3.273597000	-1.390424000	1.498985000
H	5.234917000	-0.416411000	0.321499000
C	-0.106845000	1.398364000	5.889433000
H	0.450515000	1.193027000	6.810221000
H	-0.311225000	2.472294000	5.858622000
H	-1.067387000	0.875227000	5.950704000
C	0.839919000	-0.591125000	4.692025000
H	1.282663000	-0.939205000	5.631427000
H	-0.141392000	-1.069080000	4.604282000
H	1.472607000	-0.948501000	3.874317000
C	4.926846000	4.764779000	2.069226000
H	5.157683000	3.836943000	1.537804000
H	4.924508000	5.581476000	1.340747000
H	5.739348000	4.964358000	2.776885000
C	3.307712000	5.959763000	3.576245000
H	4.032354000	6.053636000	4.393057000
H	3.396219000	6.846581000	2.939470000
H	2.306219000	5.962355000	4.017080000

68

G

Ni	0.392329000	2.535602000	1.118423000
N	-0.657479000	4.032703000	1.414885000
N	1.334350000	3.205430000	2.897857000
C	-0.438451000	4.699789000	2.587062000
C	-1.364940000	5.746968000	2.710490000
H	-1.439633000	6.452007000	3.528303000
C	-2.150936000	5.702646000	1.563158000
H	-2.985807000	6.349074000	1.341301000
C	-1.688597000	4.621678000	0.767414000
C	0.639908000	4.215412000	3.354755000
H	0.873916000	4.657917000	4.324970000
C	-2.108380000	4.105039000	-0.532774000
C	-2.750343000	4.898331000	-1.509693000
C	-3.021885000	4.342927000	-2.761615000
H	-3.501580000	4.963469000	-3.514478000
C	-2.673988000	3.033292000	-3.067519000
H	-2.888407000	2.625881000	-4.051558000
C	-2.051523000	2.250625000	-2.105067000
H	-1.796655000	1.216499000	-2.326410000
C	-1.770812000	2.758301000	-0.832480000
C	-3.122632000	6.336255000	-1.277730000
H	-3.371474000	6.821331000	-2.225214000
H	-2.307658000	6.895340000	-0.808159000
H	-3.998572000	6.429831000	-0.624869000
C	-1.148038000	1.874167000	0.180064000
H	0.856791000	1.835009000	-1.254907000
H	-0.951779000	0.884402000	-0.249853000
C	2.359185000	2.687521000	3.734570000
C	2.109373000	1.475000000	4.414403000
C	3.112961000	0.945130000	5.224671000
H	2.935959000	0.015952000	5.758330000
C	4.334781000	1.593271000	5.369494000
H	5.104237000	1.169989000	6.009089000
C	4.565411000	2.784744000	4.696587000
H	5.522145000	3.285816000	4.814915000
C	3.595134000	3.352942000	3.864820000
C	0.751998000	0.804503000	4.305332000
H	0.351784000	1.052738000	3.311937000
C	3.886955000	4.655974000	3.139638000
H	3.181854000	4.727816000	2.303325000
H	-1.798465000	1.752603000	1.059067000
C	1.635737000	1.796415000	-0.502186000
C	2.788388000	2.589725000	-0.686358000
C	1.656043000	0.811082000	0.509731000
C	3.919932000	2.401501000	0.080725000
C	2.821978000	0.613321000	1.277039000
C	3.934891000	1.400643000	1.065199000
Cl	2.786345000	3.780593000	-1.946488000
H	0.852397000	0.082363000	0.569251000
H	4.795606000	3.016507000	-0.097421000
H	2.844375000	-0.166889000	2.029798000
H	4.827663000	1.254905000	1.664803000
C	-0.220017000	1.379158000	5.343059000
H	0.145901000	1.188267000	6.358071000
H	-0.341932000	2.459513000	5.226901000
H	-1.208483000	0.917208000	5.244951000
C	0.801327000	-0.718569000	4.421662000
H	1.066239000	-1.043130000	5.433879000
H	-0.183180000	-1.142170000	4.198674000
H	1.524618000	-1.162659000	3.729978000
C	5.304754000	4.711388000	2.562994000
H	5.545864000	3.814393000	1.986784000
H	5.408132000	5.579517000	1.904044000
H	6.059981000	4.814142000	3.349786000
C	3.670993000	5.869550000	4.053122000
H	4.335961000	5.818188000	4.922653000
H	3.888561000	6.799021000	3.515899000
H	2.645363000	5.931630000	4.425555000

102

E-wB97XD

Ni	1.732243000	3.467015000	1.331061000
P	3.641844000	2.328976000	0.910203000
N	-0.148139000	3.959411000	1.439966000
N	1.513937000	3.034752000	3.279331000
C	-0.580771000	3.922677000	2.748320000
C	-1.913102000	4.346574000	2.849115000
H	-2.505496000	4.413039000	3.760053000
C	-2.304413000	4.643544000	1.545988000
H	-3.276798000	4.991239000	1.201864000
C	-1.195557000	4.389188000	0.708098000
C	0.337397000	3.422618000	3.686764000
H	0.068841000	3.355386000	4.749697000
C	-1.256560000	4.495954000	-0.772421000
C	-1.135470000	5.748928000	-1.396348000
C	-1.272917000	5.824290000	-2.785857000
H	-1.161601000	6.792835000	-3.280250000
C	-1.545360000	4.688574000	-3.539966000
H	-1.650679000	4.764570000	-4.624807000
C	-1.685586000	3.454853000	-2.910559000
H	-1.907635000	2.562262000	-3.501656000
C	-1.547197000	3.343200000	-1.526097000
C	-0.888303000	6.991884000	-0.582866000
H	-0.548231000	7.820101000	-1.220625000
H	-0.133031000	6.822872000	0.196454000
H	-1.809819000	7.311951000	-0.070589000
C	-1.705914000	2.010199000	-0.843296000
H	-1.960621000	1.219313000	-1.562703000
H	-2.490841000	2.050861000	-0.072695000
C	2.378824000	2.529126000	4.290305000
C	2.186219000	1.219942000	4.769784000
C	2.976222000	0.788681000	5.841262000
H	2.832473000	-0.216370000	6.245733000
C	3.939249000	1.619317000	6.399909000
H	4.544389000	1.268034000	7.238703000
C	4.146858000	2.894519000	5.879733000
H	4.924519000	3.526378000	6.311654000
C	3.377712000	3.373102000	4.818769000
C	1.138385000	0.295940000	4.161916000
H	0.802961000	0.756460000	3.221209000
C	3.572273000	4.770119000	4.249720000
H	3.387793000	4.693026000	3.165819000
C	4.541880000	2.776627000	-0.628112000
C	4.986925000	4.098313000	-0.766826000
H	4.781645000	4.831026000	0.017217000
C	5.649905000	4.505016000	-1.919417000
H	5.975710000	5.542548000	-2.015439000
C	5.871273000	3.598405000	-2.955499000
H	6.381529000	3.920000000	-3.865896000
C	5.425229000	2.286061000	-2.829249000
H	5.582846000	1.572171000	-3.640538000
C	4.762686000	1.875928000	-1.672663000
H	4.399153000	0.850030000	-1.605622000
C	3.030909000	0.623793000	0.615389000
C	1.650093000	0.436425000	0.498956000
H	0.983743000	1.296880000	0.609353000
C	1.120975000	-0.828642000	0.249861000
H	0.039819000	-0.953526000	0.161451000
C	1.972015000	-1.923224000	0.122850000
H	1.561786000	-2.917507000	-0.066537000
C	3.352185000	-1.749354000	0.243924000
H	4.022363000	-2.606212000	0.148567000
C	3.880389000	-0.484515000	0.487085000
H	4.961183000	-0.359435000	0.585138000
C	5.041494000	2.071635000	2.087239000
C	4.997175000	1.038638000	3.030610000
H	4.127503000	0.387119000	3.090721000
C	6.055595000	0.826941000	3.908351000
H	5.989989000	0.017670000	4.637660000
C	7.179769000	1.647749000	3.862885000
H	8.011474000	1.482014000	4.550911000
C	7.235084000	2.680758000	2.930589000
H	8.110603000	3.331538000	2.881218000
C	6.175619000	2.891670000	2.050153000
H	6.253670000	3.699978000	1.323524000
H	-0.779830000	1.721807000	-0.326147000
C	1.871832000	4.211040000	-0.398926000
C	2.230908000	5.554383000	-0.476127000
C	1.675482000	3.563828000	-1.626533000
C	2.395049000	6.248171000	-1.672720000
C	1.840194000	4.219180000	-2.842956000
C	2.199869000	5.566448000	-2.869032000
Cl	2.562150000	6.439695000	1.023487000
H	1.394804000	2.506471000	-1.635534000
H	2.677636000	7.302266000	-1.660446000
H	1.676771000	3.678059000	-3.777025000
H	2.323730000	6.089200000	-3.819676000
C	-0.084488000	0.165262000	5.077626000
H	0.194378000	-0.280475000	6.045969000
H	-0.543885000	1.143189000	5.282029000
H	-0.850858000	-0.476761000	4.616449000
C	1.700092000	-1.083817000	3.806504000
H	2.042957000	-1.633652000	4.697115000
H	0.923997000	-1.694744000	3.320932000
H	2.542373000	-1.012045000	3.102328000
C	4.985827000	5.319531000	4.439491000
H	5.749789000	4.592481000	4.133161000
H	5.113786000	6.229074000	3.833198000
H	5.178237000	5.598405000	5.488199000
C	2.548693000	5.764617000	4.814592000
H	2.647496000	5.839707000	5.909465000
H	2.714433000	6.765481000	4.387457000
H	1.514230000	5.479601000	4.582030000

102

TSED-wB97XD

Ni	1.311452000	2.635891000	1.450417000
P	2.973307000	1.022225000	1.292454000
N	0.230121000	4.185841000	1.330050000
N	1.872179000	3.467436000	3.146536000
C	0.526882000	5.144787000	2.264011000
C	-0.305115000	6.264332000	2.073373000
H	-0.320320000	7.174455000	2.669684000
C	-1.118292000	5.943137000	0.994688000
H	-1.915342000	6.552752000	0.578316000
C	-0.761223000	4.638122000	0.552266000
C	1.391790000	4.686364000	3.258922000
H	1.595519000	5.272500000	4.162194000
C	-1.382894000	3.826430000	-0.513534000
C	-1.781282000	4.406420000	-1.735790000
C	-2.413953000	3.607124000	-2.693008000
H	-2.713803000	4.051225000	-3.645576000
C	-2.648045000	2.257389000	-2.451011000
H	-3.151176000	1.646150000	-3.204471000
C	-2.213951000	1.680343000	-1.265104000
H	-2.361221000	0.610120000	-1.095073000
C	-1.557396000	2.435104000	-0.282193000
C	-1.495676000	5.848692000	-2.077475000
H	-0.520563000	6.171233000	-1.686035000
H	-2.258180000	6.529675000	-1.667217000
H	-1.492207000	5.985512000	-3.168195000
C	-0.889616000	1.757071000	0.852353000
H	-1.162997000	0.693349000	0.879473000
H	-1.131414000	2.207694000	1.825238000
C	2.444704000	2.919994000	4.332450000
C	1.662605000	2.012055000	5.082991000
C	2.165570000	1.561333000	6.302893000
H	1.577089000	0.870185000	6.908406000
C	3.417824000	1.964164000	6.757597000
H	3.799376000	1.591296000	7.710547000
C	4.182890000	2.836452000	5.996426000
H	5.163900000	3.148223000	6.362289000
C	3.709668000	3.344973000	4.781488000
C	0.304864000	1.549831000	4.567568000
H	0.406050000	1.441946000	3.474531000
C	4.527053000	4.389366000	4.032889000
H	4.063942000	4.524870000	3.043853000
C	4.030374000	0.488934000	2.703948000
C	3.401735000	-0.206940000	3.741520000
H	2.318976000	-0.341849000	3.722795000
C	4.141820000	-0.745964000	4.786251000

H	3.630357000	-1.273505000	5.593688000
C	5.529307000	-0.606364000	4.803726000
H	6.112575000	-1.027293000	5.625401000
C	6.166688000	0.062737000	3.763511000
H	7.253925000	0.165607000	3.760213000
C	5.422992000	0.603790000	2.714107000
H	5.941570000	1.106582000	1.896914000
C	4.199484000	1.692123000	0.109826000
C	4.812060000	2.901613000	0.457742000
H	4.549341000	3.392672000	1.396164000
C	5.739604000	3.495197000	-0.392084000
H	6.202656000	4.442243000	-0.108675000
C	6.052940000	2.892599000	-1.609119000
H	6.772409000	3.361632000	-2.283654000
C	5.431239000	1.699122000	-1.970200000
H	5.652220000	1.234486000	-2.933179000
C	4.508810000	1.098442000	-1.116531000
H	4.006254000	0.183104000	-1.429958000
C	2.520305000	-0.656857000	0.710041000
C	1.183966000	-1.056341000	0.708200000
H	0.413808000	-0.332417000	0.971315000
C	0.837286000	-2.362148000	0.359193000
H	-0.213435000	-2.659570000	0.358760000
C	1.826943000	-3.277885000	0.014319000
H	1.557188000	-4.299466000	-0.262358000
C	3.168939000	-2.891766000	0.037185000
H	3.951035000	-3.611174000	-0.214300000
C	3.515998000	-1.593518000	0.395154000
H	4.570566000	-1.312194000	0.443875000
H	0.484263000	2.157896000	0.100104000
C	1.392155000	2.473430000	-0.894215000
C	2.057711000	3.551231000	-1.488150000
C	1.167063000	1.364415000	-1.720338000
C	2.493235000	3.522914000	-2.813546000
C	1.585375000	1.308112000	-3.047038000
C	2.257308000	2.396338000	-3.595835000
Cl	2.441857000	5.026291000	-0.606844000
H	0.609442000	0.518855000	-1.308301000
H	3.018027000	4.386914000	-3.223814000
H	1.376261000	0.422335000	-3.650772000
H	2.595140000	2.378210000	-4.634406000
C	-0.795216000	2.590334000	4.818159000
H	-0.907836000	2.780125000	5.897571000
H	-0.588194000	3.546482000	4.320378000
H	-1.759444000	2.225238000	4.432591000
C	-0.136770000	0.191845000	5.116935000
H	-0.412951000	0.250488000	6.181689000
H	-1.025743000	-0.156818000	4.570722000
H	0.642912000	-0.578379000	5.016000000
C	5.982281000	3.968220000	3.811730000
H	6.047493000	2.986776000	3.326554000
H	6.498553000	4.703817000	3.175660000
H	6.537768000	3.909132000	4.760715000
C	4.476730000	5.744478000	4.752479000
H	4.938161000	5.672672000	5.750189000
H	5.024589000	6.508982000	4.180268000
H	3.446819000	6.102178000	4.892850000

102

D+PhCl-wB97XD

Ni	0.693954000	4.126060000	1.233641000
P	2.700195000	3.686554000	0.419527000
N	-0.932975000	4.947860000	1.689130000
N	0.690193000	3.672050000	3.238069000
C	-1.430987000	4.592678000	2.909703000
C	-2.716195000	5.139238000	3.071749000
H	-3.368295000	5.034120000	3.937084000
C	-2.975757000	5.852905000	1.901800000
H	-3.891694000	6.388585000	1.673757000
C	-1.833314000	5.726799000	1.064187000
C	-0.481932000	3.956214000	3.740577000
H	-0.691487000	3.773450000	4.803120000
C	-1.465941000	6.324584000	-0.229523000
C	-1.979486000	7.564522000	-0.676452000
C	-1.473821000	8.124294000	-1.853560000
H	-1.861877000	9.089326000	-2.189881000
C	-0.479108000	7.486532000	-2.588166000
H	-0.090204000	7.944949000	-3.500424000
C	0.012174000	6.262474000	-2.155238000
H	0.784953000	5.749132000	-2.733612000
C	-0.470348000	5.655843000	-0.988003000
C	-3.030930000	8.331498000	0.083640000
H	-3.136353000	9.346752000	-0.323156000
H	-2.783824000	8.410790000	1.152597000
H	-4.016843000	7.845514000	0.013527000
C	0.077123000	4.327925000	-0.591966000
H	0.747160000	3.979253000	-1.385860000
H	-0.736051000	3.581300000	-0.514394000
C	1.687969000	3.239311000	4.150600000
C	1.632356000	1.957698000	4.730699000
C	2.609886000	1.616461000	5.672097000
H	2.576017000	0.633306000	6.148554000
C	3.629770000	2.498146000	6.002536000
H	4.387305000	2.210235000	6.734718000
C	3.692322000	3.747967000	5.390354000
H	4.505952000	4.428073000	5.647784000
C	2.726762000	4.144719000	4.466127000
C	0.555713000	0.947721000	4.355262000
H	0.023267000	1.342123000	3.476944000
C	2.743996000	5.527923000	3.832450000
H	2.384166000	5.397835000	2.799286000
C	3.391627000	5.275429000	-0.173432000
C	2.698611000	6.453155000	0.127120000
H	1.755541000	6.403005000	0.676504000
C	3.188371000	7.689424000	-0.292307000
H	2.627020000	8.596258000	-0.060048000
C	4.373875000	7.760074000	-1.018401000
H	4.755551000	8.727749000	-1.351373000
C	5.069883000	6.590612000	-1.331137000
H	5.996028000	6.640949000	-1.907679000
C	4.580947000	5.356327000	-0.914139000
H	5.124807000	4.445013000	-1.174957000
C	2.763298000	2.568470000	-1.049624000

C	2.887913000	3.058644000	-2.355033000
H	3.021527000	4.126696000	-2.532257000
C	2.821818000	2.196242000	-3.448542000
H	2.920211000	2.600949000	-4.458095000
C	2.621682000	0.832567000	-3.255975000
H	2.571630000	0.156423000	-4.111975000
C	2.484416000	0.334643000	-1.962064000
H	2.334445000	-0.733802000	-1.794902000
C	2.551373000	1.195594000	-0.871220000
H	2.436276000	0.776565000	0.128713000
C	4.015207000	2.990535000	1.503245000
C	3.709344000	1.817125000	2.202687000
H	2.705188000	1.395876000	2.135568000
C	4.651322000	1.197995000	3.016561000
H	4.382367000	0.288752000	3.557436000
C	5.916117000	1.762937000	3.170058000
H	6.655020000	1.287588000	3.818273000
C	6.222549000	2.950391000	2.509924000
H	7.203388000	3.412711000	2.639622000
C	5.280834000	3.559036000	1.679732000
H	5.541705000	4.492320000	1.180817000
H	5.176456000	-0.974560000	0.916947000
C	5.410674000	-0.698244000	-0.111976000
C	5.181377000	-1.597989000	-1.149669000
C	5.924301000	0.562171000	-0.409278000
C	5.470083000	-1.265387000	-2.471383000
C	6.201482000	0.917050000	-1.728062000
C	5.978335000	0.000776000	-2.754486000
Cl	4.482212000	-3.157855000	-0.793040000
H	6.095388000	1.272043000	0.401044000
H	5.281584000	-1.987247000	-3.267261000
H	6.592639000	1.910465000	-1.956092000
H	6.192401000	0.273352000	-3.789974000
C	-0.468315000	0.772569000	5.483345000
H	0.012513000	0.375068000	6.391498000
H	-0.943447000	1.726668000	5.753413000
H	-1.262883000	0.070772000	5.186206000
C	1.142530000	-0.410479000	3.952356000
H	1.648659000	-0.905565000	4.795814000
H	0.343809000	-1.085155000	3.608550000
H	1.872123000	-0.315248000	3.134481000
C	4.129173000	6.168936000	3.766941000
H	4.879442000	5.477174000	3.361417000
H	4.098937000	7.057351000	3.117791000
H	4.471895000	6.500726000	4.760347000
C	1.758281000	6.477859000	4.526839000
H	2.039748000	6.621403000	5.582307000
H	1.765987000	7.462704000	4.034420000
H	0.726659000	6.102903000	4.495007000

90

D-wB97XD

Ni	1.211169000	4.238475000	0.827361000
P	2.748998000	5.678550000	0.191715000
N	-0.550459000	3.640889000	1.000378000
N	0.935734000	4.848914000	2.740614000
C	-1.109762000	3.856931000	2.230839000
C	-2.411844000	3.329240000	2.249943000
H	-3.109138000	3.335405000	3.085809000
C	-2.627171000	2.798885000	0.979287000
H	-3.525205000	2.281254000	0.662271000
C	-1.445942000	3.020811000	0.212598000
C	-0.255158000	4.507007000	3.152100000
H	-0.557601000	4.669050000	4.195577000
C	-1.098232000	2.728932000	-1.191638000
C	-2.084807000	2.498247000	-2.183558000
C	-1.691943000	2.297105000	-3.509670000
H	-2.460777000	2.130636000	-4.268525000
C	-0.352173000	2.318814000	-3.878820000
H	-0.063697000	2.159170000	-4.920477000
C	0.614453000	2.546309000	-2.910086000
H	1.670676000	2.563061000	-3.190153000
C	0.275510000	2.754304000	-1.565108000
C	-3.563984000	2.488209000	-1.891173000
H	-4.136847000	2.484285000	-2.828606000
H	-3.871786000	3.366842000	-1.305895000
H	-3.861552000	1.593471000	-1.322454000
C	1.389413000	2.973336000	-0.593622000
H	2.342951000	3.025171000	-1.140055000
H	1.447275000	2.109863000	0.102216000
C	1.895590000	5.289526000	3.690890000
C	2.949415000	4.398302000	4.010361000
C	3.894265000	4.802317000	4.951911000
H	4.715596000	4.134774000	5.215199000
C	3.810746000	6.049342000	5.566966000
H	4.558555000	6.345051000	6.306106000
C	2.779907000	6.914822000	5.233804000
H	2.721909000	7.892166000	5.719251000
C	1.807495000	6.560032000	4.289815000
C	3.015595000	3.021909000	3.360049000
H	2.765714000	3.172021000	2.293357000
C	0.685221000	7.536900000	3.971923000
H	0.138659000	7.145803000	3.102953000
C	1.941032000	7.328810000	0.232131000
C	0.561544000	7.400823000	0.448916000
H	-0.000535000	6.490353000	0.671127000
C	-0.100047000	8.626529000	0.372892000
H	-1.177979000	8.668475000	0.542075000
C	0.611436000	9.787383000	0.083824000
H	0.093753000	10.747448000	0.027078000
C	1.988159000	9.722560000	-0.140576000
H	2.548405000	10.629451000	-0.377560000
C	2.647589000	8.499108000	-0.077677000
H	3.720570000	8.451766000	-0.280628000
C	3.289332000	5.664553000	-1.565333000
C	2.285719000	5.457722000	-2.520432000
H	1.261982000	5.250344000	-2.199702000
C	2.582822000	5.500321000	-3.878968000
H	1.788222000	5.325982000	-4.607434000
C	3.888512000	5.751081000	-4.301046000
H	4.124507000	5.779783000	-5.367099000
C	4.890649000	5.966022000	-3.357429000
H	5.914091000	6.168753000	-3.680563000

C	4.594102000	5.927093000	-1.994576000
H	5.389618000	6.107361000	-1.269191000
C	4.300167000	5.851287000	1.144110000
C	5.273575000	4.853910000	0.994149000
H	5.107395000	4.027432000	0.297225000
C	6.458446000	4.907108000	1.723038000
H	7.213414000	4.129886000	1.586232000
C	6.671389000	5.939701000	2.636007000
H	7.596396000	5.976509000	3.215318000
C	5.694631000	6.916079000	2.813489000
H	5.841246000	7.715392000	3.542229000
C	4.517006000	6.875836000	2.070104000
H	3.758154000	7.642040000	2.230566000
C	1.211428000	8.920453000	3.580637000
H	1.894699000	8.860782000	2.720979000
H	0.378142000	9.578529000	3.291473000
H	1.744607000	9.407784000	4.412060000
C	-0.304719000	7.639711000	5.138784000
H	0.185544000	8.043754000	6.038981000
H	-1.142283000	8.307024000	4.882548000
H	-0.722079000	6.657301000	5.405317000
C	1.976557000	2.056865000	3.948696000
H	2.157913000	1.910939000	5.025508000
H	0.946515000	2.411884000	3.819771000
H	2.047704000	1.074920000	3.456055000
C	4.398112000	2.373471000	3.421411000
H	4.418375000	1.478906000	2.780886000
H	5.187251000	3.056340000	3.081334000
H	4.645336000	2.044675000	4.443687000

12

PhCl-wB97XD

C	3.014730000	2.178026000	0.233623000
C	2.712379000	1.188156000	-0.700017000
C	4.346251000	2.396831000	0.583437000
C	3.728184000	0.426791000	-1.276757000
C	5.373011000	1.643805000	0.015825000
C	5.055332000	0.657576000	-0.916486000
H	1.670578000	1.013185000	-0.977003000
Cl	4.733750000	3.632838000	1.750554000
H	3.485454000	-0.347102000	-2.007521000
H	6.407874000	1.832425000	0.304797000
H	5.856337000	0.065256000	-1.363956000
H	2.227665000	2.779354000	0.690705000

102

E'-wB97XD

Ni	1.762849000	3.237839000	1.394774000
P	3.003734000	4.303203000	-0.110437000
N	0.134640000	4.382125000	1.413471000
N	1.346986000	3.022228000	3.312737000
C	-0.265800000	4.594596000	2.714580000
C	-1.356004000	5.474468000	2.766276000
H	-1.877549000	5.810794000	3.660838000
C	-1.641005000	5.789443000	1.437976000
H	-2.443595000	6.420135000	1.060105000
C	-0.715389000	5.084817000	0.638166000
C	0.401713000	3.829229000	3.694858000
H	0.122518000	3.898058000	4.754657000
C	-0.804524000	4.955708000	-0.838160000
C	-0.508904000	6.031486000	-1.693958000
C	-0.649058000	5.854337000	-3.076274000
H	-0.392564000	6.678351000	-3.747260000
C	-1.134173000	4.662531000	-3.597987000
H	-1.244552000	4.542860000	-4.678303000
C	-1.500483000	3.628345000	-2.739589000
H	-1.920119000	2.704279000	-3.146364000
C	-1.341905000	3.756026000	-1.358937000
C	-0.146023000	7.390136000	-1.156327000
H	0.491452000	7.941130000	-1.861402000
H	0.364764000	7.333743000	-0.188161000
H	-1.060915000	7.985834000	-1.002440000
C	-1.800308000	2.656789000	-0.435533000
H	-2.230826000	1.818720000	-1.001016000
H	-2.563943000	3.036349000	0.261395000
C	2.063432000	2.288795000	4.295952000
C	1.713111000	0.945967000	4.517554000
C	2.430502000	0.229355000	5.476971000
H	2.177941000	-0.814752000	5.671615000
C	3.471254000	0.825232000	6.182261000
H	4.021020000	0.251815000	6.931875000
C	3.830088000	2.142934000	5.917108000
H	4.671775000	2.583971000	6.454246000
C	3.146928000	2.896886000	4.959194000
C	0.578795000	0.321201000	3.724256000
H	0.531087000	0.865210000	2.768671000
C	3.594131000	4.307404000	4.591178000
H	3.338029000	4.442307000	3.526285000
C	2.830447000	6.116800000	0.135455000
C	2.430708000	6.571542000	1.399045000
H	2.127336000	5.858384000	2.166729000
C	2.396789000	7.934396000	1.685477000
H	2.073587000	8.267699000	2.673597000
C	2.759380000	8.861644000	0.710263000
H	2.729628000	9.930764000	0.931110000
C	3.147359000	8.418451000	-0.552990000
H	3.418557000	9.139346000	-1.327206000
C	3.180449000	7.055557000	-0.840303000
H	3.467111000	6.730445000	-1.840809000
C	2.896702000	3.969037000	-1.901291000
C	1.841289000	3.186286000	-2.368159000
H	1.098113000	2.808438000	-1.667385000
C	1.740597000	2.874627000	-3.723683000
H	0.907689000	2.260993000	-4.070663000
C	2.693163000	3.344929000	-4.621055000
H	2.613905000	3.101180000	-5.682869000
C	3.761899000	4.117767000	-4.160686000
H	4.522217000	4.474854000	-4.858595000
C	3.870549000	4.419476000	-2.807917000
H	4.734621000	4.984799000	-2.451459000
C	4.803564000	4.087299000	0.204653000
C	5.481102000	3.012166000	-0.384310000

H	4.954341000	2.342184000	-1.067380000
C	6.816810000	2.769264000	-0.083366000
H	7.328057000	1.922664000	-0.545810000
C	7.491531000	3.588840000	0.821459000
H	8.538370000	3.393377000	1.063926000
C	6.823799000	4.657248000	1.414966000
H	7.343498000	5.304410000	2.124746000
C	5.487081000	4.908825000	1.105491000
H	4.979048000	5.751908000	1.578056000
H	-0.979665000	2.272679000	0.184137000
C	3.005457000	1.798713000	1.430593000
C	2.644879000	0.611321000	0.793531000
C	4.209906000	1.746559000	2.147245000
C	3.410521000	-0.554928000	0.826048000
C	4.988154000	0.596461000	2.228477000
C	4.593772000	-0.560892000	1.557551000
Cl	1.106285000	0.529232000	-0.074955000
H	4.564735000	2.645686000	2.651025000
H	3.066327000	-1.445931000	0.298133000
H	5.912179000	0.608839000	2.810475000
H	5.200161000	-1.467993000	1.603948000
C	-0.766772000	0.523157000	4.431512000
H	-0.768981000	0.028850000	5.416408000
H	-0.986538000	1.589201000	4.590414000
H	-1.587582000	0.098275000	3.833090000
C	0.813176000	-1.153156000	3.396291000
H	0.723098000	-1.794994000	4.287601000
H	0.065205000	-1.498464000	2.666710000
H	1.810578000	-1.306364000	2.959280000
C	5.105620000	4.512900000	4.719202000
H	5.673288000	3.731010000	4.193163000
H	5.390014000	5.484464000	4.286828000
H	5.430283000	4.520049000	5.771492000
C	2.854171000	5.390626000	5.385722000
H	3.031582000	5.267958000	6.465939000
H	3.208325000	6.392158000	5.094265000
H	1.769189000	5.362000000	5.218902000

102
TSE'D-wB97XD

Ni	1.338005000	2.993846000	0.644003000
P	3.079500000	4.391710000	0.080074000
N	0.223929000	4.412405000	1.221641000
N	1.684669000	3.293062000	3.310098000
C	0.132809000	4.890323000	2.495484000
C	-0.880376000	5.859156000	2.557463000
H	-1.187249000	6.412129000	3.443926000
C	-1.424124000	5.943563000	1.271429000
H	-2.260306000	6.561919000	0.955502000
C	-0.726627000	5.009549000	0.478761000
C	0.872964000	4.261564000	3.544902000
H	0.661622000	4.593383000	4.577171000
C	-0.973842000	4.488066000	-0.874587000
C	-1.316776000	5.326862000	-1.954150000
C	-1.492197000	4.762371000	-3.222338000
H	-1.743998000	5.413261000	-4.063929000
C	-1.334287000	3.395831000	-3.427898000
H	-1.471049000	2.970794000	-4.424691000
C	-0.996579000	2.570762000	-2.360528000
H	-0.867448000	1.498789000	-2.525189000
C	-0.812450000	3.090764000	-1.075864000
C	-1.451856000	6.817649000	-1.780747000
H	-1.378490000	7.329881000	-2.750560000
H	-0.675368000	7.215460000	-1.110633000
H	-2.425192000	7.085233000	-1.340102000
C	-0.447923000	2.152453000	0.044933000
H	-0.622697000	1.114241000	-0.281172000
H	-1.032257000	2.310022000	0.960807000
C	2.199646000	2.534362000	4.377789000
C	1.460074000	1.406911000	4.801491000
C	1.981529000	0.615123000	5.827347000
H	1.418997000	-0.253711000	6.176495000
C	3.209767000	0.912277000	6.409028000
H	3.601670000	0.285484000	7.212973000
C	3.946035000	2.001182000	5.951123000
H	4.919741000	2.211831000	6.397726000
C	3.466408000	2.820623000	4.926490000
C	0.127703000	1.076906000	4.142236000
H	0.138269000	1.562440000	3.153216000
C	4.266999000	3.999536000	4.401502000
H	4.006483000	4.090840000	3.334572000
C	2.720183000	6.150442000	0.460326000
C	2.711075000	6.526645000	1.809203000
H	2.947384000	5.789764000	2.577285000
C	2.404781000	7.830377000	2.181693000
H	2.399220000	8.101171000	3.239612000
C	2.090576000	8.778485000	1.208548000
H	1.838982000	9.800786000	1.498890000
C	2.100554000	8.414973000	-0.135536000
H	1.861916000	9.151962000	-0.905426000
C	2.417466000	7.108841000	-0.510660000
H	2.428435000	6.846334000	-1.569172000
C	3.294015000	4.364942000	-1.743709000
C	2.157711000	4.538289000	-2.544686000
H	1.179612000	4.691175000	-2.086449000
C	2.255160000	4.501967000	-3.931301000
H	1.353837000	4.640210000	-4.531812000
C	3.487813000	4.262765000	-4.538240000
H	3.564651000	4.217572000	-5.626897000
C	4.620209000	4.075626000	-3.749394000
H	5.588603000	3.883944000	-4.216615000
C	4.528231000	4.133398000	-2.358778000
H	5.427523000	3.987875000	-1.758138000
C	4.804240000	4.184747000	0.668544000
C	5.305641000	2.891930000	0.857331000
H	4.649507000	2.030552000	0.748991000
C	6.648826000	2.693075000	1.170059000
H	7.021823000	1.676363000	1.310228000
C	7.505236000	3.783509000	1.305344000
H	8.557673000	3.627973000	1.552329000
C	7.010720000	5.075968000	1.133337000
H	7.674742000	5.936329000	1.241287000
C	5.669141000	5.277307000	0.815034000
H	5.296892000	6.292879000	0.666531000
H	0.909841000	1.590522000	0.170374000
C	2.256943000	1.238858000	0.298454000
C	2.787204000	0.684892000	-0.874059000
C	2.677702000	0.662287000	1.510032000
C	3.760029000	-0.316226000	-0.848670000
C	3.637369000	-0.343021000	1.558803000
C	4.194170000	-0.822669000	0.373027000
Cl	2.216542000	1.208720000	-2.438208000
H	2.264693000	1.036703000	2.444091000
H	4.159723000	-0.697738000	-1.789271000
H	3.950511000	-0.741954000	2.525702000
H	4.953812000	-1.607067000	0.392877000
C	-1.045668000	1.676921000	4.925490000
H	-1.089801000	1.257795000	5.943556000
H	-0.952939000	2.768862000	5.016477000
H	-2.002370000	1.461461000	4.424442000
C	-0.073600000	-0.422105000	3.909620000
H	-0.200896000	-0.974871000	4.853744000
H	-0.980471000	-0.593636000	3.309587000
H	0.778350000	-0.862595000	3.369820000
C	5.779027000	3.794186000	4.480730000
H	6.077746000	2.834308000	4.035395000
H	6.295862000	4.594413000	3.930845000
H	6.143553000	3.820523000	5.520240000
C	3.870848000	5.307659000	5.098114000
H	4.095256000	5.255313000	6.175374000
H	4.427312000	6.158800000	4.673940000
H	2.796887000	5.519701000	4.994317000

102
tE-wB97XD

Ni	1.612219000	2.703408000	1.585253000
P	3.478588000	3.975486000	0.815170000
N	-0.021909000	3.690458000	0.899223000
N	1.122160000	3.730750000	3.341624000
C	-0.545839000	4.503732000	1.881612000
C	-1.620887000	5.250646000	1.375693000
H	-2.224206000	5.971230000	1.925799000
C	-1.750702000	4.868944000	0.038226000
H	-2.487748000	5.214503000	-0.684289000
C	-0.758177000	3.897568000	-0.210313000
C	0.058716000	4.455564000	3.162608000
H	-0.393303000	5.006534000	3.998760000
C	-0.586024000	3.101505000	-1.455328000
C	-1.362865000	1.939602000	-1.630734000
C	-1.164887000	1.158403000	-2.773111000
H	-1.746216000	0.241429000	-2.900419000
C	-0.245274000	1.536925000	-3.744718000
H	-0.097442000	0.914799000	-4.630580000
C	0.472873000	2.718788000	-3.593864000
H	1.179053000	3.033098000	-4.366443000
C	0.310142000	3.516396000	-2.458191000
C	-2.443144000	1.554836000	-0.650428000
H	-2.552551000	0.462247000	-0.590894000
H	-3.412358000	1.967777000	-0.974522000
H	-2.254480000	1.945421000	0.358108000
C	1.040434000	4.827345000	-2.348675000
H	1.870598000	4.884690000	-3.066661000
H	1.452894000	4.979377000	-1.345781000
C	1.695429000	3.581611000	4.631431000
C	1.697244000	2.291137000	5.205631000
C	2.290477000	2.118024000	6.457482000
H	2.292780000	1.125341000	6.914350000
C	2.869964000	3.185805000	7.133125000
H	3.322574000	3.034872000	8.115481000
C	2.876843000	4.446007000	6.547478000
H	3.346462000	5.277562000	7.077343000
C	2.308674000	4.671920000	5.289541000
C	1.065607000	1.097928000	4.506055000
H	0.686622000	1.437626000	3.531746000
C	2.398208000	6.059652000	4.668808000
H	2.152758000	5.967729000	3.601491000
C	5.016053000	3.602371000	1.734338000
C	4.908681000	3.431982000	3.119218000
H	3.938271000	3.510049000	3.612070000
C	6.035087000	3.149068000	3.886290000
H	5.929377000	3.018963000	4.965300000
C	7.280300000	3.020020000	3.272099000
H	8.164960000	2.788676000	3.869329000
C	7.391201000	3.174044000	1.890946000
H	8.362148000	3.061595000	1.403776000
C	6.264678000	3.464242000	1.121821000
H	6.360221000	3.565979000	0.038955000
C	3.315764000	5.791727000	1.028798000
C	4.414857000	6.606445000	1.329079000
H	5.401760000	6.161837000	1.475605000
C	4.256701000	7.985266000	1.454382000
H	5.122460000	8.608793000	1.687720000
C	2.997572000	8.563803000	1.300664000
H	2.874431000	9.643719000	1.408695000
C	1.896691000	7.758296000	1.013156000
H	0.903488000	8.196677000	0.897034000
C	2.054601000	6.381450000	0.874224000
H	1.182428000	5.764097000	0.659981000
C	3.906086000	3.773001000	-0.950455000
C	3.501913000	2.605399000	-1.605150000
H	2.907064000	1.858886000	-1.076332000
C	3.829289000	2.406791000	-2.945298000
H	3.500177000	1.494238000	-3.445519000
C	4.547790000	3.374526000	-3.642575000
H	4.798001000	3.219412000	-4.694559000
C	4.938598000	4.550127000	-2.998697000
H	5.491416000	5.317706000	-3.544460000
C	4.618970000	4.750064000	-1.658768000
H	4.916230000	5.678986000	-1.167231000
H	0.355797000	5.668981000	-2.541658000
C	1.696553000	0.819218000	0.914687000
C	2.752829000	-0.085404000	0.780050000
C	0.420451000	0.255301000	0.724854000
C	2.595197000	-1.433701000	0.458007000
C	0.215274000	-1.087981000	0.411935000
C	1.310561000	-1.937428000	0.269999000
Cl	4.410833000	0.456773000	1.062251000
H	-0.455444000	0.898327000	0.832398000
H	3.470533000	-2.079125000	0.367591000
H	-0.799561000	-1.470185000	0.275789000
H	1.171587000	-2.991376000	0.018605000
C	-0.147764000	0.571096000	5.278158000
H	0.141761000	0.184692000	6.268561000
H	-0.896151000	1.363159000	5.431979000
H	-0.626110000	-0.250431000	4.723179000
C	2.090018000	-0.005822000	4.229860000
H	2.446854000	-0.467181000	5.164816000
H	1.643339000	-0.795256000	3.606109000
H	2.964185000	0.385546000	3.688567000
C	3.814812000	6.638825000	4.747495000
H	4.552603000	5.957885000	4.298488000
H	3.859735000	7.591961000	4.201384000
H	4.120938000	6.837966000	5.786405000
C	1.391473000	7.029407000	5.299465000
H	1.610774000	7.181778000	6.368523000
H	1.435449000	8.009621000	4.799867000
H	0.358751000	6.658153000	5.227496000

102
TStED-wB97XD

Ni	1.151243000	2.802287000	0.815714000
P	2.719453000	4.132215000	-0.406325000
N	-0.627100000	3.583273000	1.110111000
N	1.224260000	3.193894000	3.009233000
C	-1.029232000	3.569635000	2.413513000
C	-2.409619000	3.830373000	2.485300000
H	-3.017181000	3.883213000	3.387416000
C	-2.831409000	4.020812000	1.165875000
H	-3.841227000	4.246710000	0.831732000
C	-1.689847000	3.872612000	0.340280000
C	0.001427000	3.421911000	3.382720000
H	-0.235193000	3.567484000	4.447648000
C	-1.462789000	4.125558000	-1.098736000
C	-1.961075000	5.297525000	-1.709892000
C	-1.585416000	5.595174000	-3.023548000
H	-1.961766000	6.508839000	-3.490956000
C	-0.728029000	4.758131000	-3.730168000
H	-0.437390000	5.005324000	-4.754021000
C	-0.240365000	3.605510000	-3.130975000
H	0.424477000	2.942485000	-3.689439000
C	-0.594304000	3.261283000	-1.817617000
C	-2.850131000	6.271304000	-0.976688000
H	-2.491682000	6.460827000	0.046023000
H	-3.880057000	5.893011000	-0.883613000
H	-2.899347000	7.228640000	-1.514592000
C	-0.115391000	1.980961000	-1.249571000
H	-0.968977000	1.380128000	-0.897534000
H	0.481603000	1.438224000	0.077392000
C	2.247636000	3.334167000	3.987585000
C	2.687106000	2.232190000	4.744820000
C	3.709481000	2.437315000	5.678253000
H	4.054623000	1.600049000	6.288153000
C	4.292525000	3.686828000	5.849456000
H	5.088355000	3.824121000	6.584861000
C	3.862075000	4.761262000	5.077125000
H	4.333361000	5.737164000	5.206450000
C	2.842225000	4.608036000	4.137078000
C	2.040022000	0.866169000	4.594601000
H	1.538305000	0.851992000	3.618169000
C	2.378400000	5.774608000	3.279553000
H	1.981914000	5.339925000	2.350169000
C	4.438814000	3.914810000	0.186082000
C	4.626018000	3.482710000	1.501758000
H	3.764551000	3.249516000	2.125460000
C	5.907650000	3.342474000	2.029707000
H	6.025272000	3.015250000	3.064895000
C	7.017309000	3.615332000	1.233070000
H	8.024827000	3.503196000	1.639753000
C	6.841751000	4.018362000	-0.092243000
H	7.710642000	4.215979000	-0.723736000
C	5.559897000	4.164580000	-0.617092000
H	5.431532000	4.462654000	-1.660780000
C	2.405519000	5.933359000	-0.338090000
C	3.403366000	6.883536000	-0.588330000
H	4.427830000	6.565312000	-0.791696000
C	3.099035000	8.242026000	-0.567497000
H	3.883776000	8.976127000	-0.761694000
C	1.798343000	8.663207000	-0.287204000
H	1.562286000	9.729457000	-0.266958000
C	0.804015000	7.722322000	-0.027245000
H	-0.215435000	8.042740000	0.196650000
C	1.104569000	6.361338000	-0.051816000
H	0.325117000	5.628810000	0.164109000
C	2.884017000	3.775583000	-2.195301000
C	3.105657000	2.444398000	-2.572113000
H	3.157662000	1.662446000	-1.810909000
C	3.247475000	2.107875000	-3.914747000
H	3.416819000	1.066518000	-4.196330000
C	3.166662000	3.097087000	-4.896534000
H	3.274063000	2.832317000	-5.950663000
C	2.942641000	4.420883000	-4.527209000
H	2.867722000	5.198647000	-5.290100000
C	2.798259000	4.760594000	-3.181923000
H	2.597697000	5.797555000	-2.908220000
H	0.477576000	1.397892000	-1.967770000
C	0.625314000	0.712053000	1.146754000
C	1.686482000	-0.187870000	1.226148000
C	-0.584415000	0.328708000	1.740663000
C	1.585483000	-1.425320000	1.853138000
C	-0.707381000	-0.888784000	2.405824000
C	0.374719000	-1.769232000	2.451610000
Cl	3.245797000	0.295214000	0.565410000
H	-1.440042000	1.006896000	1.673084000
H	2.444833000	-2.096167000	1.889771000
H	-1.654088000	-1.161436000	2.876732000
H	0.279542000	-2.733239000	2.956144000
C	0.957866000	0.653171000	5.659466000
H	1.390264000	0.668978000	6.673187000
H	0.188845000	1.438307000	5.611682000

H	0.457945000	-0.316879000	5.511762000
C	3.059047000	-0.274487000	4.607472000
H	3.594944000	-0.343703000	5.566980000
H	2.549901000	-1.236228000	4.452027000
H	3.803221000	-0.149484000	3.806146000
C	3.504576000	6.733403000	2.894247000
H	4.347136000	6.201710000	2.426637000
H	3.129509000	7.475964000	2.175184000
H	3.888759000	7.288303000	3.764971000
C	1.226770000	6.543251000	3.938438000
H	1.551635000	6.989501000	4.892159000
H	0.883726000	7.354299000	3.277170000
H	0.363271000	5.895920000	4.145945000

102

tD+PhCl-wB97XD

Ni	0.844705000	2.077285000	1.596407000
P	2.447655000	0.438062000	1.082143000
N	-0.078019000	3.672509000	1.038759000
N	1.300955000	3.378388000	3.205670000
C	-0.168977000	4.692947000	1.930766000
C	-1.218710000	5.550196000	1.528932000
H	-1.547677000	6.460995000	2.026828000
C	-1.775178000	4.971106000	0.380047000
H	-2.621996000	5.346203000	-0.191016000
C	-1.050149000	3.777117000	0.132816000
C	0.644107000	4.503810000	3.081193000
H	0.698127000	5.268558000	3.867407000
C	-1.185018000	2.600306000	-0.755394000
C	-1.286449000	2.721224000	-2.153044000
C	-1.239619000	1.567507000	-2.942957000
H	-1.304317000	1.659038000	-4.030353000
C	-1.096287000	0.310437000	-2.356645000
H	-1.051309000	-0.582237000	-2.985279000
C	-1.015566000	0.186770000	-0.975941000
H	-0.913900000	-0.803171000	-0.522663000
C	-1.070705000	1.314826000	-0.132649000
C	-1.408203000	4.077443000	-2.799241000
H	-1.262965000	4.012464000	-3.886702000
H	-0.671043000	4.784049000	-2.388843000
H	-2.402542000	4.516175000	-2.621504000
C	-0.947050000	1.136898000	1.335651000
H	-1.035865000	0.081799000	1.626157000
H	-1.641325000	1.762523000	1.914360000
C	2.200251000	3.205046000	4.282329000
C	2.052017000	2.051502000	5.090526000
C	2.933187000	1.862711000	6.153763000
H	2.833634000	0.982876000	6.790348000
C	3.939944000	2.784507000	6.427773000
H	4.611248000	2.627270000	7.274729000
C	4.095739000	3.892598000	5.608717000
H	4.903509000	4.599776000	5.813594000
C	3.255564000	4.117640000	4.511795000
C	0.922612000	1.070466000	4.818670000
H	0.839109000	0.979763000	3.721600000
C	3.551918000	5.300646000	3.596999000
H	2.908757000	5.221400000	2.710841000
C	3.828763000	0.284346000	2.261706000
C	4.469703000	1.472473000	2.636140000
H	4.144573000	2.424492000	2.206703000
C	5.505959000	1.450377000	3.562239000
H	5.988127000	2.384860000	3.853675000
C	5.896835000	0.242097000	4.139707000
H	6.698049000	0.226300000	4.881571000
C	5.259451000	-0.942261000	3.776346000
H	5.563899000	-1.889407000	4.226805000
C	4.230090000	-0.925744000	2.834338000
H	3.736433000	-1.858110000	2.551790000
C	3.232663000	0.759617000	-0.537852000
C	4.576579000	0.466537000	-0.796646000
H	5.207502000	0.052215000	-0.006707000
C	5.113234000	0.709056000	-2.060416000
H	6.163663000	0.482400000	-2.255035000
C	4.312191000	1.239775000	-3.072075000
H	4.736366000	1.431828000	-4.059944000
C	2.972253000	1.531459000	-2.818979000
H	2.337699000	1.952021000	-3.601254000
C	2.433598000	1.297659000	-1.556126000
H	1.382042000	1.535971000	-1.371141000
C	1.757777000	-1.243482000	0.904502000
C	0.952426000	-1.730247000	1.941941000
H	0.785028000	-1.117318000	2.829009000
C	0.356162000	-2.983645000	1.844442000
H	-0.269358000	-3.351170000	2.660458000
C	0.545133000	-3.757611000	0.698856000
H	0.067656000	-4.736195000	0.614760000
C	1.340282000	-3.276898000	-0.339565000
H	1.488666000	-3.877575000	-1.239340000
C	1.948163000	-2.025734000	-0.238433000
H	2.562318000	-1.655179000	-1.061361000
H	1.515069000	6.021024000	0.436417000
C	2.541416000	5.827686000	0.119120000
C	3.484302000	6.852284000	0.099101000
C	2.935096000	4.544109000	-0.256547000
C	4.797722000	6.621351000	-0.307878000
C	4.247911000	4.289739000	-0.650720000
C	5.173783000	5.332425000	-0.681475000
Cl	3.021968000	8.454118000	0.620880000
H	2.194377000	3.743750000	-0.229701000
H	5.515243000	7.443131000	-0.319033000
H	4.545165000	3.280690000	-0.941319000
H	6.202638000	5.143222000	-0.995101000
C	-0.423131000	1.615399000	5.311687000
H	-0.412369000	1.743665000	6.405880000
H	-0.654130000	2.586777000	4.853621000
H	-1.238831000	0.922515000	5.053200000
C	1.178247000	-0.330125000	5.372574000
H	1.175340000	-0.344320000	6.473777000
H	0.379765000	-1.015550000	5.048464000
H	2.142663000	-0.733040000	5.026174000
C	4.993117000	5.277261000	3.075792000
H	5.204622000	4.357767000	2.510191000
H	5.160035000	6.123830000	2.394345000
H	5.729255000	5.356040000	3.891245000
C	3.248457000	6.644702000	4.269097000
H	3.920772000	6.821851000	5.124099000
H	3.380202000	7.469180000	3.551818000
H	2.217426000	6.688035000	4.650769000

90

tD-wB97XD

Ni	0.862466000	2.016065000	1.602611000
P	2.460887000	0.358269000	1.146163000
N	-0.055679000	3.603228000	1.005249000
N	1.312544000	3.332951000	3.191254000
C	-0.144960000	4.635673000	1.886052000
C	-1.227363000	5.462852000	1.504971000
H	-1.563583000	6.371645000	2.002177000
C	-1.805527000	4.854697000	0.384183000
H	-2.678795000	5.199921000	-0.165796000
C	-1.059725000	3.673989000	0.131201000
C	0.657836000	4.459123000	3.043364000
H	0.701237000	5.230961000	3.824213000
C	-1.210811000	2.479466000	-0.730815000
C	-1.351311000	2.570062000	-2.127143000
C	-1.305802000	1.401247000	-2.894479000
H	-1.397370000	1.469299000	-3.981707000
C	-1.129954000	0.158872000	-2.286280000
H	-1.085410000	-0.745903000	-2.897535000
C	-1.021780000	0.064133000	-0.905281000
H	-0.902856000	-0.915456000	-0.434164000
C	-1.073661000	1.208681000	-0.084798000
C	-1.518154000	3.909807000	-2.797530000
H	-1.387417000	3.827410000	-3.885784000
H	-0.796242000	4.645008000	-2.411567000
H	-2.522057000	4.324002000	-2.613470000
C	-0.928006000	1.066446000	1.385041000
H	-1.011252000	0.018760000	1.702713000
H	-1.616780000	1.704217000	1.957237000
C	2.197658000	3.167592000	4.278900000
C	2.030592000	2.029127000	5.105400000
C	2.898150000	1.850553000	6.181449000
H	2.781312000	0.983524000	6.832594000
C	3.915764000	2.762699000	6.447354000
H	4.577888000	2.611858000	7.302609000
C	4.095277000	3.851797000	5.607688000
H	4.912905000	4.549941000	5.805258000
C	3.264140000	4.068730000	4.502022000
C	0.900240000	1.049628000	4.833067000
H	0.825546000	0.950130000	3.736653000
C	3.582982000	5.233075000	3.569660000
H	2.929700000	5.159410000	2.689028000
C	3.826926000	0.140962000	2.334868000
C	4.472664000	1.304457000	2.770959000
H	4.154366000	2.276093000	2.385628000
C	5.500609000	1.234333000	3.703872000
H	5.984424000	2.152127000	4.042572000
C	5.880265000	-0.002175000	4.226543000
H	6.675824000	-0.058648000	4.972557000
C	5.238928000	-1.164048000	3.801730000
H	5.534874000	-2.133412000	4.208521000
C	4.217341000	-1.097054000	2.853465000
H	3.721118000	-2.012085000	2.522964000
C	3.261777000	0.723407000	-0.455557000
C	4.599310000	0.411619000	-0.723307000
H	5.220236000	-0.043408000	0.052286000
C	5.141311000	0.683062000	-1.978441000
H	6.186950000	0.441215000	-2.180716000
C	4.350861000	1.258994000	-2.973120000
H	4.778126000	1.468588000	-3.956266000
C	3.017406000	1.571579000	-2.710609000
H	2.391043000	2.024146000	-3.481783000
C	2.474214000	1.314085000	-1.454183000
H	1.426780000	1.566949000	-1.262253000
C	1.750939000	-1.308281000	0.916385000
C	0.950067000	-1.822023000	1.944441000
H	0.804047000	-1.241847000	2.857470000
C	0.330872000	-3.060177000	1.804873000
H	-0.292013000	-3.449101000	2.612949000
C	0.493513000	-3.791605000	0.627520000
H	-0.002305000	-4.757658000	0.510845000
C	1.285936000	-3.284553000	-0.400296000
H	1.413895000	-3.851895000	-1.324509000
C	1.915970000	-2.048396000	-0.257792000
H	2.525934000	-1.654701000	-1.073122000
C	-0.447182000	1.603249000	5.310841000
H	-0.446195000	1.738473000	6.404355000
H	-0.668885000	2.572810000	4.844122000
H	-1.263677000	0.912757000	5.048691000
C	1.148863000	-0.348069000	5.397466000
H	1.135725000	-0.358110000	6.498741000
H	0.352239000	-1.033431000	5.068568000
H	2.115866000	-0.754032000	5.061504000
C	5.023509000	5.167887000	3.046001000
H	5.230092000	4.209817000	2.546485000
H	5.200566000	5.970588000	2.313899000
H	5.760824000	5.291580000	3.854603000
C	3.312835000	6.586891000	4.236548000
H	3.963446000	6.732486000	5.113641000
H	3.503567000	7.413559000	3.534890000
H	2.272404000	6.668943000	4.584249000

Polyethylene Characterization by ^{1}H NMR - Determination of M_n and Degree of Branching

Determination of Polyethylene M_n by End-group Analysis: The $\boldsymbol{M_n}$ was determined from equation S1:[7]

$$\boldsymbol{M_n} = \frac{\left(\frac{I_{tot}}{4}\right)}{\left(\frac{I_2 + 2I_3 + 2I_4 + I_5 + I_6}{2}\right)} \times 28\ \text{g/mol} \tag{S1}$$

where I_2-I_6 are the integrals of the vinyl groups that can be found in a PE sample. In this case only the internal end-group protons (-C*H*=C*H*-), **B**, and the terminal end-group protons (CH_2=CH-), **A**, were found (Figure S17), thus only the I_2 and I_5 integrals were used in the calculation. The molecular weights measured by GPC/SEC (see below) were, on average, 1.4 times higher than those determined by ^{1}H NMR, a ratio lower ratio than typically reported in the literature.[7,8]

Determination of the Degree of Branching of Polyethylene: The degree of branching (***N***) was determined from equation S2:[7]

$$\boldsymbol{N} = \left[\frac{\boldsymbol{N_{branches}}}{1000} \times \frac{\boldsymbol{M_n}}{14\ \text{g}\,\text{mol}^{-1}} - 2\right] \times \frac{1000}{\boldsymbol{M_n}} \times 14\ \text{g}\,\text{mol}^{-1} \quad \text{(branches/1000C atoms)} \tag{S2}$$

which includes a correction, for low molecular weight oligomers (typically 10^4 g/mol), to the formerly reported equation S3,[8] in order to discount the two methyl terminal end-groups:

$$\boldsymbol{N_{branches}} = \frac{I_{CH_3}}{I_{tot}} \times \frac{2}{3} \times 1000 \quad \text{(branches/1000C atoms)} \tag{S3}$$

where I_{CH_3} represents the integral of the methyl protons ^{1}H NMR resonances and I_{tot} the overall integral of all proton resonances.

^{1}H NMR Spectra of Polyethylene Products

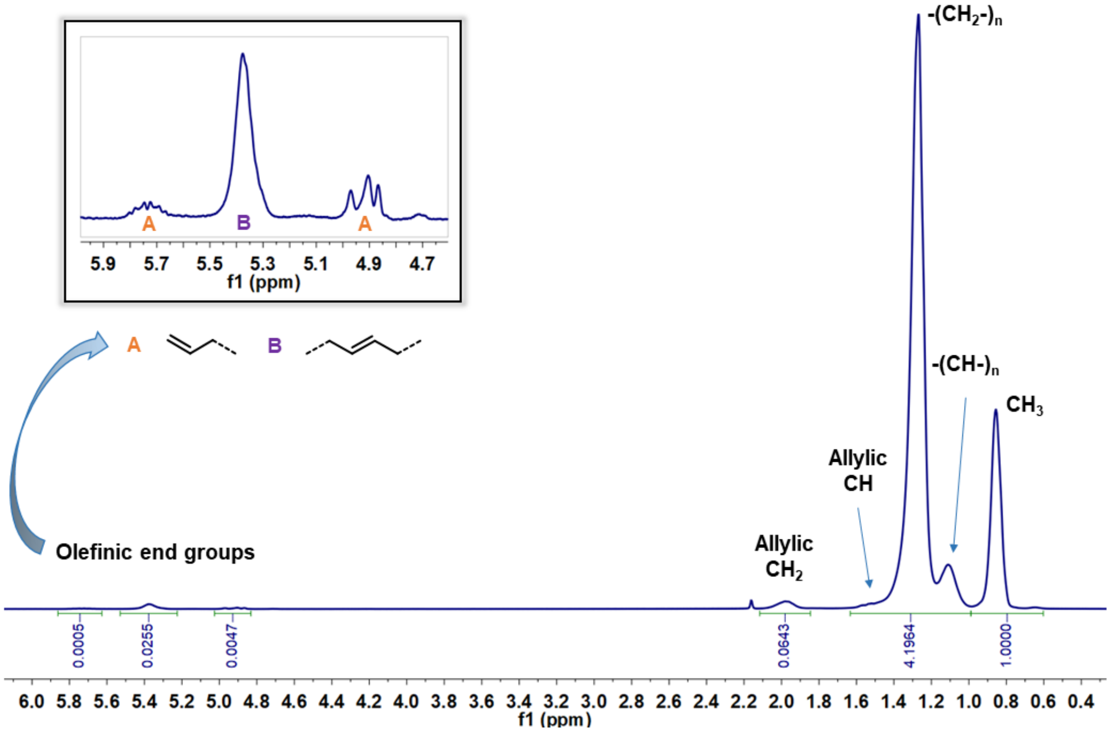

Figure S17 ^{1}H NMR spectrum (300 MHz, C_6D_6:1,2,4-TCB (1:2), 80 ºC) of the PE obtained using catalyst **1***, at 50 ºC and 9 bar of ethylene, and corresponding ^{1}H resonances assignments.

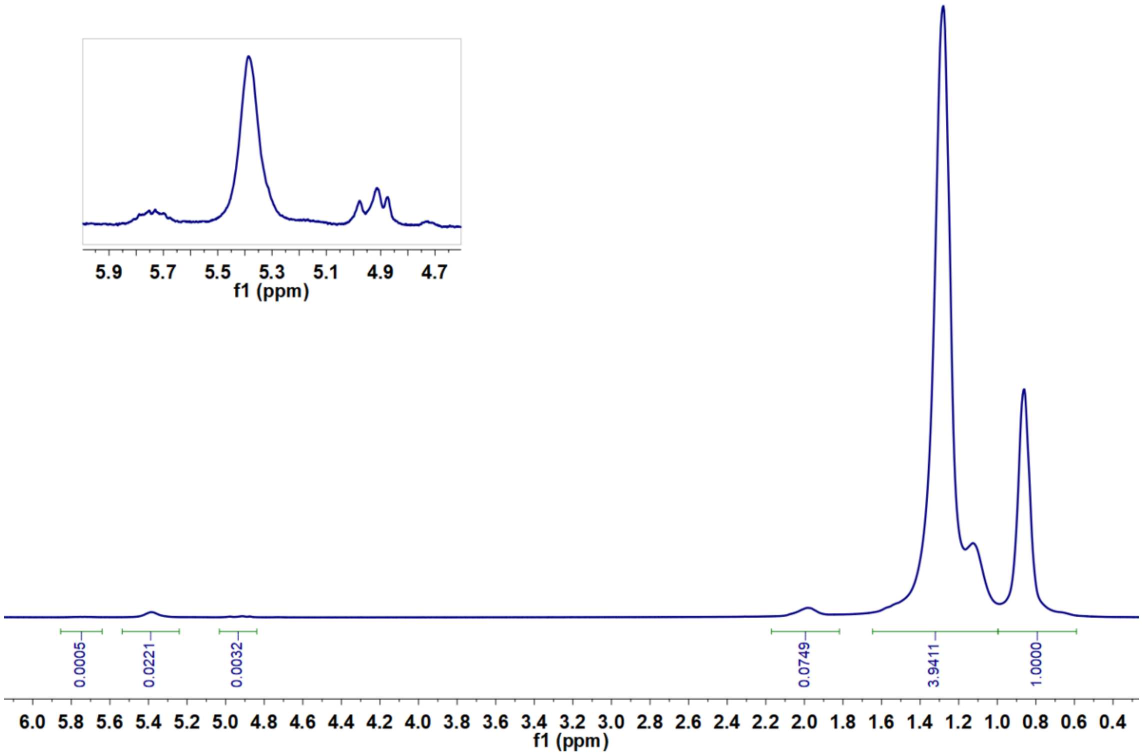

Figure S18 ^{1}H NMR spectrum (300 MHz, C_6D_6:1,2,4-TCB (1:2), 80 ºC) of the PE obtained using the catalyst **1***, at 25 ºC and 3 bar of ethylene.

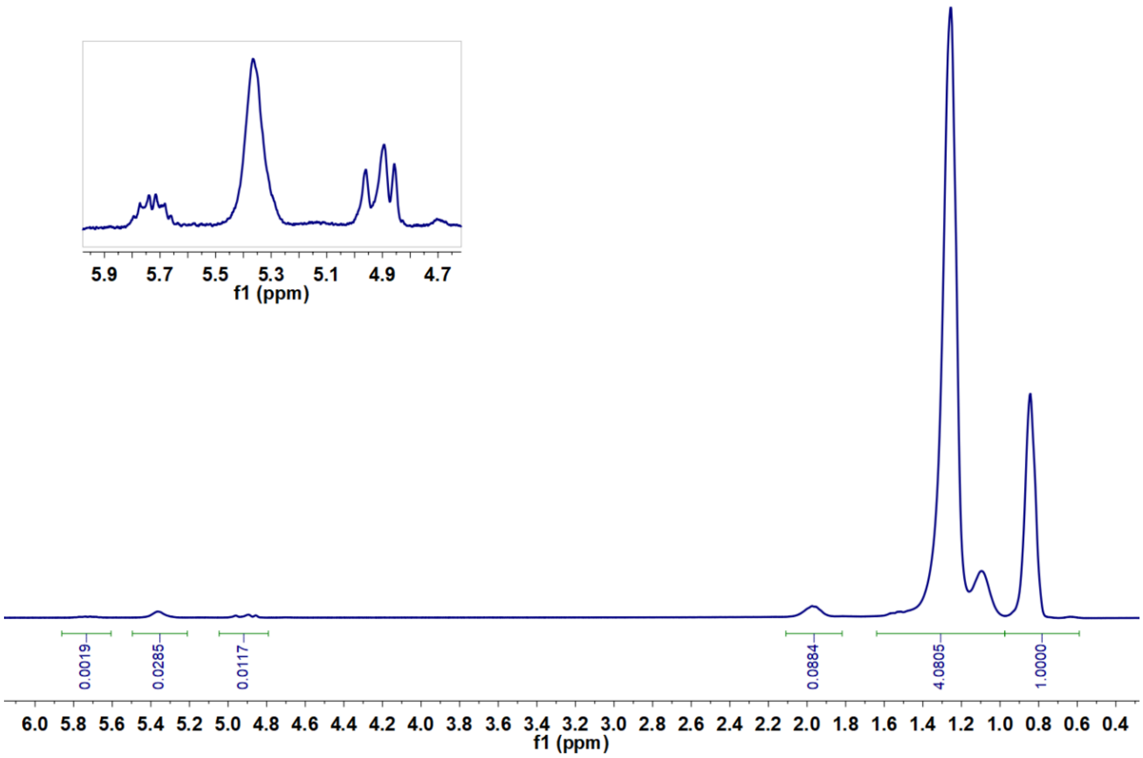

Figure S19 ^{1}H NMR spectrum (300 MHz, C_6D_6:1,2,4-TCB (1:2), 80 ºC) of the PE obtained using catalyst **1**, at 50 ºC and 9 bar of ethylene.

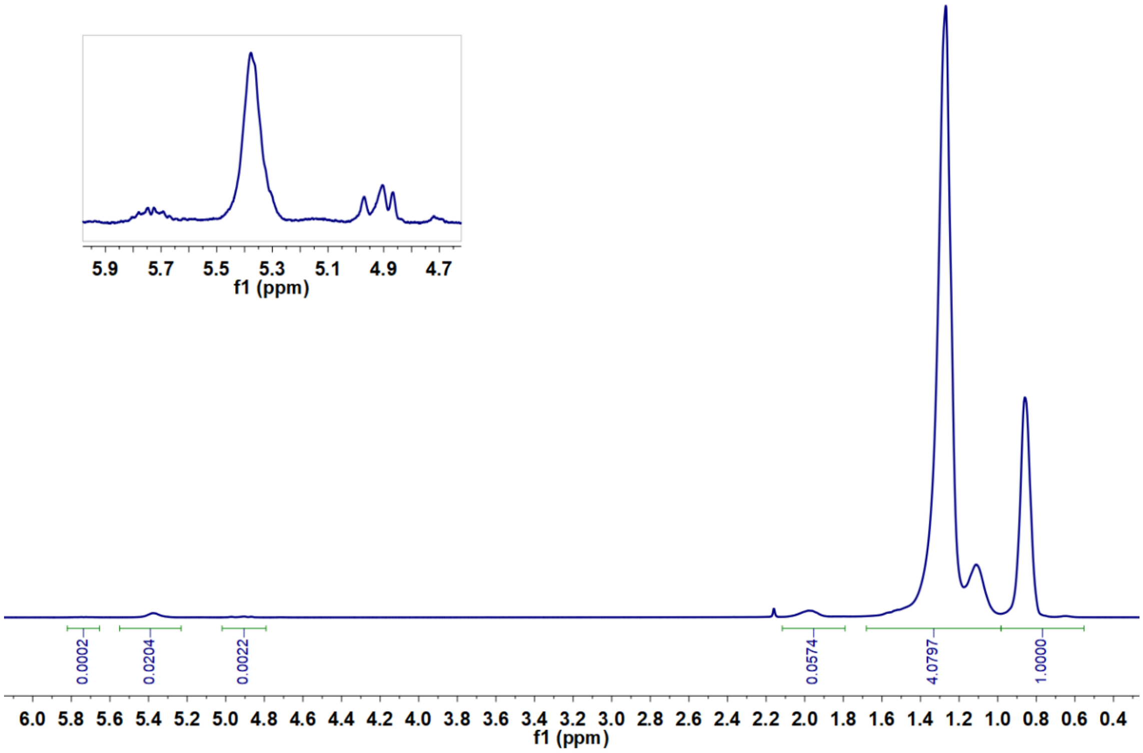

Figure S20 ^{1}H NMR spectrum (300 MHz, C_6D_6:1,2,4-TCB (1:2), 80 ºC) of the PE obtained using catalyst **1***, at 25 ºC and 9 bar of ethylene.

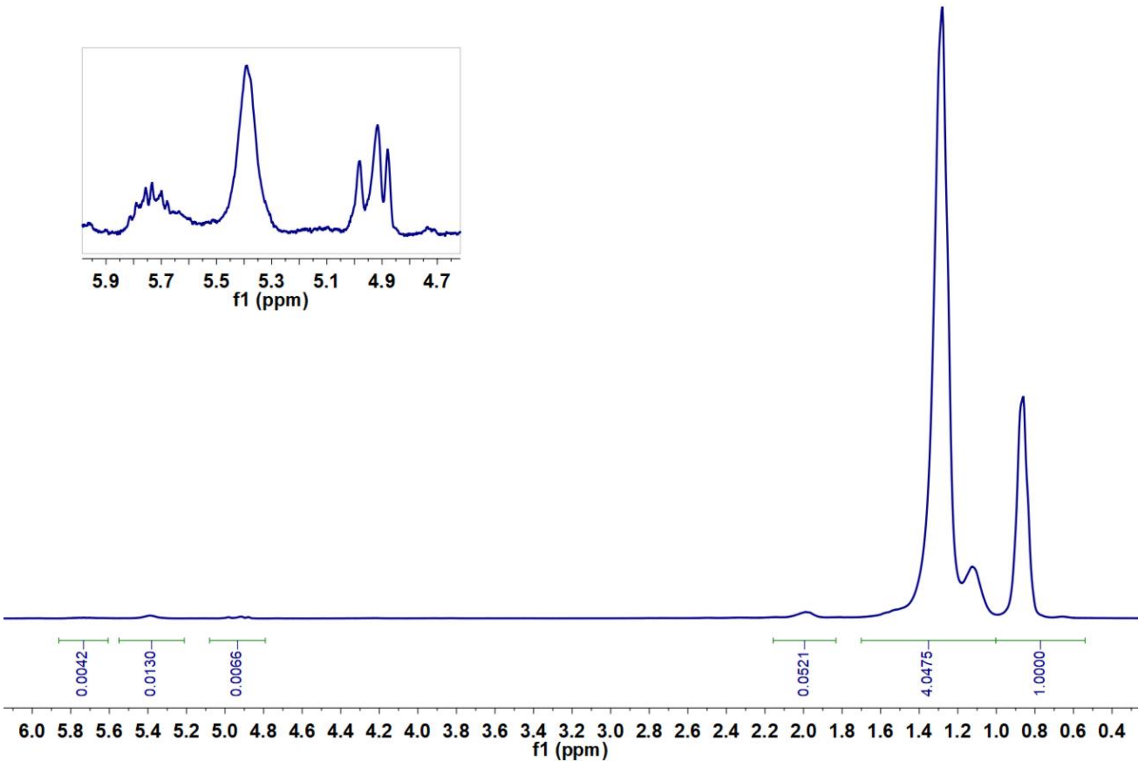

Figure S21 ^{1}H NMR spectrum (300 MHz, C_6D_6:1,2,4-TCB (1:2), 80 ºC) of the PE obtained using the catalyst **1**, at 50 ºC and 15 bar of ethylene.

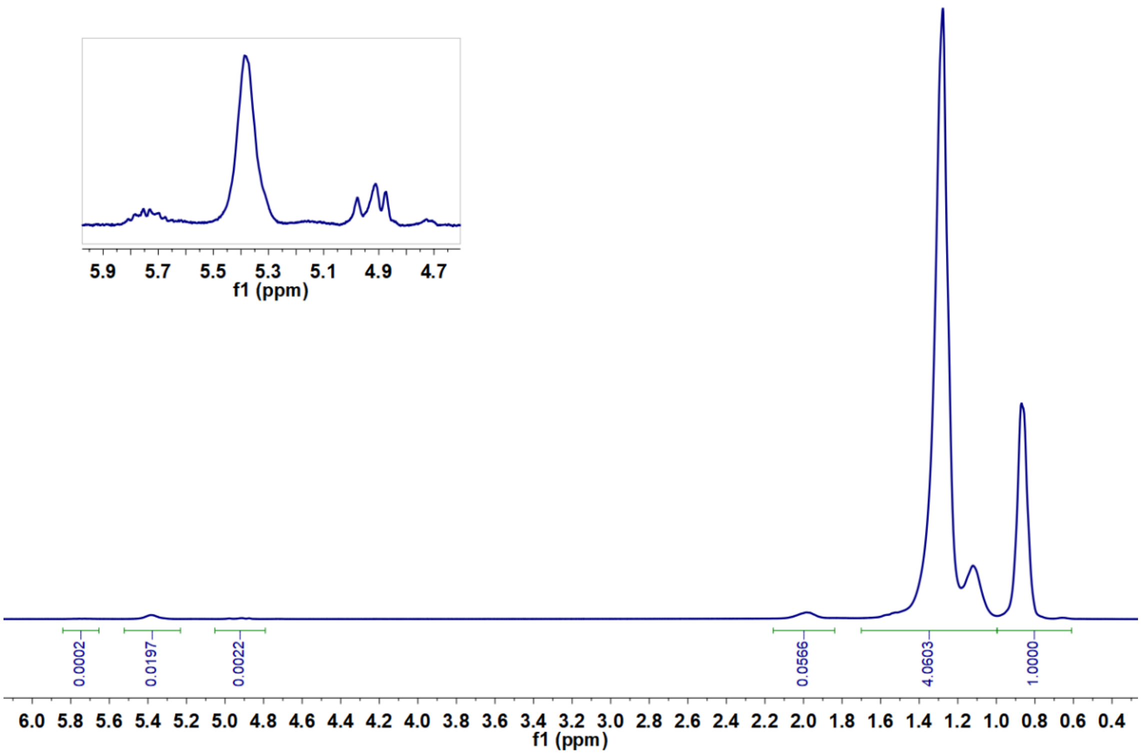

Figure S22 ^{1}H NMR spectrum (300 MHz, C_6D_6:1,2,4-TCB (1:2), 80 ºC) of the PE obtained using the catalyst **1***, at 25 ºC and 15 bar of ethylene.

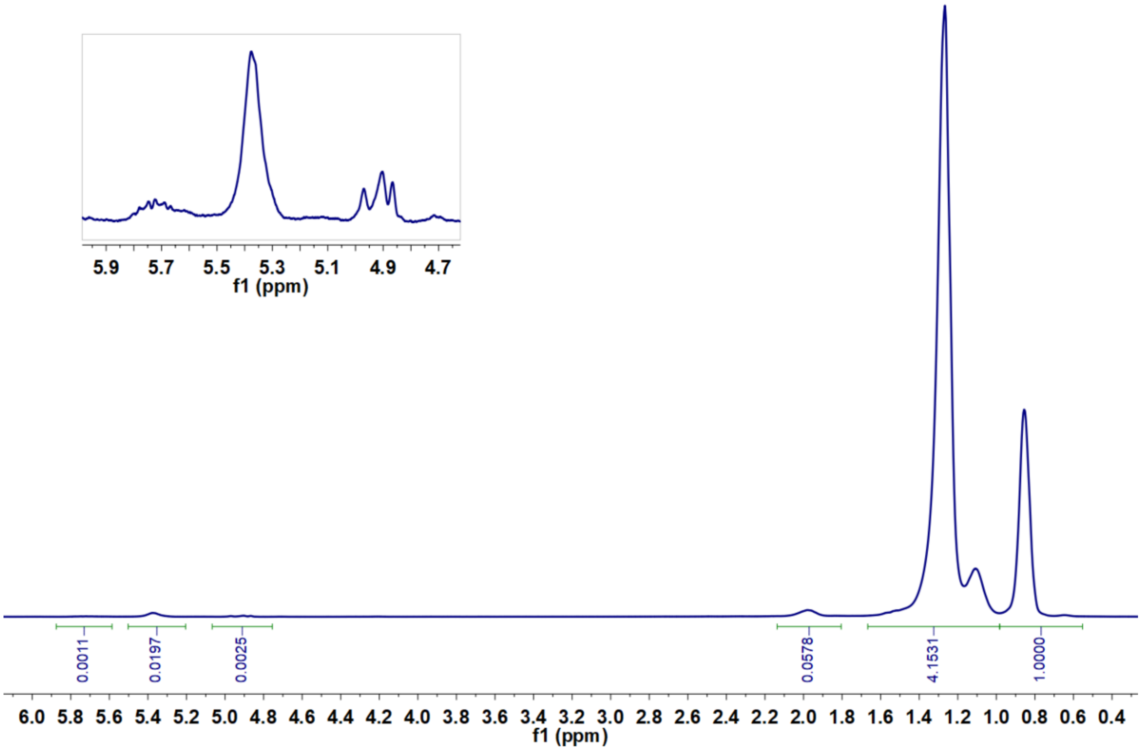

Figure S23 ^{1}H NMR spectrum (300 MHz, C_6D_6:1,2,4-TCB (1:2), 80 ºC) of the PE obtained using the catalyst **1***, at 50 ºC and 15 bar of ethylene.

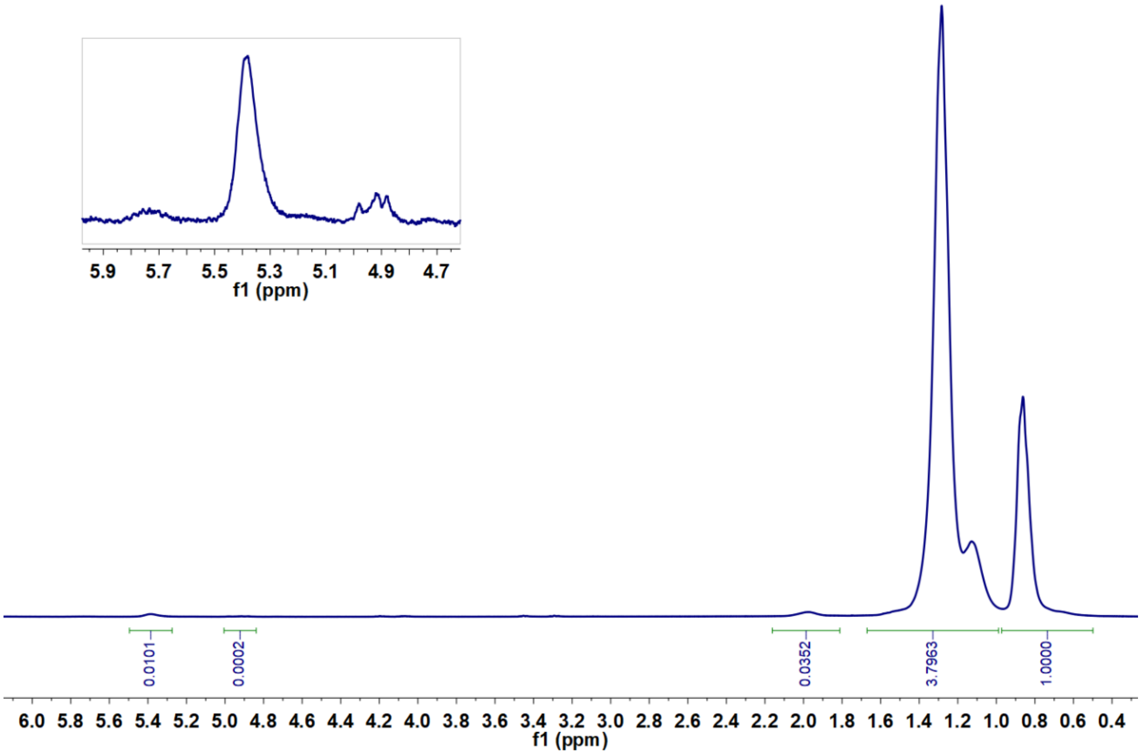

Figure S24 ^{1}H NMR spectrum (300 MHz, C_6D_6:1,2,4-TCB (1:2), 80 ºC) of the PE obtained using the catalyst **2**, at 25 ºC and 3 bar of ethylene.

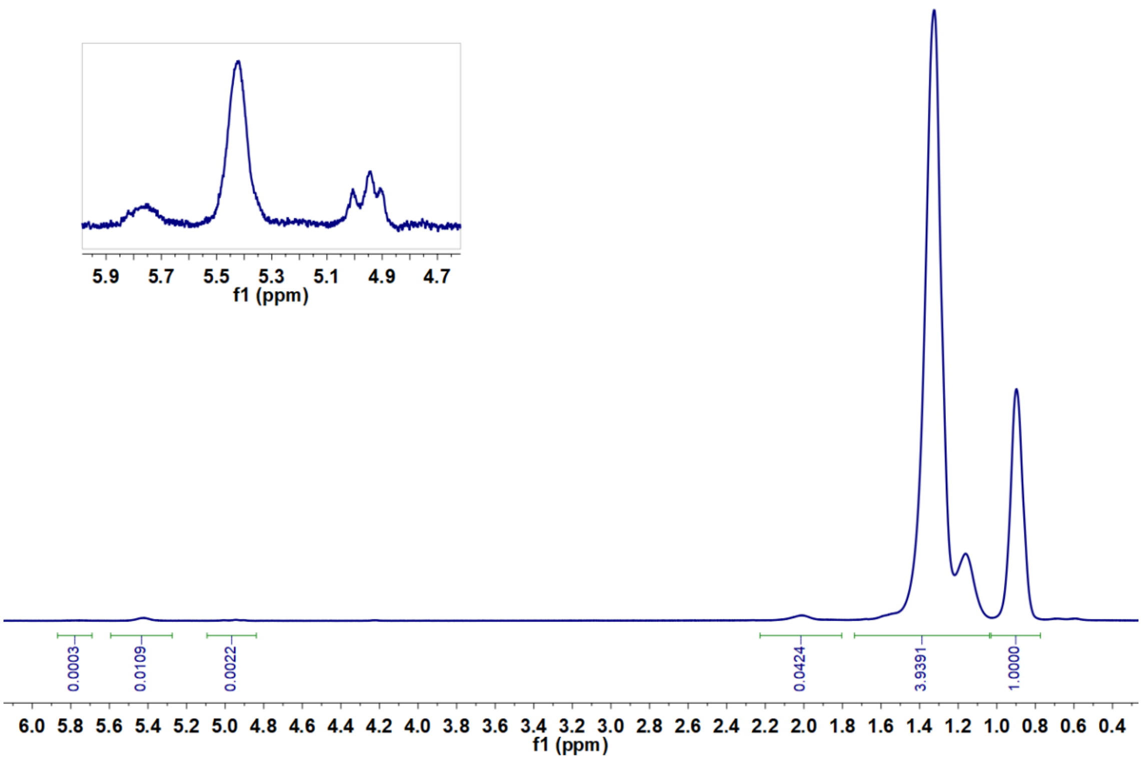

Figure S25 ^{1}H NMR spectrum (300 MHz, C_6D_6:1,2,4-TCB (1:2), 80 ºC) of the PE obtained using the catalyst **2**, at 50 ºC and 3 bar of ethylene.

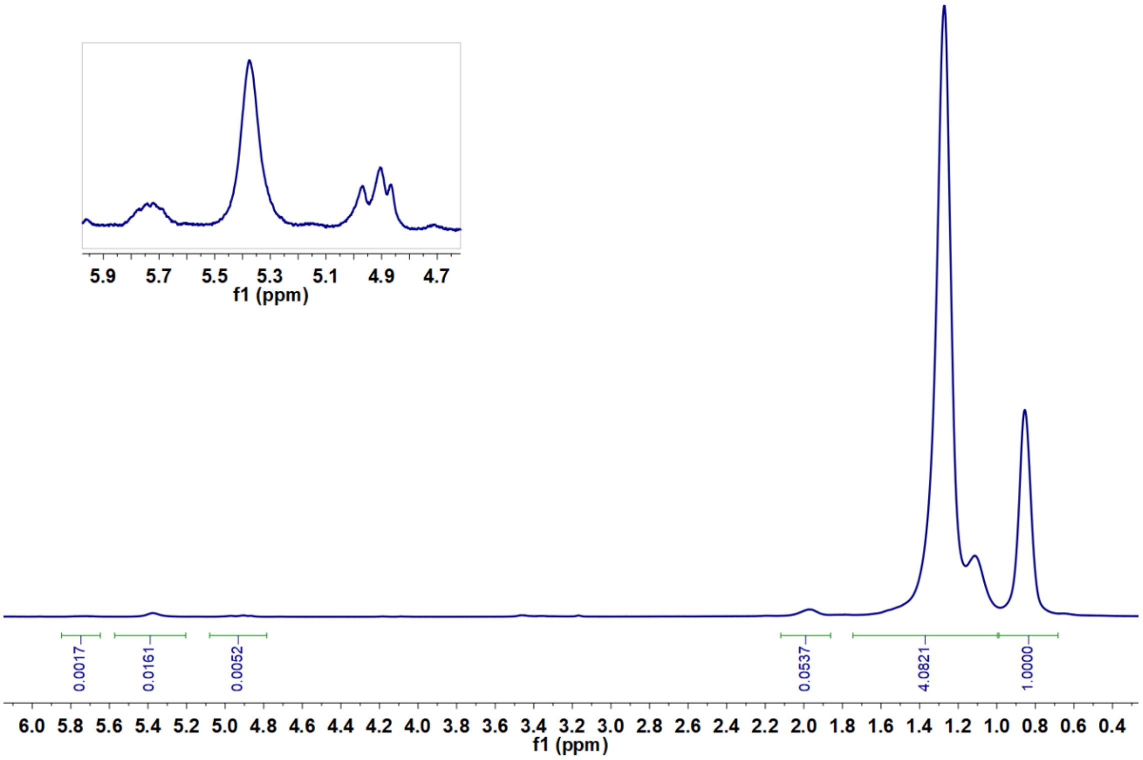

Figure S26 ^{1}H NMR spectrum (300 MHz, C_6D_6:1,2,4-TCB (1:2), 80 ºC) of the PE obtained using the catalyst **2***, at 25 ºC and 3 bar of ethylene.

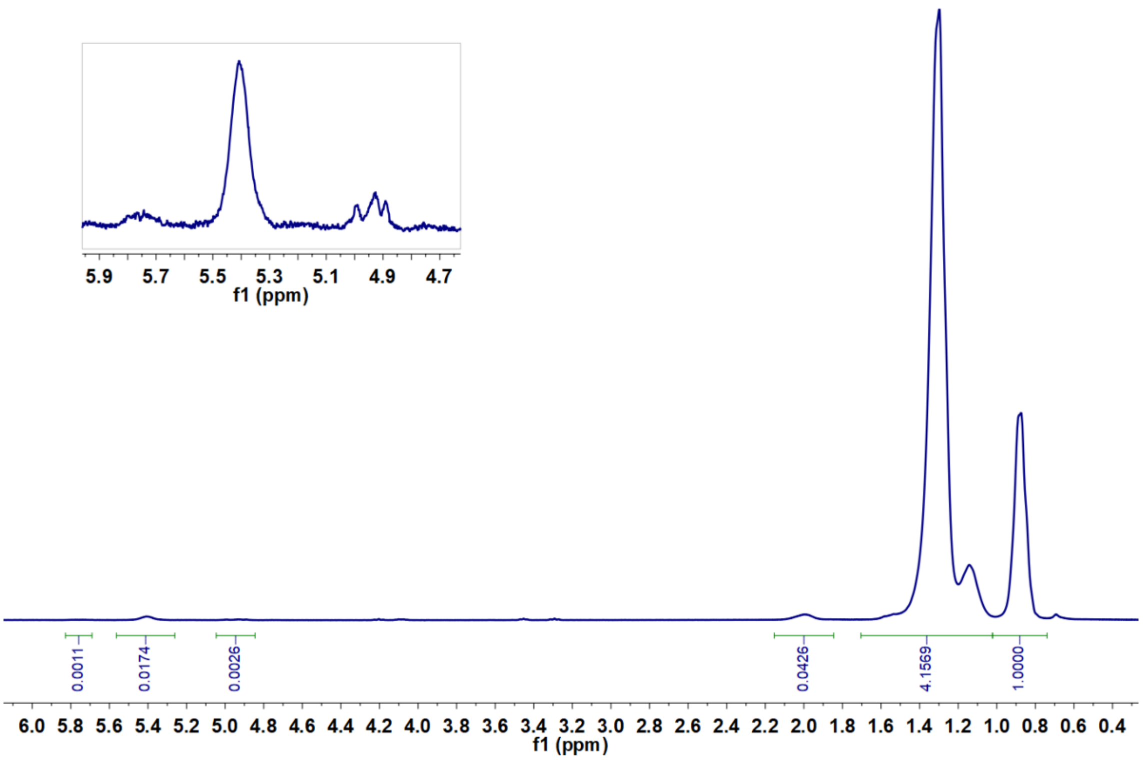

Figure S27 ^{1}H NMR spectrum (300 MHz, C_6D_6:1,2,4-TCB (1:2), 80 ºC) of the PE obtained using the catalyst **2***, at 50 ºC and 3 bar of ethylene.

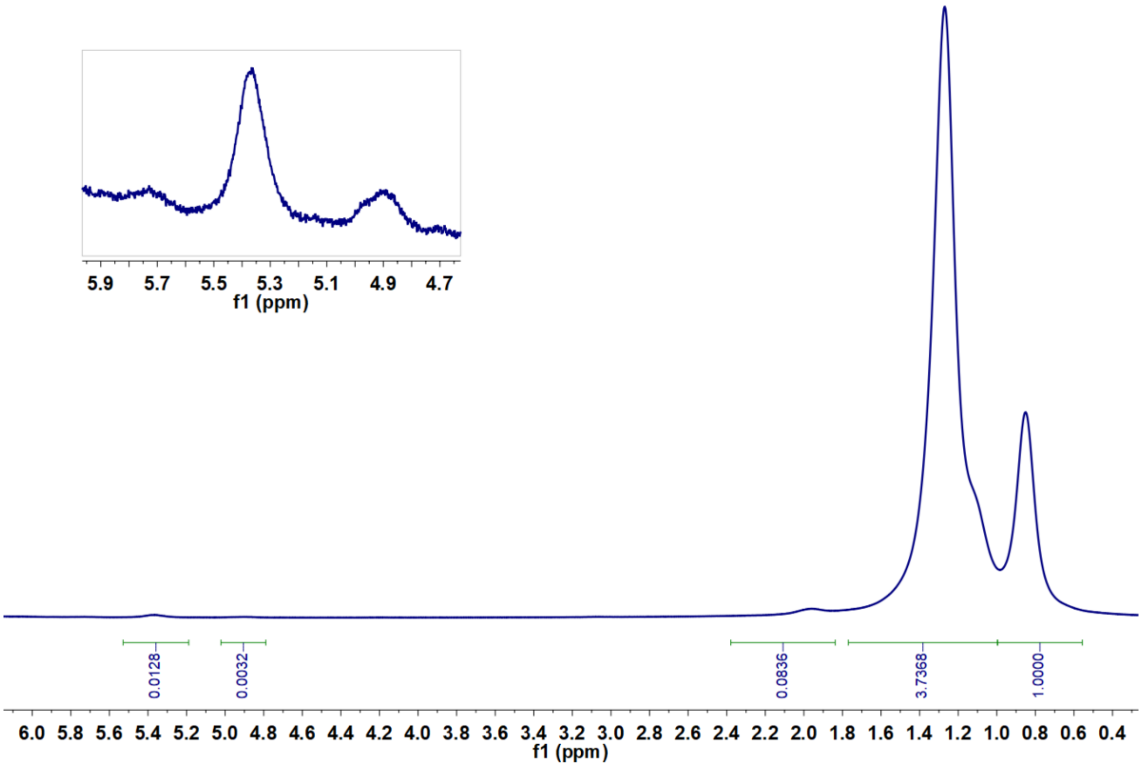

Figure S28 ^{1}H NMR spectrum (300 MHz, C_6D_6:1,2,4-TCB (1:2), 80 ºC) of the PE obtained using catalyst **2**, at 25 ºC and 9 bar of ethylene.

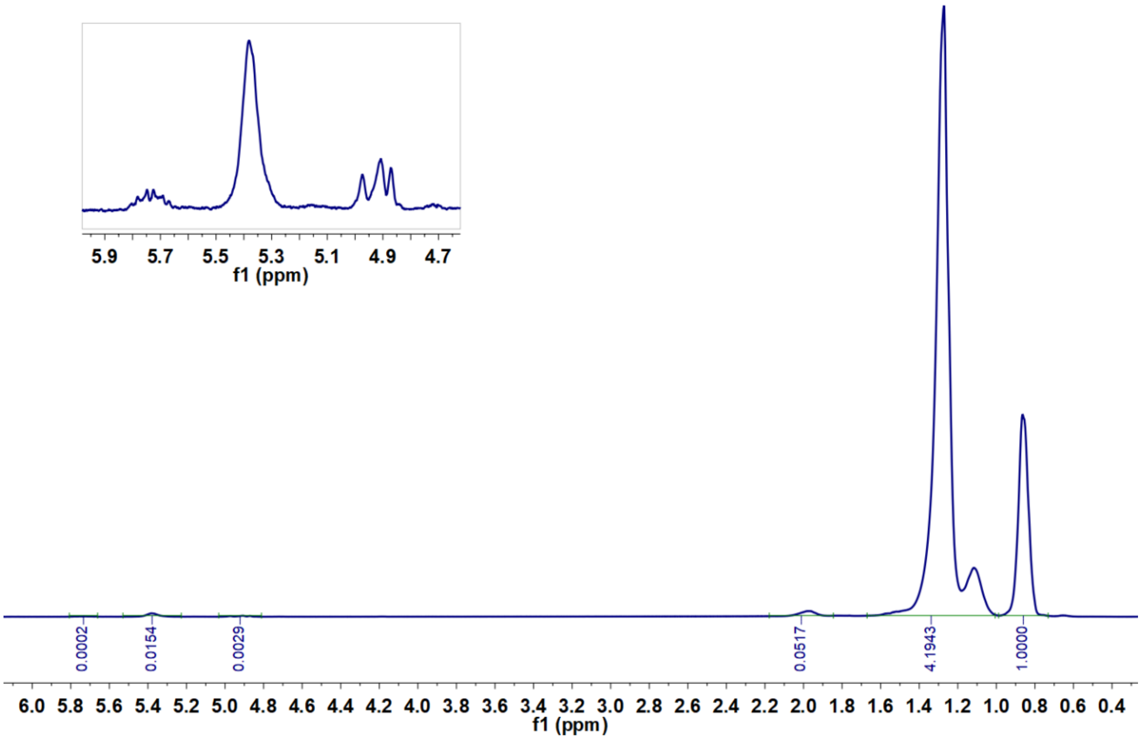

Figure S29 ^{1}H NMR spectrum (300 MHz, C_6D_6:1,2,4-TCB (1:2), 80 ºC) of the PE obtained using catalyst **2**, at 50 ºC and 9 bar of ethylene.

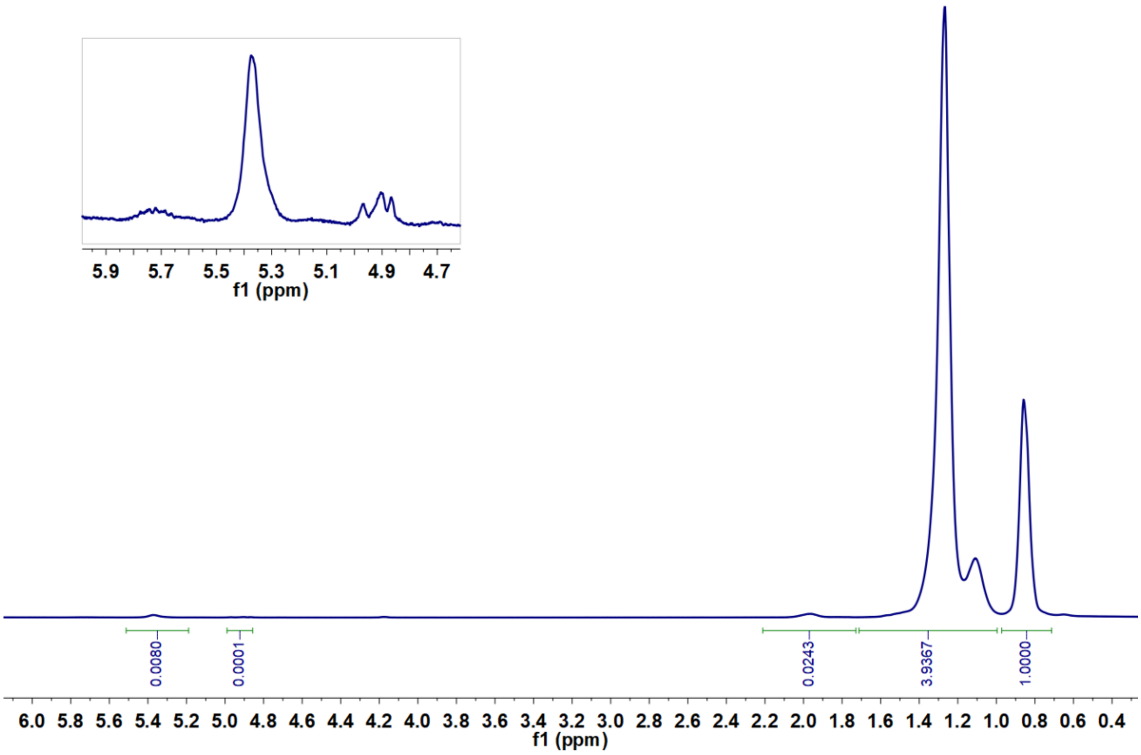

Figure S30 ^{1}H NMR spectrum (300 MHz, C_6D_6:1,2,4-TCB (1:2), 80 ºC) of the PE obtained using catalyst **2***, at 25 ºC and 9 bar of ethylene.

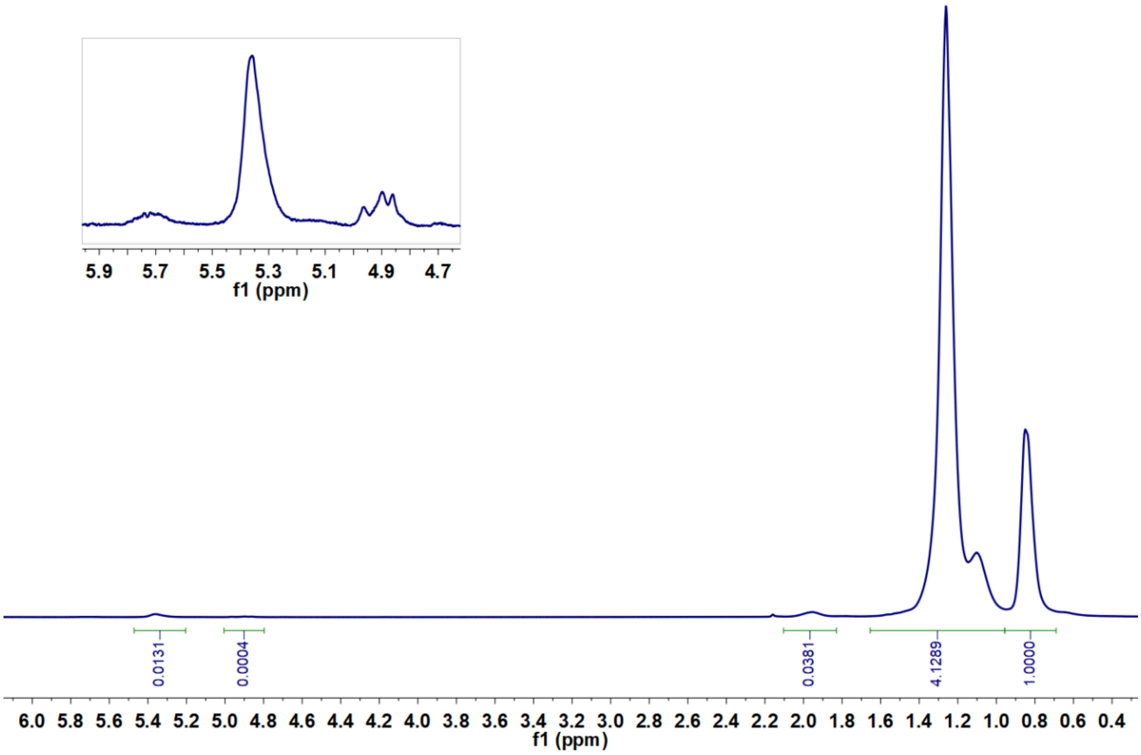

Figure S31 ^{1}H NMR spectrum (300 MHz, C_6D_6:1,2,4-TCB (1:2), 80 ºC) of the PE obtained using catalyst **2***, at 50 ºC and 9 bar of ethylene.

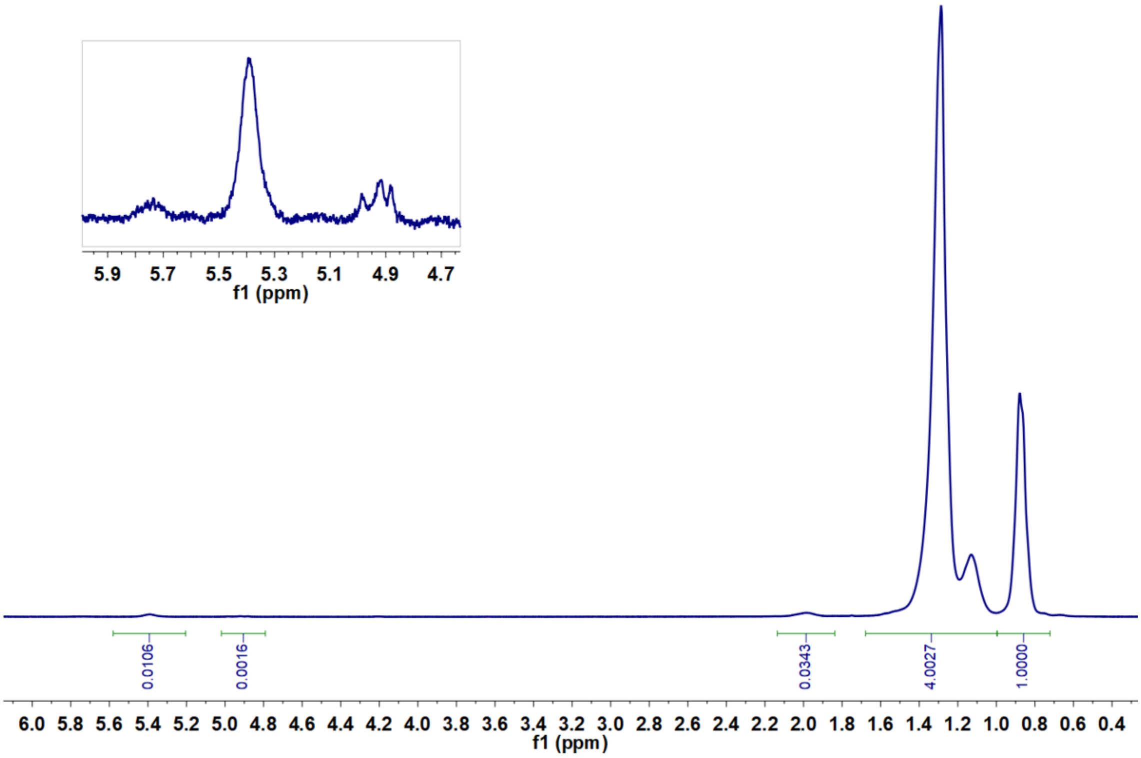

Figure S32 ^{1}H NMR spectrum (300 MHz, C_6D_6:1,2,4-TCB (1:2), 80 ºC) of the PE obtained using the catalyst **2**, at 25 ºC and 15 bar of ethylene.

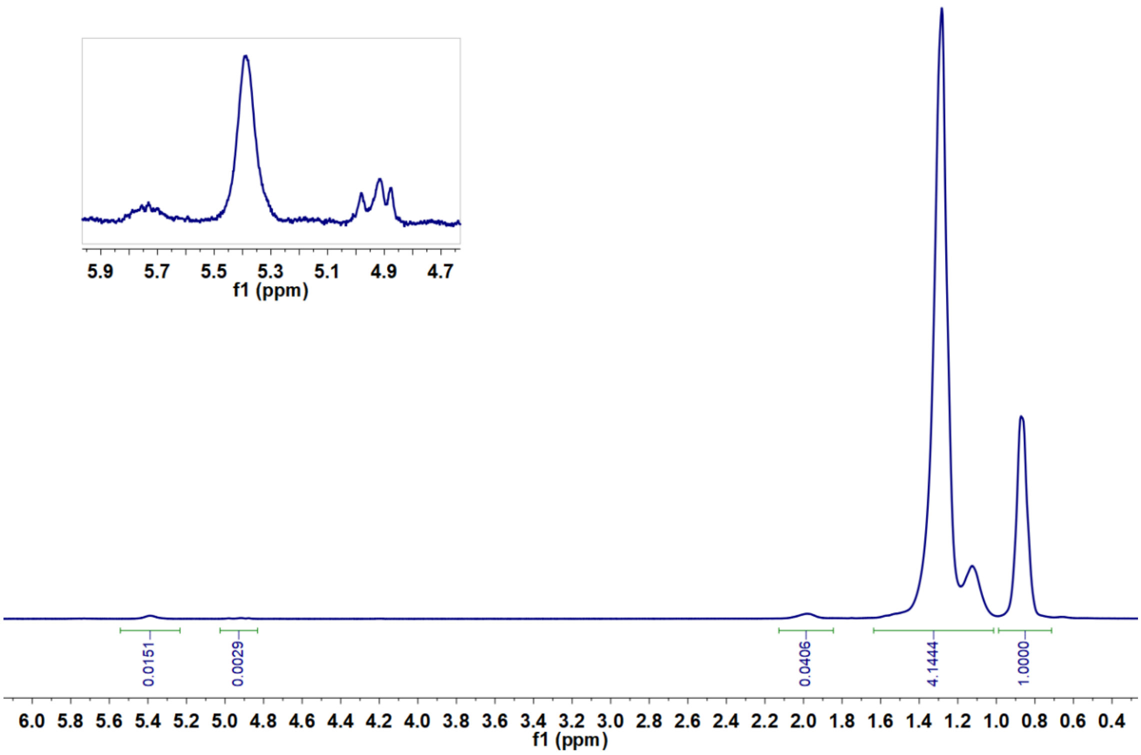

Figure S33 ^{1}H NMR spectrum (300 MHz, C_6D_6:1,2,4-TCB (1:2), 80 ºC) of the PE obtained using the catalyst **2**, at 50 ºC and 15 bar of ethylene.

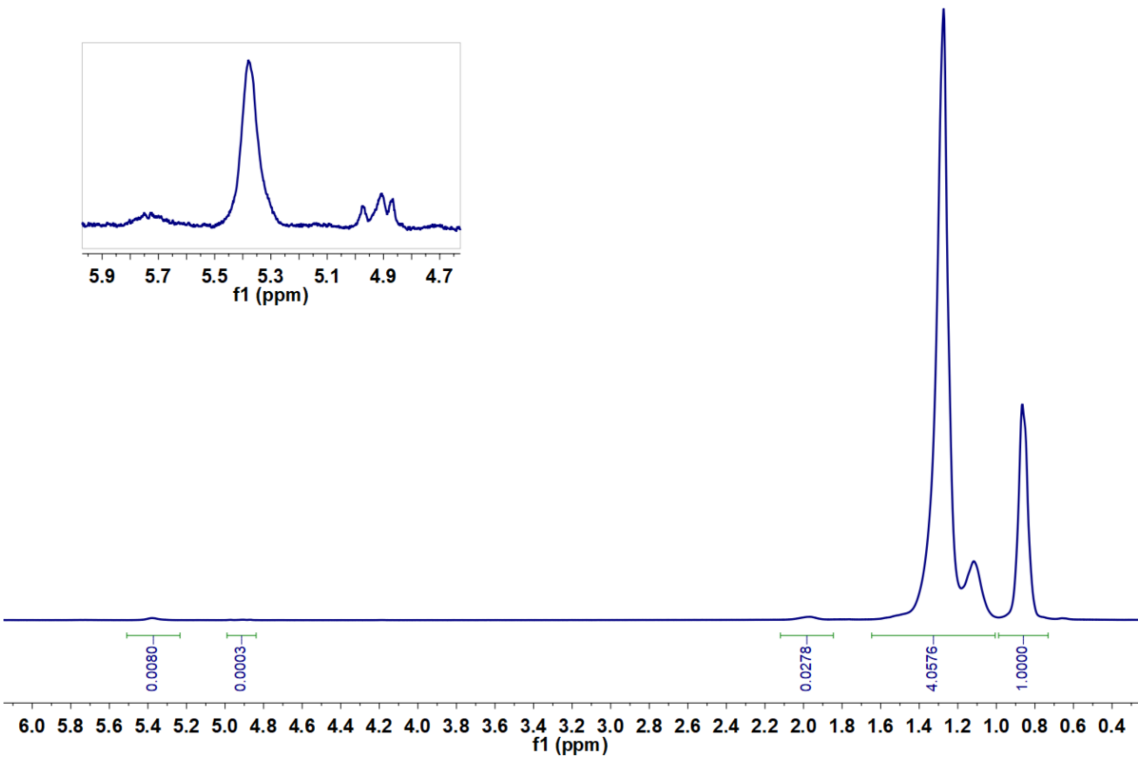

Figure S34 ^{1}H NMR spectrum (300 MHz, C_6D_6:1,2,4-TCB (1:2), 80 ºC) of the PE obtained using the catalyst **2***, at 25 ºC and 15 bar of ethylene.

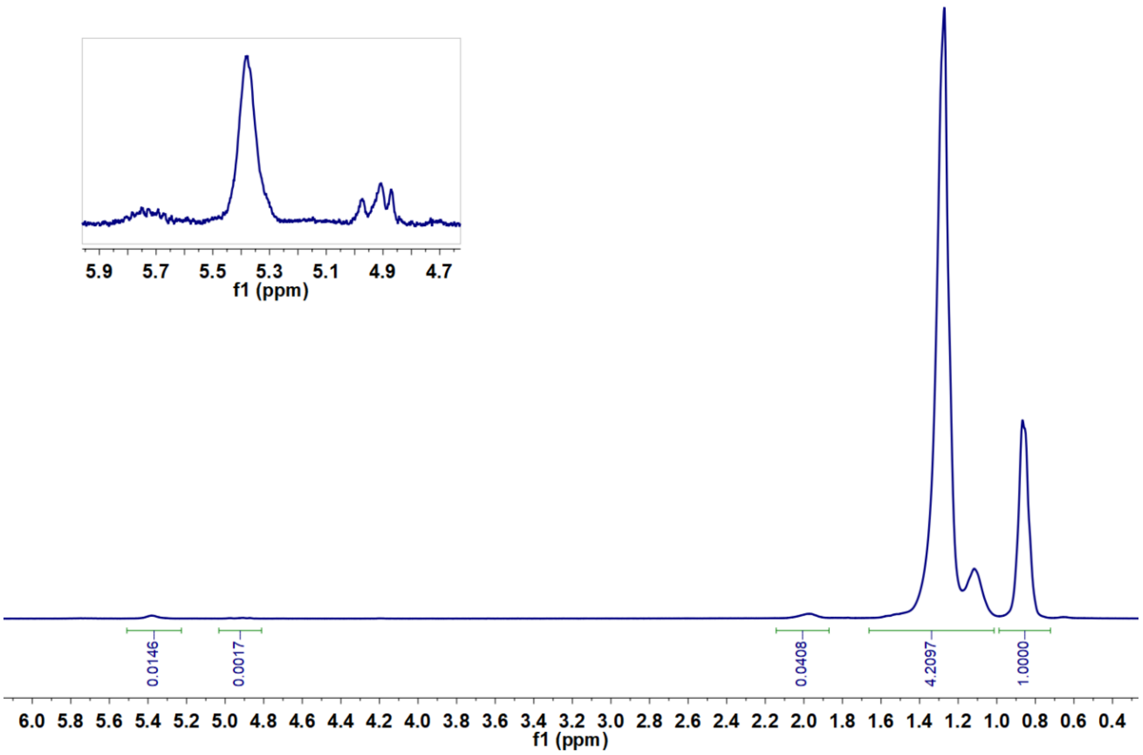

Figure S35 ^{1}H NMR spectrum (300 MHz, C_6D_6:1,2,4-TCB (1:2), 80 ºC) of the PE obtained using the catalyst **2***, at 50 ºC and 15 bar of ethylene.

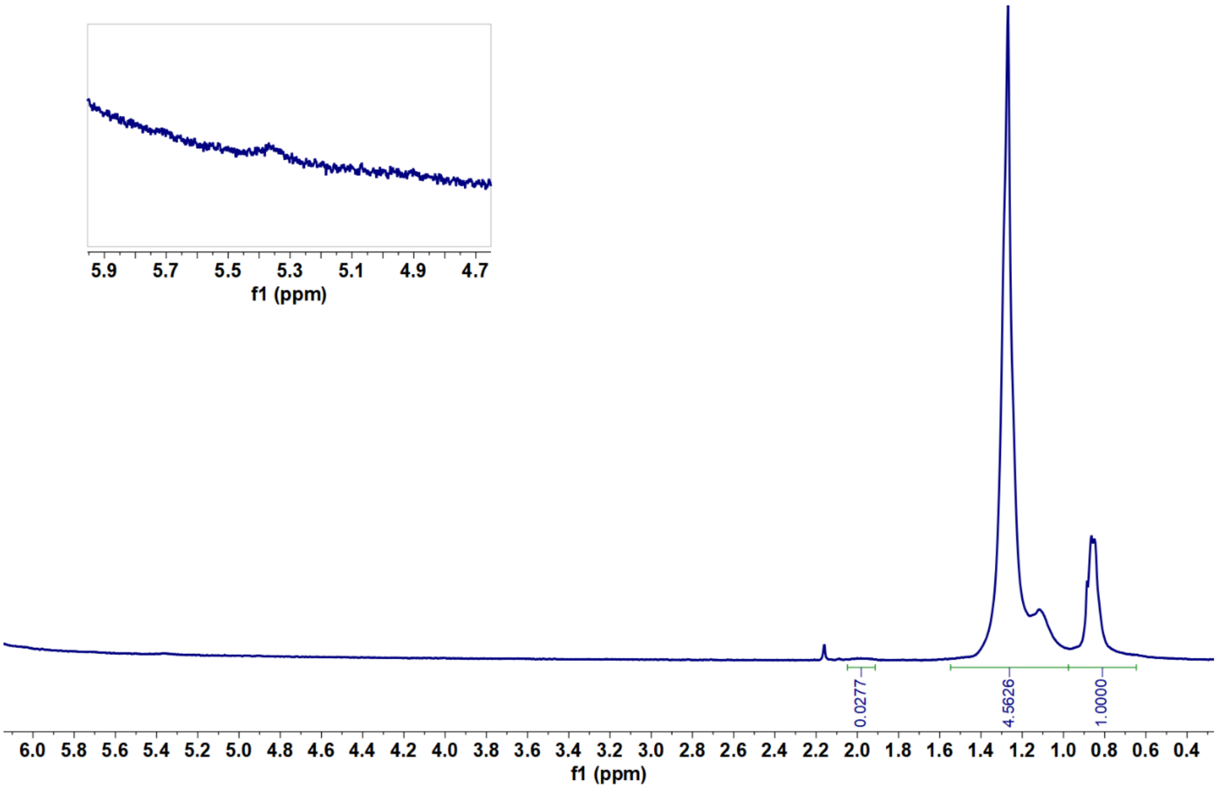

Figure S36 ^{1}H NMR spectrum (300 MHz, C_6D_6:1,2,4-TCB (1:2), 80 ºC) of the PE obtained using catalyst **3***, at 25 ºC and 3 bar of ethylene.

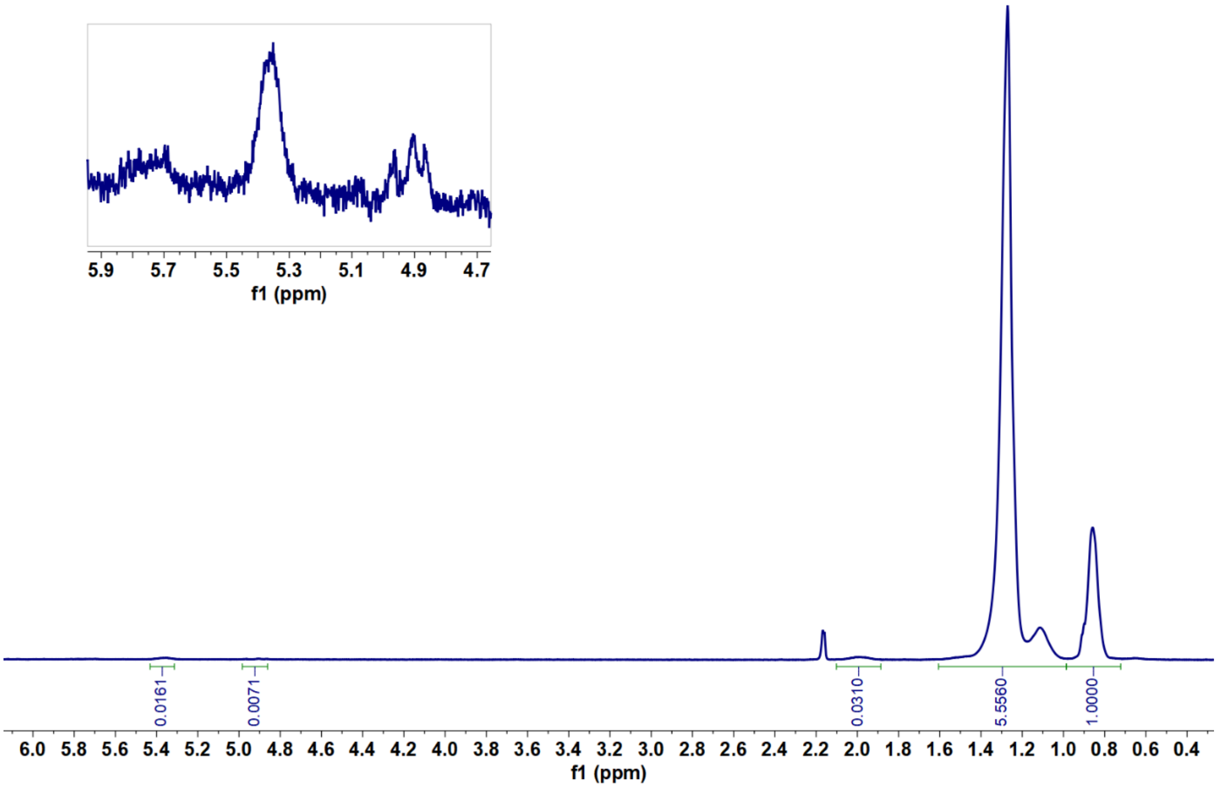

Figure S37 ^{1}H NMR spectrum (300 MHz, C_6D_6:1,2,4-TCB (1:2), 80 ºC) of the PE obtained using catalyst **3***, at 50 ºC and 3 bar of ethylene.

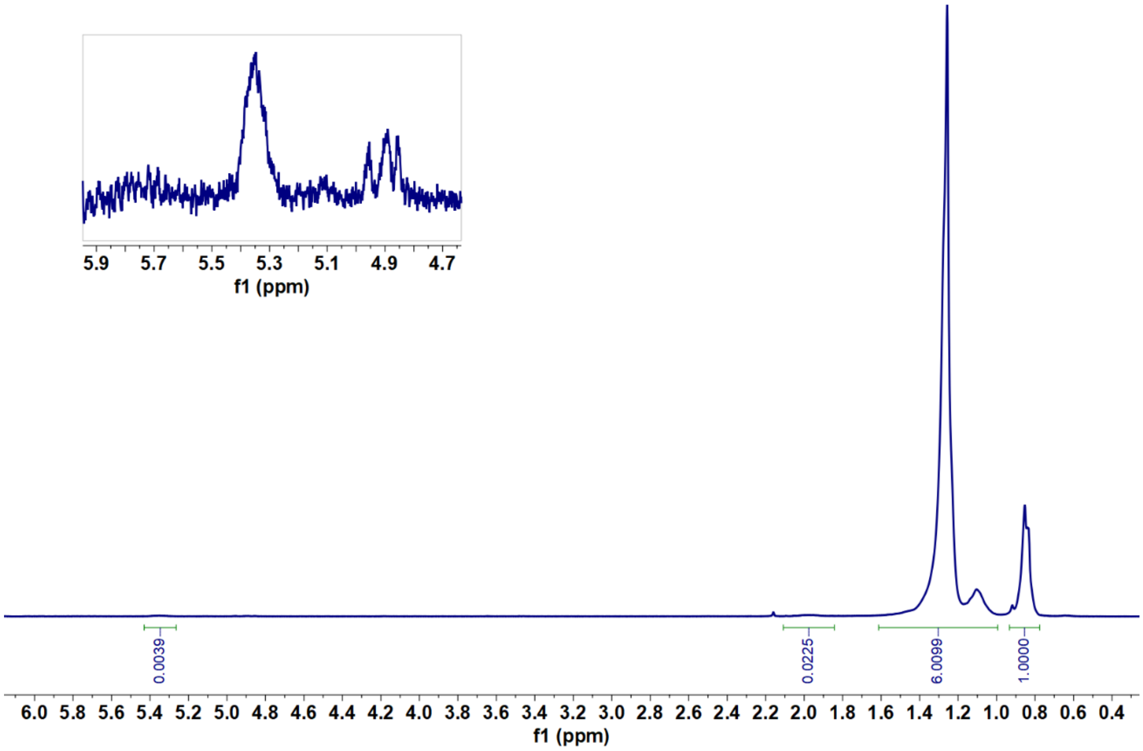

Figure S38 ^{1}H NMR spectrum (300 MHz, C_6D_6:1,2,4-TCB (1:2), 80 ºC) of the PE obtained using catalyst **3**, at 25 ºC and 9 bar of ethylene.

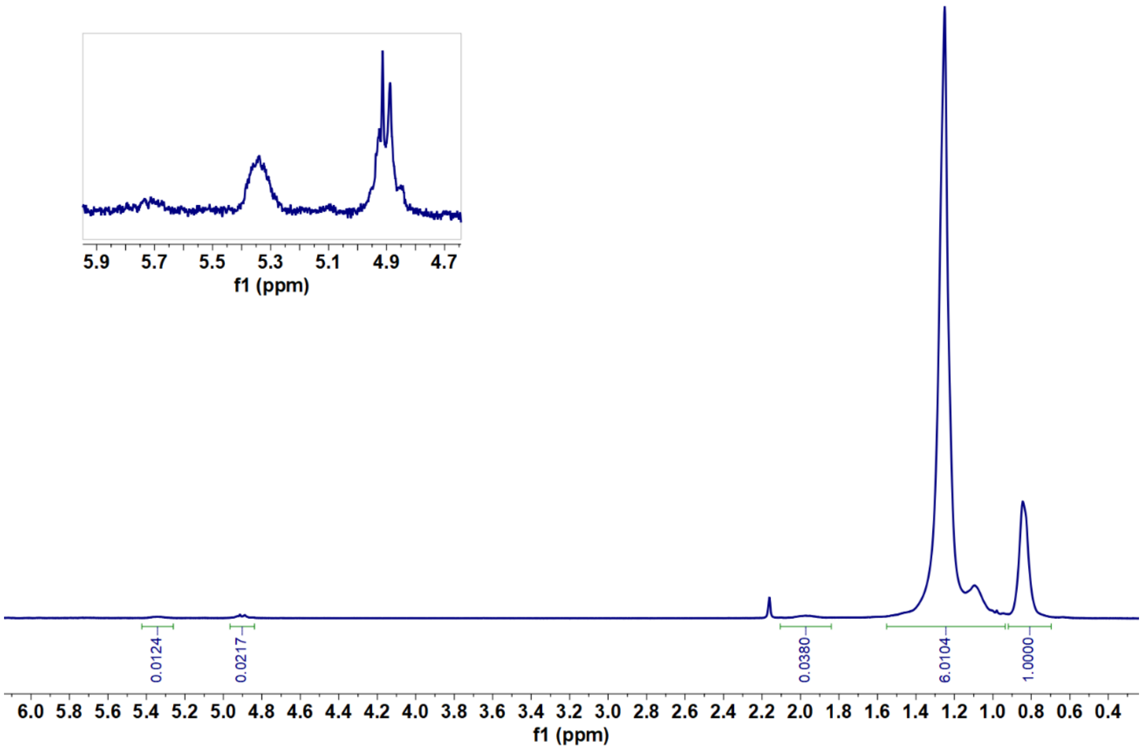

Figure S39 ^{1}H NMR spectrum (300 MHz, C_6D_6:1,2,4-TCB (1:2), 80 ºC) of the PE obtained using catalyst **3**, at 50 ºC and 9 bar of ethylene.

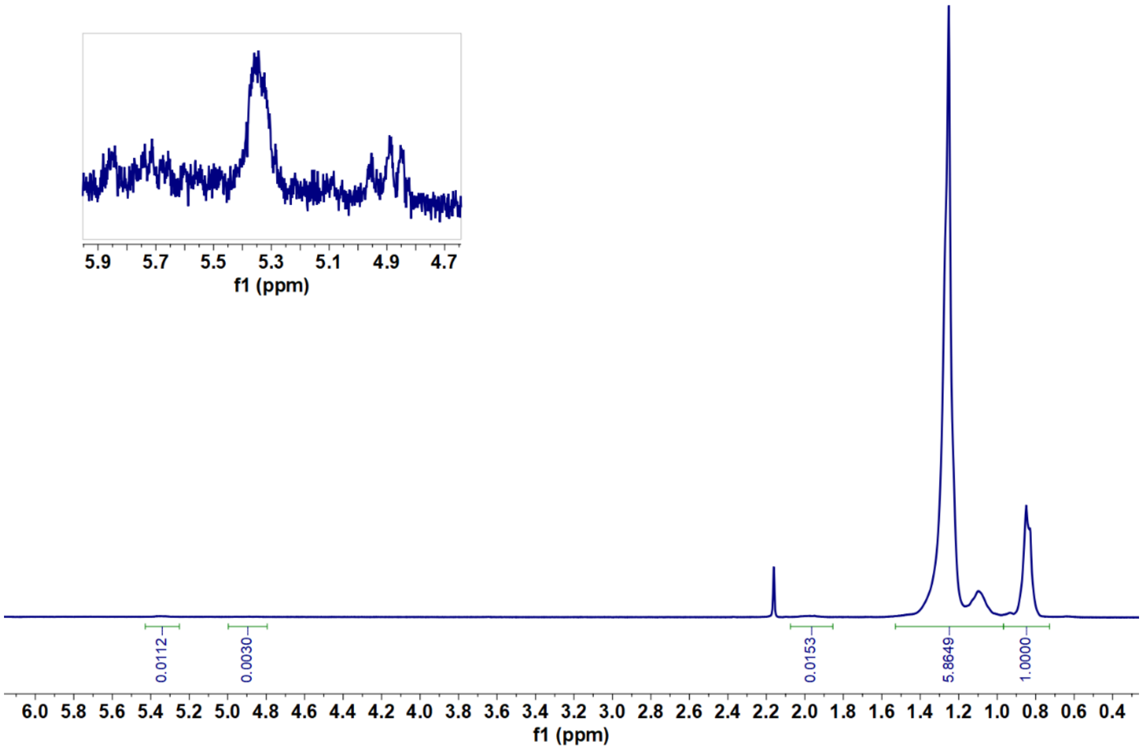

Figure S40 ^{1}H NMR spectrum (300 MHz, C_6D_6:1,2,4-TCB (1:2), 80 ºC) of the PE obtained using catalyst **3***, at 25 ºC and 9 bar of ethylene.

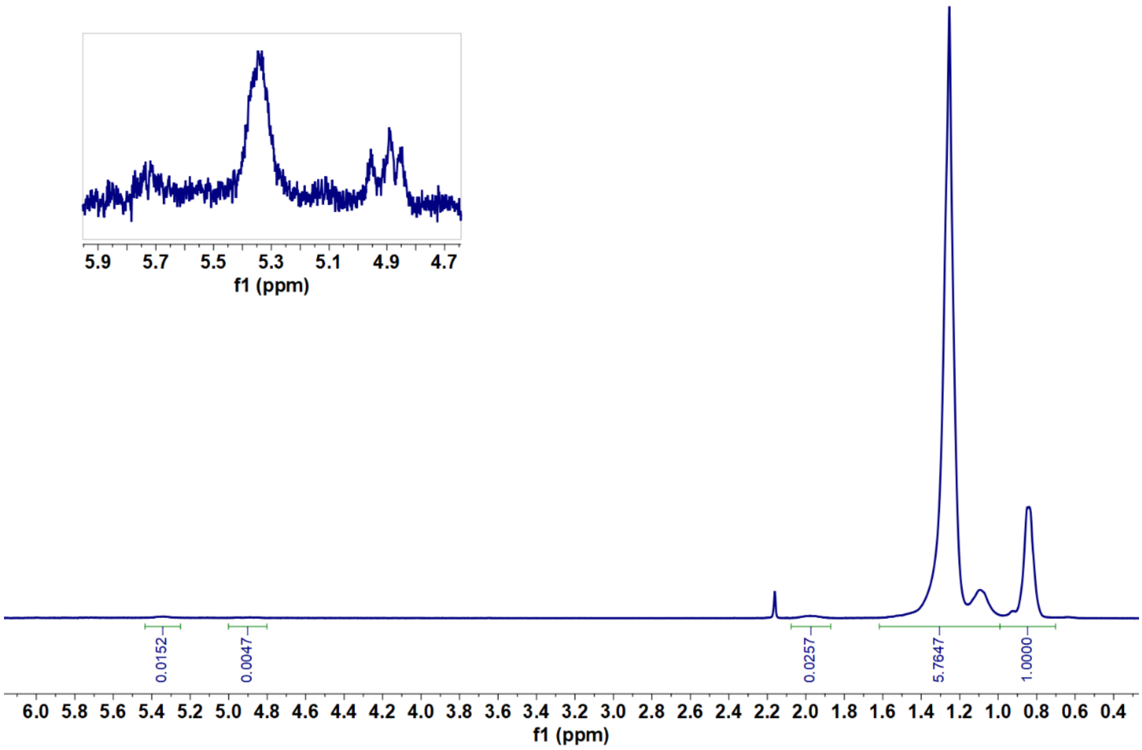

Figure S41 ^{1}H NMR spectrum (300 MHz, C_6D_6:1,2,4-TCB (1:2), 80 ºC) of the PE obtained using catalyst **3***, at 50 ºC and 9 bar of ethylene.

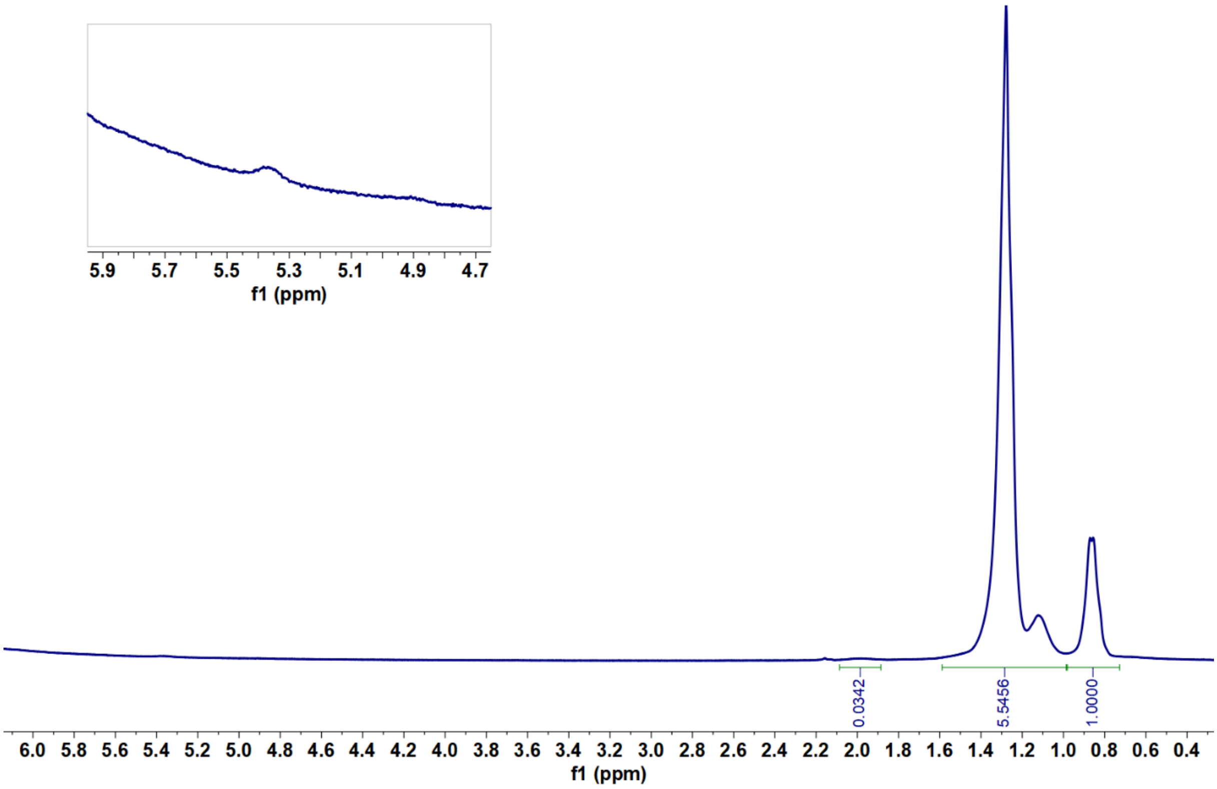

Figure S42 ^{1}H NMR spectrum (300 MHz, C_6D_6:1,2,4-TCB (1:2), 80 ºC) of the PE obtained using catalyst **3***, at 25 ºC and 15 bar of ethylene.

Polyethylene Characterization by $^{13}C\{^1H\}$ NMR - Microstructure Analysis

Determination of the Distribution of Branches (%): The $^{13}C\{^1H\}$ resonances characteristic of the PEs microstructure were identified as described in the literature.[9] The distribution of branches in the PEs is detailed in Table 2 of the manuscript. The assignment of the $^{13}C\{^1H\}$ NMR resonances is typically exemplified in Figure S43. The integration areas (I) used to quantify the different types of branches ($\boldsymbol{I_{Branch\ type}}$) correspond to the area of the methyl group of each type of branch (methyl, ethyl, propyl, butyl, *sec*-butyl and longer). Their calculation is performed according to the following equations:[9f,10]

$$\boldsymbol{I_{Methyl}} = I_6 - I_4 \text{ (where } I_4 = I_7) \quad \text{(S4)}$$

$$\boldsymbol{I_{Ethyl}} = I_1 \quad \text{(S5)}$$

$$\boldsymbol{I_{Propyl}} = I_4 \quad \text{(S6)}$$

$$\boldsymbol{I_{Butyl}} = I_3 - [(I_8 + I_{17})/2] \quad \text{(S7)}$$

$$\boldsymbol{I_{Sec\text{-}Butyl}} = (I_2 + I_5)/2 \quad \text{(S8)}$$

$$\boldsymbol{I_{Longer}} = (I_8 + I_{17})/2 \quad \text{(S9)}$$

The total intensity of methyl groups, $\boldsymbol{I_{Total\ CH3}}$, is given by:

$$\boldsymbol{I_{Total\ CH3}} = (I_2 + I_5)/2 + I_1 + I_3 + I_6 \quad \text{(S10)}$$

where I_6 is the total integral $I_6 + I_7$, with $I_7 = I_4$.

The branches distribution (%) is determined by the ratio between the intensities of each one of the branch types and the overall methyl groups intensity multiplied by 100 (equation S11):

$$\boldsymbol{Branch\ type\ (\%)} = \frac{I_{Branch\ type}}{I_{Total\ CH_3}} \times 100 \quad \text{(S11)}$$

$^{13}C\{^1H\}$ NMR Spectra of Selected Polyethylene Products

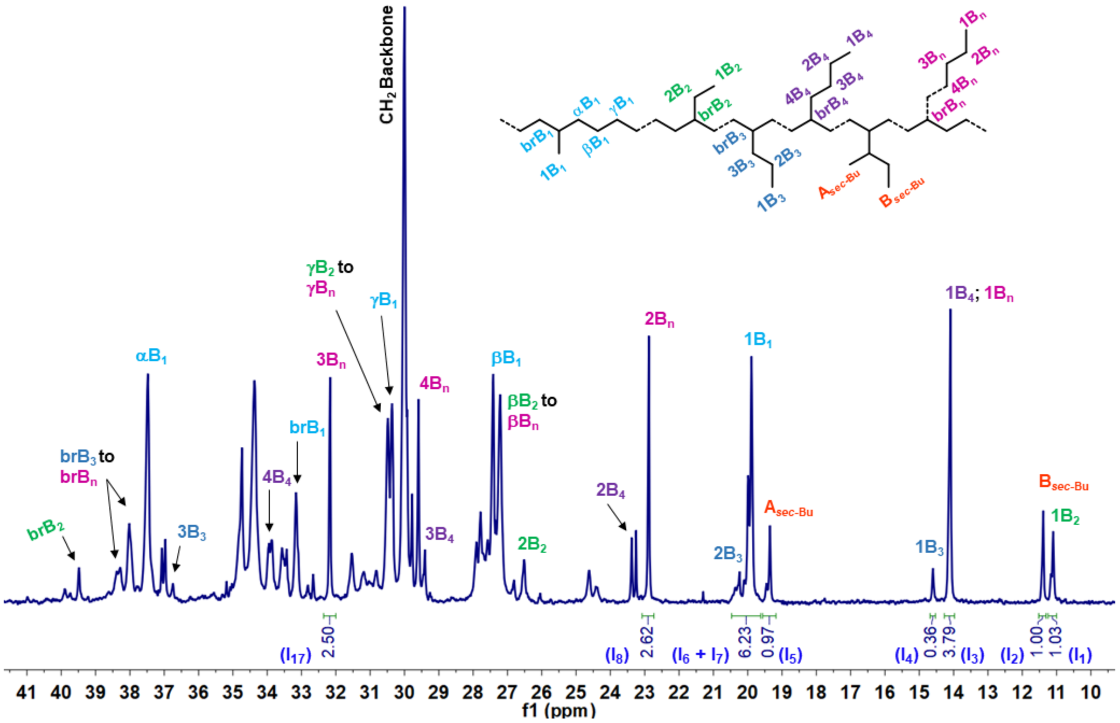

Figure S43 $^{13}C\{^1H\}$ NMR spectrum (75 MHz, C_6D_6:1,2,4-TCB (1:2), 80 ºC) of the PE obtained with catalyst system **1***, at 50 ºC and 9 bar of ethylene, and the corresponding ^{13}C resonances assignments.

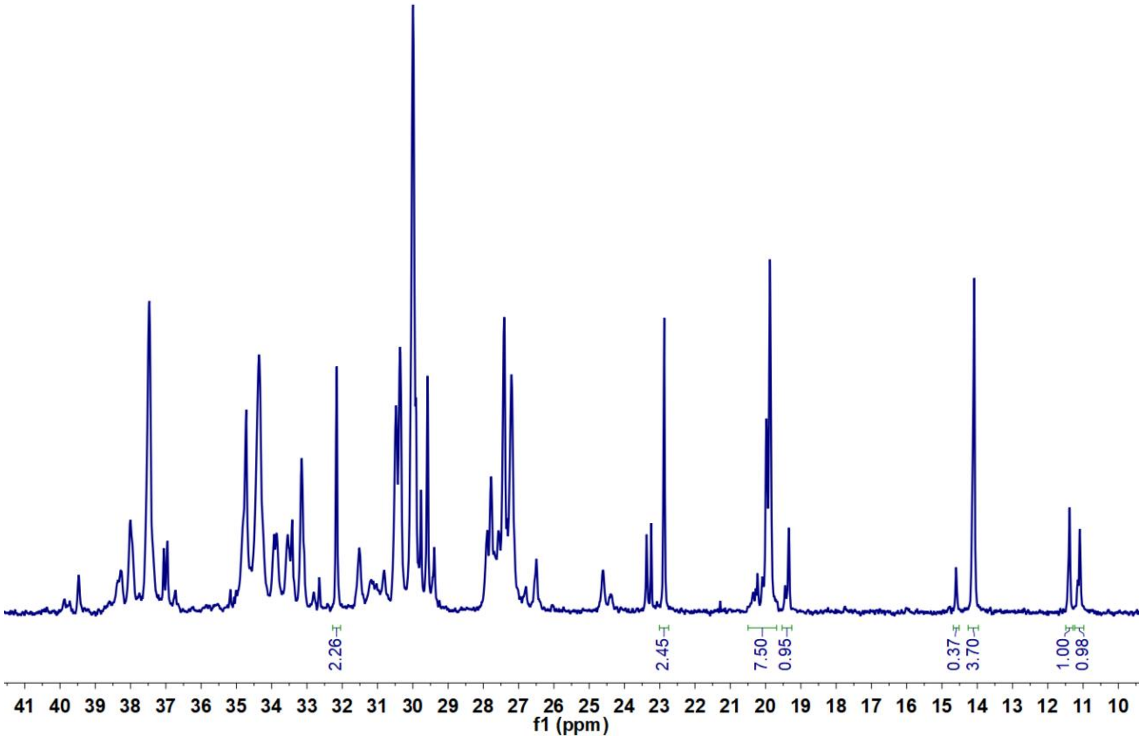

Figure S44 $^{13}C\{^1H\}$ NMR spectrum (75 MHz, C_6D_6:1,2,4-TCB (1:2), 80 ºC) of the PE obtained with catalyst system **1***, at 25 ºC and 9 bar of ethylene.

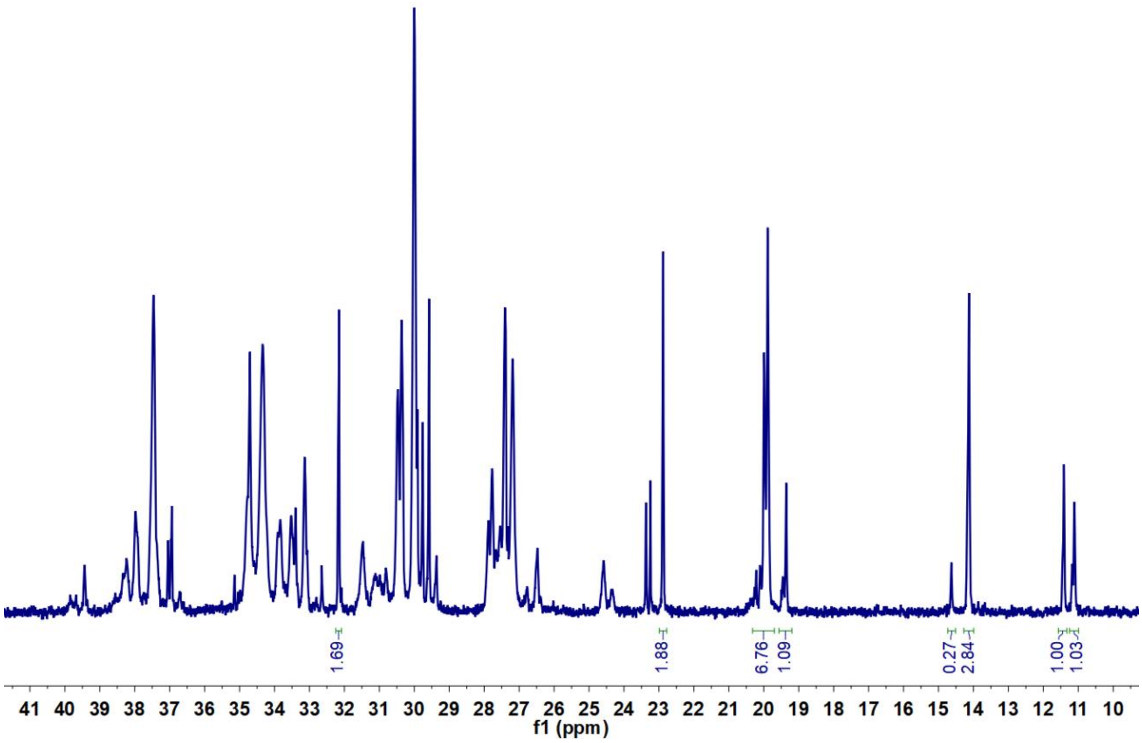

Figure S45 $^{13}C\{^{1}H\}$ NMR spectrum (75 MHz, C_6D_6:1,2,4-TCB (1:2), 80 ºC) of the PE obtained with catalyst system **2***, at 25 ºC and 3 bar of ethylene.

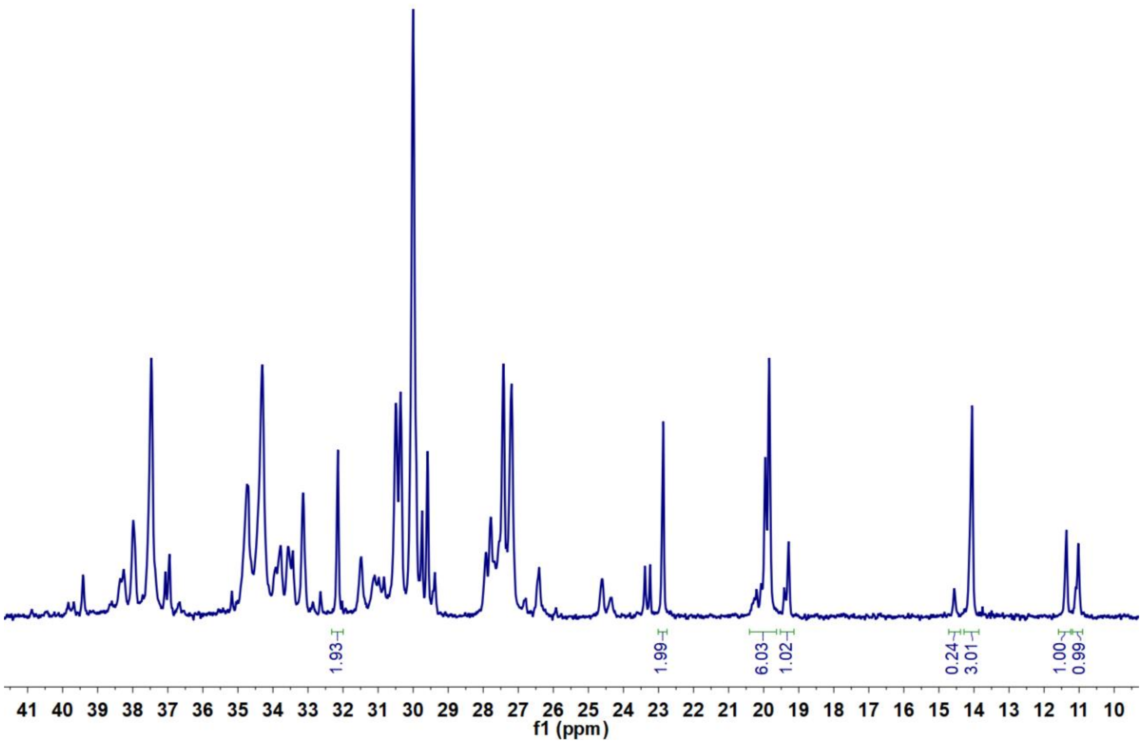

Figure S46 $^{13}C\{^{1}H\}$ NMR spectrum (75 MHz, C_6D_6:1,2,4-TCB (1:2), 80 ºC) of the PE obtained with catalyst system **2***, at 50 ºC and 3 bar of ethylene.

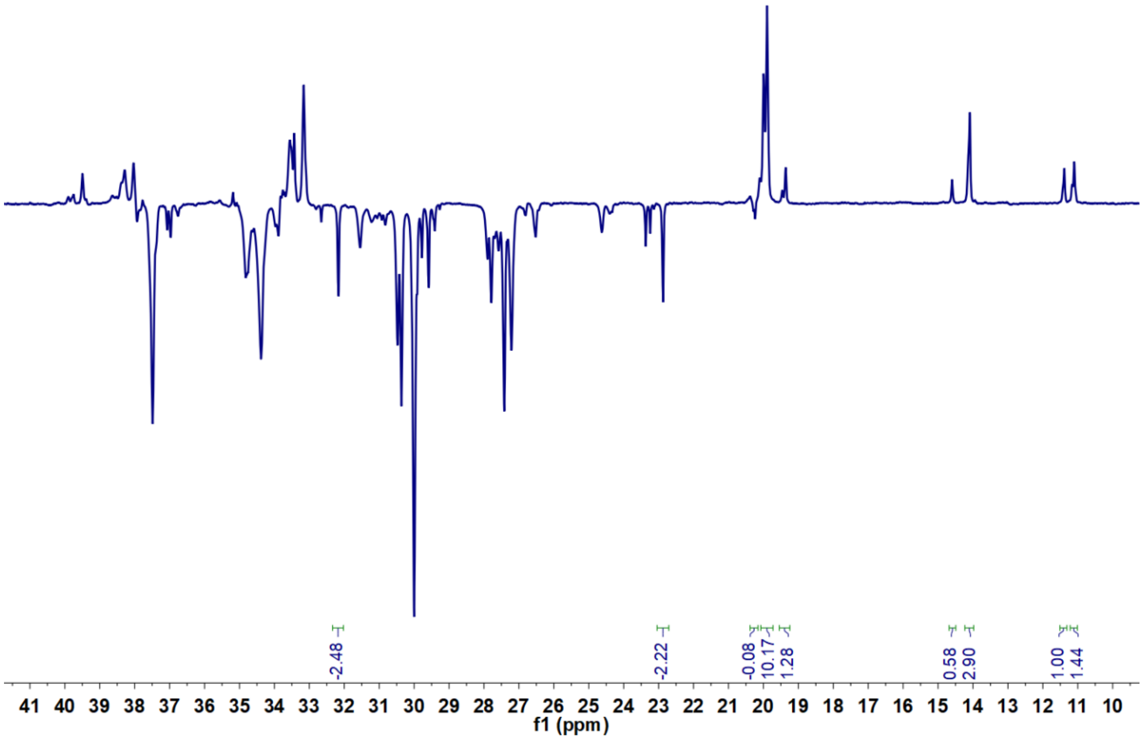

Figure S47 ^{13}C APT NMR spectrum (75 MHz, C_6D_6:1,2,4-TCB (1:2), 80 ºC) of the PE obtained with catalyst system **2***, at 25 ºC and 9 bar of ethylene.

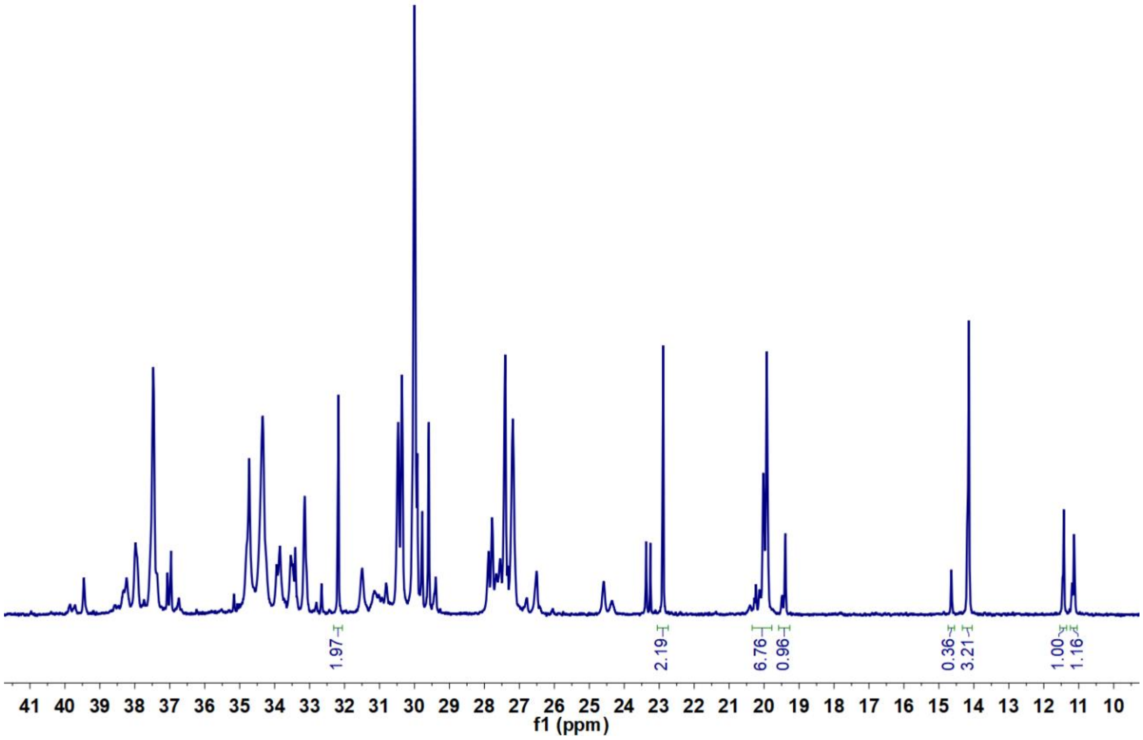

Figure S48 $^{13}C\{^1H\}$ NMR spectrum (75 MHz, C_6D_6:1,2,4-TCB (1:2), 80 ºC) of the PE obtained with catalyst system **2***, at 50 ºC and 9 bar of ethylene.

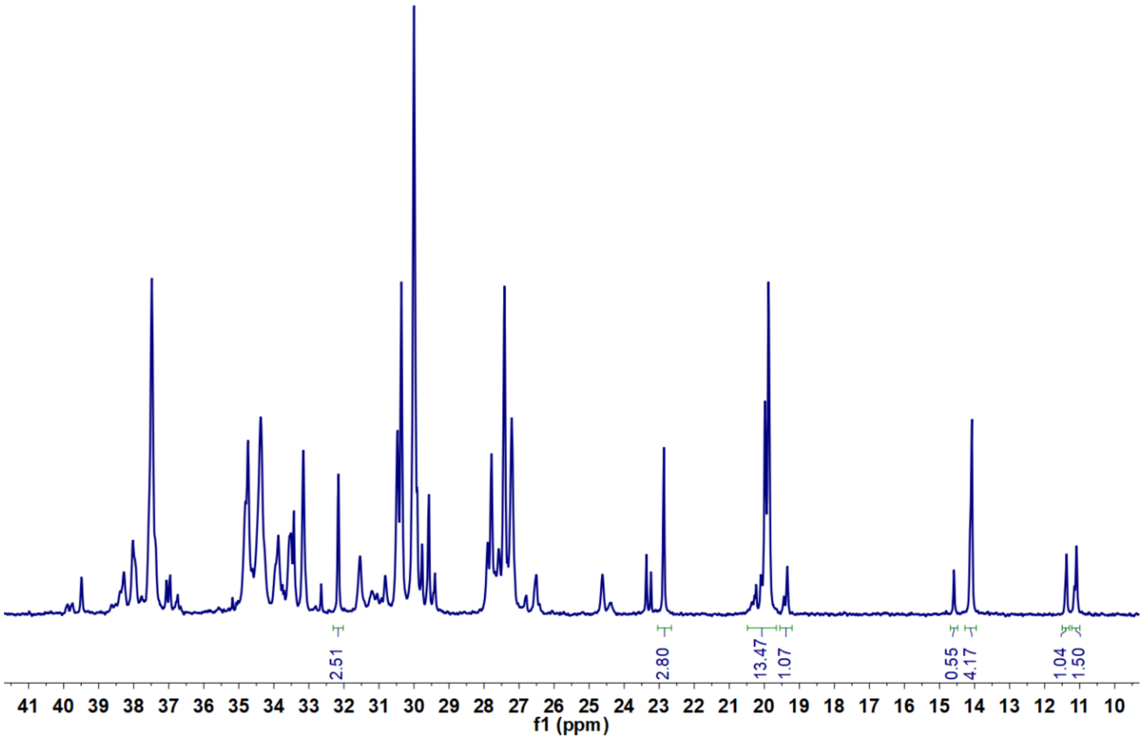

Figure S49 $^{13}C\{^1H\}$ NMR spectrum (75 MHz, C_6D_6:1,2,4-TCB (1:2), 80 ºC) of the PE obtained with catalyst system **2***, at 25 ºC and 15 bar of ethylene.

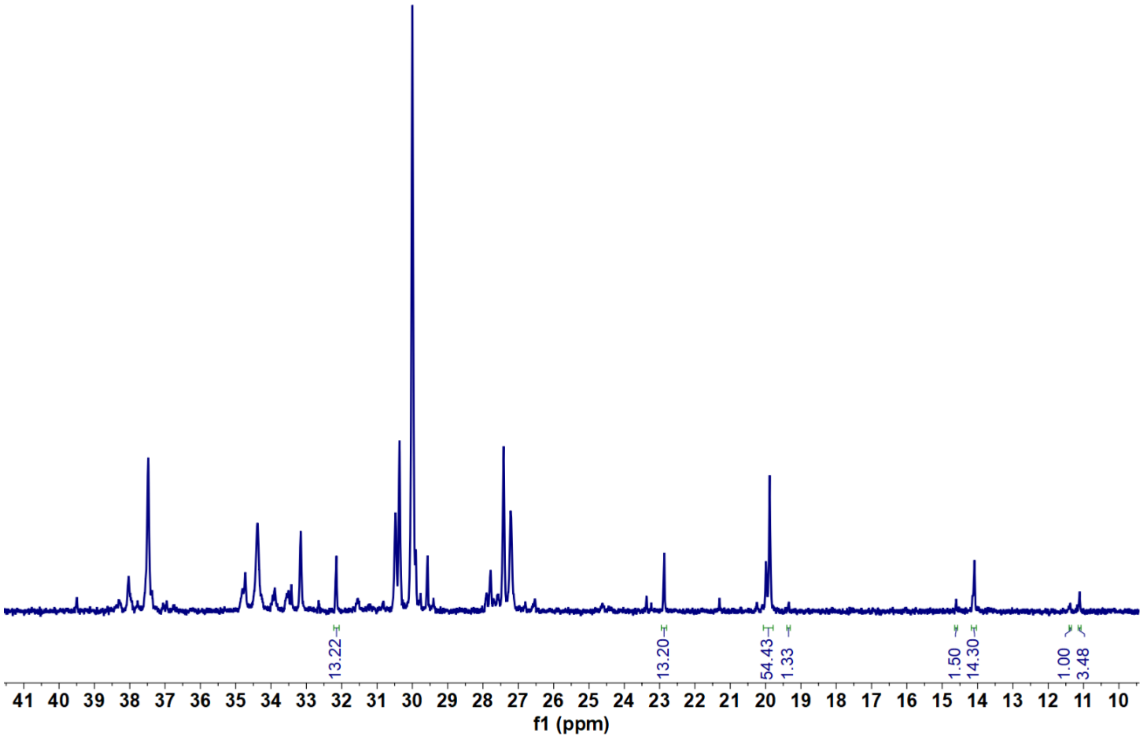

Figure S50 $^{13}C\{^1H\}$ NMR spectrum (75 MHz, C_6D_6:1,2,4-TCB (1:2), 80 ºC) of the PE obtained with catalyst system **3***, at 25 ºC and 9 bar of ethylene.

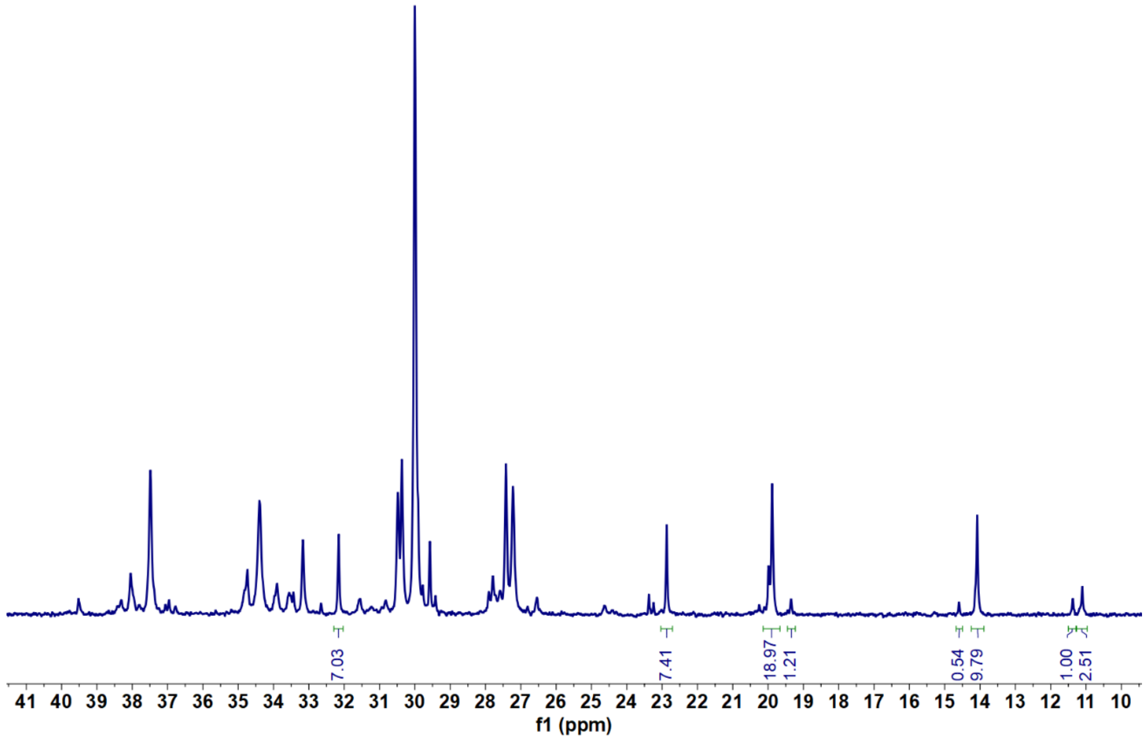

Figure S51 $^{13}C\{^{1}H\}$ NMR spectrum (75 MHz, C_6D_6:1,2,4-TCB (1:2), 80 ºC) of the PE obtained with catalyst system **3***, at 50 ºC and 9 bar of ethylene.

GPC/SEC Chromatograms of Polyethylene Products

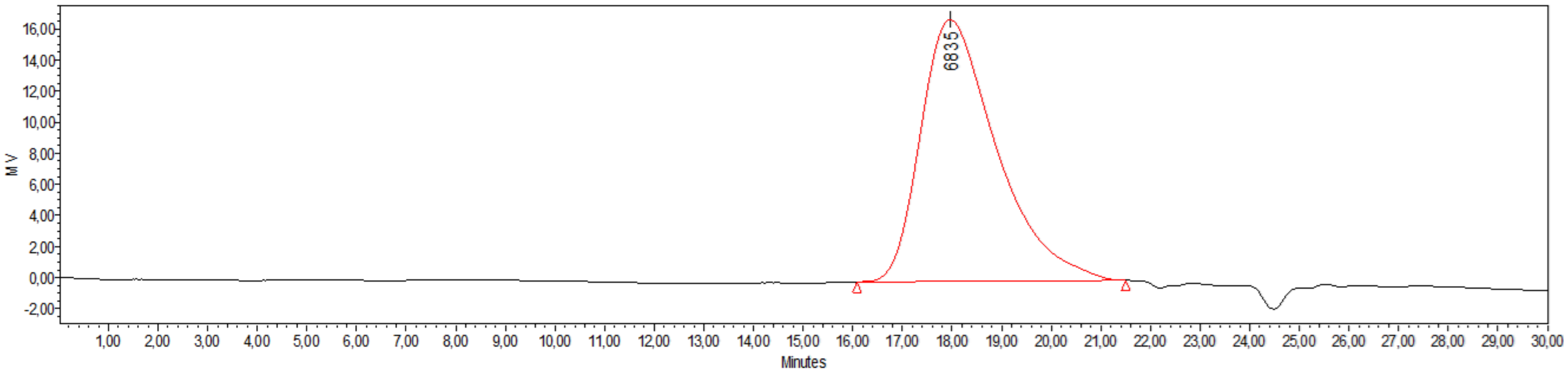

Figure S52 GPC/SEC chromatogram of the PE obtained with catalyst system **1***, at 3 bar and 25 ºC.

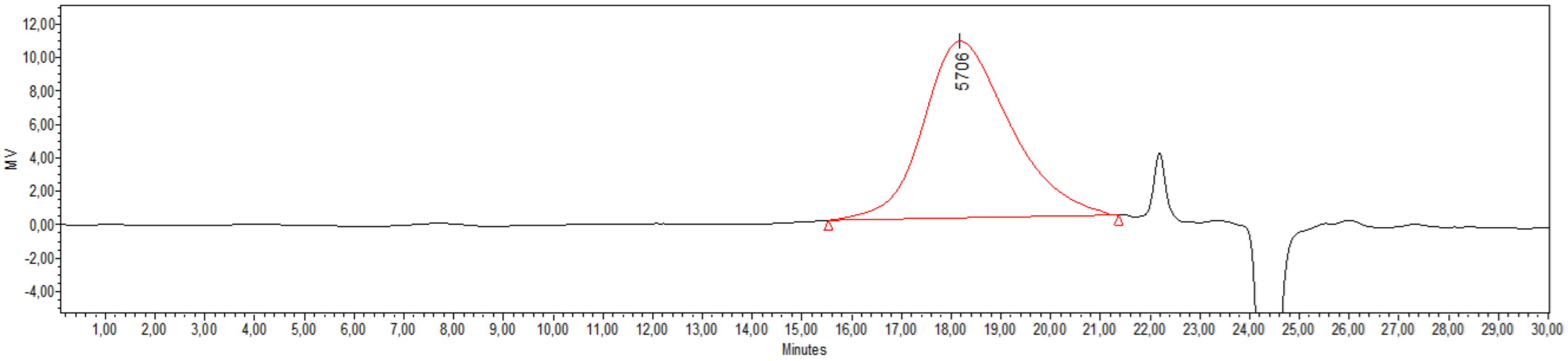

Figure S53 GPC/SEC chromatogram of the PE obtained with catalyst system **1***, at 3 bar and 50 ºC.

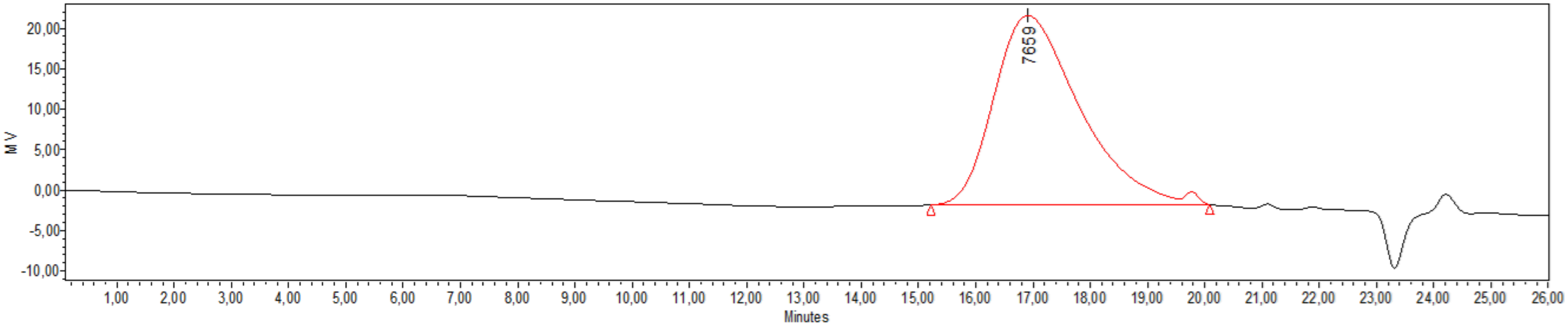

Figure S54 GPC/SEC chromatogram of the PE obtained with catalyst **1**, at 9 bar and 25 ºC.

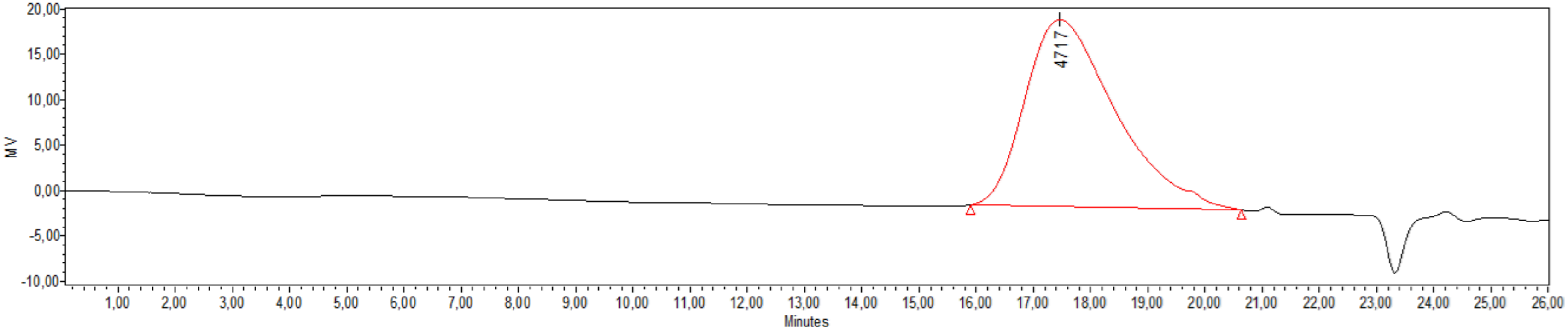

Figure S55 GPC/SEC chromatogram of the PE obtained with catalyst **1**, at 9 bar and 50 ºC.

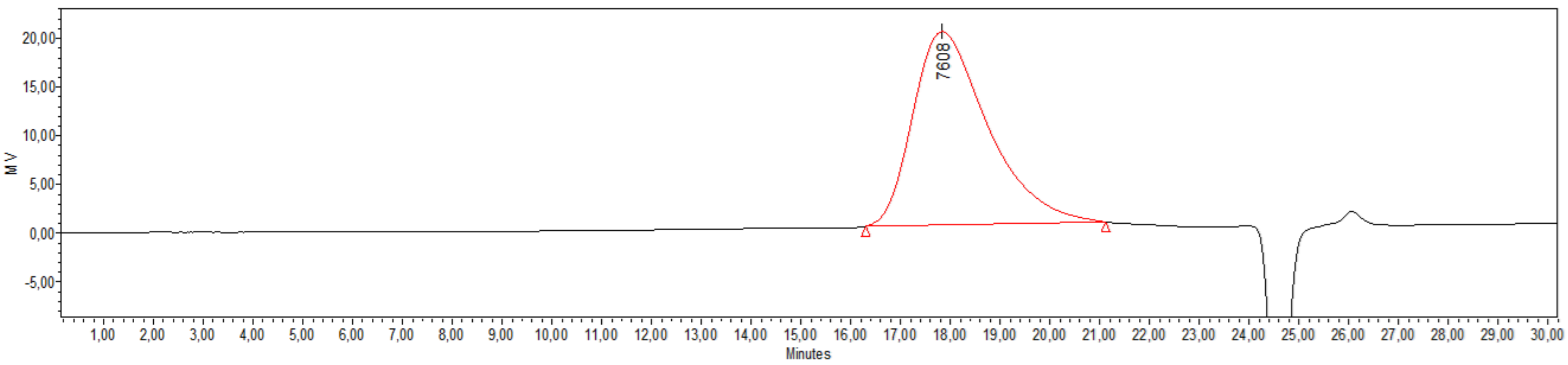

Figure S56 GPC/SEC chromatogram of the PE obtained with catalyst system **1***, at 9 bar and 25 ºC.

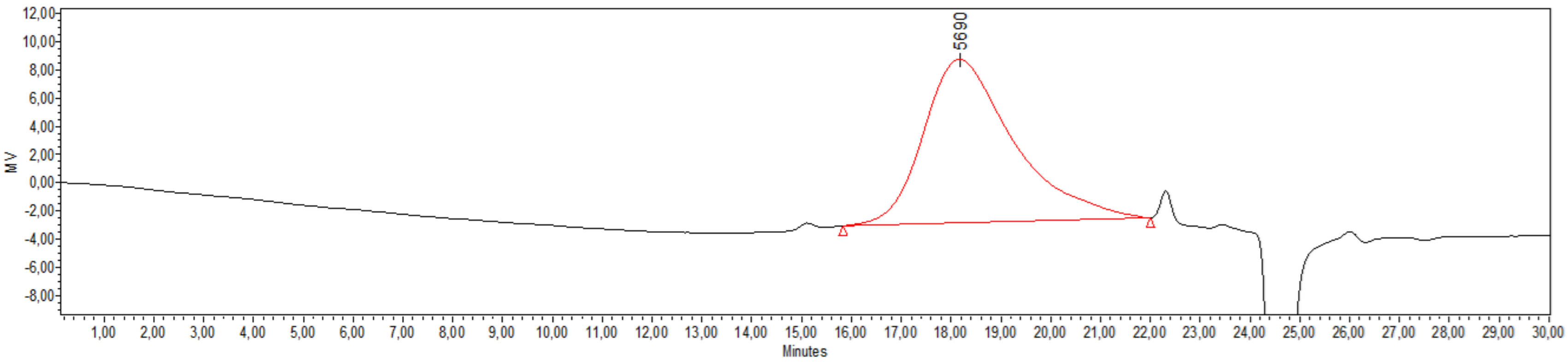

Figure S57 GPC/SEC chromatogram of the PE obtained with catalyst system **1***, at 9 bar and 50 ºC.

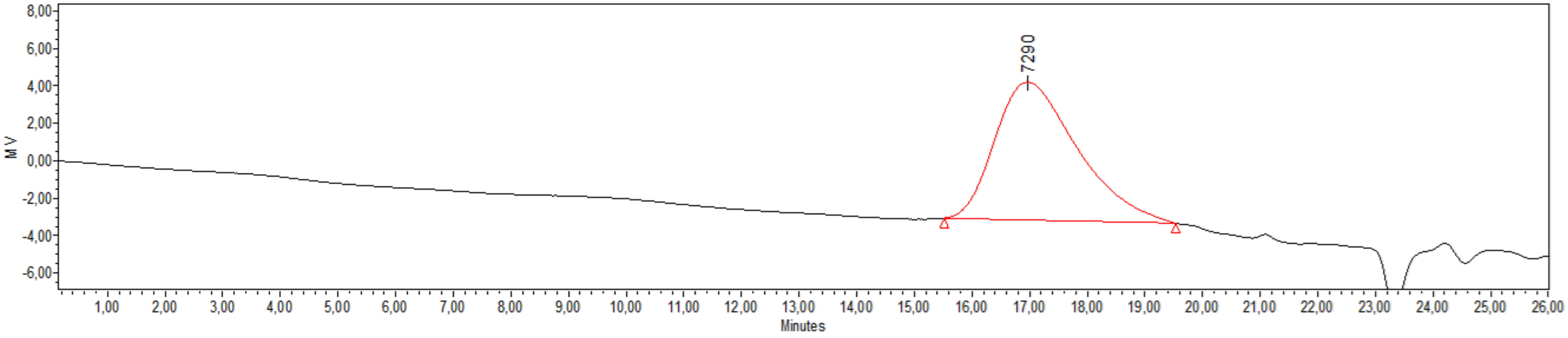

Figure S58 GPC/SEC chromatogram of the PE obtained with catalyst **1**, at 15 bar and 25 ºC.

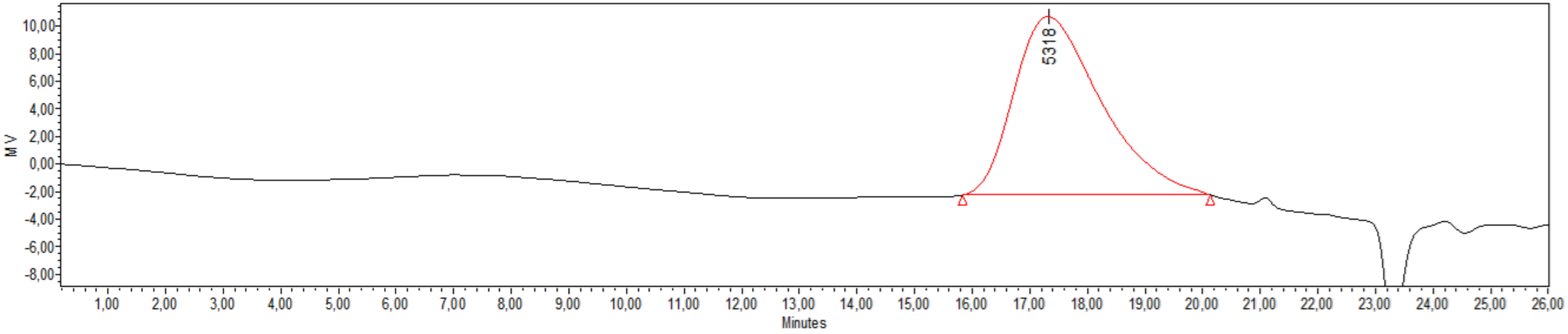

Figure S59 GPC/SEC chromatogram of the PE obtained with catalyst **1**, at 15 bar and 50 ºC.

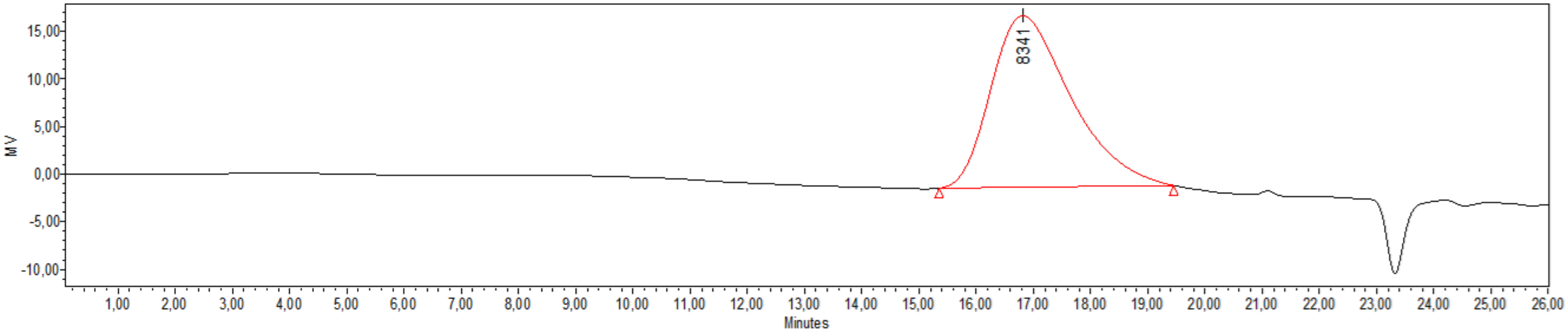

Figure S60 GPC/SEC chromatogram of the PE obtained with catalyst system **1***, at 15 bar and 25 ºC.

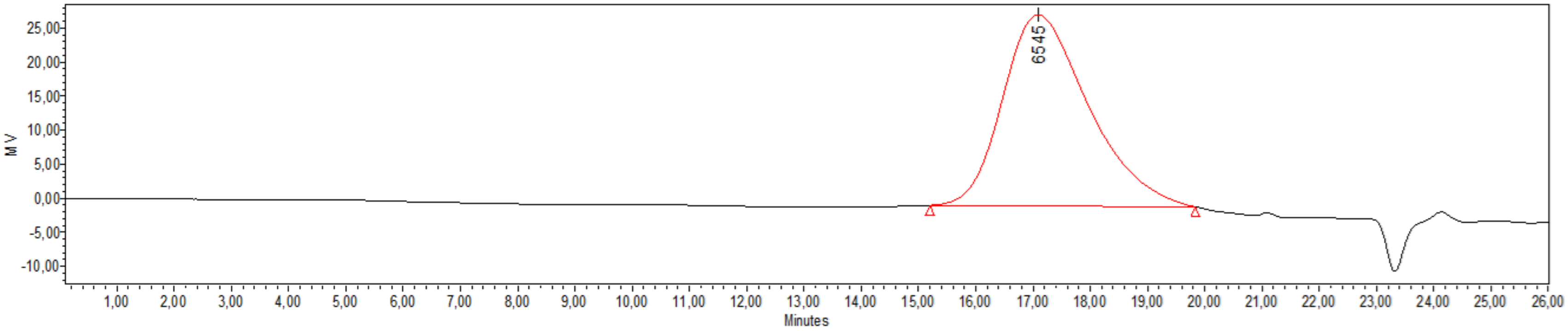

Figure S61 GPC/SEC chromatogram of the PE obtained with catalyst system **1***, at 15 bar and 50 ºC.

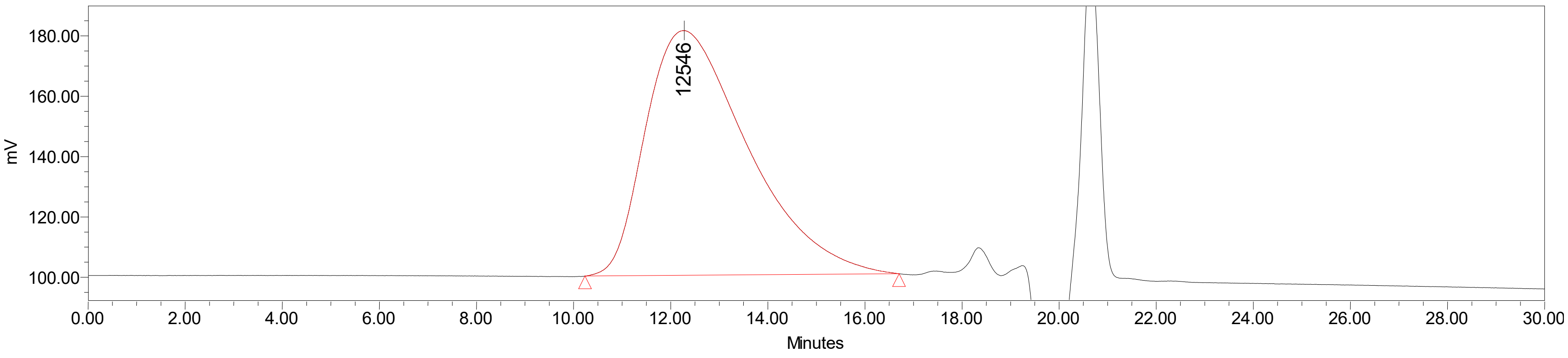

Figure S62 GPC/SEC chromatogram of the PE obtained with catalyst **2**, at 3 bar and 25 ºC.

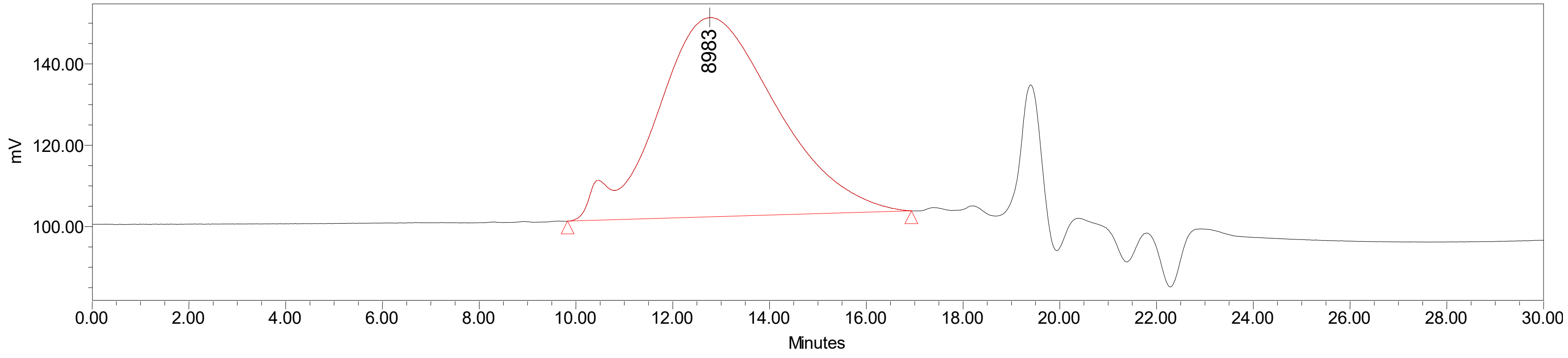

Figure S63 GPC/SEC chromatogram of the PE obtained with catalyst **2**, at 3 bar and 50 ºC.

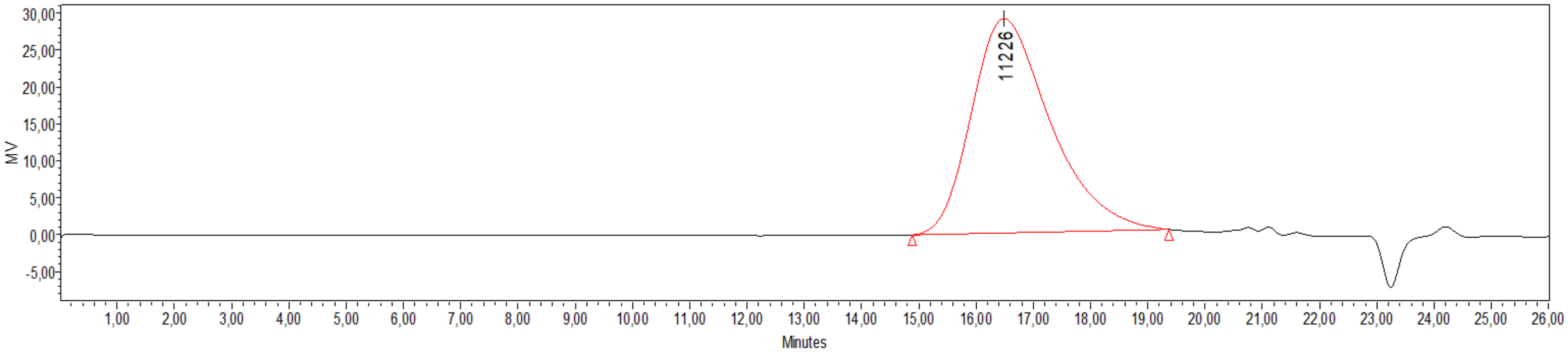

Figure S64 GPC/SEC chromatogram of the PE obtained with catalyst system **2***, at 3 bar and 25 ºC.

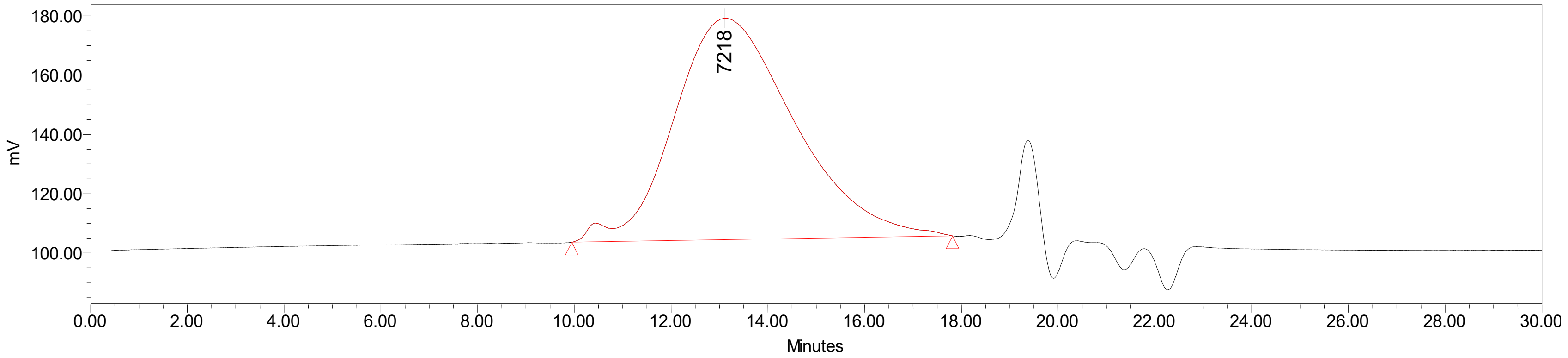

Figure S65 GPC/SEC chromatogram of the PE obtained with catalyst system **2***, at 3 bar and 50 ºC.

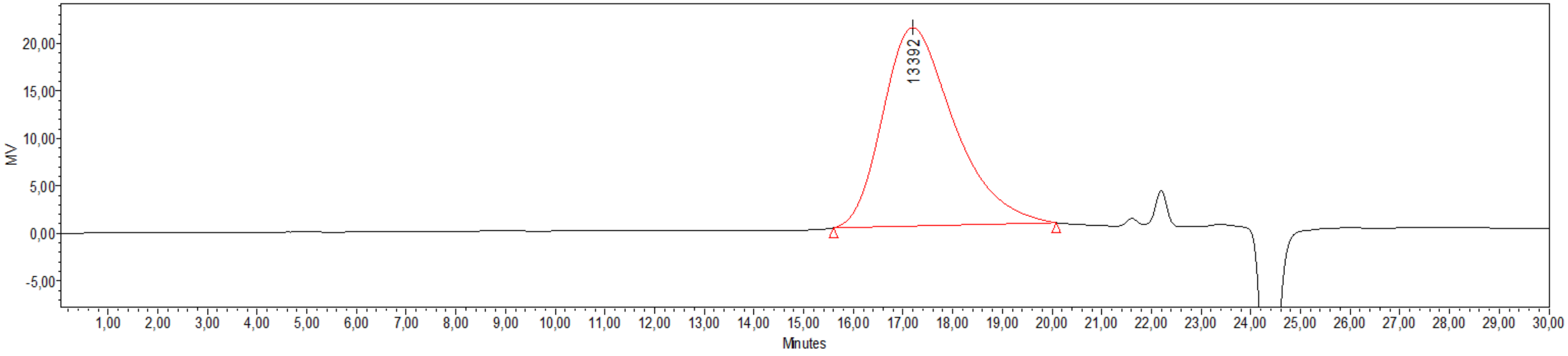

Figure S66 GPC/SEC chromatogram of the PE obtained with catalyst **2**, at 9 bar and 25 ºC.

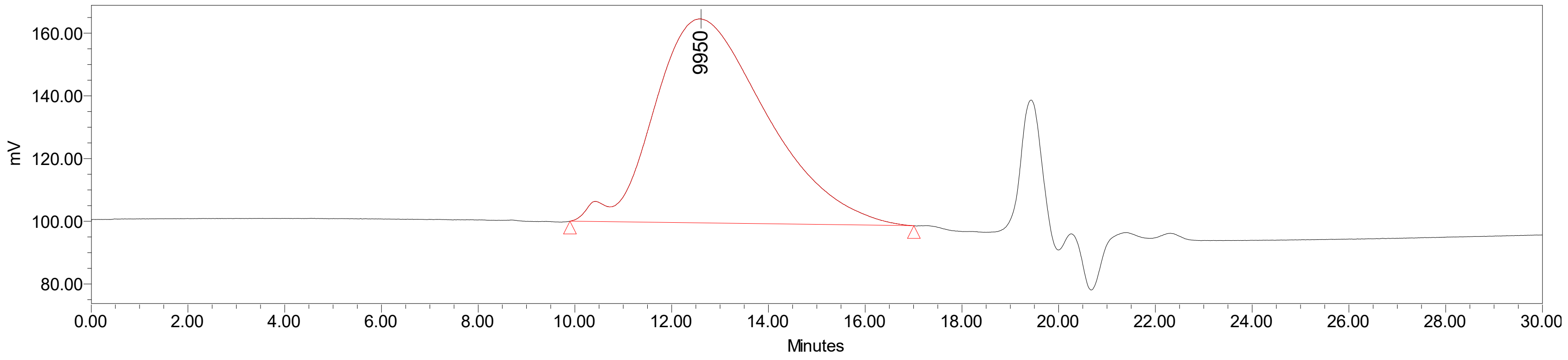

Figure S67 GPC/SEC chromatogram of the PE obtained with catalyst **2**, at 9 bar and 50 ºC.

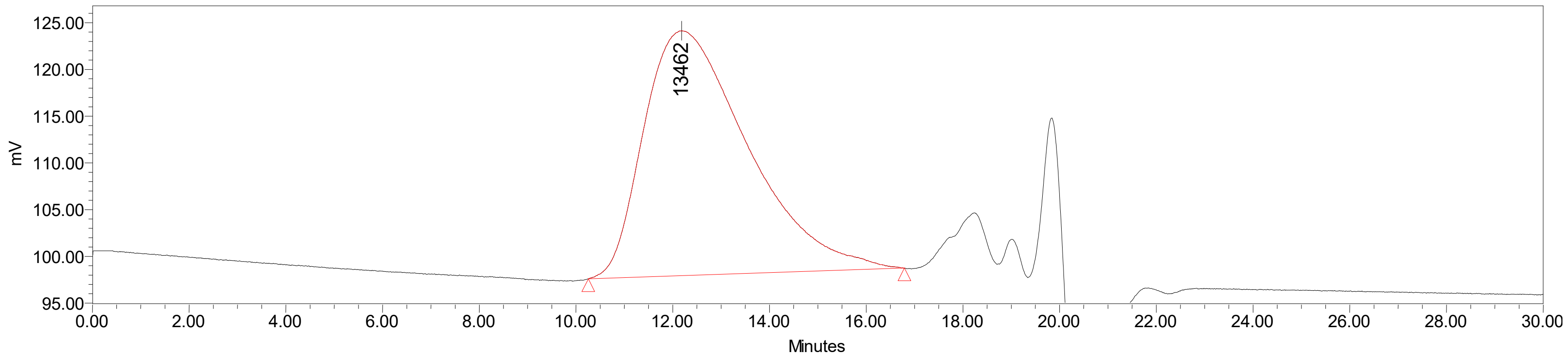

Figure S68 GPC/SEC chromatogram of the PE obtained with catalyst system **2***, at 9 bar and 25 ºC.

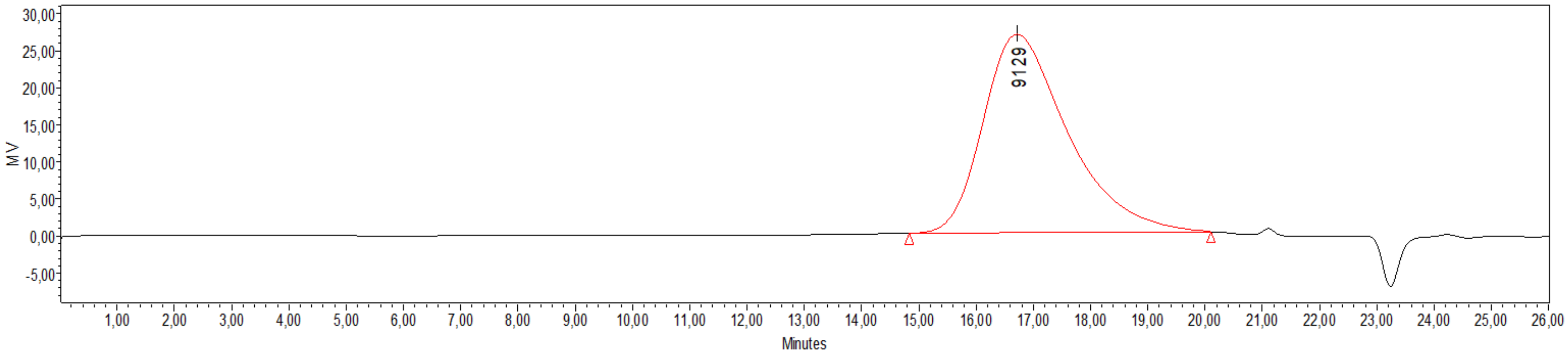

Figure S69 GPC/SEC chromatogram of the PE obtained with catalyst system **2***, at 9 bar and 50 ºC.

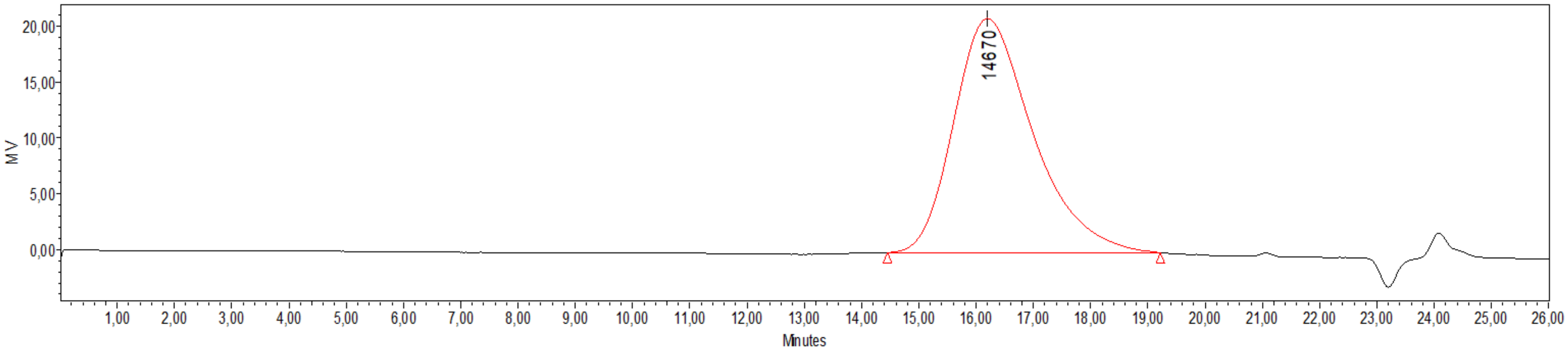

Figure S70 GPC/SEC chromatogram of the PE obtained with catalyst **2**, at 15 bar and 25 ºC.

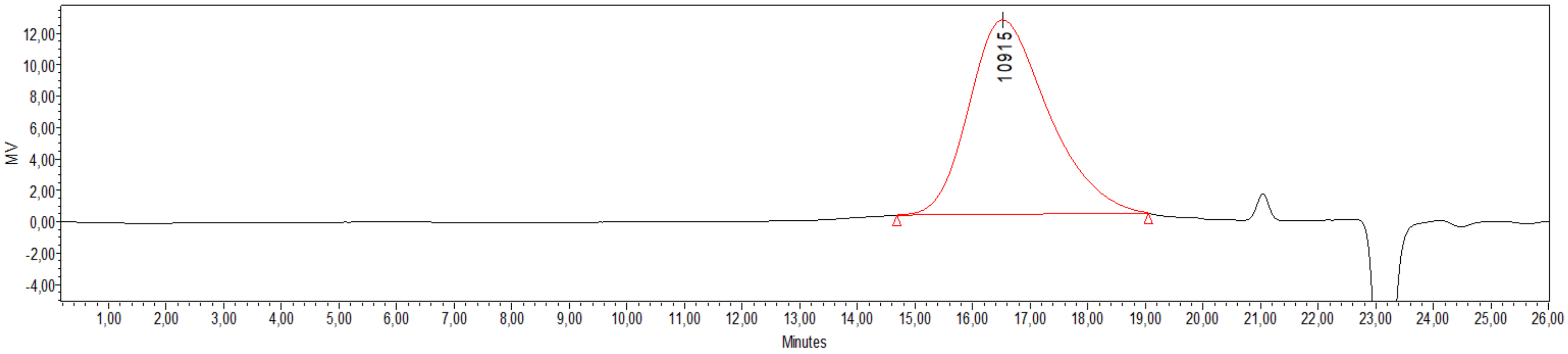

Figure S71 GPC/SEC chromatogram of the PE obtained with catalyst **2**, at 15 bar and 50 ºC.

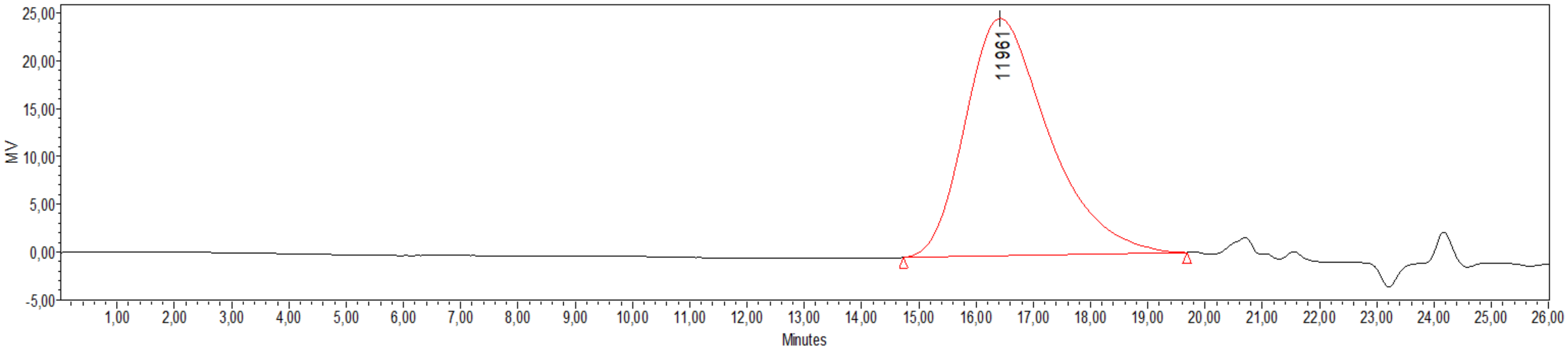

Figure S72 GPC/SEC chromatogram of the PE obtained with catalyst system **2***, at 15 bar and 25 ºC.

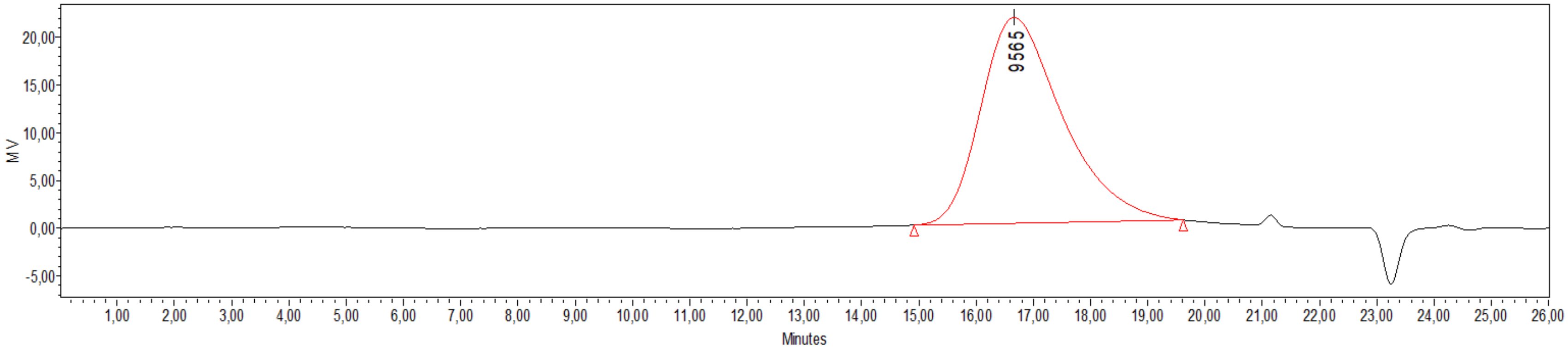

Figure S73 GPC/SEC chromatogram of the PE obtained with catalyst system **2***, at 15 bar and 50 ºC.

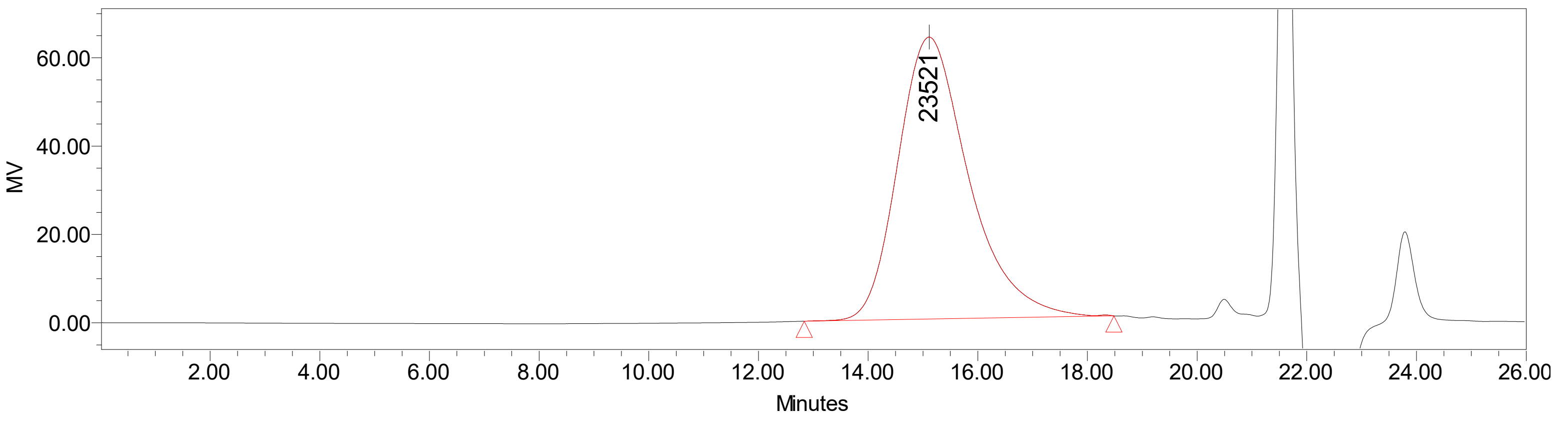

Figure S74 GPC/SEC chromatogram of the PE obtained with catalyst **3***, at 3 bar and 25 ºC.

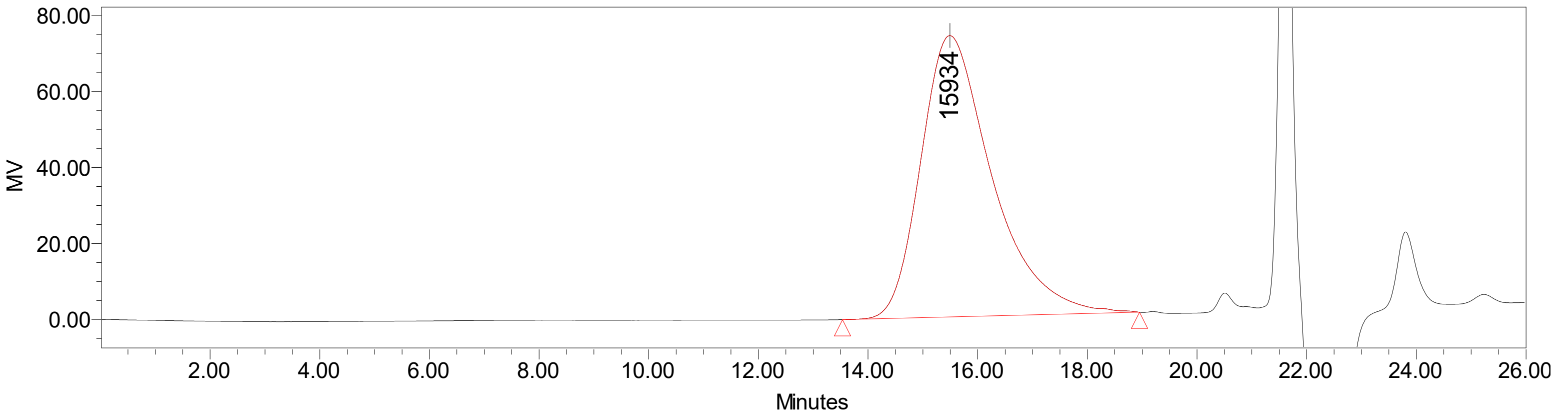

Figure S75 GPC/SEC chromatogram of the PE obtained with catalyst **3**, at 3 bar and 50 ºC.

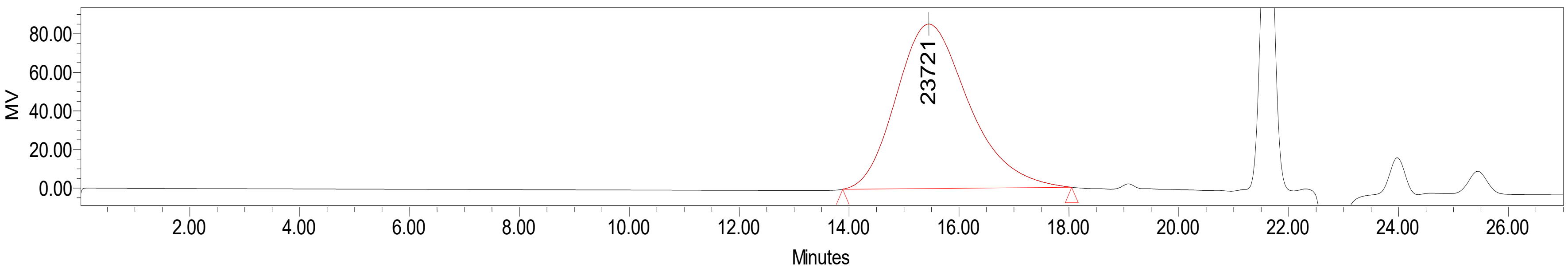

Figure S76 GPC/SEC chromatogram of the PE obtained with catalyst **3**, at 9 bar and 25 ºC.

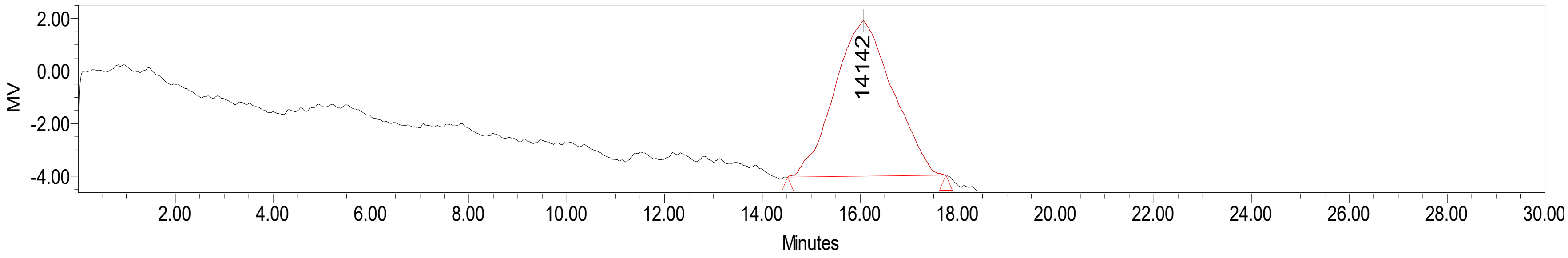

Figure S77 GPC/SEC chromatogram of the PE obtained with catalyst **3**, at 9 bar and 50 ºC.

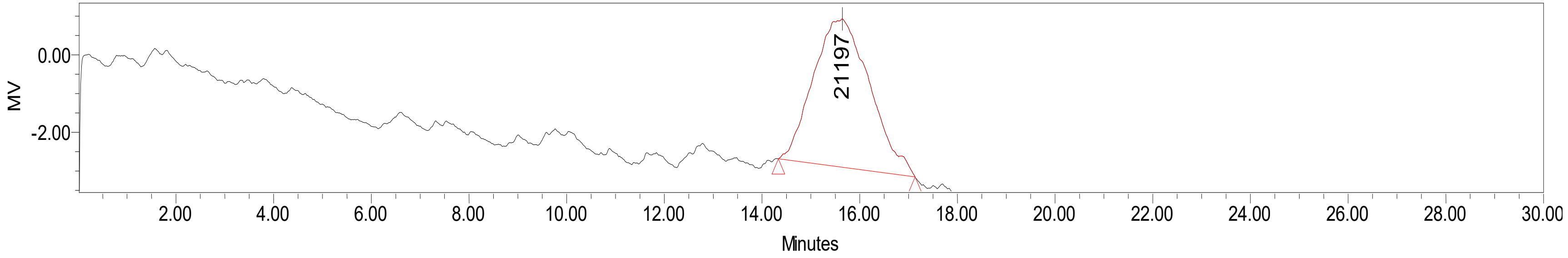

Figure S78 GPC/SEC chromatogram of the PE obtained with catalyst system **3***, at 9 bar and 25 ºC.

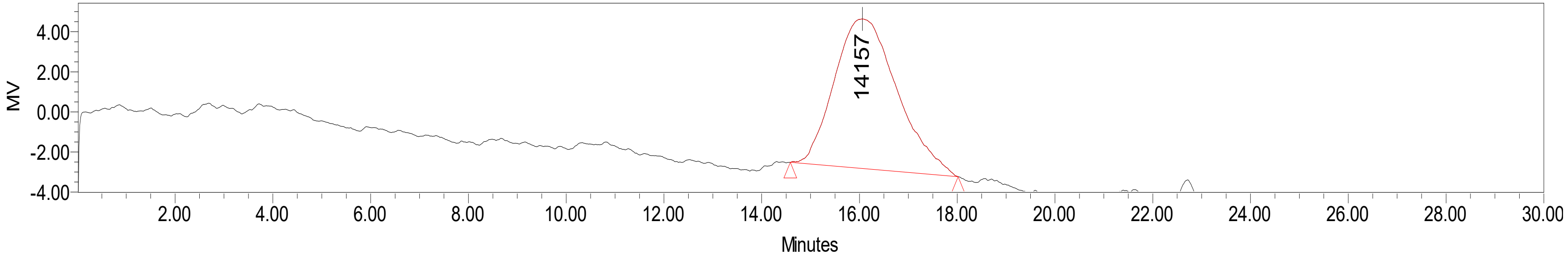

Figure S79 GPC/SEC chromatogram of the PE obtained with catalyst system **3***, at 9 bar and 50 ºC.

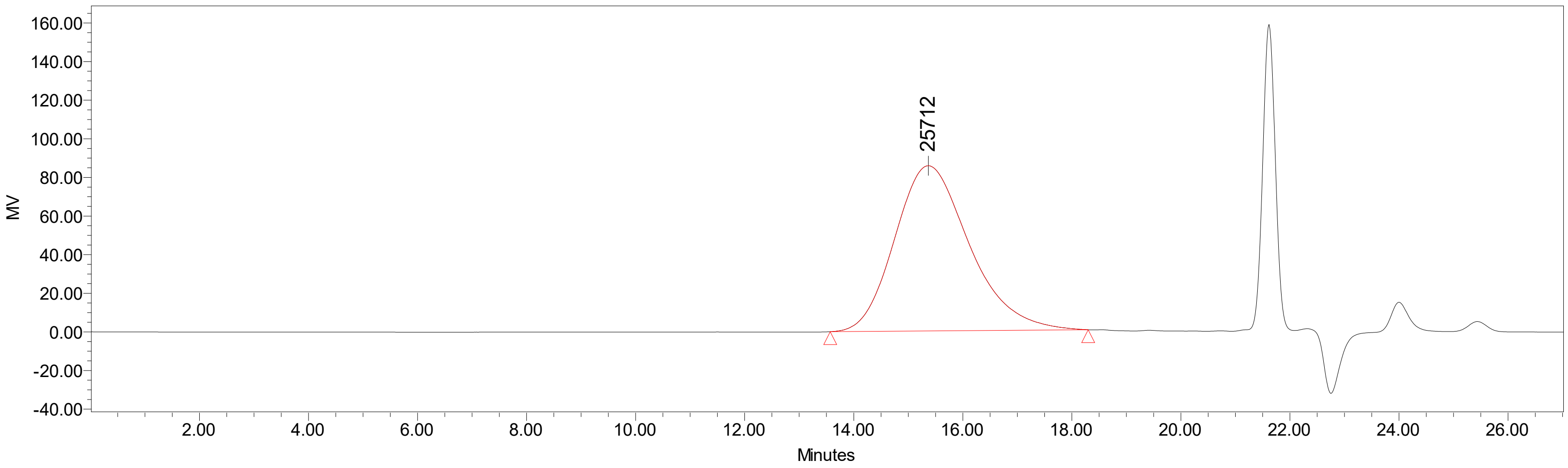

Figure S80 GPC/SEC chromatogram of the PE obtained with catalyst **3***, at 15 bar and 25 ºC.

Dynamic Viscosity Measurements of Selected Polyethylene Samples

The dynamic viscosity (η) of two selected samples of polyethylene obtained in this work were measured using a Brookfield DV-II + Pro rotational viscometer equipped with a CPE-40 cone-plate spindle and integrated temperature control. Measurements followed the principles of ASTM D2196 (cone-plate method) and were performed at 40 ± 0.1 ºC and 100 ± 0.1 ºC. Approximately 0.5 mL of sample was placed on the plate, the cone lowered to the working gap, and any excess material trimmed. Samples were allowed to thermally equilibrate for 20 min prior to measurements. The CPE-40 spindle and target speeds were chosen to maintain instrument torque within 10–90 %. The instrument reported viscosity directly in mPa·s (cP) once the speed was set. Each sample was measured in triplicate, and the results were reported as the mean η value with the corresponding standard error (Table S7). The obtained values may be compared to those reported in the literature.[11]

Table S7 Selected dynamic viscosities (η) of two samples of PE at 40 ± 0.1 ºC and 100 ± 0.1 ºC. The samples were obtained in polymerizations performed at T = 50 and P_{abs}= 9 bar, catalyzed by systems **2*** and **3***, respectively.

**Entry**	**Cat.**	**M_n** (g mol^{-1})	**N** ^{a}	η (mPa s)	
				**40 ºC**	**100 ºC**
18	**2***	6600	123	4400 ± 40	230.5 ± 0.2
28	**3***	10500	98	9440 ± 20	508.9 ± 0.7

^{a} Branches/1000C atoms, determined by ^{1}H NMR (see Table 1 of the article).

Possible Polymerization Mechanistic Scheme

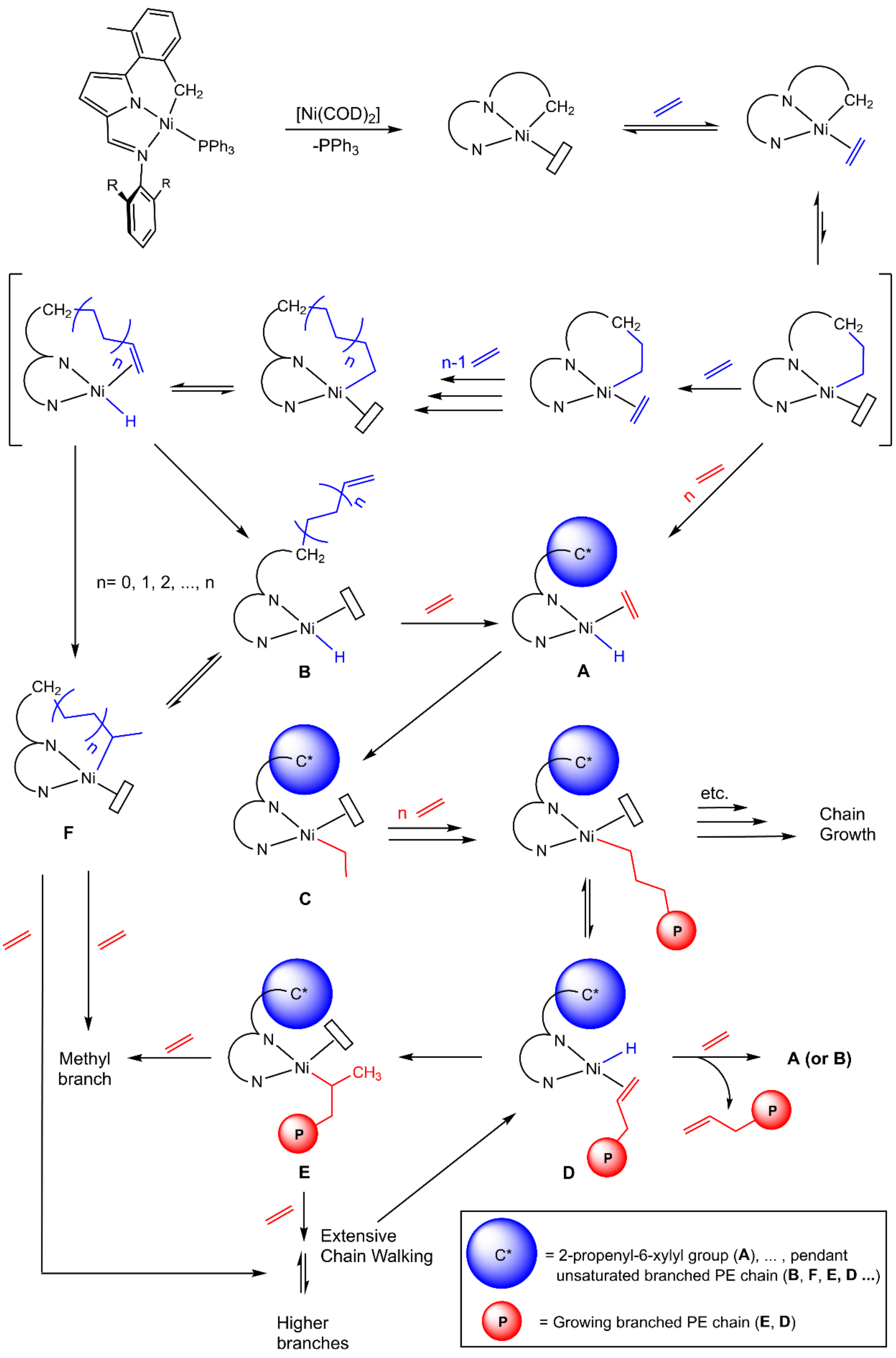

Figure S81 Possible mechanism for the initial steps of ethylene polymerization catalyzed by complexes **1**-**3**, including the transformation of the neutral precatalysts (initiation), followed by chain propagation, chain transfer, and chain-walking (isomerization/branching) steps, consistent with a Brookhart-type mechanism.[12]

NMR Tube-Scale Monitoring of the Reaction of Ethylene with Precatalyst 2

Attempts to investigate the initial insertions of ethylene into the *C,N,N'*-tridentate nickel precatalyst **2** were conducted in an NMR-tube scale experiment under mild conditions (50 ºC and 1 atm ethylene), using an initial ethylene:**2** molar ratio of 7:1. For this purpose, 13 μmol of precatalyst **2** were dissolved in 0.5 mL of benzene-d_6, and the solution was exposed to 88 μmol of ethylene gas. After 25.5 hours of reaction, corresponding to 89% ethylene consumption, the ^{1}H NMR spectra revealed no significant changes in the chemical environment of the precatalyst (see Figure S81). The only noticeable spectral variations over this period were a gradual decrease in the resonance of dissolved ethylene (δ 5.25 ppm) and the appearance of new signals in the ranges δ 5.9–4.9 and 2.1–0.8 ppm, attributable to unsaturated olefinic and aliphatic protons of ethylene oligomers, respectively. Upon extreme magnification of the spectral baseline, a few very weak additional resonances could be observed, though they could not be assigned.

In parallel, ^{1}H DOSY NMR experiments were performed on both pure complex **2** (Figure S82) and the reaction mixture after 25.5 hours at 50 °C. Superimposition of the DOSY spectra of pure **2** and the ethylene-exposed mixture (Figure S83) showed no evidence of species with lower diffusion coefficients (i.e., larger hydrodynamic volumes or larger molecular size) than precatalyst **2**, suggesting that no bulky insertion products were formed. Instead, only faster-diffusing species, consistent with small ethylene oligomers, were clearly detected. Apart from residual dissolved ethylene (with the highest diffusion coefficient), four distinct oligomeric species of increasing size were observed, none of which exceeded the hydrodynamic size of complex **2**.

In conclusion, under the applied conditions (50 °C and 1 atm ethylene), no stoichiometric insertion of ethylene into the Ni-C bond of the metalacycle was detected. Instead, a slow formation of ethylene oligomers occurred. These observations suggest that only a very small fraction of the precatalyst is converted into catalytically active species, which remain below the detection limit of ^{1}H NMR and likely exist in the reaction medium at extremely low concentrations.

These findings preclude experimental validation of the hypothesis that oligo-/polymer chains remain attached to the ligand framework following ethylene insertion, due to the intrinsic nature of the catalyst system, which does not exhibit characteristics of a living polymerization. Nonetheless, this possibility cannot be definitively ruled out.

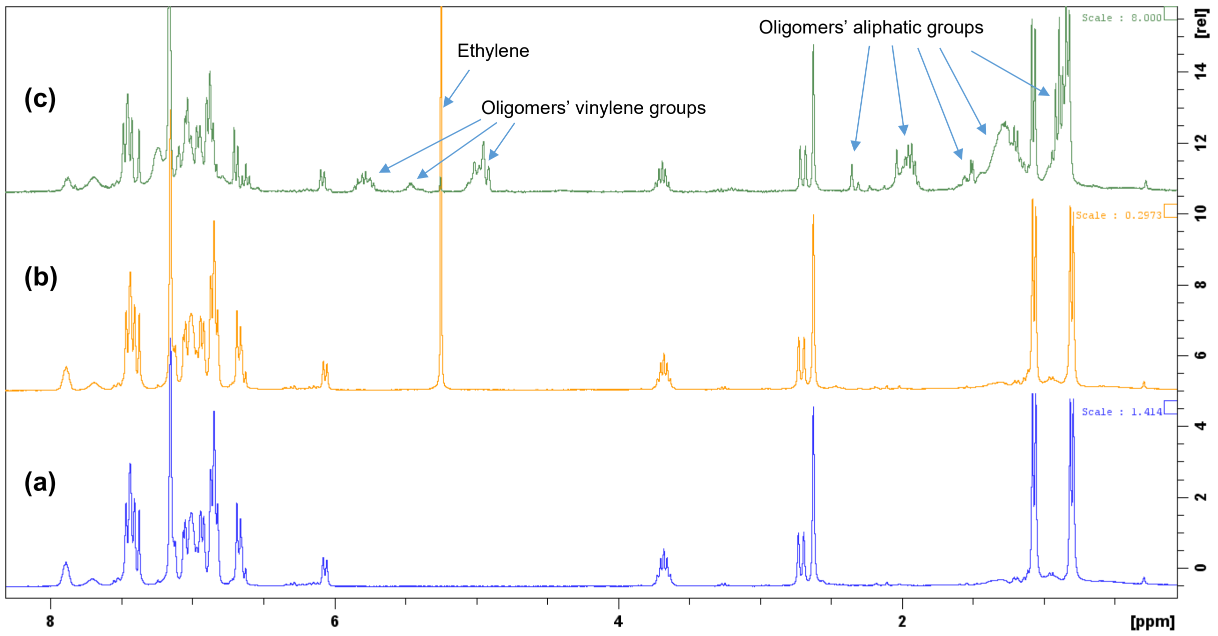

Figure S82 ^{1}H NMR spectra (300 MHz, C_6D_6, 50 ºC) of: (a) pure complex **2** (13 µmol), bottom spectrum (blue); (b) complex **2** in the presence of ethylene (7 equivalents), after 17 min of reaction, middle spectrum (orange); and (c) complex **2** in the presence of ethylene (7 equivalents), after 25.5 h of reaction, top spectrum (green).

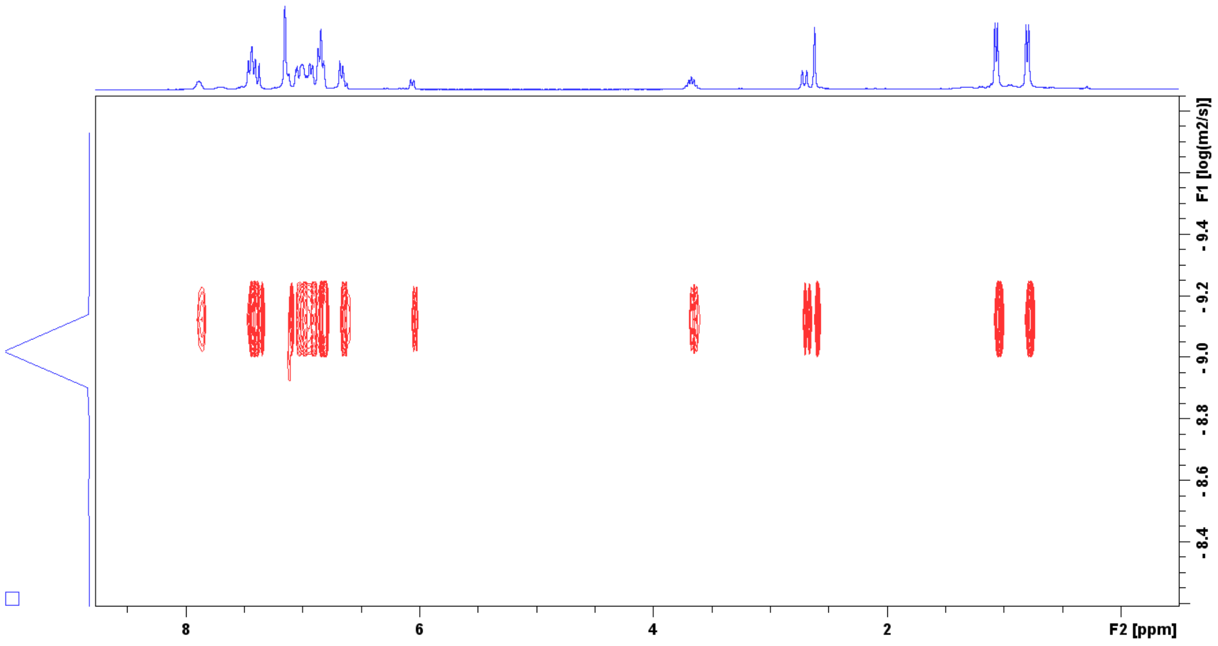

Figure S83 ^{1}H DOSY spectrum (300 MHz, C_6D_6, 50 ºC) of complex **2** (13 µmol), corresponding to ^{1}H NMR spectrum (a) shown in Figure S81. The Y-axis represents $log_{10}(D)$, where D is the self-diffusion coefficient (in m^2/s). For complex **2**, a diffusion coefficient of $D = 5.7 \times 10^{-10}$ m^2/s was determined under these conditions (C_6D_6, 50 ºC).

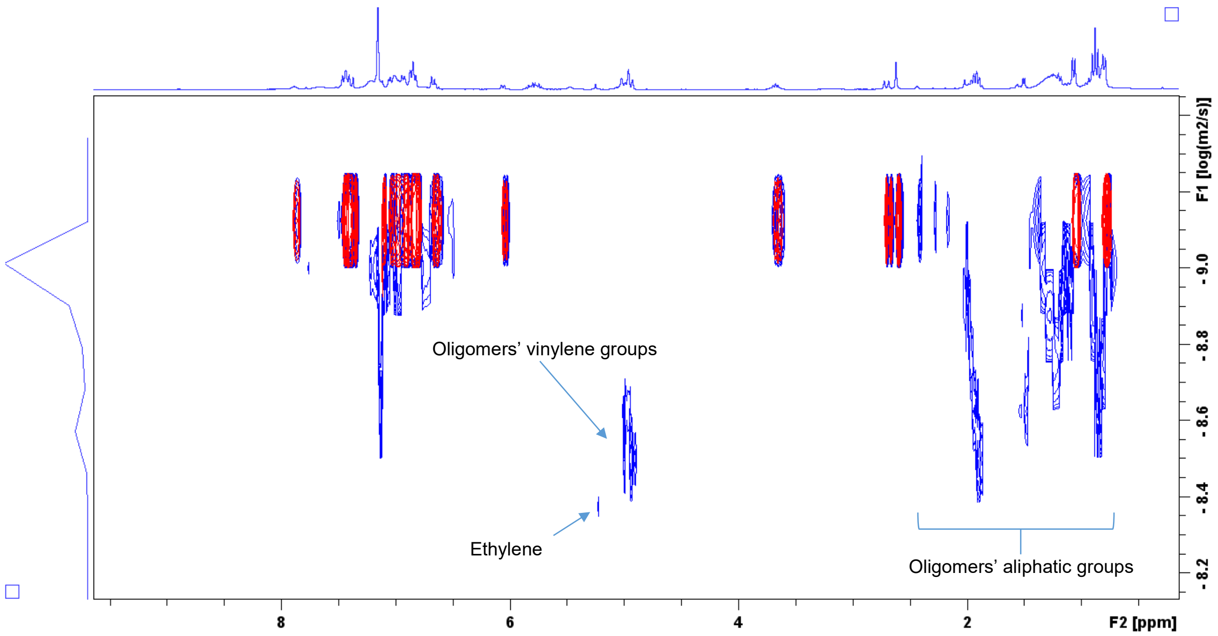

Figure S84 Superimposed ^{1}H DOSY spectra (300 MHz, C_6D_6, 50 ºC) of pure complex **2** (red trace) and of the reaction mixture containing complex **2** and ethylene (7 equivalents), after 25.5 h of reaction (blue trace). No species with lower diffusion coefficients (D) than complex **2** are detected. In contrast, at least five species with higher values of D (ranging from *ca.* 6×10^{-10} to 3×10^{-9} m^2/s), consistent with ethylene oligomers formed during the reaction, are clearly observed. These display signals attributable to unsaturated vinylene protons (δ 5.9–4.9 ppm) and aliphatic protons (δ 2.1–0.8 ppm).

References and Notes

1 Cruz, T. F. C.; Figueira, C. A.; Waerenborgh, J. C.; Pereira, L. C. J.; Li, Y.; Lescouëzec, R.; Gomes, P. T. Synthesis, characterization and magnetism of homoleptic bis(5-aryl-2-iminopyrrolyl) complexes of iron(II) and cobalt(II). *Polyhedron* **2018**, *152*, 179–187. DOI: 10.1016/j.poly.2018.06.026.

2 See Electronic Supplementary Information of: Figueira, C. A.; Lopes, P. S.; Gomes, C. S. B.; Gomes, J. C. S.; Lemos, F.; Gomes, P. T. New phenyl–nickel complexes of bulky 2-iminopyrrolyl chelates: synthesis, characterisation and application as aluminium-free catalysts for the production of hyperbranched polyethylene. *Dalton Trans*. **2018**, *47*, 15857–15872. 10.1039/C8DT02824A.

3 Yang, L.; Powell, D. R.; Houser, R. P. Structural variation in copper(I) complexes with pyridylmethylamide ligands: structural analysis with a new four-coordinate geometry index, τ_4. *Dalton Trans*. **2007**, 955–944. DOI: 10.1039/B617136B.

4 Poater, A.; Ragone, F.; Giudice, S.; Costabile, C.; Dorta, R.; Nolan, S. P.; Cavallo, L. Thermodynamics of N-Heterocyclic Carbene Dimerization: The Balance of Sterics and Electronics. *Organometallics* **2008**, *27*, 2679–2681. DOI: 10.1021/om8001119.

5 (a) Falivene, L.; Cao, Z.; Petta, A.; Serra, L.; Poater, A.; Oliva, R.; Scarano, V.; Cavallo L. Towards the online computer-aided design of catalytic. *Nat. Chem.* **2019**, *11*, 872–879. DOI: 10.1038/s41557-019-0319-5; (b) SambVca 2.1 Application website: https://www.aocdweb.com/OMtools/sambvca2.1/ (last accessed: 15/10/2025).

6 Pettersen, E. F.; Goddard, T. D.; Huang, C. C.; Meng, E. C.; Couch, G. S.; Croll, T. I.; Morris, J. H.; Ferrin, T. E. *Protein Science* **2021**, *30*, 70–82. DOI: 10.1002/pro.3943.

7 Wiedemann, T.; Voit, G.; Tchernook, A.; Roesle, P.; Göttker-Schnetmann, I.; Mecking, S. Monofunctional Hyperbranched Ethylene Oligomers. *J. Am. Chem. Soc.* **2014**, *136*, 2078–2085. DOI: 10.1021/ja411945n.

8 Gottfried, A. C.; Brookhart, M. Living and Block Copolymerization of Ethylene and α-Olefins Using Palladium(II)−α-Diimine Catalysts. *Macromolecules* **2003**, *36*, 3085–3100. DOI: 10.1021/ma025902u.

9 (a) Galland, G. B.; Souza, R. F.; Mauler, R. S.; Nunes, F. F. ^{13}C NMR Determination of the Composition of Linear Low-Density Polyethylene Obtained with [η^3-Methallyl-nickel-diimine]PF_6 Complex. *Macromolecules* **1999**, *32*, 1620–1625. DOI: 10.1021/ma981669h; (b) Liu, W.; Ray III, D. G.; Rinaldi, P. L. Resolution of signals from long-chain branching

in polyethylene by ^{13}C NMR at 188.6 MHz. *Macromolecules* **1999**, *32*, 3817–3819. DOI: 10.1021/ma990009p; (c) Jurkiewicz, A.; Eilerts, N. W.; Hsieh, E. T. ^{13}C NMR Characterization of Short Chain Branches of Nickel Catalyzed Polyethylene. *Macromolecules* **1999**, *32*, 5471–5476. DOI: 10.1021/ma990339+; (d) Cotts, P. M.; Guan, Z.; McCord, E.; McLain, S. Novel Branching Topology in Polyethylenes as Revealed by Light Scattering and ^{13}C NMR. *Macromolecules* **2000**, *33*, 6945–6952, DOI: 10.1021/ma000926r; (e) Galland, G. B.; Quijada, R.; Rojas, R.; Bazan, G.; Komon, Z. J. A. NMR Study of Branched Polyethylenes Obtained with Combined Fe and Zr Catalysts. *Macromolecules* **2002**, *35*, 339–345. DOI: 10.1021/ma010744c; (f) Azoulay, J. D.; Bazan, G. C.; Galland, G. B. Microstructural Characterization of Poly(1-hexene) Obtained Using a Nickel α-Keto-β-diimine Initiator. *Macromolecules* **2010**, *43*, 2794–2800. DOI: 10.1021/ma9025543.

10 Wang, F.; Tanaka, R.; Cai, Z.; Nakayama, Y.; Shiono, T. Synthesis of Highly Branched Polyolefins Using Phenyl Substituted α-Diimine Ni(II) Catalysts. *Polymers*, **2016**, *8*, 160. DOI: 10.3390/polym8040160.

11 Wang, J.; Ye, Z.; Zhu, S. Topology-Engineered Hyperbranched High-Molecular-Weight Polyethylenes as Lubricant Viscosity-Index Improvers of High Shear Stability. *Ind. Eng. Chem. Res.* **2007**, *46*, 1174−1178. DOI: 10.1021/ie0613624.

12 (a) Johnson, L. K.; Killian, C. M.; Brookhart, M. New Pd(II)- and Ni(II)-Based Catalysts for Polymerization of Ethylene and α-Olefins *J. Am. Chem. Soc*. **1995**, *117*, 6414–6415. DOI: 10.1021/ja00128a054; (b) Youkin, T. R.; Connor, E. F.; Henderson, J. I.; Friedrich, S. K.; Grubbs, R. H.; Bansleben, D. A. Neutral, Single-Component Nickel(II) Polyolefin Catalysts That Tolerate Heteroatoms. *Science* **2000**, *287*, 460–462. DOI: 10.1126/science.287.5452.460; (c) Jenkins, J. C.; Brookhart, M. A Mechanistic Investigation of the Polymerization of Ethylene Catalyzed by Neutral Ni(II) Complexes Derived from Bulky Anilinotropone Ligands. *J. Am. Chem. Soc.* **2004**, *126*, 5827–5842. DOI: 10.1021/ja030634; (d) Guan, Z.; Cotts, P. M.; McCord, E. F.; McLain, J. Chain Walking: A New Strategy to Control Polymer Topology. *Science* **1999**, *283*, 2059−2062. DOI: 10.1126/science.283.5410.2059; (e) Svejda, S. A.; Brookhart, M. Ethylene Oligomerization and Propylene Dimerization Using Cationic (α-Diimine)nickel(II) Catalysts. *Organometallics* **1999**, *18*, 65−74. DOI: 10.1021/om980736t; (f) Tempel, D. J.; Johnson, L. K.; Huff, R. L.; White, P. S.; Brookhart, M. Mechanistic Studies of Pd(II)−α-

Diimine-Catalyzed Olefin Polymerizations. *J. Am. Chem. Soc.* **2000**, *122*, 6686−6700. DOI: 10.1021/ja000893v; (g) Chen, C. Designing catalysts for olefin polymerization and copolymerization: beyond electronic and steric tuning. *Nat. Rev. Chem.* **2018**, *2*, 6–14. DOI: 10.1038/s41570-018-0003-0.
